# The Synthesis of Aryl‐β‐*C*‐Glycosides from Native Saccharides

**DOI:** 10.1002/chem.202501216

**Published:** 2025-05-30

**Authors:** Daan V. Bunt, Joey van Looij, Alexander F. Lenze, Viktor Štuhec, Martin D. Witte, Adriaan J. Minnaard

**Affiliations:** ^1^ Stratingh Institute for Chemistry University of Groningen Nijenborgh 7, 9747 AG Groningen The Netherlands

**Keywords:** β‐selective, carbohydrates, C‐glycosides, one‐pot, protecting‐group‐free

## Abstract

The synthesis of *p*‐nitrophenol‐β‐*C*‐glycosides from native mono‐, di‐, and trisaccharides is described. The one‐pot‐two‐step procedure uses water as the solvent and produces exclusively β‐*C*‐glycosides, the desired stereochemistry for most medicinal chemistry applications. The versatility of the approach is illustrated by the synthesis of the nanomolar SGLT2 inhibitor [3‐(4‐ethylbenzyl)phenyl]‐β‐*C*‐glucoside.

## Graphical abstract

Native mono‐, di‐, and trisaccharides are converted into *p*‐nitrophenol‐β‐*C*‐glycosides in a one‐pot reaction using water as the solvent. A sequence of condensation reactions is used to construct the aromatic ring from acyclic precursors. Using this approach, a modular synthesis of SGLT2‐inhibitor scaffolds was developed and applied to a novel protecting‐group‐free synthesis of a nanomolar SGLT2 inhibitor.

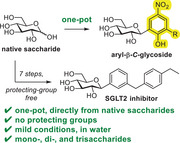

## 1 Introduction

**Figure 1:**
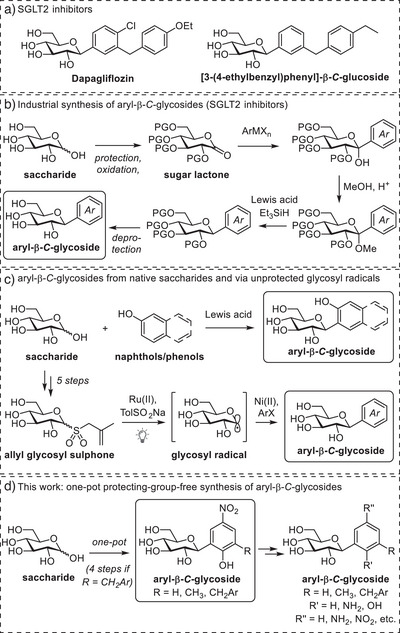
a) Structures of dapagliflozin and [3‐(4‐ethylbenzyl)phenyl]‐β‐*C*‐glucoside, both nanomolar SGLT2‐inhibitors. b) Synthetic routes to aryl‐β‐*C*‐glycosides used in industry. c) Recent protecting‐group‐free approaches toward aryl‐*C*‐glycosides. d) This work: protecting‐group‐free stereoselective synthesis of aryl‐β‐*C*‐glycosides from nonaromatic precursors.

In aryl‐*C*‐glycosides (IUPAC nomenclature: *C*‐glycosidic compounds), the sugar moiety is connected via a carbon‐carbon (C─C) bond to an aromatic residue. In contrast to *O*‐, *N*‐, or *S*‐glycosidic bonds, the *C*‐glycosidic bond is unsusceptible to hydrolysis, and therefore *C*‐glycosides resist glycosidases. Due to this, aryl‐*C*‐glycosides are valuable pharmaceutical scaffolds, with SGLT2 inhibitors such as dapagliflozin as important anti‐diabetic drugs (Figure 1a). As a consequence, synthetic methods for the preparation of aryl‐*C*‐glycosides receive considerable attention.^[^ ^1^, ^2^, ^3^, ^4^, ^5^ ^]^

Because SGLT2 inhibitors and the majority of natural *C*‐glycosides are β‐configured, there is a strong interest in β‐selective *C*‐glycosylation methods. Currently, the predominant strategy for the synthesis of SGLT2 inhibitors revolves around the addition of an aryl organometallic nucleophile onto a protected sugar lactone, followed by ketalization and subsequent stereoselective hydride reduction.^[^ ^6^ ^]^ Although this sequence takes three steps, it is usually preferred over organometallic reactions with glycosyl halides, which tend to produce mixtures of anomers,^[^ ^2^ ^]^ an issue that can be avoided by involving neighboring‐group participation.^[^ ^7^ ^]^ Alternatively, Friedel‐Crafts‐like acid‐mediated *C*‐glycosylations via oxocarbenium intermediates are typically β‐selective, but are restricted to electron‐rich aryl groups and often suffer from poor yields and low regioselectivity.^[^ ^2^ ^]^

Moreover, as free hydroxyl groups are generally not compatible with organometallic reagents and the apolar solvents typically used in the methods discussed, protecting groups are required. For the synthesis of SGLT2 inhibitors, two protection and deprotection cycles of the saccharide moiety are generally necessary.^[^ ^6^ ^]^ The combined protection and deprotection steps significantly impact atom economy and add to the overall environmental impact.

Nevertheless, the number of protecting‐group‐free preparation methods of aryl‐*C*‐glycosides is still severely limited. The earliest reports described the direct use of native saccharides in combination with electron‐rich phenol and naphthol acceptors, catalyzed by TMSOTf or TMSOTf‐AgClO_4_ in CH_3_CN or CH_2_Cl_2_.^[^ ^8^, ^9^, ^10^ ^]^ Under these conditions, the 2,6‐dideoxysaccharides olivose and digitoxose were converted to the corresponding aryl‐*C*‐glycosides in good yields with excellent β‐selectivity, whereas 2‐deoxyglucose could only be used in water with Montmorillonite K‐10 as the acid activator.^[^ ^11^ ^]^ Nevertheless, saccharides with 2‐OH groups were not used in these studies, severely limiting the scope.

This limitation was addressed in later work which employed Sc(OTf)_3_ in EtOH‐water or CH_3_CN‐water mixtures and allowed the direct *C*‐glycosylation of glucose and xylose with phloroacetophenone.^[^ ^12^, ^13^, ^14^ ^]^ Despite the attractive simplicity of this method, mixtures of mono‐ and bis‐*C*‐glycosylated products were obtained. It was demonstrated later that a high selectivity for mono‐*C*‐glycosylated products can be achieved using Pr(OTf)_3_ instead of Sc(OTf)_3_, leading to *C*‐glycosides of several mono‐ and disaccharides with the isoflavone naringenin, in modest yields.^[^ ^15^ ^]^ Whereas glucosyl and galactosyl products were obtained with the β‐configuration, mannosyl, and rhamnosyl products had the α‐configuration.

Currently, no other methods are available to prepare aryl‐*C*‐glycosides directly from native saccharides. Nevertheless, recent efforts in the field of photoredox chemistry have demonstrated that the generation of glycosyl radicals is a promising new approach for *C*‐glycosylation that does not require the use of protecting groups.^[^ ^16^, ^17^ ^]^ By combining photoredox activation with Ni‐catalysis, aryl‐*C*‐glycosides have been prepared in one step from unprotected allyl glycosyl sulphones and aryl iodides.^[^ ^18^ ^]^ The use of a dimethoxy‐bipyridine ligand ensured a preference for 1,2‐*trans*‐configured aryl‐*C*‐glycoside products. Whereas this results in good β‐selectivity for saccharides with equatorially‐configured 2‐OH groups (e.g., glucose, galactose, xylose), axially‐configured 2‐OH saccharides (e.g., mannose and rhamnose) produce mainly α‐configured products. While this is a valuable method to make aryl‐*C*‐glycosides, preparation of the allyl glycosyl sulphone donors from native saccharides requires five steps, including protection and deprotection.

**Figure 2:**
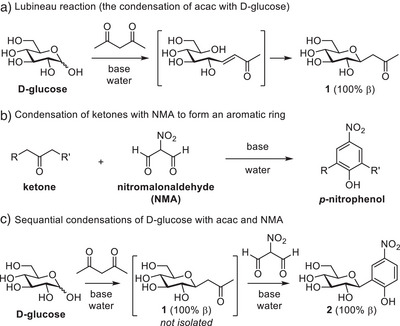
a) The Lubineau reaction, using acetylacetone (acac). b) Construction of aromatic rings from ketones and NMA. c) One‐pot approach using acac and NMA to synthesize an aryl‐β‐*C*‐glucoside.

Contrary to the conventional and intuitive disconnection of the C─C bond between saccharide and aglycon, we decided to base our approach on the Lubineau reaction; the Knoevenagel condensation of aldoses with 1,3‐diketones under basic aqueous conditions (Figure 2a). This established reaction is carried out in water and provides 2‐oxoalkyl‐*C*‐glycosides with excellent β‐selectivity and yield, directly from native saccharides.^[^ ^19^ ^]^ It is also long known that the condensation of dialkyl ketones with malonyl dialdehydes leads to the formation of phenols,^[^ ^20^ ^]^ and these two reactions are now combined to effectively generate the desired β‐configured sp^3^‐sp^2^ bond in aryl‐*C*‐glycosides. We selected readily available nitromalonaldehyde^[^ ^21^, ^22^ ^]^ (NMA) to demonstrate that this approach gives access to various aryl‐*C*‐glycosides, including di‐ and trissaccharides, that are otherwise challenging to obtain. And we demonstrate its applicability in the protecting‐group‐free synthesis of a known SGLT2 inhibitor.

## 2 Results and Discussion

The Lubineau reaction is an unusual but highly efficient approach to synthesize *C*‐glycosides from native saccharides in water. Acetylacetone (acac) is typically used, which undergoes a Knoevenagel condensation with the aldehyde functionality of the open form of the saccharide. The pyranose ring can now reform in an intramolecular conjugate addition on the Knoevenagel adduct, which is followed by a retro‐Claisen fragmentation, eliminating AcOH. This yields a propanone moiety connected to the anomeric carbon via a C─C bond. Reversible elimination of the pyranose ring oxygen ensures that the thermodynamic equatorially configured pyranose product **1** is obtained (Figure 2a).^[^ ^19^ ^]^ Excellent yields are reported for a range of monosaccharides and several disaccharides, often with complete β‐selectivity. Variations in the 1,3‐diketone provide the respective products, but often in moderate yields due to solubility issues.^[^ ^23^, ^24^, ^25^ ^]^

Ketones flanked by two methylene groups can undergo a double condensation reaction with 1,3‐dialdehydes to form aromatic rings.^[^ ^20^ ^]^ NMA is a notable example, which is commercially available and obtained from readily available mucobromic acid.^[^ ^21^ ^]^ It yields the corresponding *p*‐nitrophenols in high yields (Figure 2b).^[^ ^21^, ^22^ ^]^ Both the Lubineau reaction and the reaction with NMA are carried out in water at high pH, which provided the opportunity to combine the reactions in one pot.

The study commenced with the Lubineau reaction of glucose and acac, using NaHCO_3_ as the base.^[^ ^19^ ^]^ Upon completion of the reaction, NMA and NaOH were added, and full conversion of **1** was observed after overnight reaction at room temperature. This was accompanied by the appearance of a bright yellow color, consistent with the characteristic absorption of the *p*‐nitrophenolate anion around 405 nm, which disappeared upon acidification.^[^ ^26^ ^]^ Extraction of the acidified reaction mixture yielded a hygroscopic amorphous solid. ^1^H‐NMR analysis showed the presence of a single glycoside with three new signals in the aromatic region, consistent with an *ortho*‐substituted *p*‐nitrophenol,^[^ ^27^ ^]^ and a *J*_1‐2_ of 9.2 Hz, consistent with β‐configured glucosides.^[^ ^28^ ^]^ These data are consistent with product **2** (Figure 2c), and show that the β‐configuration of intermediate **1** was maintained in the reaction with NMA, despite possible anomerization under basic conditions.^[^ ^29^ ^]^ Besides AcOH, no impurities were present, demonstrating that organic solvents successfully extract and isolate the desired glycosidic product from the aqueous reaction mixture, in a moderate yield of 39% at this stage of the research. This is a significant advantage, considering the difficult isolation of Lubineau products.

A series of other monosaccharides was subjected to the same reaction conditions and mostly provided the respective *p*‐nitrophenol‐*C*‐glycosides via extraction, demonstrating the success of the one‐pot sequence and extractive workup. Nevertheless, several side products could be identified, which were determined to originate from the Lubineau reaction. Galactose, fucose, and arabinose, with identical relative configurations at the 2‐, 3‐, and 4‐positions, can form a stable all‐*trans* β‐furanose‐configured Lubineau product next to the pyranose product. This was found to accumulate over time, consistent with earlier reports.^[^ ^23^ ^]^ These side products reacted with NMA and were extracted along with the desired pyranose‐configured products. Moreover, nearly all reactions formed small amounts of furan‐containing side products, via a cyclization‐dehydration sequence of the Lubineau products.^[^ ^23^ ^]^

In order to reduce the formation of these side products, we used NaOH as the base instead of NaHCO_3_, which allowed a drastic reduction of the reaction time.^[^ ^25^ ^]^ Within 5 hours, most monosaccharides had formed one major β‐pyranose‐configured product. After subsequent reaction with NMA, the products isolated by extraction contained no or minimal amounts of the aforementioned side products.

Nevertheless, the Lubineau reaction of ribose did not converge to one major product, and after reaction with NMA, provided a mixture of products. Fructose, a keto‐saccharide, is known to react sluggishly in the Lubineau reaction,^[^ ^30^ ^]^ and we could not identify the desired *p*‐nitrophenol‐*C*‐fructoside product. The Lubineau reaction of N‐acetylglucosamine (GlcNAc) has been reported to cause epimerization at the C2‐position.^[^ ^31^ ^]^ In our one‐pot procedure, a complex mixture was obtained. All other studied monosaccharides gave extraction products of acceptable purity (Table 1).

**Table 1:** One‐pot procedure for monosaccharides on 1 mmol scale.

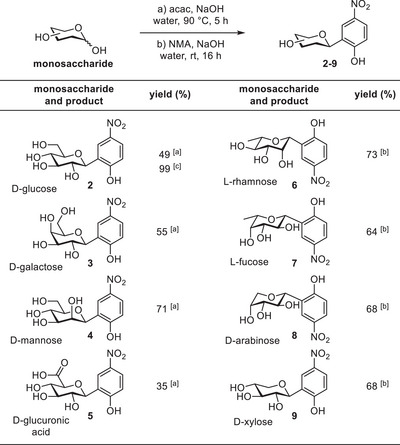

^[a]^ Extracted 3x with 2‐MeTHF.

^[b]^ Extracted 3x with EtOAc.

^[c]^ Extracted 10x with 2‐MeTHF (25 mmol scale).

2‐MeTHF was identified as the most effective extraction solvent. However, for the less polar products of 6‐deoxy and five‐carbon saccharides (**6**‐**9**), EtOAc provided better separation between the desired product and residual intermediate or side products. The yields in Table 1 were obtained after three rounds of extraction with the given solvent. Higher yields were obtained with exhaustive extraction, as was demonstrated in the synthesis of **2** on 25 mmol scale. In this case, 2‐MeTHF was used for 10 extraction rounds, yielding the product in nearly quantitative yield.

The method thus provides ready access to unprotected *p*‐nitrophenol‐β‐*C*‐glycosides of a series of monosaccharides, using a convenient extractive workup. The unique substitution pattern and electron‐poor character of the nitrophenol moiety contrast the densely functionalized and electron‐rich phenols and naphthols typically used in one‐step acid‐mediated aryl‐*C*‐glycoside preparation methods. Since the majority of aryl‐*C*‐glycoside natural products contain the glycosidic bond *ortho* to a phenolic OH group, our method is a valuable addition to the existing procedures, and could be useful in the synthesis of natural product mimics.

Moreover, our method provides the generally desired β‐configured products. This is especially noteworthy for mannose and rhamnose, as known protecting‐group‐free acid‐mediated aryl‐*C*‐glycoside preparation methods yield mannoside and rhamnoside products with the α‐configuration. This is undoubtedly the result of their axially configured 2‐OH groups, which are also known to complicate β‐selective arylations using conventional methods with protected glycosyl donors, due to steric hindrance and/or neighboring group participation.^[^ ^7^, ^32^, ^33^ ^]^

The *J*_1‐2_ of aryl‐*C*‐mannosides in the ^4^C_1_ conformation is 3–5 Hz for the α‐configuration,^[^ ^18^, ^34^, ^35^ ^]^ producing a doublet. The same coupling is ∼1 Hz for the β‐configuration,^[^ ^32^, ^36^ ^]^ which is often interpreted as a broad singlet. For mannoside **4**, a broad singlet is observed for the signal corresponding to 1′‐H, whereas the 2′‐H signal indicates a *J*_1‐2_ of 0.9 Hz. A 1D‐NOESY showed a coupling between 1′‐H and 5′‐H, also indicating a β‐configuration.^[^ ^37^ ^]^ Rhamnoside **6** has the same relative configuration as mannoside **4**, and shows a nearly identical *J*_1‐2_ coupling constant, indicating a β‐configuration for **6**. This strongly indicates that our aryl‐*C*‐glycoside preparation method is not affected by the configuration of the 2‐position and consistently provides β‐configured ^4^C_1_ pyranoside products for the monosaccharides listed in Table 1.

We subsequently extended the one‐pot sequence to various di‐ and trisaccharides (Tables 2, 3). Earlier reports of successful Lubineau reactions with cellobiose, maltose, and lactose indicated an opportunity to prepare their respective aryl‐*C*‐glycosides using our one‐pot procedure.^[^ ^19^, ^23^, ^24^ ^]^ Using NaOH in the Lubineau reaction resulted in complex mixtures. Therefore, NaHCO_3_ was used, which has been successfully used with disaccharides in aforementioned reports. Unexpectedly, the subsequent reaction with NMA and NaOH was significantly slower compared to the monosaccharides, and required several days to form significant amounts of the *p*‐nitrophenol‐*C*‐glycosides. Extraction with 2‐MeTHF was possible in most cases, and allowed for the separation of the desired *p*‐nitrophenol‐*C*‐glycosides from the remaining Lubineau intermediates, but resulted in poor yields (see Supporting Information). Therefore, an alternative workup was developed, in which the acidified reaction mixture was lyophilized, followed by a solid‐liquid extraction using EtOH. This provided a significantly higher recovery of the desired products, compared to extraction with 2‐MeTHF. However, the EtOH extraction was not fully selective, and resulted in contamination of the products with unreacted Lubineau intermediates. These could be successfully separated from the desired products by treatment with NaHSO_3_, presumably forming a charged bisulfite adduct of the ketone,^[^ ^38^ ^]^ which is less soluble in EtOH.

**Table 2:** One‐pot procedure using disaccharides, solid‐liquid extraction with EtOH, yield based on the ratio of product to remaining Lubineau intermediate.

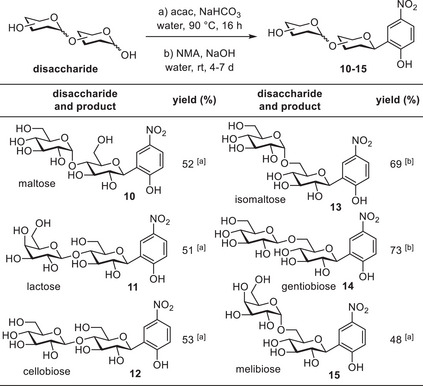

^[a]^ 4‐day reaction time.

^[b]^ 7‐day reaction time.

**Table 3:** One‐pot procedure using trisaccharides, solid‐liquid extraction with EtOH, yield based on the ratio of product to remaining Lubineau intermediate.

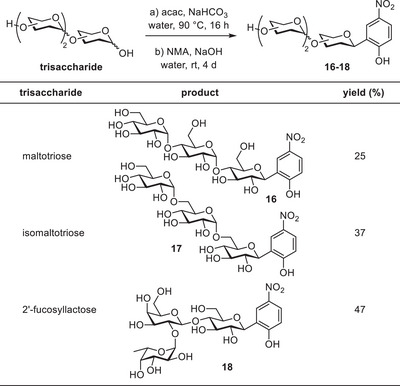

**Figure 3:**
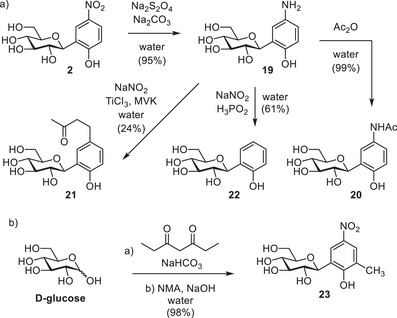
a) Reduction of the nitro group and subsequent transformations of *p*‐aminophenol‐*C*‐glucoside. b) One‐pot procedure using heptanedione.

To expand the scope of our method, subsequent transformations of the *p*‐nitrophenol moiety were investigated starting from **2**. Attempts to hydrogenate the nitro group using heterogeneous catalysts (Pd/C and Raney nickel) led to mixtures of products, but sodium dithionite in basic aqueous medium produced the desired *p*‐aminophenol moiety cleanly (Figure 3a). Isolation of the product (**19**) was achieved by precipitation of the salts and byproducts with EtOH.

Subsequently, the amino group in **19** was converted successfully in several reactions (Figure 3a). Selective acetylation of **19** using acetic anhydride in water produced “paracetamol”‐*C*‐glucoside (**20**). Furthermore, we could generate the aryl diazonium salt from **19**, which was used in a Meerwein arylation with methyl vinyl ketone (MVK) to produce **21**. Reduction of this aryl diazonium salt in situ with hypophosphorous acid generated phenol‐*C*‐glucoside **22**, a compound that surprisingly has not been described in literature. We expect that these transformations are applicable to the full scope of mono‐, di, and trisaccharides presented in Tables 1, 2, 3.

Reaction with heptanedione instead of acac (Figure 3b) is also possible, despite its diminished water solubility. The expected product *o*‐methyl‐*p*‐nitrophenol‐*C*‐glucoside (**23**) formed successfully, showing that extra substituents on the Lubineau product still allow successful aromatization with NMA.

**Figure 4:**
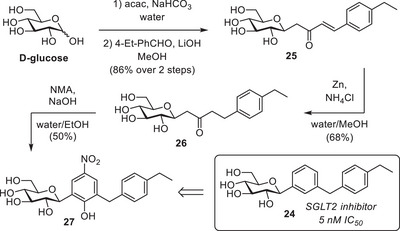
Structure of the target SGLT2 inhibitor and the synthesis of its carbon scaffold via our approach.

In light of the latter result, we saw an opportunity to apply our method for a novel synthesis of the known SGLT2 inhibitor [3‐(4‐ethylbenzyl)phenyl]‐β‐*C*‐glucoside (**24**) (Figure 4).^[^ ^39^, ^40^, ^41^ ^]^ The majority of the marketed SGLT2 inhibitors, including **24**, have a diphenylmethane‐β‐*C*‐glycoside scaffold, in which the methylene linker is positioned *meta* to the *C*‐glycosidic bond. In analogy with the synthesis of **23**, it is possible to achieve this substitution pattern with our current method, but the required 1,3‐diketone would not be soluble in water. Therefore, we designed an alternative synthesis route in which **1** is condensed with 4‐ethylbenzaldehyde followed by hydrogenation of the alkene. Subsequent condensation with NMA would then provide the carbon skeleton of **24**.

The required saturated ketone **26** was obtained from D‐glucose in a sequence of the Lubineau reaction with acac,^[^ ^19^ ^]^ an aldol condensation with 4‐ethylbenzaldehyde using LiOH,^[^ ^42^ ^]^ and a selective reduction of the alkene using zinc.^[^ ^43^, ^44^ ^]^ Subsequently, **26** was condensed with NMA using NaOH in a mixture of water and EtOH, producing nitrophenol **27** with the desired carbon scaffold, which could be isolated via extraction in 50% yield (Figure 4).

To obtain target compound **24**, both the nitro group and the hydroxyl group on the *p*‐nitrophenol ring had to be replaced by hydrogen. The removal of the nitro group by a reduction–deamination sequence had already been demonstrated in the synthesis of **22** (Figure 3a). Therefore, what remained was the removal of the hydroxyl group.

The deoxygenation of a phenol usually requires derivatization, in order to weaken the C─O bond.^[^ ^45^ ^]^ We attempted selective sulfonylation of **27**, but even the small mesyl or triflyl group could not be introduced. An alternative radical‐mediated sulfonylation strategy^[^ ^46^ ^]^ did provide the benzenesulfonate ester, but subsequent reduction attempts^[^ ^47^, ^48^, ^49^, ^50^ ^]^ met with failure. The *ortho*‐substituents apparently prevent the insertion of transition‐metal catalysts into the C─O bond.

Therefore, we considered an alternative strategy, in which the *p*‐nitrophenol moiety is converted into a *p*‐nitroaniline moiety. This C─N bond can then be cleaved via reduction of the corresponding diazonium ion, as was demonstrated in the synthesis of **22** (Figure 3a). The conversion of nitrophenols to nitroanilines is achieved via alkylation of the phenol with haloacetamides, followed by an intramolecular S_N_Ar known as the Smiles rearrangement.^[^ ^51^ ^]^ The latter step is facilitated by a *para*‐nitro substituent.

Surprisingly, when **27** was subjected to the reaction conditions for the initial alkylation (chloroacetamide, KI, K_2_CO_3_ in DMF, at 90 °C),^[^ ^52^, ^53^ ^]^ the subsequent *O* to *N* rearrangement occurred simultaneously, resulting in a 1:1 mixture of **27** and **28**. This despite literature suggesting the Smiles rearrangement requires temperatures up to 150 °C.^[^ ^52^, ^53^ ^]^ This simultaneous rearrangement turned out to be undesirable, however, as conversion of **27** halted as soon as **28** formed, which was determined by Ultra‐high Performance Liquid Chromatography‐Mass Spectrometry (UPLC‐MS) with parallel UV/Vis detection. Full conversion of **27** could not be achieved despite repeated addition of reagents, suggesting **28** or a by‐product of its formation is preventing a reaction between **27** and chloroacetamide.

**Figure 5:**
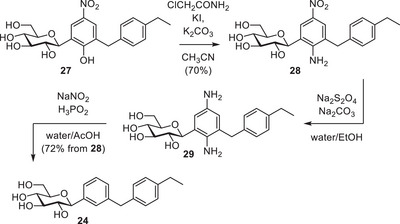
One‐pot Smiles rearrangement and subsequent deamination.

In CH_3_CN, the formation of **28** did not seem to affect the conversion of **27**. Despite the lower boiling point of CH_3_CN compared to DMF, the rearrangement step still proceeded, although significantly more slowly. Additional base helped to push the rearrangement step to completion, yielding *p*‐nitroaniline **28** in 70% yield via extraction (Figure 5).

With the *p*‐nitroaniline moiety in place, the nitro group could be reduced as described earlier (Figure 3a), and the resulting diaminobenzene moiety was reductively deaminated with the H_3_PO_2_/NaNO_2_ combination used in the synthesis of **22** (Figure 3a). The final product **24** was obtained in 7 steps from D‐glucose, and the synthesis required neither protecting groups nor column chromatography, demonstrating the broader applicability of this approach for the construction of aryl‐*C*‐glycosides.

It is important to note that in this approach, the synthesis of SGLT2 inhibitors is essentially modular, and both the saccharide and the aromatic aldehyde, like 4‐ethylbenzaldehyde can be varied. Combined with the transformations of the *p*‐nitrophenol moiety demonstrated in this work, a library of novel SGLT2 inhibitor candidates can be readily constructed.

## 3 Conclusion

We developed a one‐pot, protecting‐group‐free method for the synthesis of *p*‐nitrophenol‐β‐*C*‐glycosides, directly from native mono‐, di‐, or trisaccharides. The unique substitution pattern obtained via our approach is not readily accessible via conventional C‐glycosylation approaches, and provides a valuable addition to existing acid‐mediated methods using native saccharides with densely functionalized and electron‐rich phenols and naphthols. Moreover, products are obtained exclusively, or highly enriched, in the β‐configuration, including *C*‐mannoside **4** and *C*‐rhamnoside **6**, whereas other methods mainly yield the respective α‐configured products. A brief exploration into subsequent protecting‐group‐free transformations was performed using compound **2**, yielding several other previously unavailable aryl‐*C*‐glycosides. Variation of the 1,3‐diketone was demonstrated in the synthesis of **23**, which can be used to introduce *ortho* substituents. Finally, the applicability of the concept of aromatic ring construction for aryl‐*C*‐glycoside synthesis was demonstrated by the successful synthesis of SGLT2 inhibitor **24**. This resulted in an attractive synthetic route that does not require protecting groups or column chromatography. Due to the modular nature of this approach for SGLT2 inhibitor synthesis, we expect it has applicability for the facile synthesis of various previously inaccessible potential SGLT2 inhibitors.

## Supporting Information

Experimental procedures and characterization data of all new compounds are provided in the Supporting Information. The authors have cited additional references within the Supporting Information.^[^ ^54^, ^55^ ^]^

## Conflict of Interests

The authors declare no conflict of interest.

## Supporting information



Supporting Information

## Data Availability

The data that support the findings of this study are available in the supplementary material of this article.
