# Supplementary material for: The Synthesis of Aryl‐β‐C‐Glycosides from Native Saccharides
Source: Chemistry. 2025 May 30;31(37):e202501216. doi: 10.1002/chem.202501216 (PMC12223352; doi:10.1002/chem.202501216)
Supplement: Supplementary file 1 — Supporting Information [file CHEM-31-e202501216-s001.pdf]

The Synthesis of Aryl-β-*C*-Glycosides from Native Saccharides

Supporting Information

Daan V. Bunt, Joey van Looij, Alexander F. Lenze, Viktor Štuhec, Martin D. Witte*, and Adriaan J. Minnaard*

Stratingh Institute for Chemistry, University of Groningen, Nijenborgh 7, 9747 AG, Groningen, The Netherlands.
E-mail: a.j.minnaard@rug.nl, m.d.witte@rug.nl

Table of Contents

General Information 2

Experimental procedures and characterization data 2

References 14

Spectral data per compound (**2-28**) 15

General Information

Reactions were performed under ambient conditions and exposed to air, unless specified otherwise. TLC analysis was performed using silica gel 60/Kieselguhr F254, 0.25 mm (Merck) and compounds were visualized using 254 nm UV light, Seebach stain[1], $KMnO_4$ stain, or with ammonia vapor (deprotonation of the *p*NP). ^{1}H and ^{13}C NMR spectra were recorded on an Agilent 400 NMR spectrometer at 400 and 100.59 MHz, respectively, using CD_3OD as the solvent. Chemical shifts are reported in ppm with the solvent resonance as the internal standard (for CD_3OD δ 3.31 ppm for ^{1}H, δ 49.00 ppm for ^{13}C). Specific rotations were determined with a Schmidt + Haensch Polartronic MH8 polarimeter in a 100 mm path-length cell. IR spectroscopic analyses were done using a Perkin-Elmer Spectrum Two UATR FT-IR spectrometer. UV/vis spectra were recorded using a Shimadzu LC-20 series UV/vis detector with a demineralized water/acetonitrile + 0.1% FA mobile phase. UPLC/UV-MS was performed using a ThermoFisher Scientific Vanquish UPLC System with UV-Vis detector in combination with an LCQ Fleet mass spectrometer, using a poroshell 120 EC C18, 1.9 μm, 2,1x 50 mm column. High resolution mass spectra (HRMS) were recorded on a ThermoScientific LTQ Orbitrap XL mass spectrometer with electron spray ionization (ESI) or atmospheric pressure chemical ionization (APCI) in positive or negative mode. Prior to the extractions, 2-MeTHF was freshly distilled to remove the stabilizer. Note: the distilled 2-MeTHF should be used directly. Storage of 2-MeTHF leads to the formation of organoperoxides.

Experimental procedures and characterization data

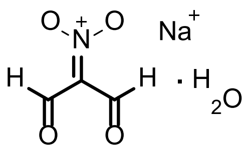

NaNMA

Sodium nitromalonaldehyde monohydrate (NaNMA). According to a literature procedure[2]. ^{1}H NMR (400 MHz, CD_3OD) δ 9.78 (s, 2H). ^{13}C NMR (101 MHz, CD_3OD) δ 183.88, 133.62. HR-MS (ESI): m/z: calcd for $C_3H_2NO_4$ ($[M-H]^-$): 115.9989, found: 115.9989.

General procedure A

Acetylacetone (100 mg, 1.00 mmol), saccharide (1.05 mmol, 1.05 eq.) and NaOH solution (2 M, 0.6 mL, 1.2 eq.) were combined and 0.4 mL water was added. The mixture was stirred in a sealed vial at 90 °C for 5 h, after which it was allowed to cool to room temperature. NaOH solution (2 M, 0.6 mL, 1.2 eq.) and NaNMA (173 mg, 1.10 mmol, 1.1 eq.) were added and the mixture was stirred in a sealed vial at room temperature for 16 h, after which the pH was adjusted to 3 using $NaHSO_4$ (300 mg, 2.50 mmol, 2.5 eq.). The resulting mixture was extracted with freshly distilled 2-MeTHF or EtOAc (3 x 15 mL), dried with anhydrous Na_2SO_4, filtered and concentrated. Residual acetic acid was co-evaporated using *n*-heptane.

General procedure B

Acetylacetone (100 mg, 1.00 mmol), saccharide (1.05 mmol, 1.05 eq.) and $NaHCO_3$ (101 mg, 1.20 mmol, 1.2 eq.) were dissolved in 1 mL water and stirred at 90 °C for 16 h, after which it was allowed to

cool to room temperature. NaOH solution (2 M, 0.75 mL, 1.5 eq.) and NaNMA (173 mg, 1.10 mmol, 1.1 eq.) were added and the mixture was stirred in a sealed vial at room temperature for 24 h, after which the pH was adjusted to 3 using $NaHSO_4$ (300 mg, 2.50 mmol, 2.5 eq.). The resulting mixture was extracted with freshly distilled 2-MeTHF (3 x 15 mL), dried with anhydrous Na_2SO_4, filtered and concentrated. Residual acetic acid was co-evaporated using *n*-heptane.

General procedure C

Acetylacetone (100 mg, 1.00 mmol), saccharide (1.05 mmol, 1.05 eq.) and $NaHCO_3$ (101 mg, 1.20 mmol, 1.2 eq.) were dissolved in 1 mL water and stirred at 90 °C for 16 h, after which it was allowed to cool to room temperature. NaOH solution (2 M, 0.75 mL, 1.5 eq.) and NaNMA (173 mg, 1.10 mmol, 1.1 eq.) were added and the mixture was stirred in a sealed vial at room temperature for 4-7 days, after which the pH was adjusted to 3 using $NaHSO_4$ (300 mg, 2.50 mmol, 2.5 eq.). The resulting mixture was lyophilized and EtOH was added. Insoluble material was removed by filtration over Celite, after which the filtrate was concentrated and analyzed by ^{1}H-NMR to determine the yield via integration of the desired product and remaining intermediate. Remaining glycosyl propanone intermediate can be removed by adding a minimal amount of sat. aq. $NaHSO_3$ solution and stirring the mixture overnight. The mixture is then diluted with EtOH until no more precipitation occurs, filtered, and concentrated to yield the purified product.

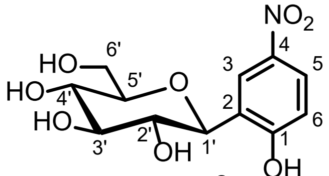

2

***p*-nitrophenol-β-D-*C*-glucoside (2).** Prepared using D-glucose according to general procedure A. Extracted with 2-MeTHF. Obtained as a beige amorphous solid (146 mg, 49%) of reasonable purity. An analytically pure sample was obtained by silica gel column chromatography using an EtOAc/EtOH mixture.

On 25 mmol scale: Prepared using D-glucose according to general procedure B. Acidification was done using 2 M aq. HCl, and extraction was carried out with 2-MeTHF (10 x 100 mL). This yielded a beige amorphous solid (7.52 g, >99%). R_f=0.56 (10 % EtOH in EtOAc). $[\alpha]_D^{20}$=+60 (*c*=0.10 in methanol). ^{1}H NMR (400 MHz, CD_3OD) δ 8.30 (d, *J* = 2.8 Hz, 1H, 3-H), 8.06 (dd, *J* = 8.9, 2.9 Hz, 1H, 5-H), 6.90 (d, *J* = 9.0 Hz, 1H, 6-H), 4.69 (d, *J* = 8.8 Hz, 1H, 1'-H), 3.90 (d, *J* = 12.0 Hz, 1H, 6'-H), 3.73 (dd, *J* = 12.2, 4.4 Hz, 1H, 6'-H), 3.58 – 3.42 (m, 4H, 2'-5'-H).^{13}C NMR (101 MHz, CD_3OD) δ 163.27 (C1), 141.83 (C4), 128.38 (C2), 125.95 (C5), 125.86 (C3), 116.77 (C6), 82.54 (C5'), 79.82 (C3'), 76.57 (C1'), 76.32 (C2'), 71.81 (C4'), 62.99 (C6'). IR (ATR, cm^{-1}): $\tilde{\nu}$ = 3305 (br) (O-H), 2921 (m) (C-H), 1593 (s) (NO_2), 1337 (s) (NO_2), 1284 (s) (C-O). UV/Vis: λ_{max} = 317 nm. HR-MS (ESI): m/z: calcd for $C_{12}H_{14}NO_8$ ($[M-H]^-$): 300.0725, found: 300.0724.

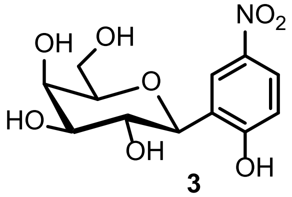

3

***p*-nitrophenol-β-D-*C*-galactoside (3).** Prepared using D-galactose according to general procedure A. Extracted with 2-MeTHF. Obtained as a beige amorphous solid (167 mg, 55%) of reasonable purity. An analytically pure sample was obtained by silica gel column chromatography using an EtOAc/EtOH mixture. R_f=0.51 (10 % EtOH in EtOAc). $[\alpha]_D^{20}$=+78 (*c*=0.10 in methanol). ^{1}H NMR (400 MHz, CD_3OD) δ 8.33 (d, *J* = 3.0 Hz, 1H, 3-H), 7.94 (dd, *J* = 9.1, 3.1 Hz, 1H, 5-H), 6.56 (d, *J* = 9.1 Hz,

1H, 6-H), 4.62 (d, *J* = 9.4 Hz, 1H, 1'-H), 3.95 (dd, *J* = 3.2, 0.6 Hz, 1H, 4'-H), 3.84 – 3.80 (m, 1H, 2'-H), 3.80 – 3.71 (m, 2H, 6-H_2), 3.68 – 3.65 (m, 1H, 5'-H), 3.61 (dd, *J* = 9.3, 3.3 Hz, 1H, 3'-H).^{13}C NMR (101 MHz, CD_3OD) δ 163.26 (C1), 141.87 (C4), 128.46 (C2), 126.22 (C3), 125.94 (C5), 116.85 (C6), 81.10 (C5'), 77.50 (C1'), 76.44 (C3'), 73.30 (C2'), 70.92 (C4'), 62.87 (C6'). IR (ATR, cm^{-1}): $\tilde{v}$ = 3271 (br) (O-H), 2916 (w) (C-H), 1593 (s) (NO_2), 1275 (s) (C-O). UV/Vis: λ_{max} = 318 nm. HR-MS (ESI): m/z: calcd for $C_{12}H_{14}NO_8$ ($[M-H]^-$): 300.0725, found: 300.0717.

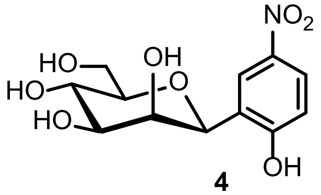

4

***p*-nitrophenol-β-D-*C*-mannoside (4).** Prepared using D-mannose according to general procedure A. Extracted with 2-MeTHF. Obtained as a beige amorphous solid (215 mg, 71%). R_f=0.53 (10 % EtOH in EtOAc). $[\alpha]_D^{20}$=+54 (*c*=0.10 in methanol). ^{1}H NMR (400 MHz, CD_3OD) δ 8.43 (d, *J* = 2.9, 1H, 3-H), 8.04 (dd, *J* = 8.9, 2.9 Hz, 1H, 5-H), 6.88 (d, *J* = 8.9 Hz, 1H, 6-H), 4.85 (br s, 1H, 1'-H), 4.13 (dd, *J* = 3.0, 0.9 Hz, 1H, 2'-H), 3.97 (dd, *J* = 11.9, 2.3 Hz, 1H, 6'-H), 3.85 (dd, *J* = 11.9, 5.9 Hz, 1H, 6'-H), 3.73 – 3.64 (m, 2H, 3'/4'-H), 3.41 (ddd, *J* = 8.7, 5.9, 2.0 Hz, 1H, 5'-H). ^{13}C NMR (101 MHz, CD_3OD) δ 160.92 (C1), 141.77 (C4), 127.89 (C2), 126.16 (C3), 125.37 (C5), 115.56 (C6), 82.73 (C5'), 76.50 (C1'), 76.47 (C3'), 71.46 (C2'), 68.70 (C4'), 63.15 (C6'). IR (ATR, cm^{-1}): $\tilde{v}$ = 3307 (br) (O-H), 2925 (m) (C-H), 1593 (s) (NO_2), 1334 (s) (NO_2), 1281 (s) (C-O). UV/Vis: λ_{max} = 320 nm. HR-MS (ESI): m/z: calcd for $C_{12}H_{14}NO_8$ ($[M-H]^-$): 300.0725, found: 300.0726.

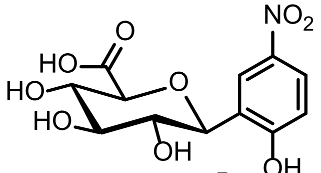

5

***p*-nitrophenol-β-D-*C*-glucuronide (5).** Prepared using D-glucuronic acid according to an adapted version of general procedure A, using one additional equivalent of base to neutralize the carboxylate, and one additional equivalent of acid in the workup. Extracted with 2-MeTHF. Obtained as an orange amorphous solid (109 mg, 35%) of reasonable purity. An analytically pure sample was obtained by silica gel column chromatography using an EtOAc/EtOH mixture. R_f=0.14 (10 % EtOH in EtOAc). $[\alpha]_D^{20}$=+18 (*c*=0.10 in methanol). ^{1}H NMR (400 MHz, CD_3OD) δ 8.32 (d, *J* = 2.8 Hz, 1H, 3-H), 8.08 (dd, *J* = 9.0, 2.8 Hz, 1H, 5-H), 6.94 (d, *J* = 9.0 Hz, 1H, 6-H), 4.74 (d, *J* = 9.0 Hz, 1H, 1'-H), 3.92 (d, *J* = 9.7 Hz, 1H, 5'-H), 3.68 (t, *J* = 9.2 Hz, 1H, 4'-H), 3.62 – 3.50 (m, 2H, 2'/3'-H). ^{13}C NMR (101 MHz, CD_3OD) δ 173.23 (C6'), 163.11 (C1), 141.93 (C4), 127.49 (C2), 126.17 (C5), 125.88 (C3), 116.73 (C6), 80.93 (C5'), 79.24 (C3'), 76.66 (C1'), 75.68 (C2'), 73.44 (C4'). IR (ATR, cm^{-1}): $\tilde{v}$ = 3356 (br) (O-H), 2921 (m) (C-H),2474 (br) (O-H), 1715 (s) (C=O), 1591 (s) (NO_2), 1337 (s) (NO_2), 1282 (s) (C-O). UV/Vis: λ_{max} = 317 nm. HR-MS (ESI): m/z: calcd for $C_{12}H_{12}NO_9$ ($[M-H]^-$): 314.0518, found: 314.0516.

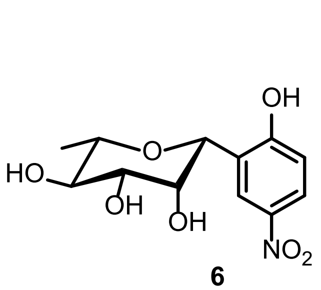

6

***p*-nitrophenol-β-L-*C*-rhamnoside (6).** Prepared using L-rhamnose monohydrate according to general procedure A. Extracted with EtOAc. Obtained as a beige amorphous solid (208 mg, 73%). R_f=0.71 (10 % EtOH in EtOAc). $[\alpha]_D^{20}$=+30 (*c*=0.10 in methanol). ^{1}H NMR (400 MHz, CD_3OD) δ 8.34 (d, *J* = 2.9, 1H, 3-H), 8.04 (dd, *J* = 8.9, 2.9 Hz, 1H, 5-H), 6.87 (d, *J* = 8.9 Hz, 1H, 6-H), 4.81 (br s, 1H, 1'-H), 4.13 (dd, *J* = 3.4, 0.9 Hz, 1H, 2'-H), 3.61 (dd, *J* = 9.0, 3.4 Hz, 1H, 3'-H), 3.49 – 3.37 (m, 2H, 4'/5'-H), 1.42 (d, *J* = 5.8 Hz, 3H, 6-H_3). ^{13}C NMR (101 MHz, CD_3OD) δ 160.88 (C1), 141.73 (C4), 128.09 (C2), 125.99 (C3), 125.30 (C5), 115.54 (C6), 78.08 (C5'),

76.30 (C1'), 76.20 (C3'), 74.03 (C4'), 71.48 (C2'), 18.38 (C6'). IR (ATR, cm^{-1}): ṽ = 3340 (br) (O-H), 2922 (m) (C-H), 2852 (m) (C-H) 1592 (s) (NO_2), 1333 (s) (NO_2), 1274 (s) (C-O). UV/Vis: λ_{max} = 320 nm. HR-MS (APCI): m/z: calcd for $C_{12}H_{16}NO_7$ ($[M+H]^+$): 286.0921, found: 286.0922.

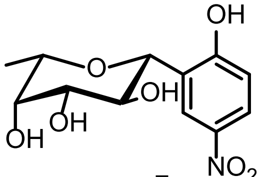

7

***p*-nitrophenol-β-L-*C*-fucoside (7).** Prepared using L-fucose according to general procedure A. Extracted with EtOAc. Obtained as a beige amorphous solid (182 mg, 64%) of reasonable purity. An analytically pure sample was obtained by silica gel column chromatography using an EtOAc/EtOH mixture. R_f=0.71 (10 % EtOH in EtOAc). $[\alpha]_D^{20}$=−52 (*c*=0.10 in methanol). ^{1}H NMR (400 MHz, CD_3OD) δ 8.37 (d, *J* = 2.9 Hz, 1H, 3-H), 8.04 (dd, *J* = 9.0, 3.0 Hz, 1H, 5-H), 6.82 (d, *J* = 9.1 Hz, 1H, 6-H), 4.59 (d, *J* = 9.6 Hz, 1H, 1'-H), 3.85 – 3.71 (m, 3H, 2'/4'/5'-H), 3.63 (dd, *J* = 9.3, 3.3 Hz, 1H, 3'-H), 1.31 (d, *J* = 6.5 Hz, 3H, 6'-H_3).^{13}C NMR (101 MHz, CD_3OD) δ 163.16 (C1), 141.88 (C4), 128.39 (C2), 126.21 (C3), 125.92 (C5), 116.84 (C6), 77.49 (C1'), 76.56 (C3'), 76.41 (C4'/5'), 73.57 (C4'/5'), 72.82 (C2'), 17.21 (C6'). IR (ATR, cm^{-1}): ṽ = 3307 (br) (O-H), 2926 (m) (C-H), 1589 (s) (NO_2), 1338 (s) (NO_2), 1266 (s) (C-O). UV/Vis: λ_{max} = 317 nm. HR-MS (ESI): m/z: calcd for $C_{12}H_{14}NO_7$ ($[M-H]^-$): 284.0776, found: 284.0770.

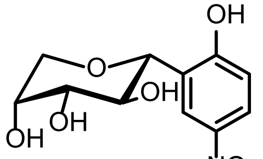

8

***p*-nitrophenol-β-D-*C*-arabinoside (8).** Prepared using D-arabinose according to general procedure A. Extracted with EtOAc. Obtained as a beige amorphous solid (185 mg, 68%) of reasonable purity. An analytically pure sample was obtained by silica gel column chromatography using an EtOAc/EtOH mixture. R_f=0.69 (10 % EtOH in EtOAc). $[\alpha]_D^{20}$=+56 (*c*=0.10 in methanol). ^{1}H NMR (400 MHz, CD_3OD) δ 8.34 (d, *J* = 3.0 Hz, 1H, 3-H), 8.01 (dd, *J* = 9.0, 3.0 Hz, 1H, 5-H), 6.76 (d, *J* = 9.2 Hz, 1H, 6-H), 4.54 (d, *J* = 9.4 Hz, 1H, 1'-H), 4.03 (dd, *J* = 12.5, 1.7 Hz, 1H, 5'-H), 3.97 – 3.92 (m, 1H, 4'-H), 3.84 (t, *J* = 9.5 Hz, 1H, 2'-H), 3.74 (d, *J* = 12.3 Hz, 1H, 5'-H), 3.65 (dd, *J* = 9.5, 3.0 Hz, 1H, 3'-H). ^{13}C NMR (101 MHz, CD_3OD) δ 163.16 (C1), 141.85 (C4), 128.32 (C2), 126.03 (C3), 125.96 (C5), 116.82 (C6), 77.80 (C1'), 75.83 (C3'), 73.13 (C2'), 72.05 (C5'), 70.87 (C4'). IR (ATR, cm^{-1}): ṽ = 3324 (br) (O-H), 2921 (m) (C-H), 1592 (s) (NO_2), 1337 (s) (NO_2), 1275 (s) (C-O). UV/Vis: λ_{max} = 318 nm. HR-MS (ESI): m/z: calcd for $C_{11}H_{12}NO_7$ ($[M-H]^-$): 270.0619, found: 270.0622.

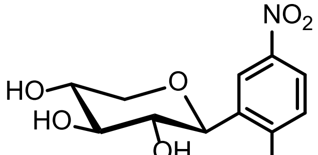

9

***p*-nitrophenol β-D-*C*-xyloside (9).** Prepared using D-xylose according to general procedure A. Extracted with EtOAc. Obtained as a beige amorphous solid (184 mg, 68%) of reasonable purity. An analytically pure sample was obtained by silica gel column chromatography using an EtOAc/EtOH mixture. R_f=0.66 (10 % EtOH in EtOAc). $[\alpha]_D^{20}$=+26 (*c*=0.10 in methanol). ^{1}H NMR (400 MHz, CD_3OD) δ 8.18 (d, *J* = 2.8 Hz, 1H, 3-H), 7.99 (dd, *J* = 9.1, 3.0 Hz, 1H, 5-H), 6.71 (d, *J* = 9.1 Hz, 1H, 6-H), 4.61 (d, *J* = 9.2 Hz, 1H, 1'-H), 4.02 (dd, *J* = 11.0, 5.4 Hz, 1H, 5'-H), 3.62 (ddd, *J* = 10.5, 8.6, 5.5 Hz, 1H, 4'-H), 3.47 (t, *J* = 8.6 Hz, 1H, 2'-H), 3.41 (t, *J* = 9.0 Hz, 1H, 3'-H), 3.36 (t, *J* = 10.4 Hz, 1H, 5'-H). ^{13}C NMR (101 MHz, CD_3OD) δ 163.23 (C1), 141.76 (C4), 128.19 (C2), 126.04 (C5), 125.60 (C3), 116.76 (C6), 79.86 (C3'), 77.41 (C1'), 76.08 (C2'), 71.56 (C5'), 71.44 (C4'). IR (ATR, cm^{-1}): ṽ = 3305 (br) (O-H), 2922 (m) (C-H), 2853 (m) (C-H), 1591 (s) (NO_2), 1337 (s) (NO_2), 1274 (s) (C-O).UV/Vis: λ_{max} = 317 nm. HR-MS (ESI): m/z: calcd for $C_{11}H_{12}NO_7$ ($[M-H]^-$): 270.0619, found: 270.0612.

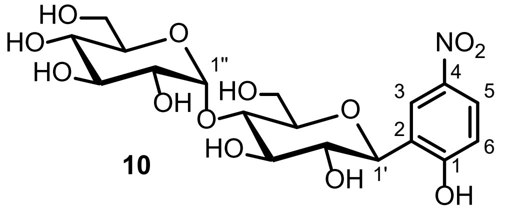

10

***p*-nitrophenol β-D-*C*-maltoside (10).** Prepared using D-maltose monohydrate according to general procedure B. Extracted with 2-MeTHF. Obtained as a beige amorphous solid (141 mg, 30%). According to general procedure C: stirred for 4 d after NaNMA addition. 52% NMR yield. R_f=0.69 (40 % EtOH, 1% AcOH in EtOAc). $[\alpha]_D^{20}$=+62 (*c*=0.10 in methanol). ^{1}H NMR (400 MHz, CD_3OD) δ 8.32 (d, *J* = 2.9 Hz, 1H, 3-H), 8.07 (dd, *J* = 9.1, 2.9 Hz, 1H, 5-H), 6.92 (d, *J* = 9.0 Hz, 1H, 6-H), 5.24 (d, *J* = 3.8 Hz, 1H, 1''-H), 4.70 (d, *J* = 9.7 Hz, 1H, 1'-H), 3.91 (dd, *J* = 12.3, 2.2 Hz, 1H), 3.89 – 3.82 (m, 2H), 3.79 (t, *J* = 8.9 Hz, 1H), 3.76 – 3.63 (m, 4H), 3.61 – 3.50 (m, 2H), 3.47 (dd, *J* = 9.7, 3.7 Hz, 1H), 3.31-3.27 (m, 1H). ^{13}C NMR (101 MHz, CD_3OD) δ 163.29 (C1), 141.86 (C4), 128.18 (C2), 126.03 (C5), 125.94 (C3), 116.75 (C6), 102.99 (C1''), 81.51, 81.14, 79.62, 76.53 (C1'), 75.89, 75.14, 74.81, 74.30, 71.56, 62.78, 62.44. IR (ATR, cm^{-1}): $\tilde{\nu}$ = 3289 (br) (O-H), 2923 (m) (C-H), 1568 (s) (NO_2), 1337 (s) (NO_2), 1288 (s) (C-O). UV/Vis: λ_{max} = 317 nm. HR-MS (ESI): m/z: calcd for $C_{18}H_{24}NO_{13}$ ([M-H]$^-$): 462.1253, found: 462.1253.

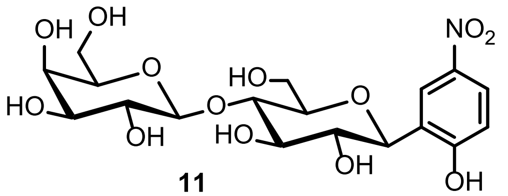

11

***p*-nitrophenol β-D-*C*-lactoside (11).** Prepared using D-lactose monohydrate according to general procedure B. Extracted with freshly distilled 2-MeTHF. Obtained as a beige amorphous solid (70 mg, 15%). According to general procedure C: stirred for 4 d after NaNMA addition. 51% NMR yield. R_f=0.56 (40 % EtOH, 1% AcOH in EtOAc). $[\alpha]_D^{20}$=+34 (*c*=0.10 in methanol). ^{1}H NMR (400 MHz, CD_3OD) δ 8.32 (d, *J* = 2.9 Hz, 1H, 3-H), 8.07 (dd, *J* = 9.0, 2.9 Hz, 1H, 5-H), 6.91 (d, *J* = 9.0 Hz, 1H, 6-H), 4.71 (d, *J* = 9.7 Hz, 1H, 1'-H), 4.43 (d, *J* = 7.6 Hz, 1H, 1''-H), 3.97 – 3.86 (m, 2H), 3.84 (dd, *J* = 3.4, 1.1 Hz, 1H), 3.82 – 3.73 (m, 2H), 3.73 – 3.67 (m, 2H), 3.65 – 3.54 (m, 4H), 3.51 (dd, *J* = 9.7, 3.3 Hz, 1H). ^{13}C NMR (101 MHz, CD_3OD) δ 163.80 (C1), 141.61 (C4), 128.24 (C2), 126.07 (C5), 125.87 (C3), 116.88 (C6), 105.14 (C1''), 81.05, 80.80, 78.15, 77.12, 76.43 (C1'), 76.02, 74.86, 72.59, 70.34, 62.53, 62.17. IR (ATR, cm^{-1}): $\tilde{\nu}$ = 3337 (br) (O-H), 2922 (m) (C-H), 1592 (s) (NO_2), 1338 (s) (NO_2), 1284 (s) (C-O). UV/Vis: λ_{max} = 318 nm. HR-MS (ESI): m/z: calcd for $C_{18}H_{24}NO_{13}$ ([M-H]$^-$): 462.1253, found: 462.1252.

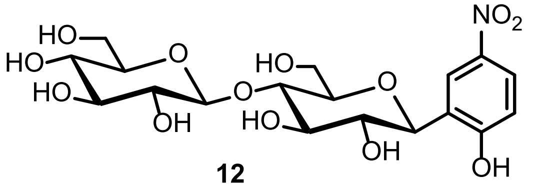

12

***p*-nitrophenol β-D-*C*-cellobioside (12).** Prepared using D-cellobiose according to general procedure B. Extracted with freshly distilled 2-MeTHF. Obtained as a beige amorphous solid (91 mg, 20%). According to general procedure C: stirred for 4 d after NaNMA addition. 53% NMR yield. R_f=0.69 (40 % EtOH, 1% AcOH in EtOAc). $[\alpha]_D^{20}$=+10 (*c*=0.10 in methanol). ^{1}H NMR (400 MHz, CD_3OD) δ 8.32 (d, *J* = 2.8 Hz, 1H, 3-H), 8.07 (dd, *J* = 9.0, 2.8 Hz, 1H, 5-H), 6.91 (d, *J* = 9.0 Hz, 1H, 6-H), 4.71 (d, *J* = 9.6 Hz, 1H, 1'-H), 4.48 (d, *J* = 7.8 Hz, 1H, 1''-H), 3.94 – 3.88 (m, 3H), 3.77 – 3.65 (m, 3H), 3.62 – 3.54 (m, 2H), 3.43 – 3.32 (m, 3H), 3.27 (dd, *J* = 9.1, 7.8 Hz, 1H). ^{13}C NMR (101 MHz, CD_3OD) δ 164.10 (C1), 141.46 (C4), 128.28 (C2), 126.10 (C5), 125.85 (C3), 116.95 (C6), 104.64 (C1''), 81.06, 80.86, 78.17, 78.15, 77.89, 76.41 (C1'), 76.14, 74.98, 71.42, 62.47, 62.11. IR (ATR, cm^{-1}): $\tilde{\nu}$ = 3304 (br) (O-H), 2876 (m) (C-H),

1590 (s) (NO_2), 1337 (s) (NO_2), 1290 (s) (C-O). UV/Vis: λ_{max} = 319 nm. HR-MS (ESI): m/z: calcd for $C_{18}H_{24}NO_{13}$ ([M-H]$^-$): 462.1253, found: 462.1250.

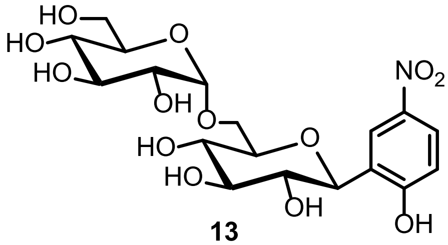

13

***p*-nitrophenol β-D-*C*-isomaltoside (13).** Prepared using D-isomaltose (0.15 mmol scale) according to general procedure B. Extracted with freshly distilled 2-MeTHF. Obtained as a beige amorphous solid (6.2 mg, 9%). According to general procedure C: stirred for 7 d after NaNMA addition. 69% NMR yield. R_f=0.85 (40 % EtOH, 1% AcOH in EtOAc). $[\alpha]_D^{20}$=+78 (*c*=0.10 in methanol). ^{1}H NMR (400 MHz, CD_3OD) δ 8.23 (d, *J* = 2.8 Hz, 1H, 3-H), 8.05 (dd, *J* = 9.0, 2.8 Hz, 1H, 5-H), 6.90 (d, *J* = 9.0 Hz, 1H, 6-H), 4.85 (d, *J* = 4.4 Hz, 1H, 1''-H), 4.65 (d, *J* = 8.6 Hz, 1H, 1'-H), 3.92 (dd, *J* = 11.2, 5.6 Hz, 1H), 3.82 (dd, *J* = 11.2, 2.1 Hz, 1H), 3.74 – 3.68 (m, 1H), 3.59 (ddd, *J* = 10.3, 7.8, 5.6 Hz, 3H), 3.55 – 3.47 (m, 4H), 3.36 (dd, *J* = 9.7, 3.8 Hz, 1H), 3.31-3.26 (m, 1H). ^{13}C NMR (101 MHz, CD_3OD) δ 164.02 (C1), 141.50 (C4), 128.18 (C2), 126.11 (C5), 125.76 (C3), 116.99 (C6), 100.16 (C1''), 81.00, 79.90, 76.74 (C1'), 75.99, 75.11, 73.71, 73.50, 71.96, 71.46, 68.38, 62.33. IR (ATR, cm^{-1}): ṽ = 3273 (br) (O-H), 2924 (m) (C-H), 1590 (s) (NO_2), 1337 (s) (NO_2), 1286 (s) (C-O). UV/Vis: λ_{max} = 318 nm. HR-MS (ESI): m/z: calcd for $C_{18}H_{24}NO_{13}$ ([M-H]$^-$): 462.1253, found: 462.1242.

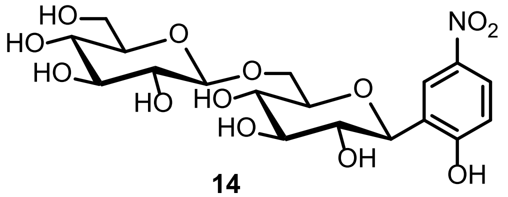

14

***p*-nitrophenol-β-D-*C*-gentiobioside (14).** Prepared using D-gentiobiose (0.15 mmol scale) according to general procedure B. Extracted with freshly distilled 2-MeTHF. Obtained as a beige amorphous solid (9.0 mg, 13%). According to general procedure C: stirred for 7 d after NaNMA addition. 73% NMR yield. R_f=0.44 (40 % EtOH, 1% AcOH in EtOAc). $[\alpha]_D^{20}$=+12 (*c*=0.10 in methanol). ^{1}H NMR (400 MHz, CD_3OD) δ 8.25 (d, *J* = 2.8 Hz, 1H, 3-H), 8.07 (dd, *J* = 9.0, 2.9 Hz, 1H, 5-H), 6.93 (d, *J* = 9.0 Hz, 1H, 6-H), 4.69 (d, *J* = 8.8 Hz, 1H, 1'-H), 4.34 (d, *J* = 7.8 Hz, 1H, 1''-H), 4.24 (dd, *J* = 11.2, 1.9 Hz, 1H), 3.85 (dd, *J* = 12.0, 2.0 Hz, 1H), 3.81 (dd, *J* = 11.2, 5.9 Hz, 1H), 3.66 (dd, *J* = 11.9, 5.0 Hz, 1H), 3.64 – 3.59 (m, 1H), 3.56 – 3.49 (m, 3H), 3.36 – 3.32 (m, 1H), 3.31 – 3.24 (m, 2H), 3.21 (dd, *J* = 9.0, 7.7 Hz, 1H). ^{13}C NMR (101 MHz, CD_3OD) δ 163.48 (C1), 141.74 (C4), 128.05 (C2), 126.09 (C5), 125.80 (C3), 116.91 (C6), 104.64 (C1''), 81.47, 79.62, 77.99, 77.94, 76.71 (C1'), 76.07, 75.08, 71.70, 71.49, 70.36, 62.67. IR (ATR, cm^{-1}): ṽ = 3273 (br) (O-H), 2883 (m) (C-H), 1588 (s) (NO_2), 1337 (s) (NO_2), 1287 (s) (C-O). UV/Vis: λ_{max} = 318 nm. HR-MS (ESI): m/z: calcd for $C_{18}H_{24}NO_{13}$ ([M-H]$^-$): 462.1253, found: 462.1245.

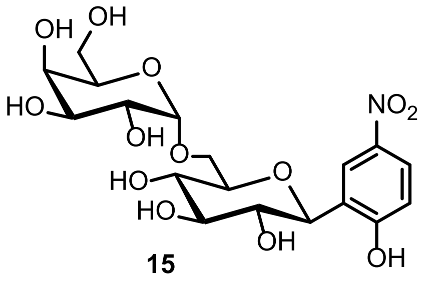

15

***p*-nitrophenol-β-D-*C*-melibioside (15).** Prepared using D-melibiose monohydrate according to general procedure B. Extracted with freshly distilled 2-MeTHF. Obtained as a beige amorphous solid (67 mg, 15%). According to general procedure C: stirred for 4 d after NaNMA addition. 48% NMR yield. R_f=0.41 (40 % EtOH, 1% AcOH in EtOAc). $[\alpha]_D^{20}$=+82 (*c*=0.10 in methanol). ^{1}H NMR (400 MHz, CD_3OD) δ 8.28 (d, *J* = 2.9 Hz, 1H, 3-H), 8.08 (dd, *J* = 9.0, 2.9 Hz 1H, 5-H), 6.93 (d, *J* = 9.0 Hz, 1H, 6-H), 4.92 (d, *J* = 3.4 Hz, 1H, 1''-H), 4.67 (d, *J* = 9.1 Hz, 1H, 1'-H), 3.96 (dd, *J* = 11.2, 5.5 Hz, 1H), 3.89 (td, *J* = 5.5, 1.8 Hz, 1H), 3.86 (dd, *J* = 3.0, 1.2 Hz, 1H), 3.82 (dd, *J* = 11.2, 2.3 Hz, 1H), 3.79 – 3.71

(m, 2H), 3.69 (dd, *J* = 5.9, 1.9 Hz, 2H), 3.67 – 3.61 (m, 1H), 3.58 – 3.50 (m, 3H). ^{13}C NMR (101 MHz, CD_3OD) δ 163.77 (C1), 141.63 (C4), 128.05 (C2), 126.13 (C5), 125.78 (C3), 116.98 (C6), 100.29 (C1''), 80.91, 79.88, 76.81 (C1'), 75.84, 72.26, 72.02, 71.56, 71.24, 70.38, 68.15, 62.89. IR (ATR, cm^{-1}): $\tilde{\nu}$ = 3297 (br) (O-H), 2887 (m) (C-H), 1588 (s) (NO_2), 1337 (s) (NO_2), 1287 (s) (C-O). UV/Vis: λ_{max} = 318 nm. HR-MS (ESI): m/z: calcd for $C_{18}H_{24}NO_{13}$ ([M-H]$^-$): 462.1253, found: 462.1249.

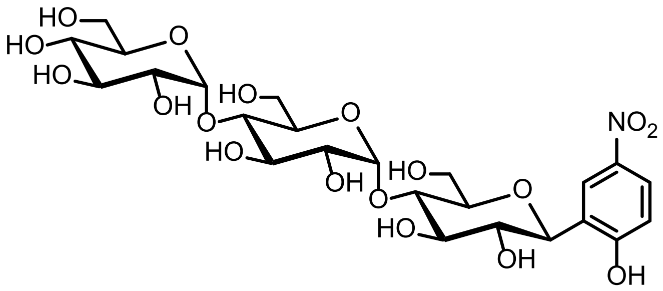

16

***p*-nitrophenol-β-D-*C*-maltotrioside (16).** Prepared using D-maltotriose monohydrate according to general procedure B. Extracted with 2-MeTHF. Obtained as a beige amorphous solid (47 mg, 8%). According to general procedure C: stirred for 4 d after NaNMA addition. 25% NMR yield. R_f=0.35 (40 % EtOH, 1% AcOH in EtOAc). $[\alpha]_D^{20}$=+96 (*c*=0.10 in methanol). ^{1}H NMR (400 MHz, CD_3OD) δ 8.32 (d, *J* = 2.8 Hz, 1H, 3-H), 8.07 (dd, *J* = 9.0, 2.9 Hz, 1H, 5-H), 6.92 (d, *J* = 9.0 Hz, 1H, 6-H), 5.24 (d, *J* = 3.8 Hz, 1H, 1''/1'''-H), 5.17 (d, *J* = 3.8 Hz, 1H, 1''/1'''-H), 4.71 (d, *J* = 9.7 Hz, 1H, 1'-H), 3.95 – 3.76 (m, 7H), 3.74 – 3.48 (m, 9H), 3.45 (dd, *J* = 9.7, 3.7 Hz, 1H), 3.30 – 3.25 (m, 1H). ^{13}C NMR (101 MHz, CD_3OD) δ 163.34 (C1), 141.83 (C4), 128.16 (C2), 126.04 (C5), 125.93 (C3), 116.75 (C6), 102.93 (C1''/C1'''), 102.78 (C1''/C1'''), 81.54, 81.39, 81.12, 79.59, 76.50 (C1'), 75.88, 75.11, 74.99, 74.79, 74.28, 73.91, 73.37, 71.52, 62.74, 62.47, 62.16. IR (ATR, cm^{-1}): $\tilde{\nu}$ = 3281 (br) (O-H), 2921 (m) (C-H), 1590 (s) (NO_2), 1337 (s) (NO_2), 1290 (s) (C-O). UV/Vis: λ_{max} = 320 nm. HR-MS (ESI): m/z: calcd for $C_{24}H_{34}NO_{18}$ ([M-H]$^-$): 624.1781, found: 624.1780.

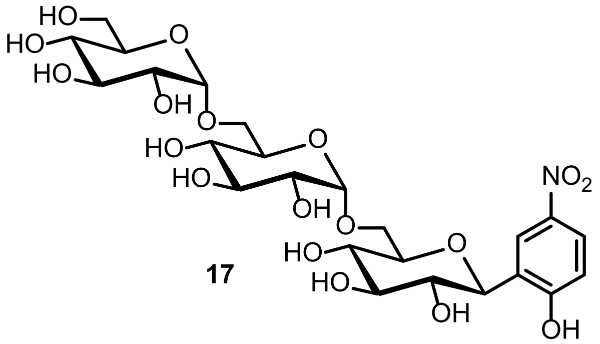

17

***p*-nitrophenol-β-D-*C*-isomaltotrioside (17).** Prepared using D-isomaltotriose (0.10 mmol scale) according to general procedure C. stirred for 4 d after NaNMA addition. 37% NMR yield. R_f=0.12 (40 % EtOH, 1% AcOH in EtOAc). $[\alpha]_D^{20}$=+104 (*c*=0.10 in methanol). ^{1}H NMR (400 MHz, CD_3OD) δ 8.24 (d, *J* = 2.9 Hz, 1H, 3-H), 8.05 (dd, *J* = 9.0, 2.9 Hz, 1H, 5-H), 6.84 (d, *J* = 9.1 Hz, 1H, 6-H), 4.87 (1H, 1''/1'''-H), 4.81 (d, *J* = 3.7 Hz, 1H, 1''/1'''-H), 4.70 – 4.66 (m, 1H, 1'-H), 3.96 (dd, *J* = 11.2, 5.8 Hz, 1H), 3.89 – 3.75 (m, 4H), 3.70 – 3.59 (m, 6H), 3.53 (m, 3H), 3.39 (m, 4H). ^{13}C NMR (101 MHz, CD_3OD) δ 170.29 (C1), 140.36 (C4), 128.41 (C2), 126.34 (C5), 125.71 (C3), 117.55 (C6), 100.01 (C1''/C1'''), 99.67 (C1''/C1'''), 80.90, 80.14, 76.75 (C1'), 76.22, 75.22, 75.20, 73.75, 73.62, 73.49, 71.95, 71.90, 71.60, 71.56, 68.50, 67.06, 62.47. IR (ATR, cm^{-1}): $\tilde{\nu}$ = 3271 (br) (O-H), 2926 (m) (C-H), 1589 (s) (NO_2), 1337 (s) (NO_2), 1286 (s) (C-O). UV/Vis: λ_{max} = 318 nm. HR-MS (ESI): m/z: calcd for $C_{24}H_{34}NO_{18}$ ([M-H]$^-$): 624.1781, found: 624.1780.

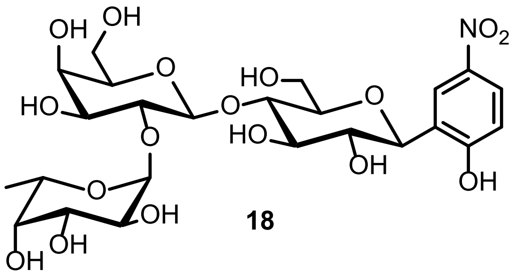

18

***p*-nitrophenol-β-*C*-2’-fucosyllactoside (18).** Prepared using 2’-fucosyllactoside according to general procedure B. Extracted with 2-MeTHF. Obtained as a beige amorphous solid (46 mg, 8%). According to general procedure C: stirred for 4 d after NaNMA addition. 47% NMR yield. R_f=0.40 (40 % EtOH, 1% AcOH in EtOAc). $[\alpha]_D^{20}$=−48 (*c*=0.10 in methanol). ^{1}H NMR (400 MHz, CD_3OD) δ 8.33 (d, *J* = 2.8 Hz, 1H, 3-H), 8.07 (dd, *J* = 9.0, 2.8 Hz, 1H, 5-H), 6.93 (d, *J* = 9.0 Hz, 1H, 6-H), 5.28 (d, *J* = 2.8 Hz, 1H, 1’”-H), 4.70 (d, *J* = 9.3 Hz, 1H, 1”-H), 4.55 (d, *J* = 6.9 Hz, 1H, 1’-H), 4.23 (q, *J* = 6.6 Hz, 1H), 3.89 (dd, *J* = 6.8, 3.2 Hz, 2H), 3.87 – 3.57 (m, 12H), 3.56 – 3.47 (m, 1H), 1.24 (d, *J* = 6.6 Hz, 3H). ^{13}C NMR (101 MHz, CD_3OD) δ 163.61 (C1), 141.67 (C4), 128.14 (C2), 126.09 (C5), 125.92 (C3), 116.81 (C6), 102.56 (C1’”), 101.69 (C1’”), 81.44, 78.68, 78.15, 77.99, 76.98, 76.46 (C1’), 76.02, 75.38, 73.58, 71.65, 70.70, 70.63, 68.25, 62.55, 61.86, 16.73 (C6’”). IR (ATR, cm^{-1}): $\tilde{\nu}$ = 3306 (br) (O-H), 2931 (m) (C-H), 1591 (s) (NO_2), 1336 (s) (NO_2), 1289 (s) (C-O). UV/Vis: λ_{max} = 318 nm. HR-MS (ESI): m/z: calcd for $C_{24}H_{34}NO_{17}$ ($[M-H]^-$): 608.1832, found: 608.1831.

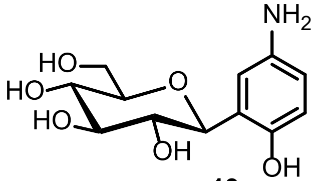

19

***p*-aminophenol-β-D-*C*-glucoside (19). 2** (4.0 g, 13.3 mmol) and Na_2CO_3 (7.0 g, 66.4 mmol, 5 eq.) were dissolved in 85 mL water and the mixture was cooled in an ice bath. To the cooled mixture, sodium dithionite ($Na_2S_2O_4$, 15 g, 86 mmol, 6.5 eq.) was added in portions, after which the mixture was stirred for 2 h, during which the characteristic yellow color faded. EtOH was added under vigorous stirring, in order to precipitate the salts as much as possible, the resulting slurry was filtered over a pad of Celite, and the filtrate was concentrated to dryness. The residue was resuspended in MeOH, solids were filtered off and the filtrate was concentrated to yield **19** as a beige amorphous solid (3.42 g, 95%). Because the compound gave inconsistent NMR data, it was converted to its hydrochloride salt (see below).

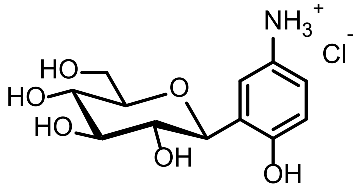

19a

***p*-aminophenol-β-D-*C*-glucoside hydrochloride (19a). 19** (100 mg, 0.37 mmol) was dissolved in a minimum amount of a 2 M ethanolic HCl solution, and concentrated to dryness to give the respective hydrochloride salt **19a** (114 mg, 100%) as a beige amorphous solid. ^{1}H NMR (400 MHz, CD_3OD) δ 7.40 (d, *J* = 2.8 Hz, 1H, 3-H), 7.17 (dd, *J* = 8.6, 2.8 Hz, 1H, 5-H), 6.93 (d, *J* = 8.6 Hz, 1H, 6-H), 4.65 (d, *J* = 9.4 Hz, 1H, 1’-H), 3.88 (dd, *J* = 11.9, 1.5 Hz, 1H, 6’-H), 3.73 (ddd, *J* = 12.0, 3.7, 1.6 Hz, 1H, 6’-H), 3.58 – 3.51 (m, 1H, 5’-H), 3.50 – 3.42 (m, 3H, 2’-H, 3’-H, 4’-H). ^{13}C NMR (101 MHz, CD_3OD) δ 157.19 (C1), 129.27 (C4), 124.25 (C5), 123.97 (C3), 123.20 (C2), 118.01 (C6), 82.33 (C5’), 79.82 (C3’), 76.97 (C1’), 76.39 (C2’), 71.73 (C4’), 62.88 (C6’). HR-MS (ESI): m/z: calcd for $C_{12}H_{18}NO_6$ ($[M+H]^+$): 272.1129, found: 272.1120.

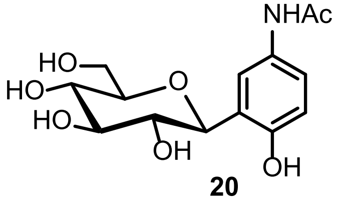

20

paracetamol-β-D-*C*-glucoside (20). 19 (500 mg, 1.84 mmol) was dissolved in 2 mL water, Ac_2O (0.21 mL, 2.21 mmol, 1.2 eq.) was added and the mixture was heated to 70 °C for 30 min. The mixture was concentrated to dryness, yielding the title compound in quantitative yield. Residual acetic acid was removed with column chromatography (0-40% EtOH in EtOAc), yielding **20** as an off-white amorphous solid (356 mg, 62%). R_f = 0.17 (10 % EtOH in EtOAc). ^{1}H NMR (400 MHz, CD_3OD) δ 7.43 (d, *J* = 2.7 Hz, 1H, 3-H), 7.29 (dd, *J* = 8.7, 2.6 Hz, 1H, 5-H), 6.77 (d, *J* = 8.7 Hz, 1H, 6-H), 4.59 (d, *J* = 9.3 Hz, 1H, 1'-H), 3.87 (dd, *J* = 12.0, 1.5 Hz, 1H, 6'-H), 3.75 – 3.67 (m, 1H, 6'-H), 3.57 – 3.47 (m, 2H, 2'-H, 3'-H), 3.46 – 3.37 (m, 2H, 4'-H, 5'-H), 2.08 (s, 3H, CH_3). ^{13}C NMR (101 MHz, CD_3OD) δ 171.50 (C=O), 153.56 (C1), 131.80 (C4), 127.18 (C2), 123.00 (C5), 122.50 (C3), 116.93 (C6), 82.31 (C5'), 79.86 (C3'), 77.81 (C1'), 75.98 (C2'), 71.83 (C4'), 63.03 (C6'), 23.47 (CH_3). HR-MS (APCI): m/z: calcd for $C_{14}H_{20}NO_7$ ($[M+H]^+$): 314.1234, found: 314.1231.

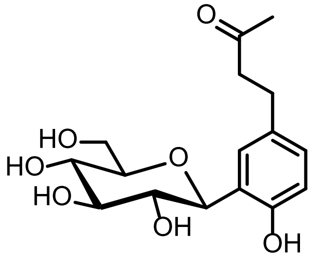

21

4-(*p*-hydroxyphenyl)-2-butanone β-D-*C*-glucoside (21). A solution of **19** (600 mg, 2.21 mmol) and conc. HCl (1 mL) in 5 mL water was cooled to 0 °C using an ice bath. $NaNO_2$ (160 mg, 2.32 mmol, 1.05 eq.) in 1 mL water was added dropwise to the first solution, and the mixture was stirred at 0 °C for 45 min (mixture A). Acetone (5 mL) was degassed using Schlenk vacuum/nitrogen cycling (3x), after which 10-15% aq. $TiCl_3$ solution (5 mL) and methyl vinyl ketone (0.54 mL, 6.64 mmol, 3 eq.) were added under N_2 atmosphere (mixture B). Mixture A was added dropwise to mixture B at 0 °C, and the mixture was allowed to reach room temperature over 2 h. It was then extracted with EtOAc, dried over anhydrous Na_2SO_4, filtered and concentrated. The desired product was purified using column chromatography (20% EtOH in EtOAc) to give the title compound as an off-white amorphous solid (173 mg, 24%). R_f=0.44 (10 % EtOH in EtOAc). ^{1}H NMR (400 MHz, CD_3OD) δ 7.18 (d, *J* = 2.3 Hz, 1H, 3-H), 6.97 (dd, *J* = 8.2, 2.3 Hz, 1H, 5-H), 6.72 (d, *J* = 8.2 Hz, 1H, 6-H), 4.55 (d, *J* = 9.2 Hz, 1H, 1'-H), 3.87 (dd, *J* = 12.0, 2.0 Hz, 1H, 6'-H), 3.72 (dd, *J* = 12.0, 5.1 Hz, 1H, 6'-H), 3.58 – 3.39 (m, 4H, 2'-5'-H), 2.77 (s, 4H, 2xCH_2), 2.12 (s, 3H, CH_3). ^{13}C NMR (101 MHz, CD_3OD) δ 211.42 (C=O), 154.83 (C1), 133.43 (C4), 129.70 (C5), 129.31 (C3), 126.71 (C2), 116.92 (C6), 82.32 (C5'), 80.01 (C3'), 78.34 (C1'), 75.82 (C2'), 71.75 (C4'), 62.94 (C6'), 46.15 (CH_2), 30.13 ($\underline{C}H_2CO$), 29.99 (CH_3). HR-MS (APCI): m/z: calcd for $C_{16}H_{23}O_7$ ($[M+H]^+$): 327.1438, found: 327.1436.

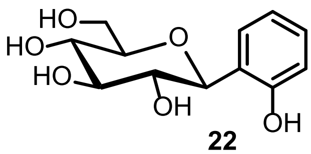

22

phenol-β-D-*C*-glucoside (22). A mixture of **19** (1.0 g, 3.69 mmol) in 50% aq. hypophosphorous acid (H_3PO_2, 8 mL, 73.7 mmol, 20 eq.) was cooled to 0 °C using an ice bath. $NaNO_2$ (1.27 g, 18.4 mmol, 5 eq.) dissolved in a minimum amount of water was added dropwise to the cooled solution, after which the mixture was allowed to slowly reach room temperature over 24 h. The reaction was then carefully neutralized using solid $NaHCO_3$, and then saturated with $MgSO_4$. The resulting mixture was extracted with acetonitrile (5x). The combined organic layers were thoroughly dried using anhydrous $MgSO_4$, filtered and concentrated to yield the desired product along with a small amount of phosphinate salt. The remaining phosphinate salt was removed by dissolving the crude product in water of pH 1, washing it with Et_2O (3x), and concentrating again to yield the product **22** free of phosphinate salts (575 mg, 61%). The last step introduced a new impurity, hence the material was purified for analysis using column

chromatography (0-30% EtOH in EtOAc), yielding the pure title compound as an off-white amorphous solid (257 mg, 27%). R_f = 0.54 (10 % EtOH in EtOAc). ^{1}H NMR (400 MHz, CD_3OD) δ 7.34 (dd, *J* = 7.7, 1.7 Hz, 1H, 3-H), 7.12 (ddd, *J* = 8.1, 7.3, 1.7 Hz, 1H, 4-H), 6.85 (td, *J* = 7.5, 1.2 Hz, 1H, 5-H), 6.81 (dd, *J* = 8.1, 1.2 Hz, 1H, 6-H), 4.59 (d, *J* = 9.3 Hz, 1H, 1'-H), 3.90 – 3.84 (m, 1H, 6'-H), 3.74 – 3.68 (m, 1H, 6'-H), 3.56 (t, *J* = 9.0 Hz, 1H, 2'-H), 3.53 – 3.48 (m, 1H, 3'-H), 3.47 – 3.41 (m, 2H, 4'-H, 5'-H). ^{13}C NMR (101 MHz, CD_3OD) δ 156.60 (C1), 129.89 (C4), 129.56 (C3), 126.70 (C2), 120.70 (C5), 116.83 (C6), 82.23 (C'5), 79.92 (C3'), 78.21 (C1'), 75.67 (C2'), 71.70 (C4'), 62.90 (C6'). HR-MS (ESI): m/z: calcd for $C_{12}H_{15}O_6$ ($[M-H]^-$): 255.0874, found: 255.0869.

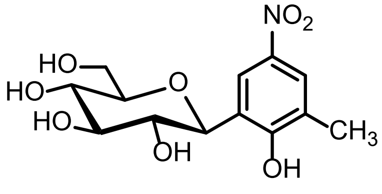

23

***o*-methyl-*p*-nitrophenol-β-D-*C*-glucoside (23).** Prepared using heptanedione (500 mg, 3.90 mmol) and D-glucose (738 mg, 4.10 mmol, 1.05 eq.) according to general procedure B to give the desired product with minor impurities (1.2 g, 98%) as an orange amorphous solid. An analytical sample was prepared by purification of 460 mg crude product using column chromatography (EtOH/EtOAc), yielding 239 mg of a pale orange amorphous solid. R_f=0.64 (10% EtOH in EtOAc). ^{1}H NMR (400 MHz, CD_3OD) δ 8.15 (d, *J* = 2.8 Hz, 1H, 3-H), 7.99 (dd, *J* = 2.8, 0.9 Hz, 1H, 5-H), 4.58 (d, *J* = 9.6 Hz, 1H, 1'-H), 3.91 (dd, *J* = 12.0, 1.6 Hz, 1H, 6'-H), 3.79 (dt, *J* = 12.2, 2.6 Hz, 1H, 6'-H), 3.59 – 3.40 (m, 4H, 2'-5'-H), 2.29 (s, 3H, CH_3). ^{13}C NMR (101 MHz, CD_3OD) δ 160.65 (C1), 141.75 (C4), 128.19 (C2), 127.45 (C6), 126.34 (C5), 123.19 (C3), 82.45 (C5'), 79.67 (C3'), 78.74 (C1'), 76.29 (C2'), 71.27 (C4'), 62.56 (C6'), 16.43 (CH_3). HR-MS (ESI): m/z: calcd for $C_{13}H_{16}NO_8$ ($[M-H]^-$): 314.0881, found: 314.0883.

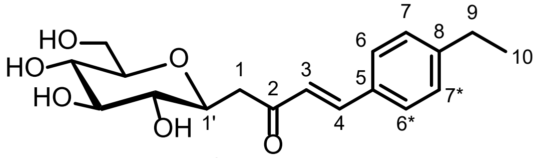

25

4-ethylbenzalacetone-β-D-*C*-glucoside (25). A mixture of D-glucose (9.31 g, 51.7 mmol, 1.15 eq.), acetylacetone (4.95 g, 49.4 mmol, 1.1 eq.), and $NaHCO_3$ (4.53 g, 53.9 mmol, 1.2 eq.) in water (32 mL) was heated at 90 °C for 16 h. After cooling to room temperature, the pH was adjusted to 5 using Amberlite H+ resin, which was subsequently filtered off. The filtrate was concentrated, yielding a brown syrup. The syrup was dissolved in MeOH (150 mL), 4-ethylbenzaldehyde (6.0 g, 44.9 mmol, 1.0 eq.) and $LiOH·H_2O$ (5.66 g, 135 mmol, 3 eq.) were added, and the mixture was stirred at room temperature. After 6 h, an additional equivalent of $LiOH·H_2O$ was added, and the mixture was stirred for 16 h. The reaction was diluted with water (150 mL) and washed with n-heptane (2x), followed by an extraction with freshly distilled 2-MeTHF (3x). The combined organic layers were dried over anhydrous Na_2SO_4, filtered and concentrated. Residual water was removed by co-evaporation with acetonitrile, yielding the product **25** as an orange amorphous solid (12.94 g, 86%) which was used in the next step without further purification. One CH_2 is missing in both ^{1}H and ^{13}C spectra due to H-D exchange[3]. R_f = 0.39 (10 % EtOH in EtOAc). ^{1}H NMR (400 MHz, CD_3OD) δ 7.65 (d, *J* = 16.2 Hz, 1H, 4-H), 7.57 (d, *J* = 8.2 Hz, 2H, 6/6*-H), 7.26 (d, *J* = 8.2 Hz, 2H, 7/7*-H), 6.87 (d, *J* = 16.2 Hz, 1H, 3-H), 3.78 (dd, *J* = 11.9, 2.4 Hz, 1H, 6'-H), 3.74 (d, *J* = 9.8 Hz, 1H, 1'-H), 3.63 (dd, *J* = 11.9, 5.1 Hz, 1H, 6'-H), 3.37 (t, *J* = 8.6 Hz, 1H, 3'-H), 3.36 – 3.27 (m, 1H, 4'-H), 3.24 (ddd, *J* = 9.3, 5.1, 2.3 Hz, 1H, 5'-H), 3.16 (dd, *J* = 9.6, 8.6 Hz, 1H, 2'-H), 2.67 (q, *J* = 7.6 Hz, 2H, 9-H_2), 1.24 (t, *J* = 7.6 Hz, 3H, 10-H_3). ^{13}C NMR (101 MHz, CD_3OD) δ 201.33 (C2), 148.85 (C8), 145.17 (C4), 133.46 (C5), 129.75 (C6/6*), 129.57 (C7/7*), 126.58 (C3),

81.61 (C5’), 79.69 (C3’), 77.47 (C1’), 75.11 (C2’), 71.67 (C4’), 62.74 (C6’), 29.78 (C9), 15.87 (C10). HR-MS (ESI): m/z: calcd for $C_{18}H_{24}O_6Na$ ($[M+Na]^+$): 359.1465, found: 359.1463.

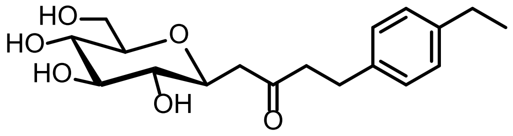

26

4-(4-ethylphenyl)2-butanone-β-D-*C*-glucoside (26). A solution of **25** (12.9 g, 38.3 mmol) in MeOH (150 mL) was added to a sat. aq. solution of NH_4Cl (150 mL). The mixture was heated to 45 °C, after which zinc granules (15 g, 230 mmol, 6 eq.) were added in three portions. After stirring for 16 h at 45 °C, the mixture was diluted with water and extracted with EtOAc (3x), followed by extraction with 2-MeTHF (3x). The organic layers were concentrated and the sticky residue was extracted with acetonitrile. Solids were filtered off, and the filtrate was concentrated to yield the title compound as a light brown amorphous solid (8.83 g, 68%) which was used in the next step without further purification. R_f = 0.44 (10 % EtOH in EtOAc). ^1H NMR (400 MHz, CD_3OD) δ 7.12 – 7.06 (m, 4H, 6/6*-H, 7/7*-H), 3.75 (dd, *J* = 11.9, 2.4 Hz, 1H, 6’-H), 3.65 (td, *J* = 9.4, 2.9 Hz, 1H, 1’-H), 3.60 (dd, *J* = 11.8, 5.1 Hz, 1H, 6’-H), 3.35 – 3.31 (m, 1H, 3’-H), 3.27 (t, *J* = 8.9 Hz, 1H, 4’-H), 3.19 (ddd, *J* = 9.3, 5.2, 2.3 Hz, 1H, 5’-H), 3.06 (t, *J* = 9.0 Hz, 1H, 2’-H), 2.88 – 2.81 (m, 5H, 3-H_2, 4-H_2, 1-H), 2.64 – 2.55 (m, 3H, 9-H_2, 1-H), 1.19 (t, *J* = 7.6 Hz, 3H, 10-H_3). ^{13}C NMR (101 MHz, CD_3OD) δ 211.16 (C2), 143.06 (C8), 139.76 (C5), 129.33 (C6/6*), 128.84 (C7/7*), 81.61 (C5’), 79.63 (C3’), 77.33 (C1’), 75.12 (C2’), 71.66 (C4’), 62.73 (C6’), 46.54 (C1), 45.95 (C3), 30.05 (C4), 29.45 (C9), 16.31 (C10). HR-MS (ESI): m/z: calcd for $C_{18}H_{26}O_6Na$ ($[M+Na]^+$): 361.1622, found: 361.1615.

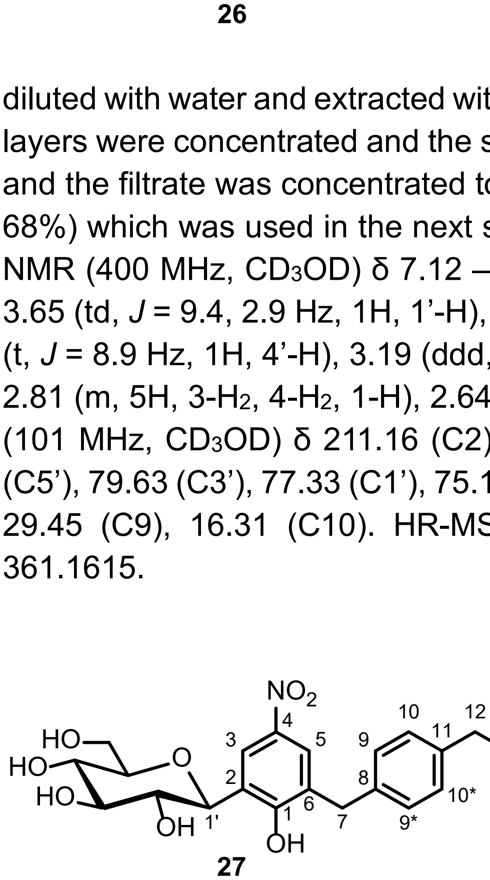

27

***o*-(4-ethylbenzyl)-*p*-nitrophenol-β-D-*C*-glucoside (27).** To a solution of **26** (6.8 g, 20.1 mmol) in ethanol (67 mL) were added a 2 M aq. NaOH solution (20 mL, 1.6 g, 40.2 mmol, 2 eq.) and **NMA** (3.47 g, 22.1 mmol, 1.1 eq.) and the mixture was stirred at room temperature for 3 days. The pH was adjusted to 5 using a 2 M aq. HCl solution, and the mixture was then extracted with Et_2O (3x). The combined organic layers were dried over anhydrous Na_2SO_4, filtered and concentrated to yield the title compound as an orange amorphous solid (4.24 g, 50%), which was used in the next step without further purification. R_f=0.69 (10 % EtOH in EtOAc). ^1H NMR (400 MHz, CD_3OD) δ 8.18 (d, *J* = 2.9 Hz, 1H, 3-H), 7.89 (d, *J* = 2.9 Hz, 1H, 5-H), 7.17 – 7.10 (m, 4H, 9/9*-H, 10/10*-H), 4.58 (d, *J* = 9.6 Hz, 1H, 1’-H), 3.98 (s, 2H, 7-H_2), 3.92 (dd, *J* = 12.1, 1.5 Hz, 1H, 6’-H), 3.84 – 3.77 (m, 1H, 6’-H), 3.57 – 3.50 (m, 1H, 3’-H), 3.50 – 3.46 (m, 2H, 4’/5’-H), 3.43 (dd, *J* = 9.6, 8.5 Hz, 1H, 2’-H), 2.60 (q, *J* = 7.6 Hz, 2H, 12-H_2), 1.20 (t, *J* = 7.6 Hz, 3H, 13-H_3). ^{13}C NMR (101 MHz, CD_3OD) δ 160.21 (C1), 143.43 (C11), 141.90 (C4), 138.20 (C8), 132.24 (C6), 129.99 (C9/9*), 128.97 (C10/10*), 127.90 (C2), 126.02 (C5), 123.40 (C3), 82.48 (C5’), 79.64 (C3’), 79.01 (C1’), 76.32 (C2’), 71.15 (C4’), 62.47 (C6’), 36.14 (C7), 29.46 (C12), 16.25 (C13). HR-MS (ESI): m/z: calcd for $C_{21}H_{24}NO_8$ ($[M-H]^-$): 418.1507, found: 418.1492.

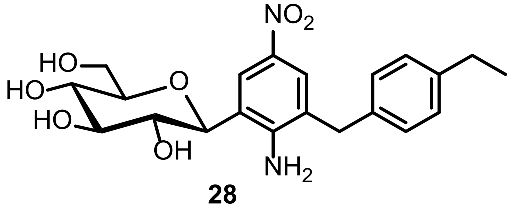

28

***o*-(4-ethylbenzyl)-*p*-nitroaniline-β-D-*C*-glucoside (28).** A mixture of **27** (2.0 g, 4.77 mmol), K_2CO_3 (1.32 g, 9.54 mmol, 2 eq.), chloroacetamide (892 mg, 9.54 mmol, 2 eq.), and KI (198 mg, 1.19 mmol, 0.25 eq.) in CH_3CN (48 mL) was stirred at reflux for 24 h. Additional K_2CO_3 (3.30 g, 23.8 mmol, 5 eq.) was added and stirring at reflux was continued for 16 h, after which the reaction cooled to room temperature. The mixture was diluted with water and brine, and extracted with EtOAc (5x). The combined organic layers were dried over anhydrous Na_2SO_4, filtered and concentrated to give the title compound as a yellow amorphous solid (1.4 g, 70%). R_f=0.58 (10 % EtOH in EtOAc). ^{1}H NMR (400 MHz, CD_3OD) δ 8.07 (d, *J* = 2.7 Hz, 1H, 3-H), 7.80 (d, *J* = 2.7 Hz, 1H, 5-H), 7.19 – 7.10 (m, 4H, 9/9*-H, 10/10*-H), 4.36 (d, *J* = 9.7 Hz, 1H, 1'-H), 3.92 – 3.86 (m, 3H, 6'-H, 7-H_2), 3.74 (dd, *J* = 12.0, 4.9 Hz, 1H, 6'-H), 3.70 – 3.64 (m, 1H, 2'-H), 3.54 – 3.41 (m, 3H, 3'/4'/5'-H), 2.62 (q, *J* = 7.7 Hz, 2H, 12-H_2), 1.22 (t, *J* = 7.6 Hz, 3H, 13-H_3). ^{13}C NMR (101 MHz, CD_3OD) δ 152.44 (C1), 143.87 (C11), 138.53 (C4), 136.68 (C8), 129.88 (C9/9*), 129.24 (C10/10*), 126.76 (C2), 126.51 (C5), 125.29 (C3), 123.40 (C6), 82.33 (C5'), 81.45 (C1'), 79.87 (C3'), 73.66 (C2'), 71.42 (C4'), 62.67 (C6'), 37.66 (C7), 29.47 (C12), 16.21 (C13). HR-MS (ESI): m/z: calcd for $C_{21}H_{27}N_2O_7$ ($[M+H]^+$): 419.1813, found: 419.1812.

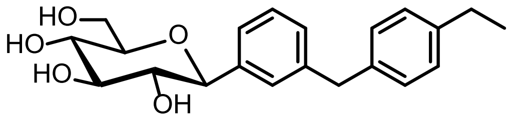

24

***o*-(4-ethylbenzyl)-phenyl-β-D-*C*-glucoside (25).** A solution of **28** (300 mg, 0.717 mmol) in 6 mL EtOH was prepared, to which a solution of Na_2CO_3 (380 mg, 3.59 mmol, 5 eq.) and $Na_2S_2O_4$ (811 mg, 4.66 mmol, 6.5 eq.) in 5 mL water was added. The mixture was stirred vigorously for 2 h at ambient temperature, after which it was extracted with EtOAc (3x). A small amount of AcOH was added to the extract to stabilize the diaminobenzene moiety. The mixture was dried over Na_2SO_4, filtered and concentrated to yield a white residue, which was directly dissolved in 3 mL fresh AcOH and a 50% aq. solution of H_3PO_2 (3.9 mL, 35.9 mmol, 50 eq.) was added. The mixture was cooled on ice, after which a solution of $NaNO_2$ (742 mg, 10.8 mmol, 15 eq.) in a minimal amount of water was added dropwise over 15 min. The resulting mixture was left to stir for 16 h, during which it was allowed to slowly reach ambient temperature. The acids were quenched using a sat. aq. $NaHCO_3$ solution, and the resulting mixture was extracted with EtOAc (3x), dried over Na_2SO_4, filtered and concentrated to yield the title compound as an off-white amorphous solid (185 mg, 72%). R_f=0.83 (10 % EtOH in EtOAc). ^{1}H NMR (400 MHz, CD_3OD) δ 7.29 (d, *J* = 1.9 Hz, 1H, 1-H), 7.27 – 7.21 (m, 2H, 3/5-H), 7.14 – 7.06 (m, 5H, 4-H, 9/9*-H, 10/10*-H), 4.09 (d, *J* = 9.4 Hz, 1H, 1'-H), 3.92 (s, 2H, 7-H_2), 3.87 (dd, *J* = 11.9, 1.7 Hz, 1H, 6'-H), 3.72 – 3.66 (m, 1H, 6'-H), 3.50 – 3.34 (m, 4H, 2'-5'-H), 2.58 (q, *J* = 7.5 Hz, 2H, 12-H_2), 1.19 (t, *J* = 7.6 Hz, 3H, 13-H_3). ^{13}C NMR (101 MHz, CD_3OD) δ 143.10 (C11), 142.75 (C6), 140.91 (C2), 139.86 (C8), 129.87 (C9/9*), 129.63 (C1), 129.54 (C4), 129.14 (C5), 128.84 (C10/10*), 126.68 (C3), 83.76 (C1'), 82.23 (C5'), 79.81 (C3'), 76.39 (C2'), 71.95 (C4'), 63.17 (C6'), 42.44 (C7), 29.44 (C12), 16.29 (C13). HR-MS (ESI): m/z: calcd for $C_{21}H_{30}NO_5$ ($[M+NH_4]^+$): 376.2119, found: 376.2121

NMR spectral data in DMSO-d6 are identical to previously reported.[4]

References

[1] D. Seebach, R. Imwinkelried, G. Stucky, *Helv. Chim. Acta* **1987**, *70*, 448–464.

[2] P. E. Fanta, *Org. Synth.* **1952**, *32*, 95.

[3] H. J. Reich, M. Jautelat, M. T. Messe, F. J. Weigert, J. D. Roberts, *J. Am. Chem. Soc.* **1969**, *91*, 7445–7454.

[4] P. P. Deshpande, J. Singh, A. Pullockaran, T. Kissick, B. A. Ellsworth, J. Z. Gougoutas, J. Dimarco, M. Fakes, M. Reyes, C. Lai, H. Lobinger, T. Denzel, P. Ermann, G. Crispino, M. Randazzo, Z. Gao, R. Randazzo, M. Lindrud, V. Rosso, F. Buono, W. W. Doubleday, S. Leung, P. Richberg, D. Hughes, W. N. Washburn, W. Meng, K. J. Volk, R. H. Mueller, *Org. Process Res. Dev.* **2012**, *16*, 577–585.

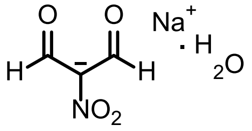

O
O
Na+
H
H
· H2O
NO2

NaNMA

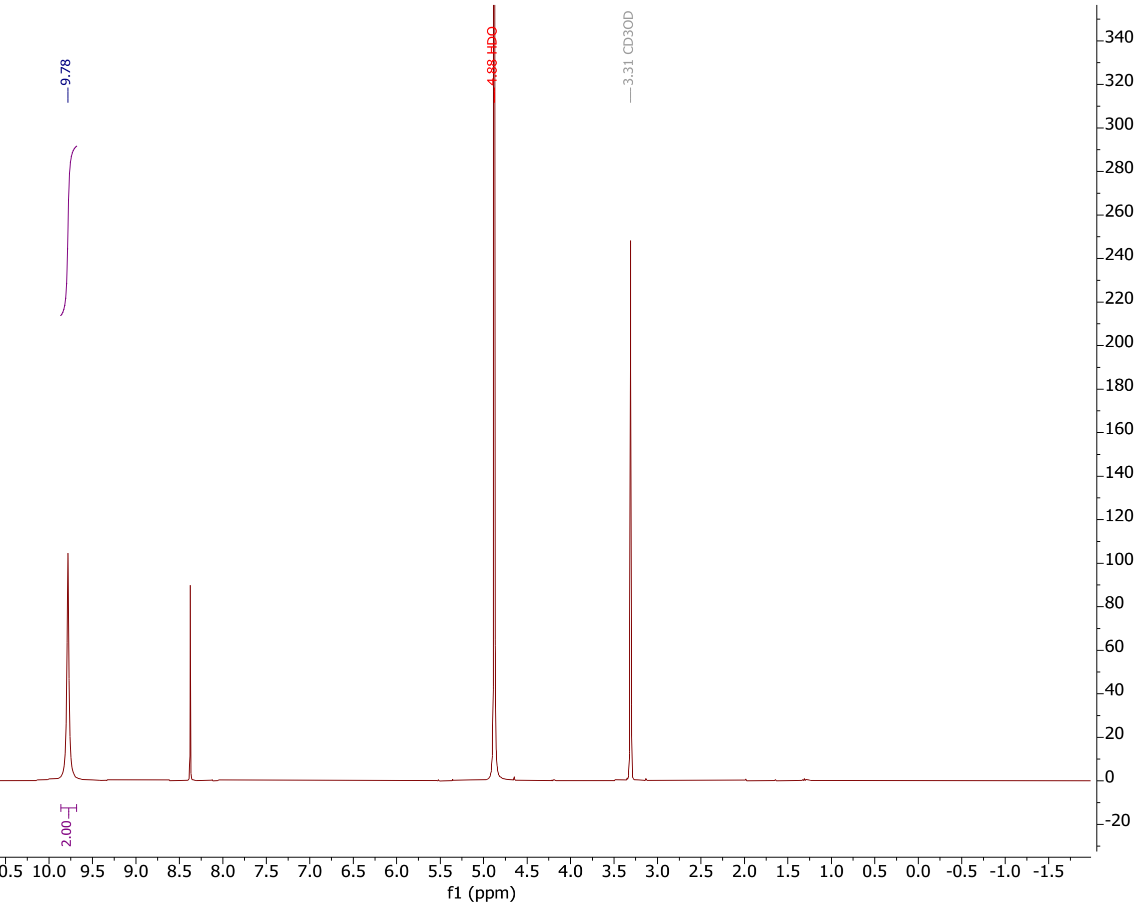

9.78
4.88 HDO
3.31 CD3OD
2.00
f1 (ppm)

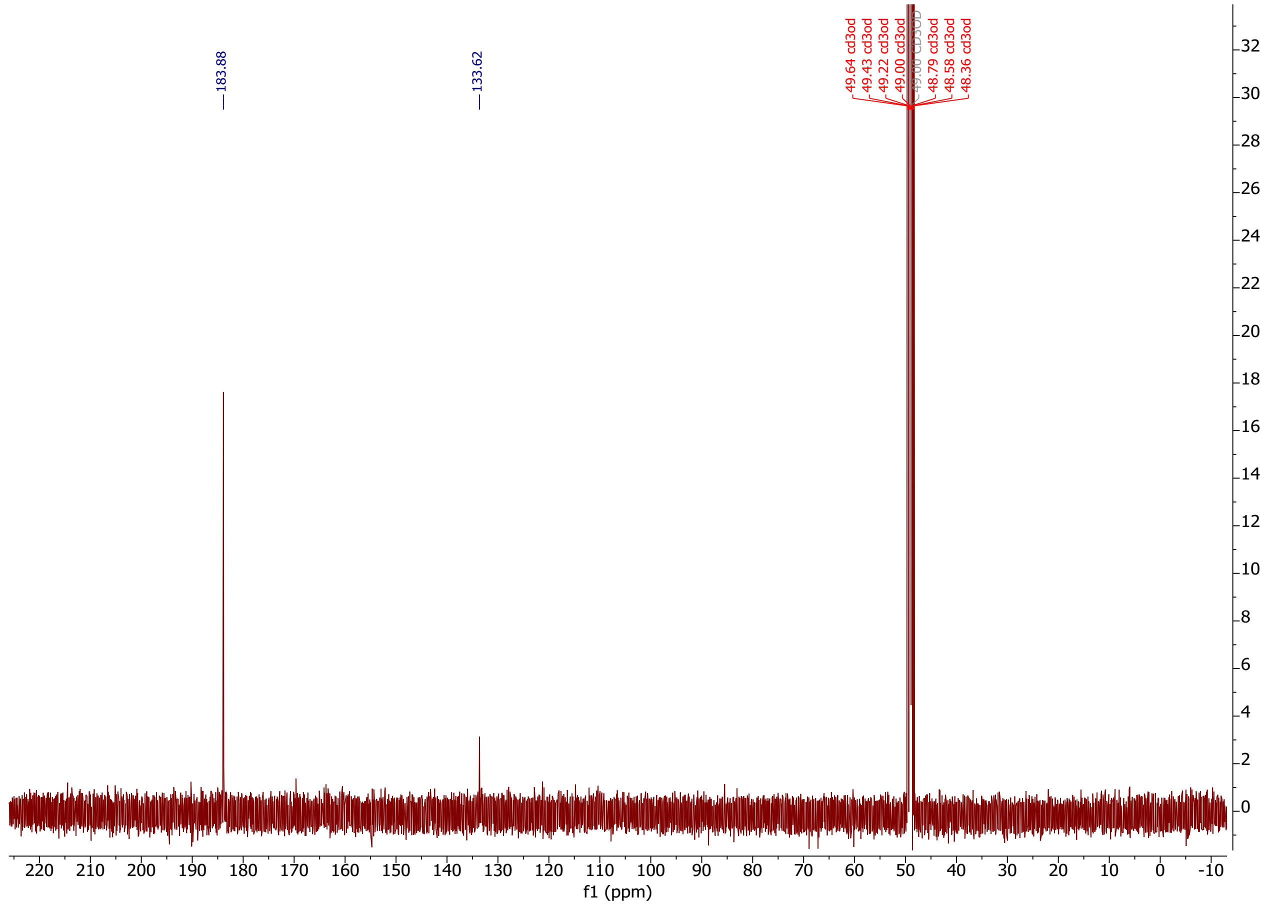

183.88
133.62
49.64 cd3od
49.43 cd3od
49.22 cd3od
49.00 cd3od
48.79 cd3od
48.58 cd3od
48.36 cd3od
49.00 CD3OD
f1 (ppm)

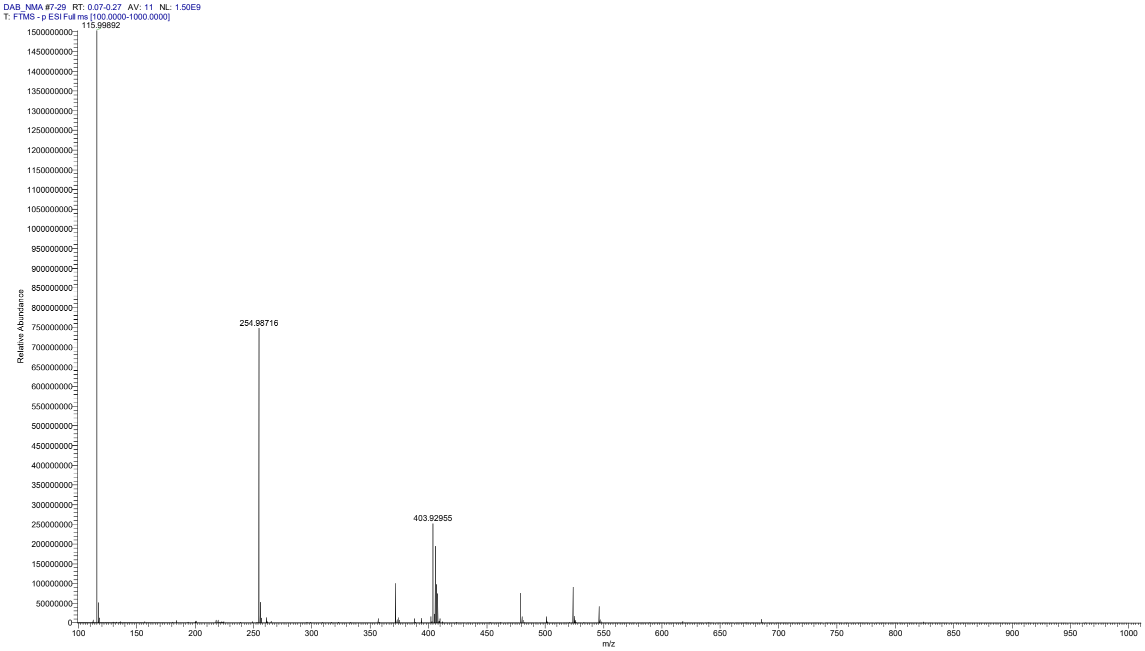

DAB_NMA #7-29 RT: 0.07-0.27 AV: 11 NL: 1.50E9
T: FTMS - p ESI Full ms [100.0000-1000.0000]
115.99892
254.98716
403.92955
Relative Abundance
m/z

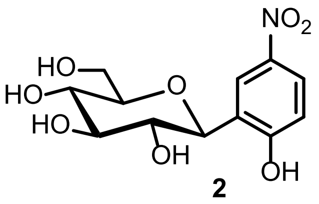
NO2
HO
HO
HO
O
OH
OH
2

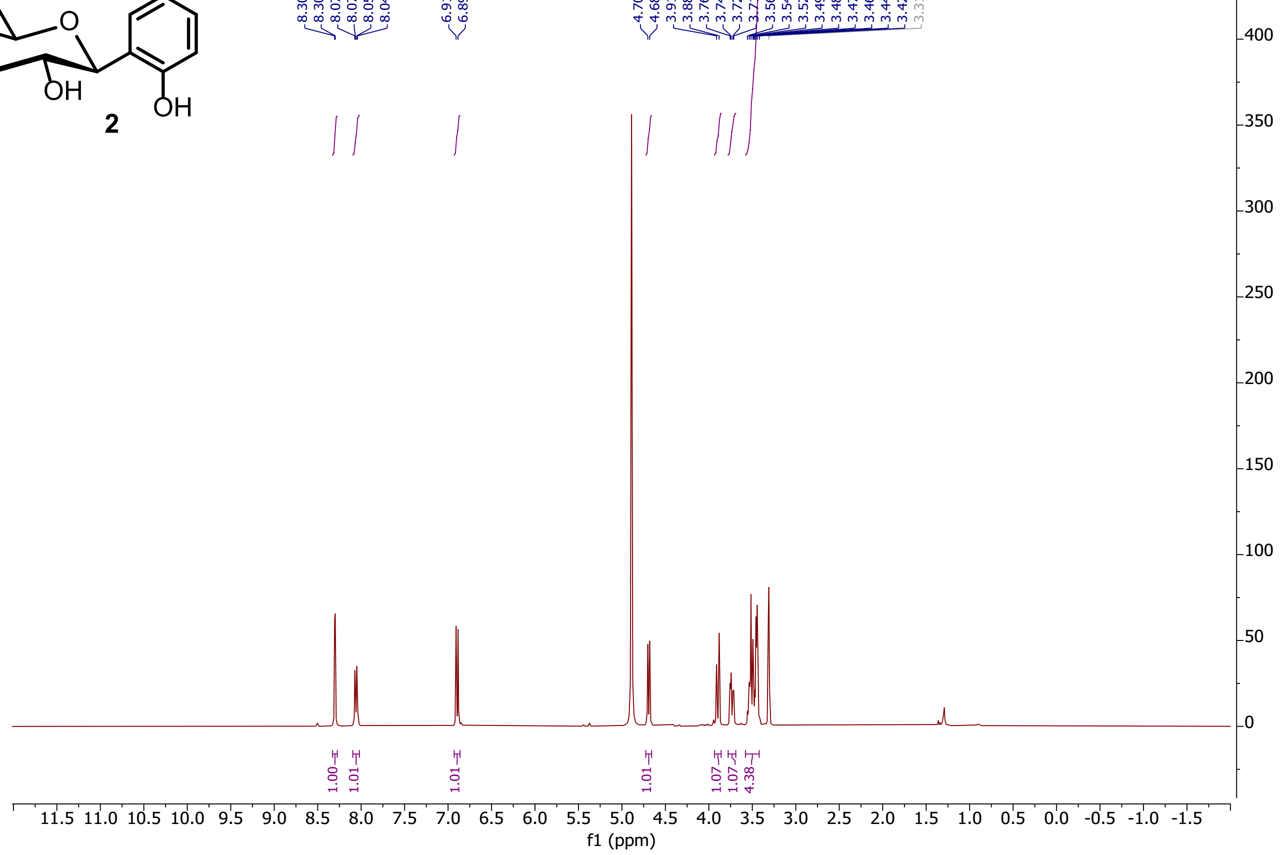
8.30
8.30
8.07
8.07
8.05
8.04
6.91
6.89
4.70
4.68
3.91
3.88
3.76
3.74
3.72
3.71
3.56
3.54
3.52
3.49
3.48
3.47
3.46
3.44
3.42
3.31 CD3OD
1.00
1.01
1.01
1.01
1.07
1.07
4.38
f1 (ppm)

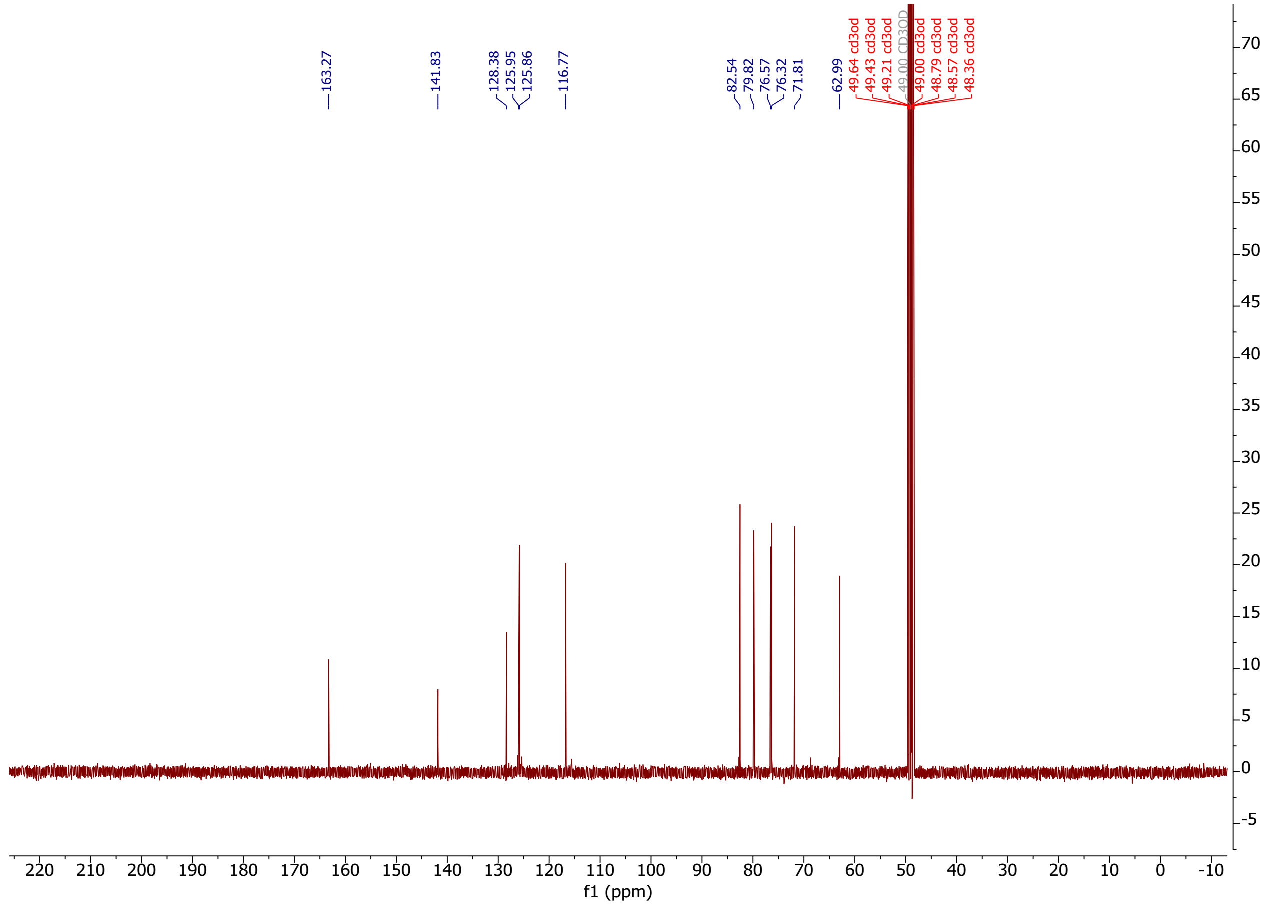
163.27
141.83
128.38
125.95
125.86
116.77
82.54
79.82
76.57
76.32
71.81
62.99
49.64 cd3od
49.43 cd3od
49.21 cd3od
49.00 CD3OD
49.00 cd3od
48.79 cd3od
48.57 cd3od
48.36 cd3od
f1 (ppm)

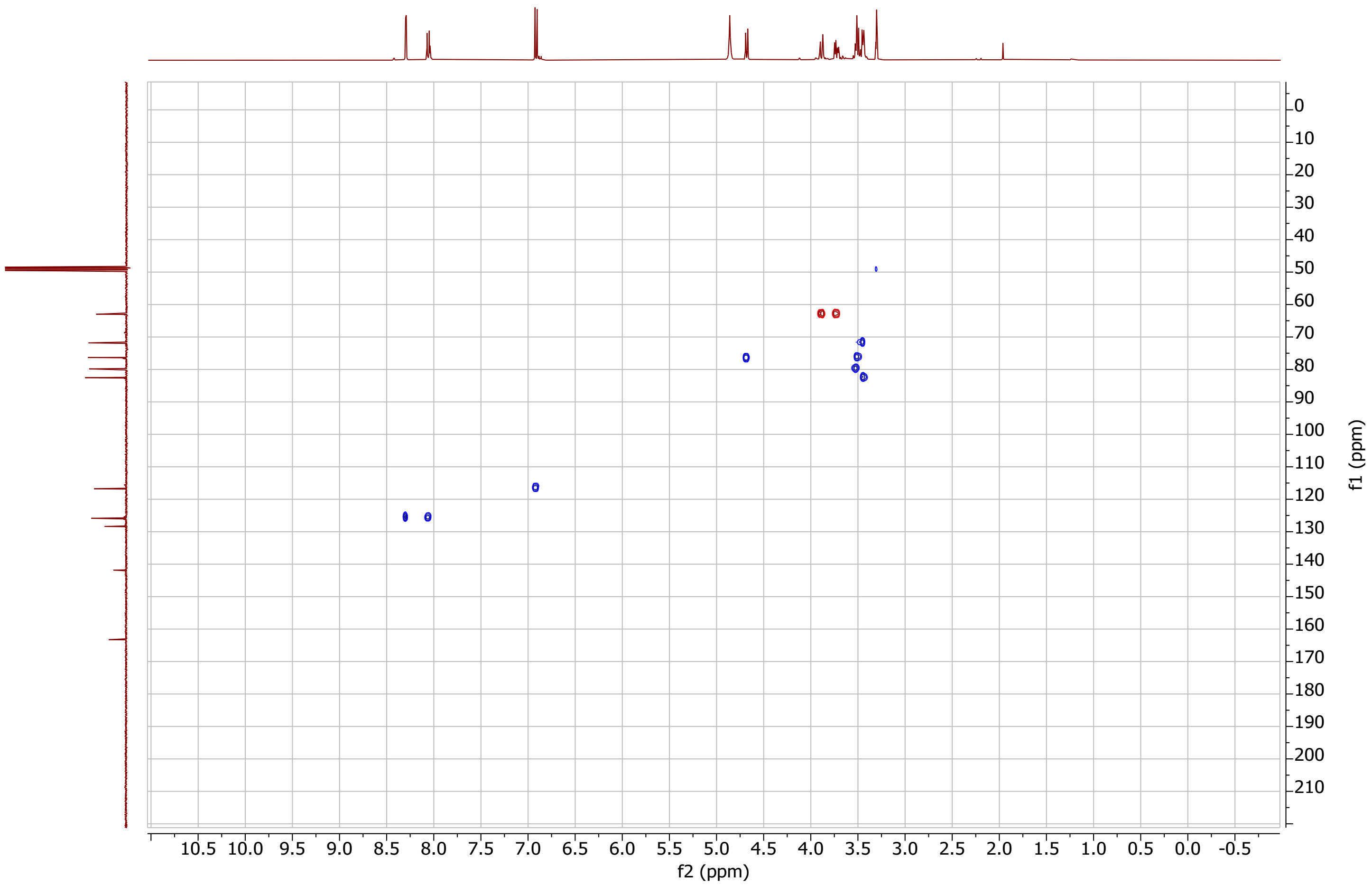
f2 (ppm)
f1 (ppm)

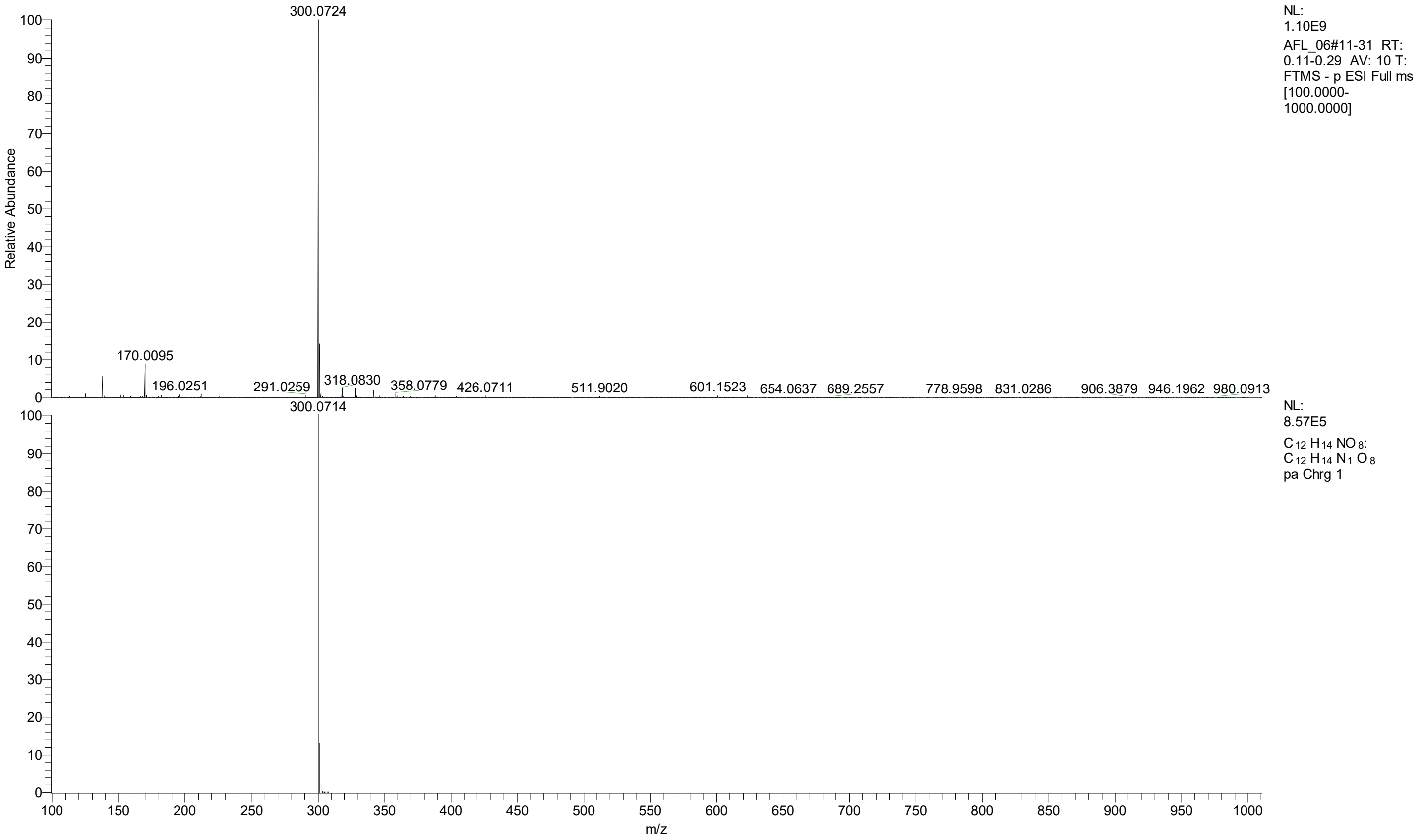
300.0724
170.0095
196.0251
291.0259
318.0830
358.0779
426.0711
511.9020
601.1523
654.0637
689.2557
778.9598
831.0286
906.3879
946.1962
980.0913
NL:
1.10E9
AFL_06#11-31 RT:
0.11-0.29 AV: 10 T:
FTMS - p ESI Full ms
[100.0000-
1000.0000]
300.0714
NL:
8.57E5
C 12 H 14 NO 8:
C 12 H 14 N 1 O 8
pa Chrg 1
Relative Abundance
m/z

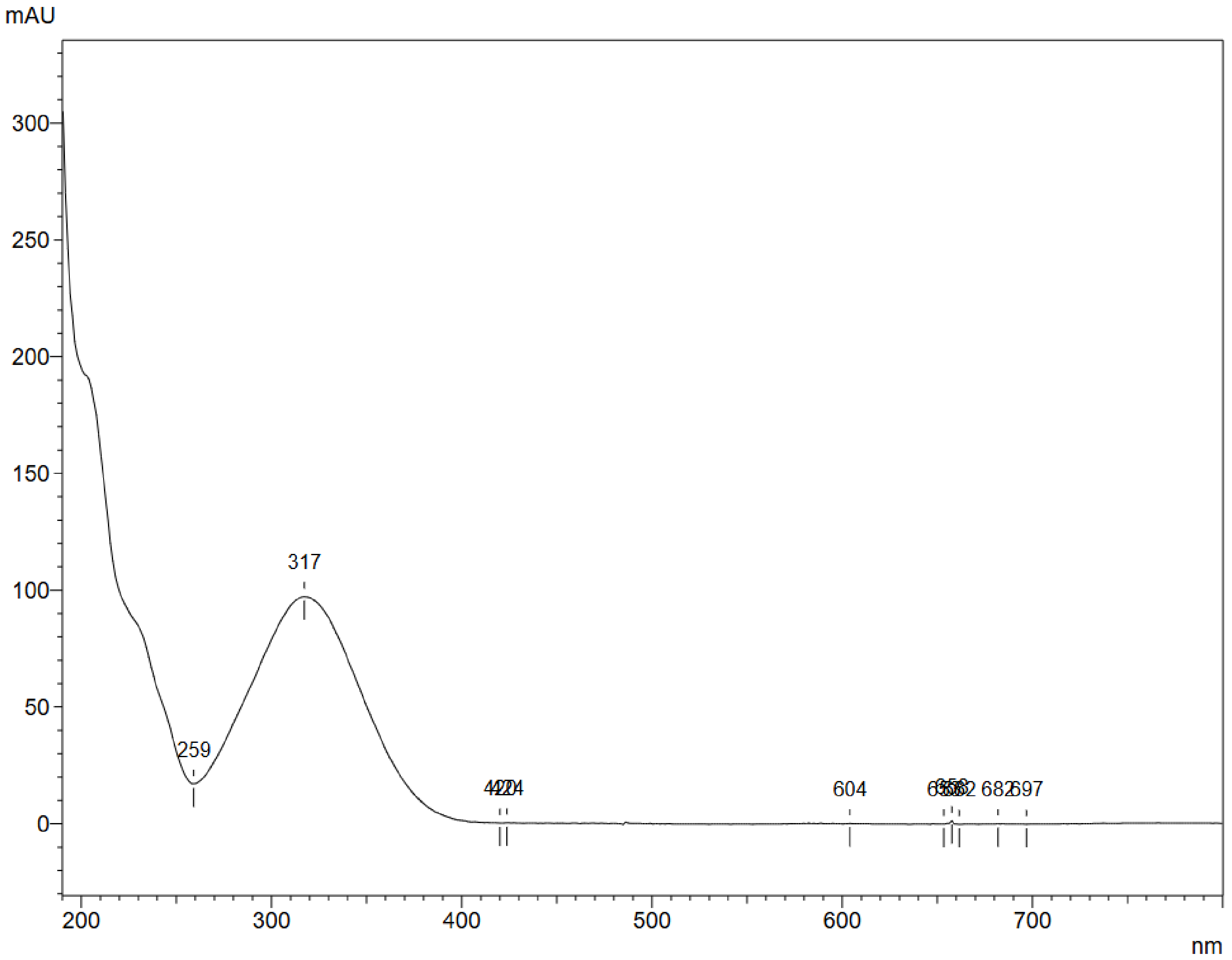
mAU
300
250
200
150
100
50
0
317
259
604
682
697
200
300
400
500
600
700
nm

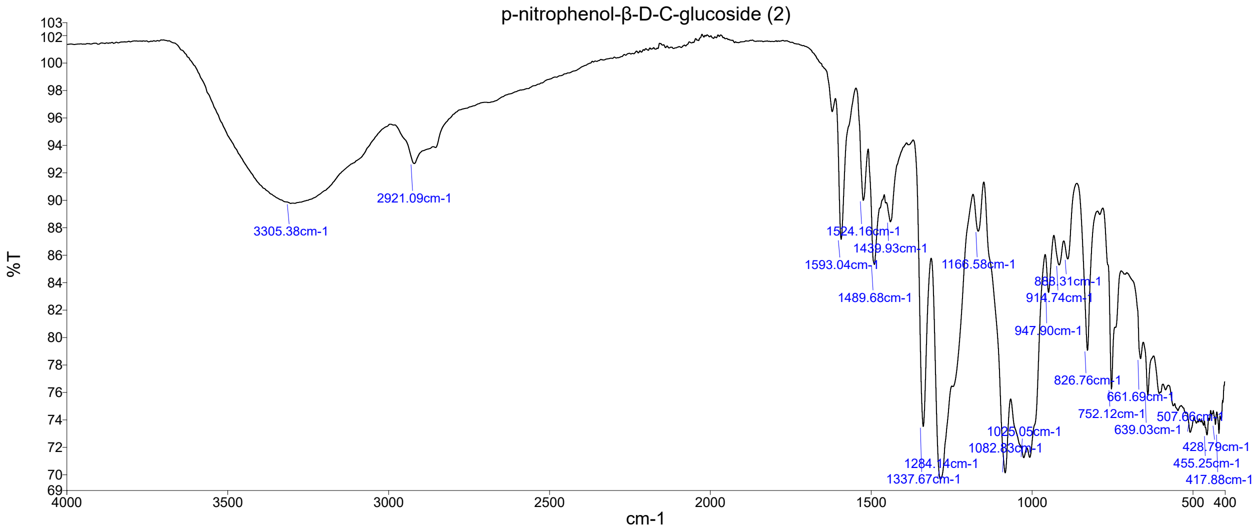
p-nitrophenol-β-D-C-glucoside (2)
%T
cm-1
3305.38cm-1
2921.09cm-1
1593.04cm-1
1524.16cm-1
1439.93cm-1
1489.68cm-1
1166.58cm-1
1284.14cm-1
1337.67cm-1
1025.05cm-1
1082.83cm-1
914.74cm-1
947.90cm-1
826.76cm-1
661.69cm-1
752.12cm-1
639.03cm-1
428.79cm-1
455.25cm-1
417.88cm-1

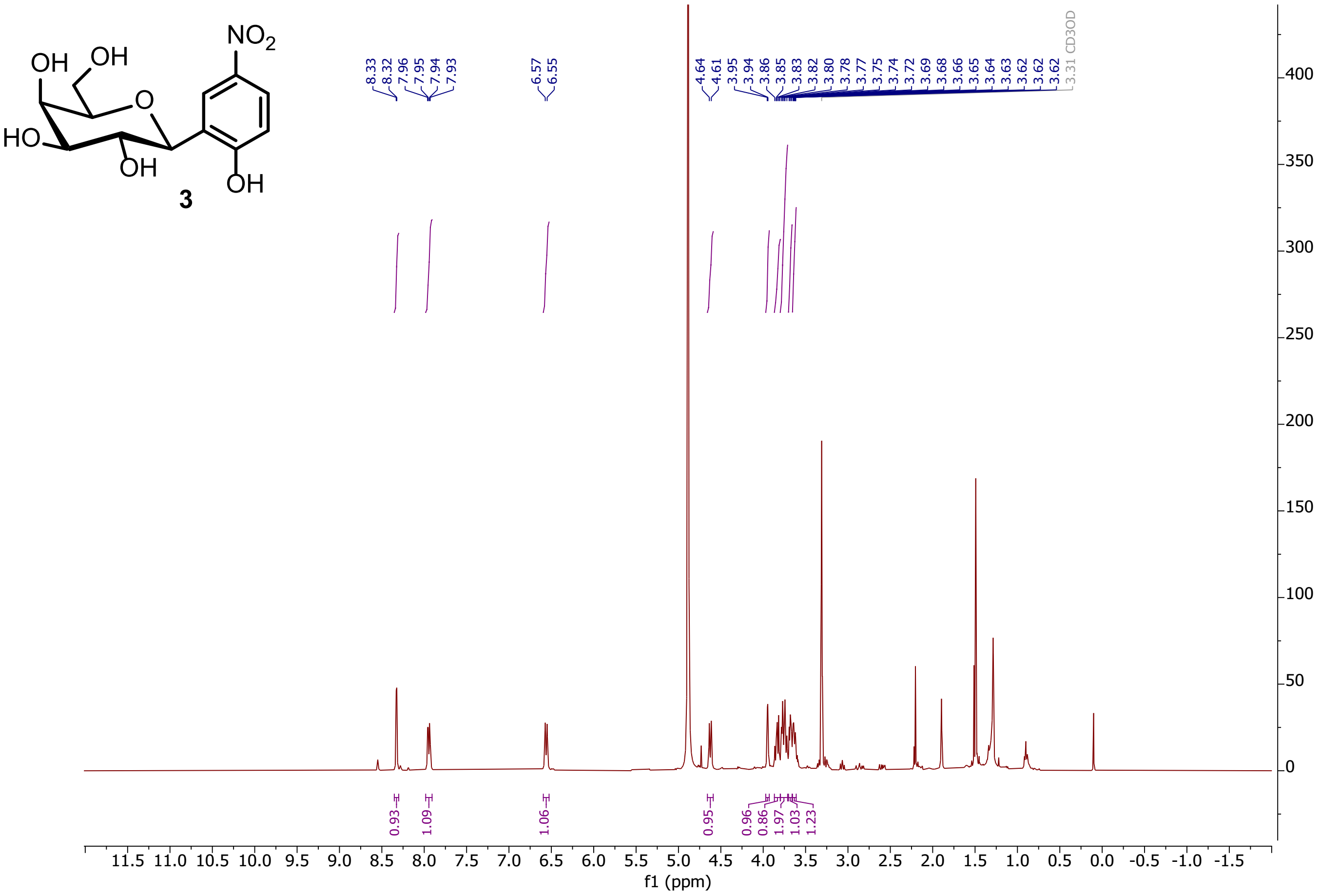
NO2
OH
OH
O
HO
OH
OH
8.33
8.32
7.96
7.95
7.94
7.93
6.57
6.55
4.64
4.61
3.95
3.94
3.86
3.85
3.83
3.82
3.80
3.78
3.77
3.75
3.74
3.72
3.69
3.68
3.66
3.65
3.64
3.63
3.62
3.62
3.62
3.31 CD3OD
0.93
1.09
1.06
0.95
0.96
0.86
1.97
1.03
1.23
f1 (ppm)

3

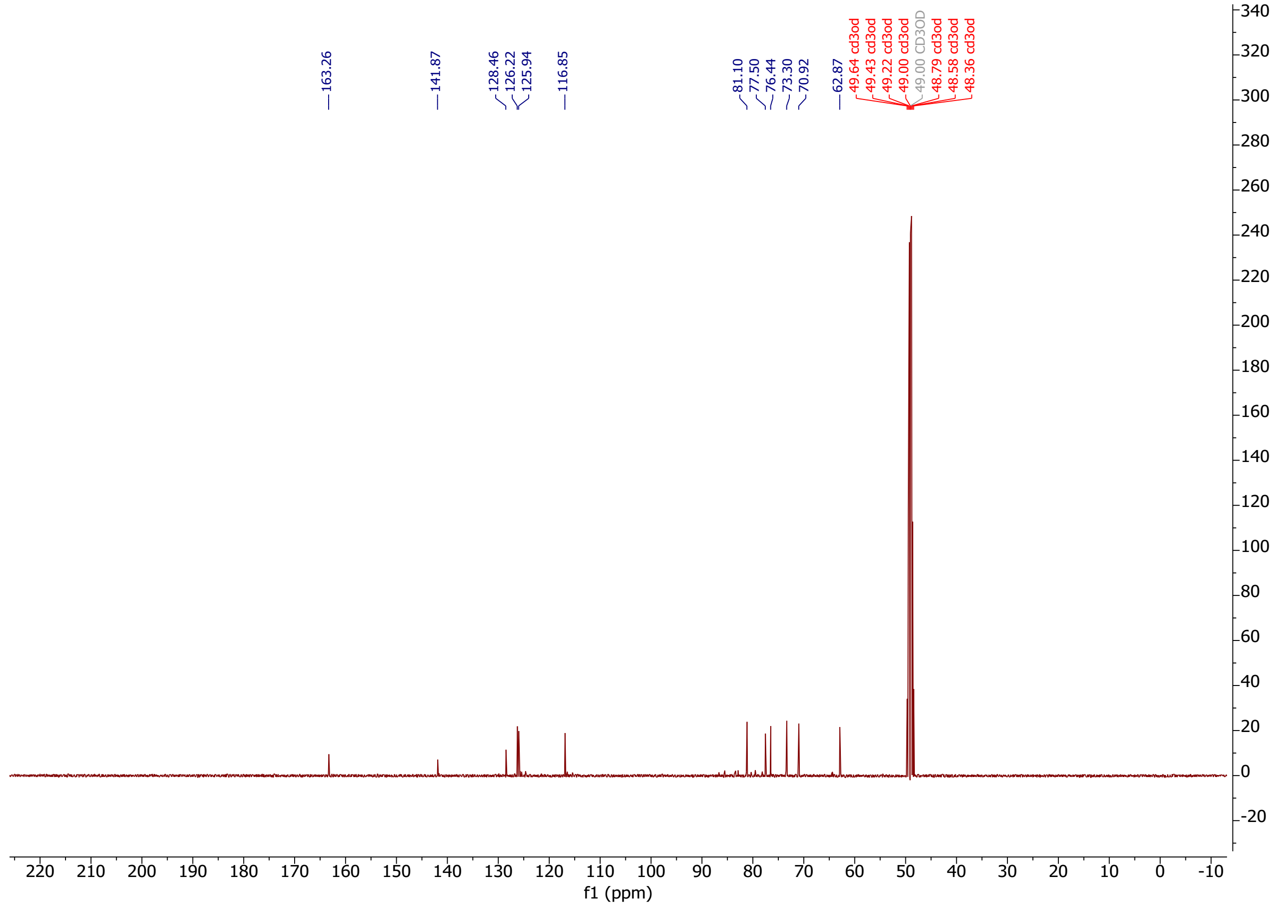
163.26
141.87
128.46
126.22
125.94
116.85
81.10
77.50
76.44
73.30
70.92
62.87
49.64 cd3od
49.43 cd3od
49.22 cd3od
49.00 cd3od
49.00 CD3OD
48.79 cd3od
48.58 cd3od
48.36 cd3od
f1 (ppm)

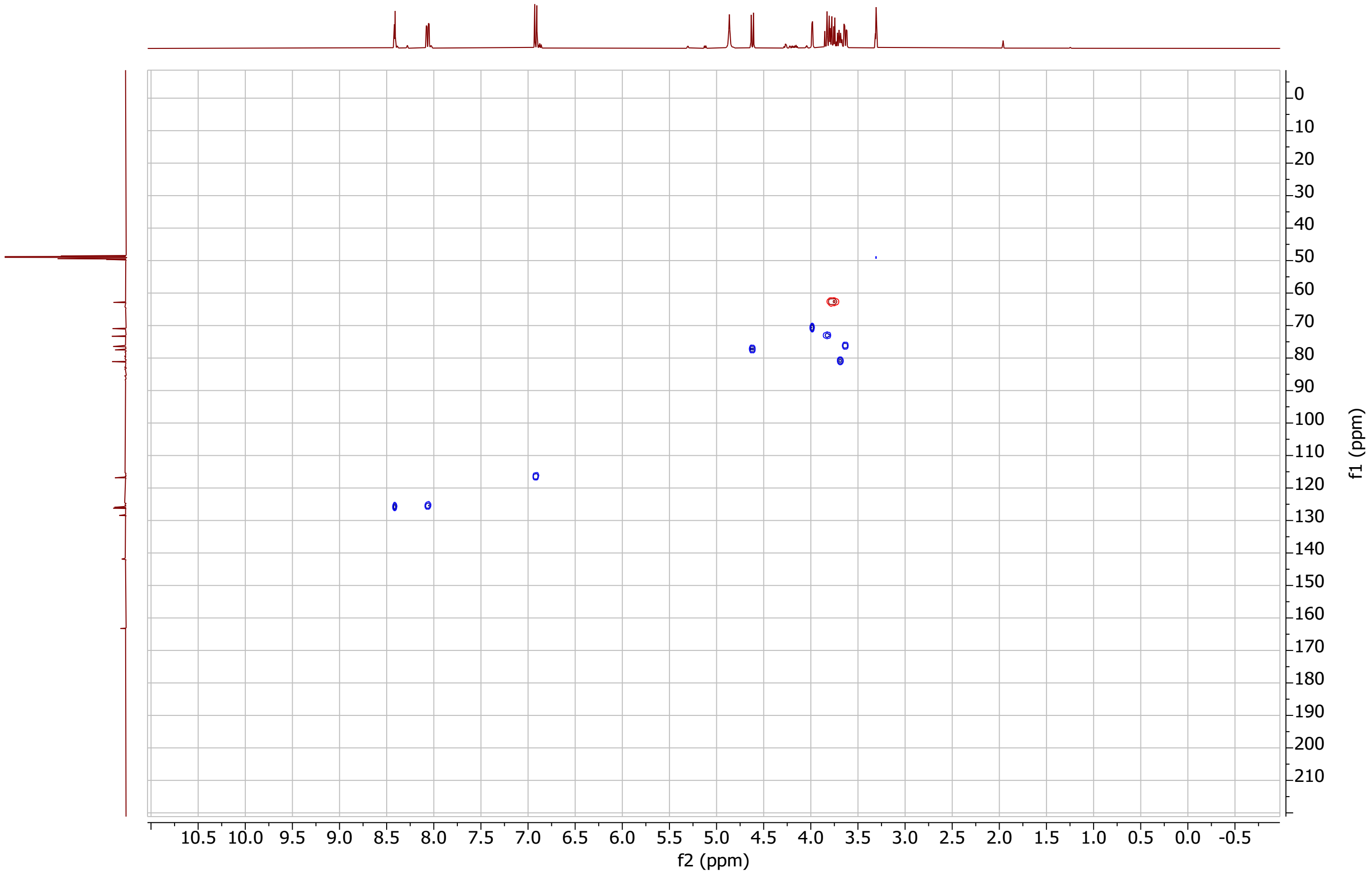
f2 (ppm)
f1 (ppm)

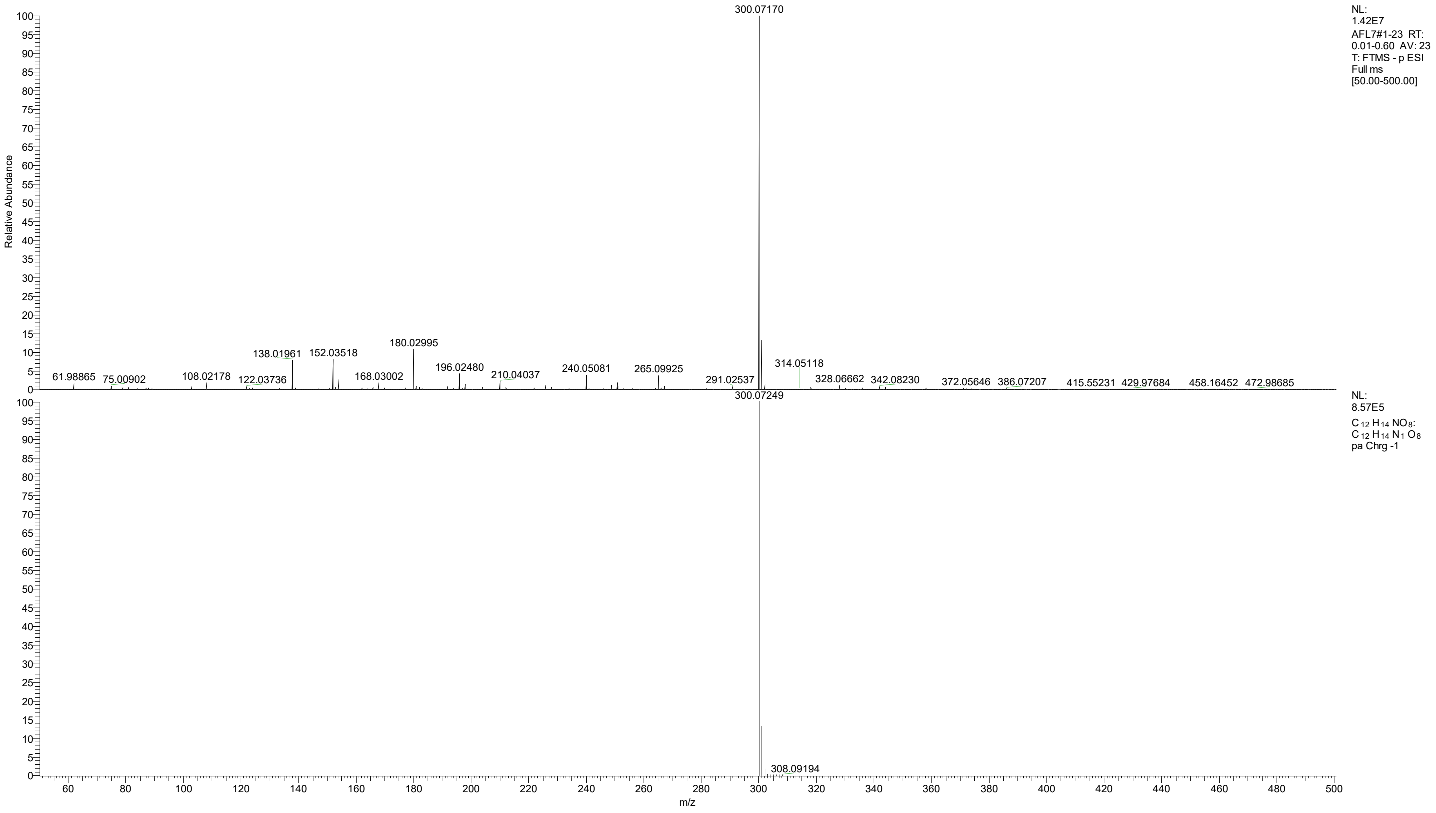
NL: 1.42E7
AFL7#1-23 RT: 0.01-0.60 AV: 23
T: FTMS - p ESI Full ms [50.00-500.00]
300.07170
180.02995
152.03518
138.01961
NL: 8.57E5
C 12 H 14 NO8: C 12 H 14 N 1 O 8
pa Chrg -1
300.07249
308.09194
Relative Abundance
m/z

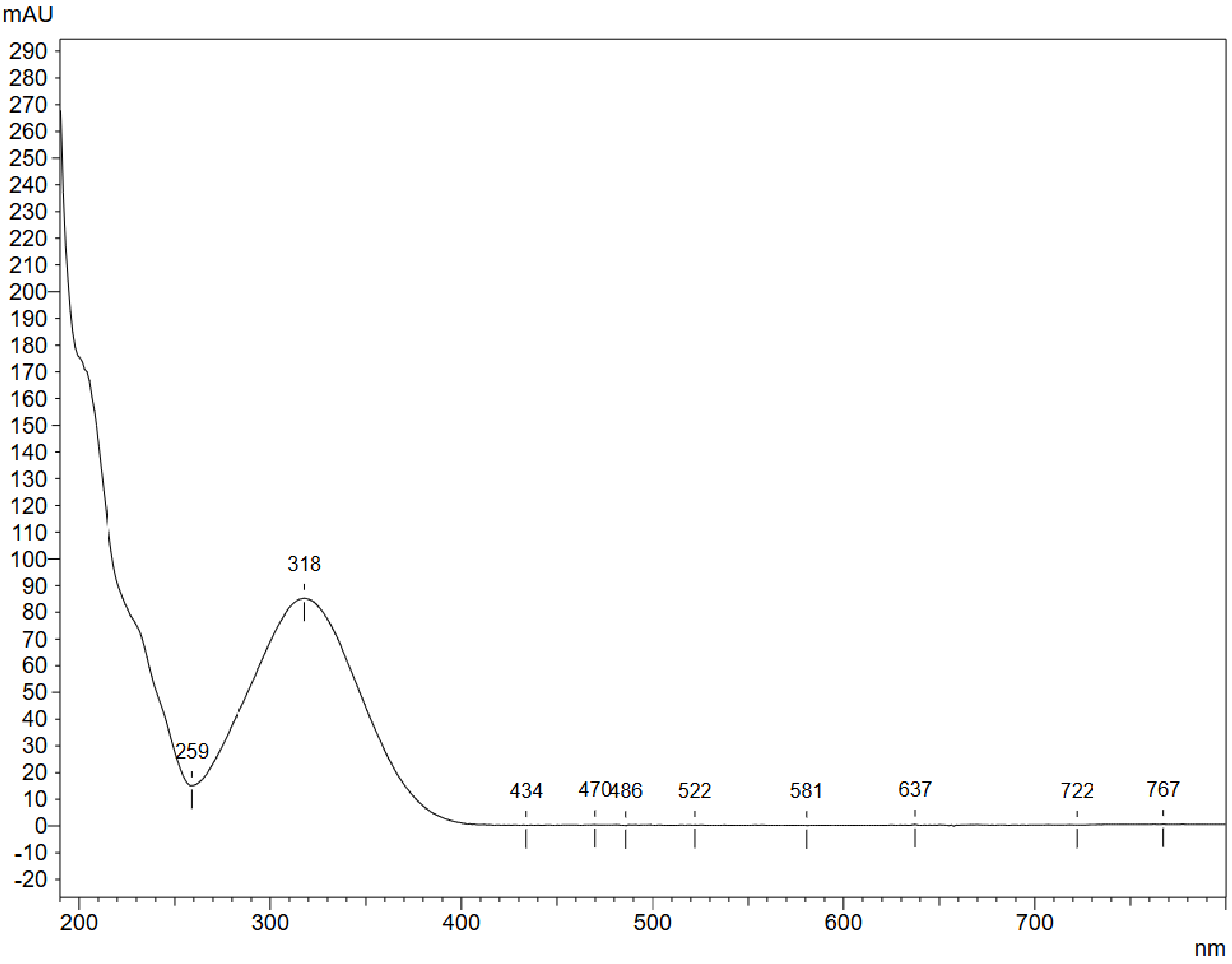
mAU
318
259
434
470
486
522
581
637
722
767
nm

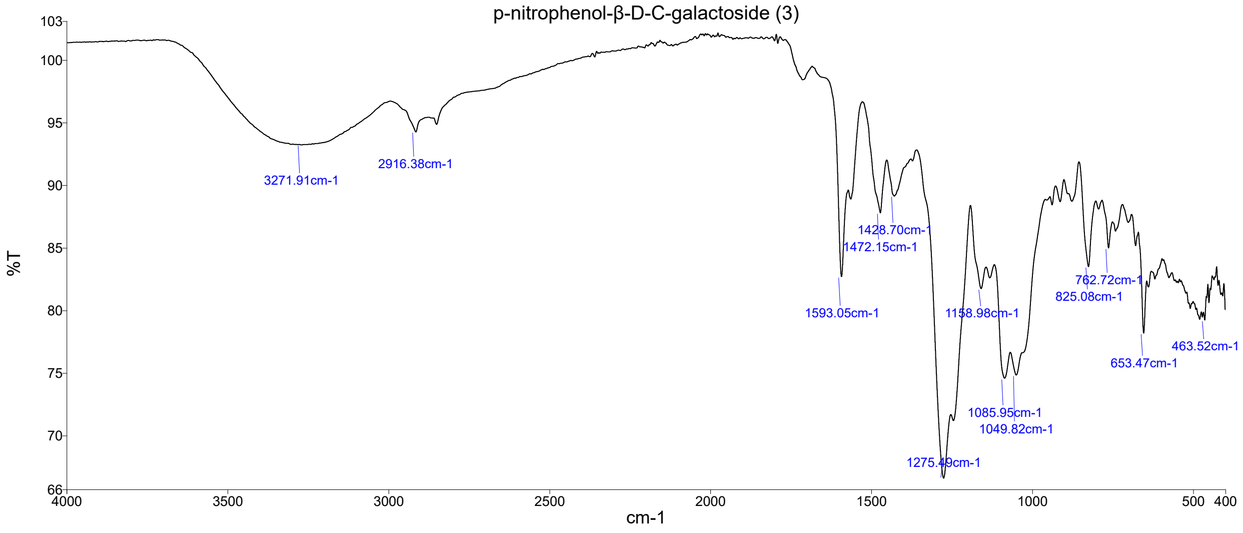
p-nitrophenol-β-D-C-galactoside (3)
%T
cm-1
3271.91cm-1
2916.38cm-1
1593.05cm-1
1472.15cm-1
1428.70cm-1
1275.49cm-1
1158.98cm-1
1085.95cm-1
1049.82cm-1
825.08cm-1
762.72cm-1
653.47cm-1
463.52cm-1

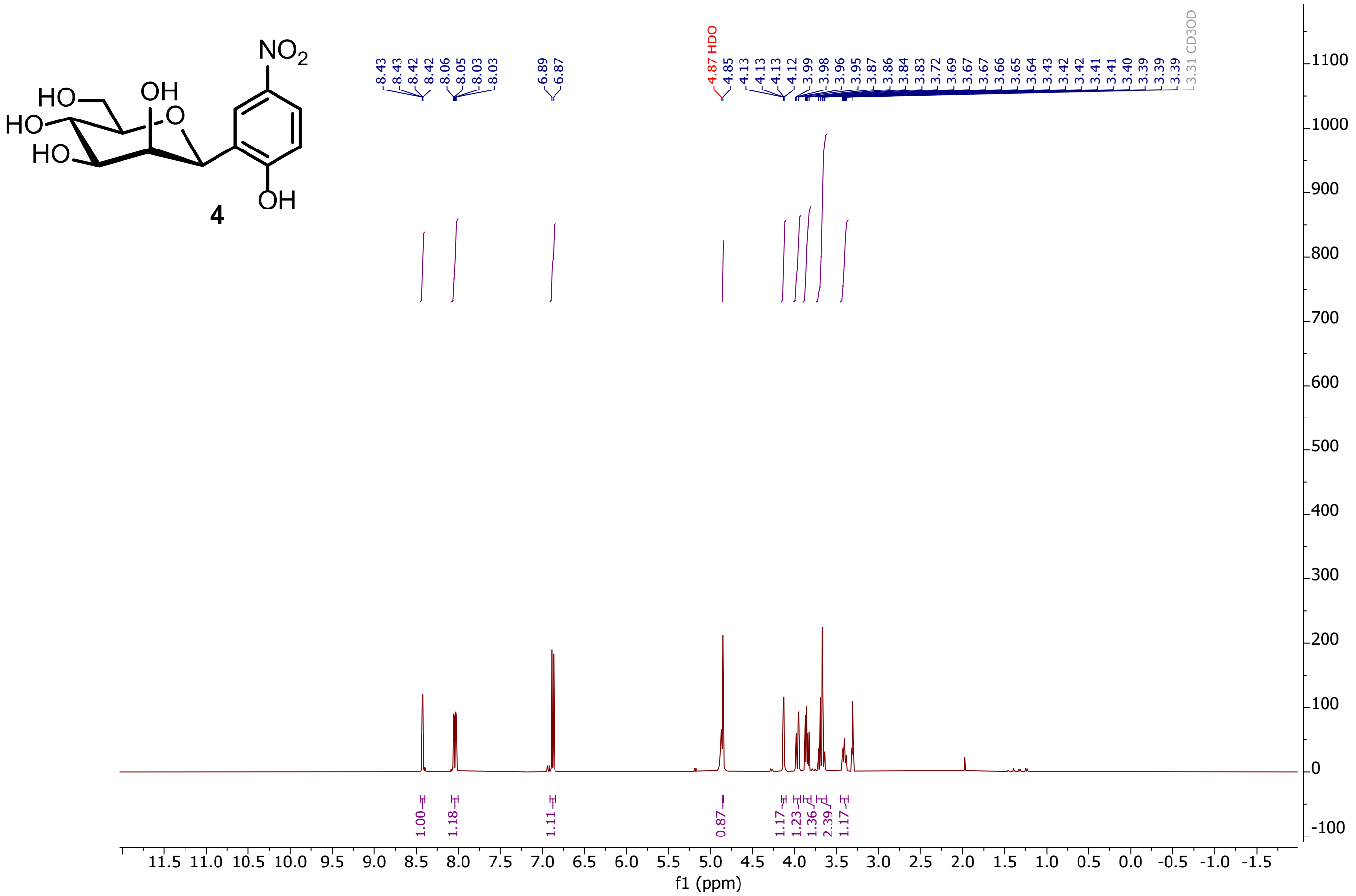
NO2
HO
OH
O
HO
HO
OH
4
8.43
8.43
8.42
8.42
8.06
8.05
8.03
8.03
6.89
6.87
4.87 HDO
4.85
4.13
4.13
4.13
4.12
3.99
3.98
3.96
3.95
3.87
3.86
3.84
3.83
3.72
3.69
3.67
3.67
3.66
3.65
3.64
3.43
3.42
3.42
3.41
3.41
3.40
3.39
3.39
3.39
3.31 CD3OD
1.00
1.18
1.11
0.87
1.17
1.23
1.36
2.39
1.17
f1 (ppm)

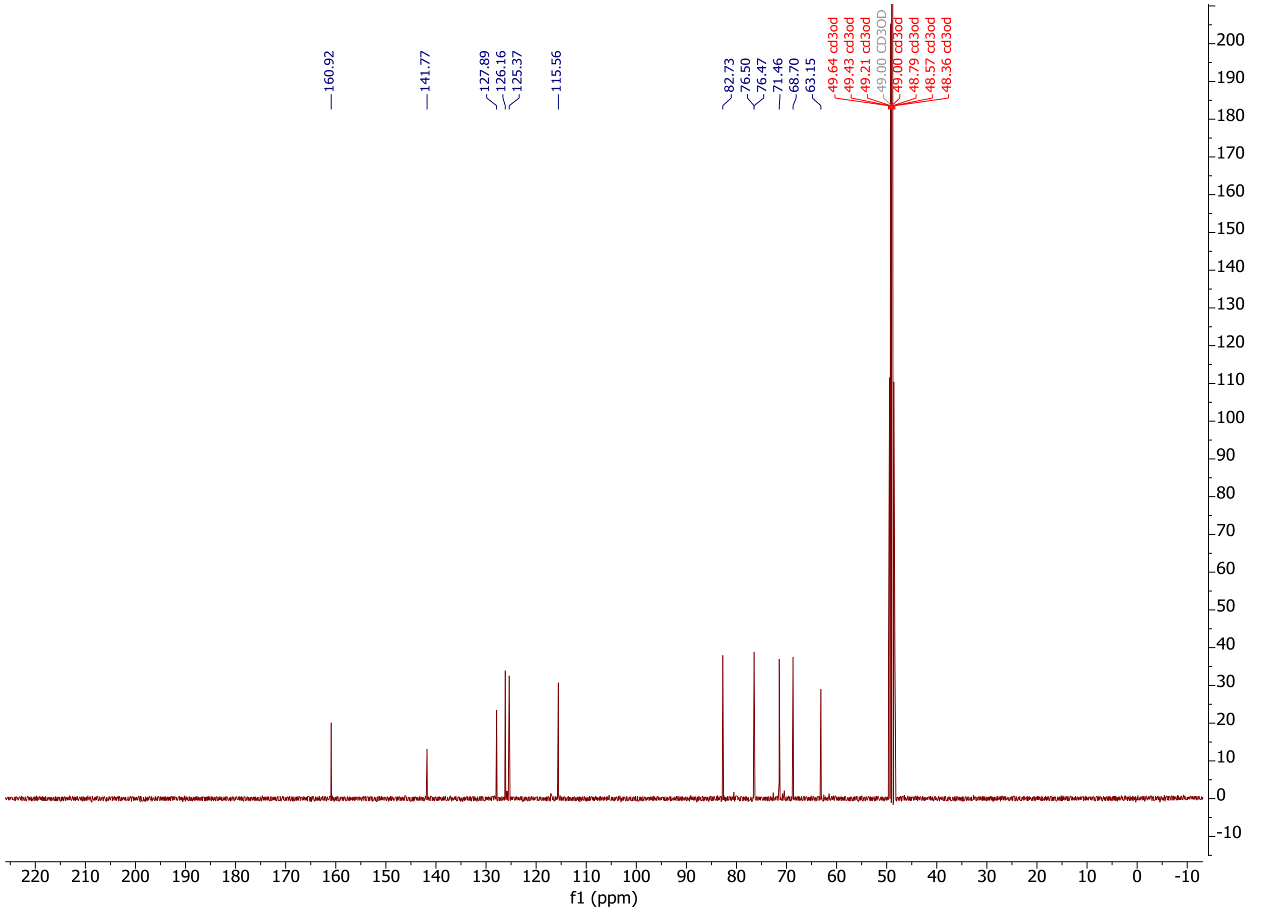
160.92
141.77
127.89
126.16
125.37
115.56
82.73
76.50
76.47
71.46
68.70
63.15
49.64 cd3od
49.43 cd3od
49.21 cd3od
49.00 CD3OD
49.00 cd3od
48.79 cd3od
48.57 cd3od
48.36 cd3od
f1 (ppm)

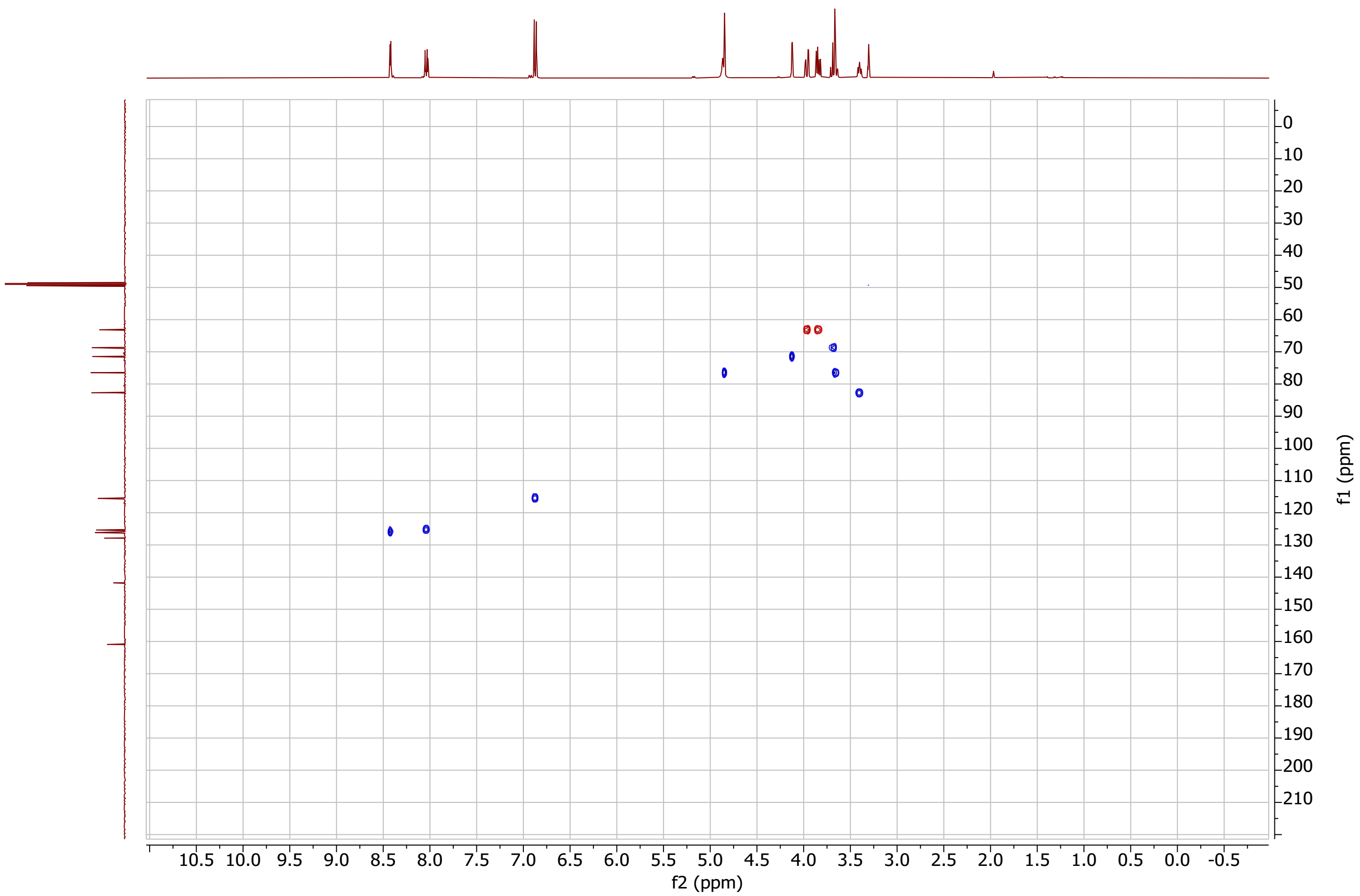

1D-NOESY showing coupling between 1'-H and 5'-H, indicating a β-configuration:

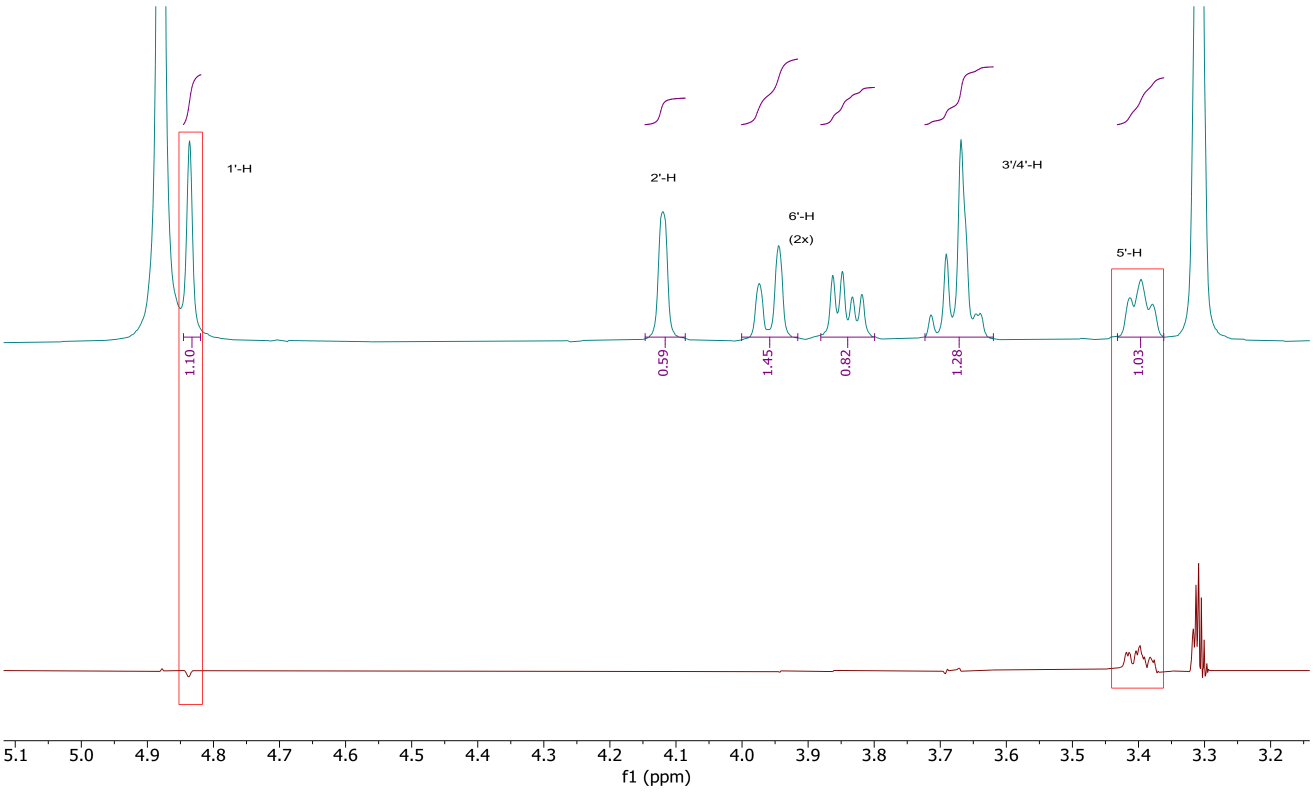

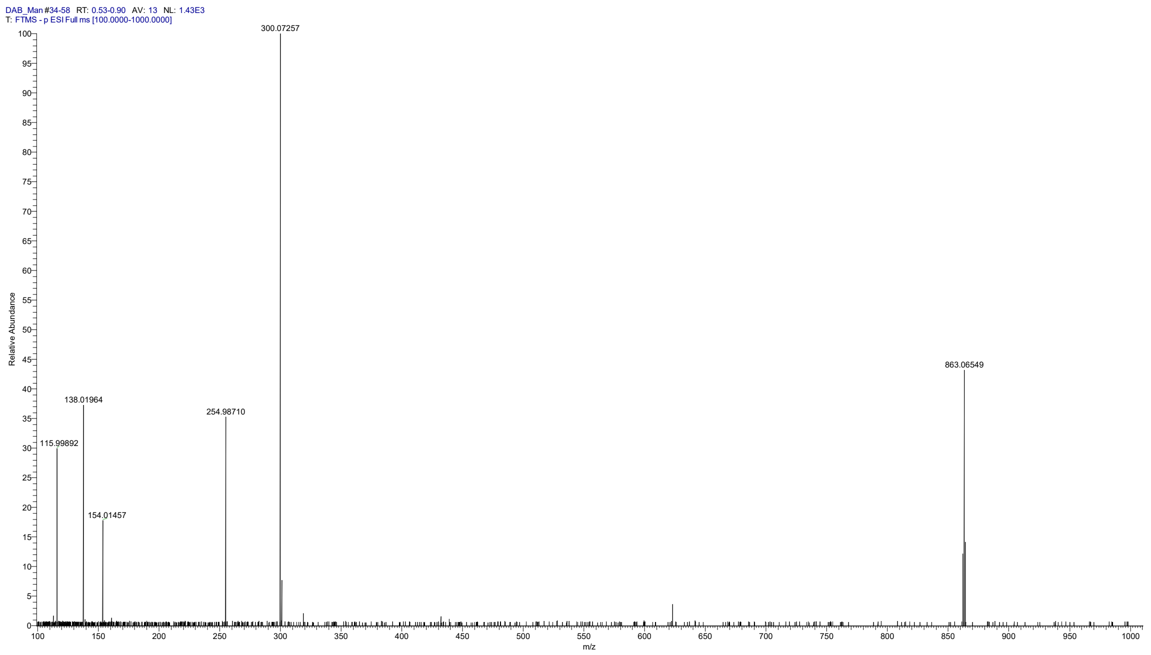
DAB_Man #34-58 RT: 0.53-0.90 AV: 13 NL: 1.43E3
T: FTMS - p ESI Full ms [100.0000-1000.0000]
300.07257
863.06549
138.01964
254.98710
115.99892
154.01457
Relative Abundance
m/z

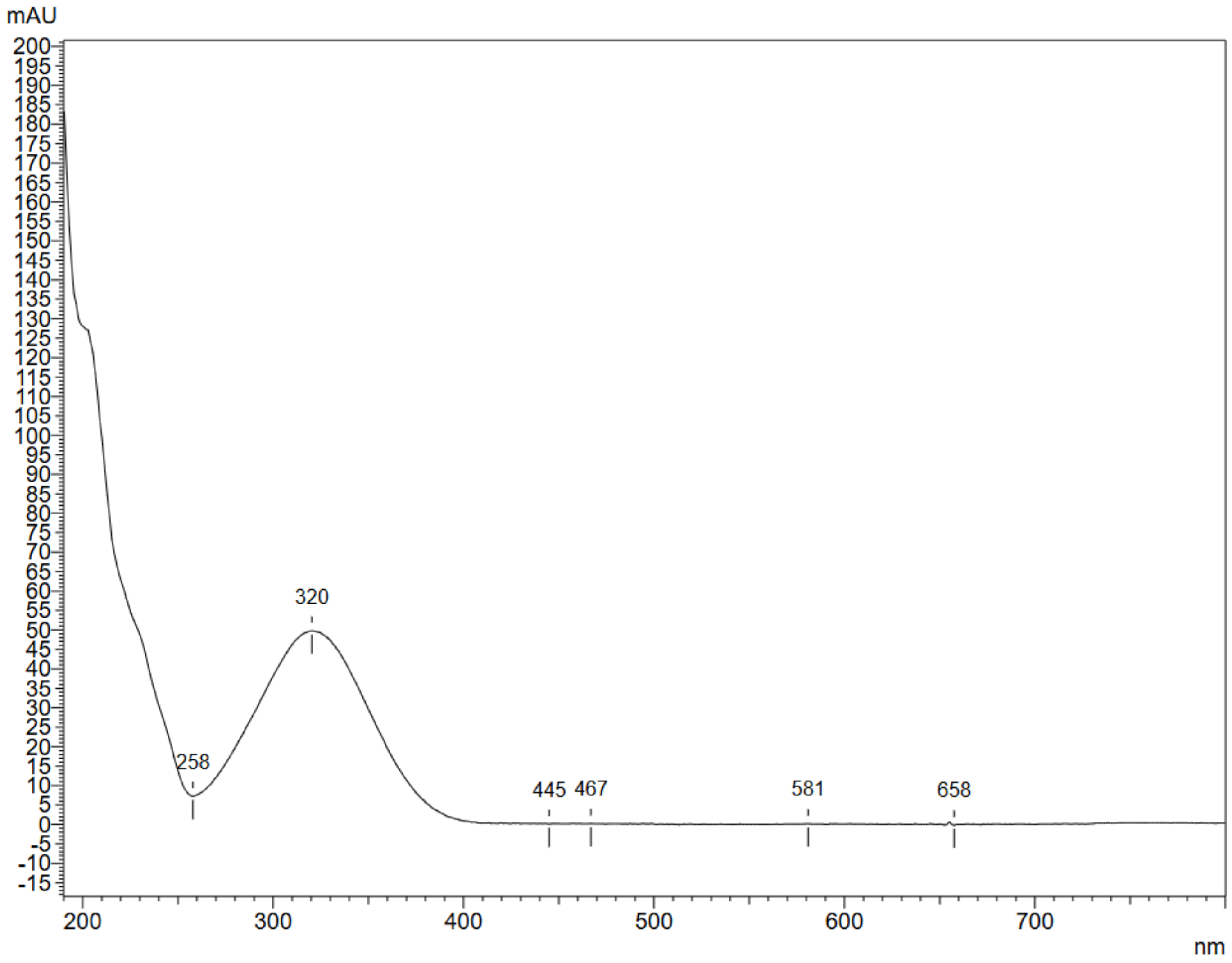
mAU
320
258
445
467
581
658
nm

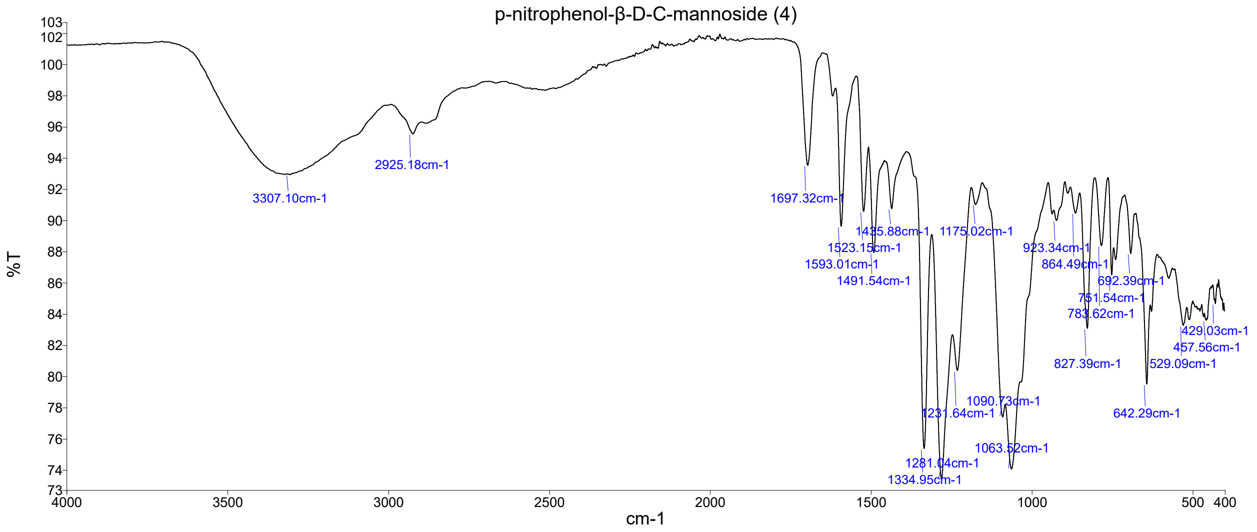
p-nitrophenol-β-D-C-mannoside (4)
%T
cm-1
3307.10cm-1
2925.18cm-1
1697.32cm-1
1593.01cm-1
1523.15cm-1
1491.54cm-1
1435.88cm-1
1334.95cm-1
1281.04cm-1
1231.64cm-1
1175.02cm-1
1090.73cm-1
1063.52cm-1
923.34cm-1
864.49cm-1
827.39cm-1
783.62cm-1
751.54cm-1
692.39cm-1
642.29cm-1
529.09cm-1
457.56cm-1
429.03cm-1

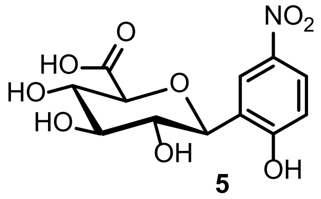
NO2
HO
O
HO
O
HO
OH
OH

5

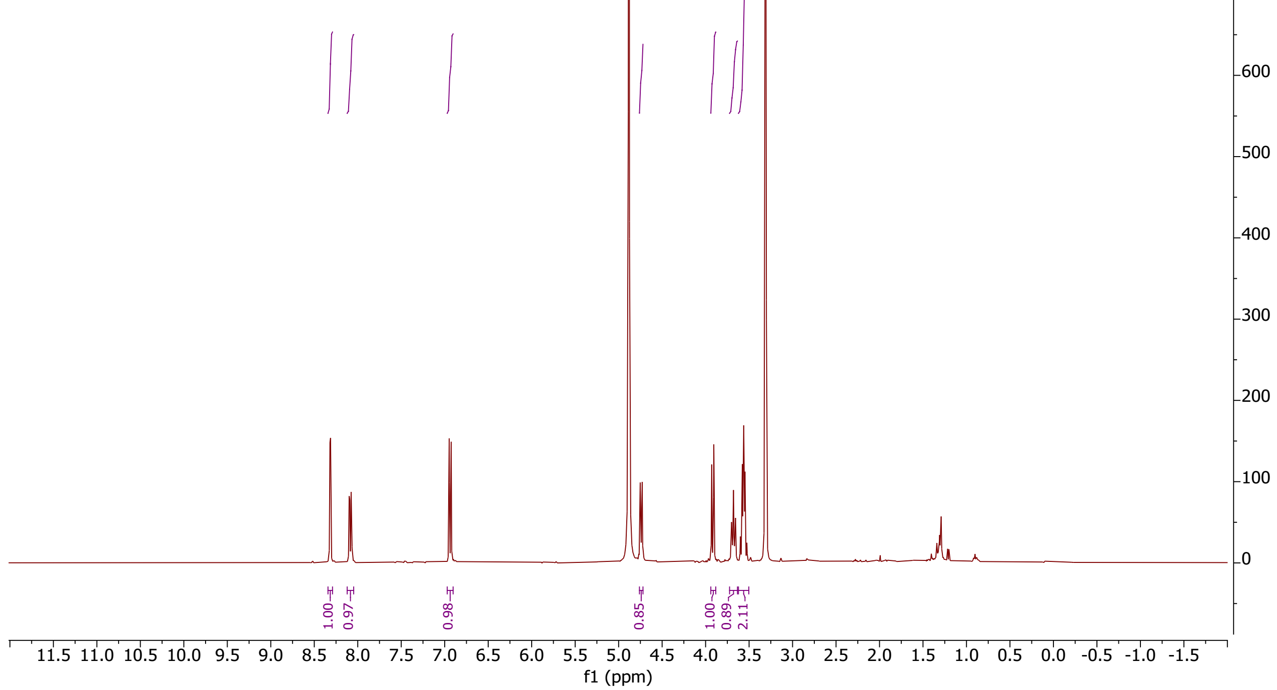
8.32
8.31
8.10
8.09
8.08
8.07
6.95
6.93
4.75
4.73
3.93
3.91
3.70
3.68
3.66
3.60
3.58
3.57
3.56
3.55
3.55
3.53
3.31 CD3OD
1.00
0.97
0.98
0.85
1.00
0.89
2.11
f1 (ppm)

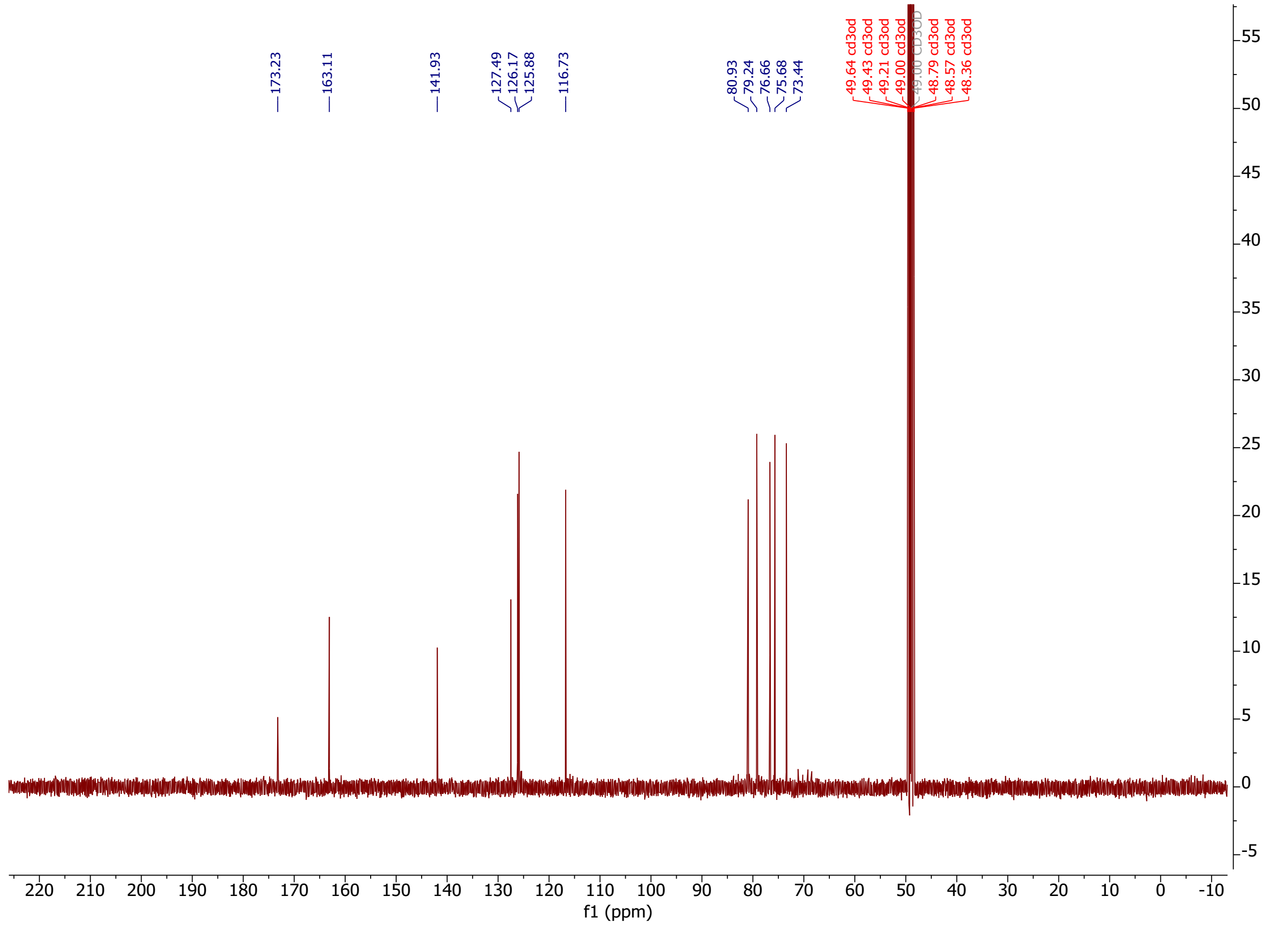
173.23
163.11
141.93
127.49
126.17
125.88
116.73
80.93
79.24
76.66
75.68
73.44
49.64 cd3od
49.43 cd3od
49.21 cd3od
49.00 CD3OD
48.79 cd3od
48.57 cd3od
48.36 cd3od
f1 (ppm)

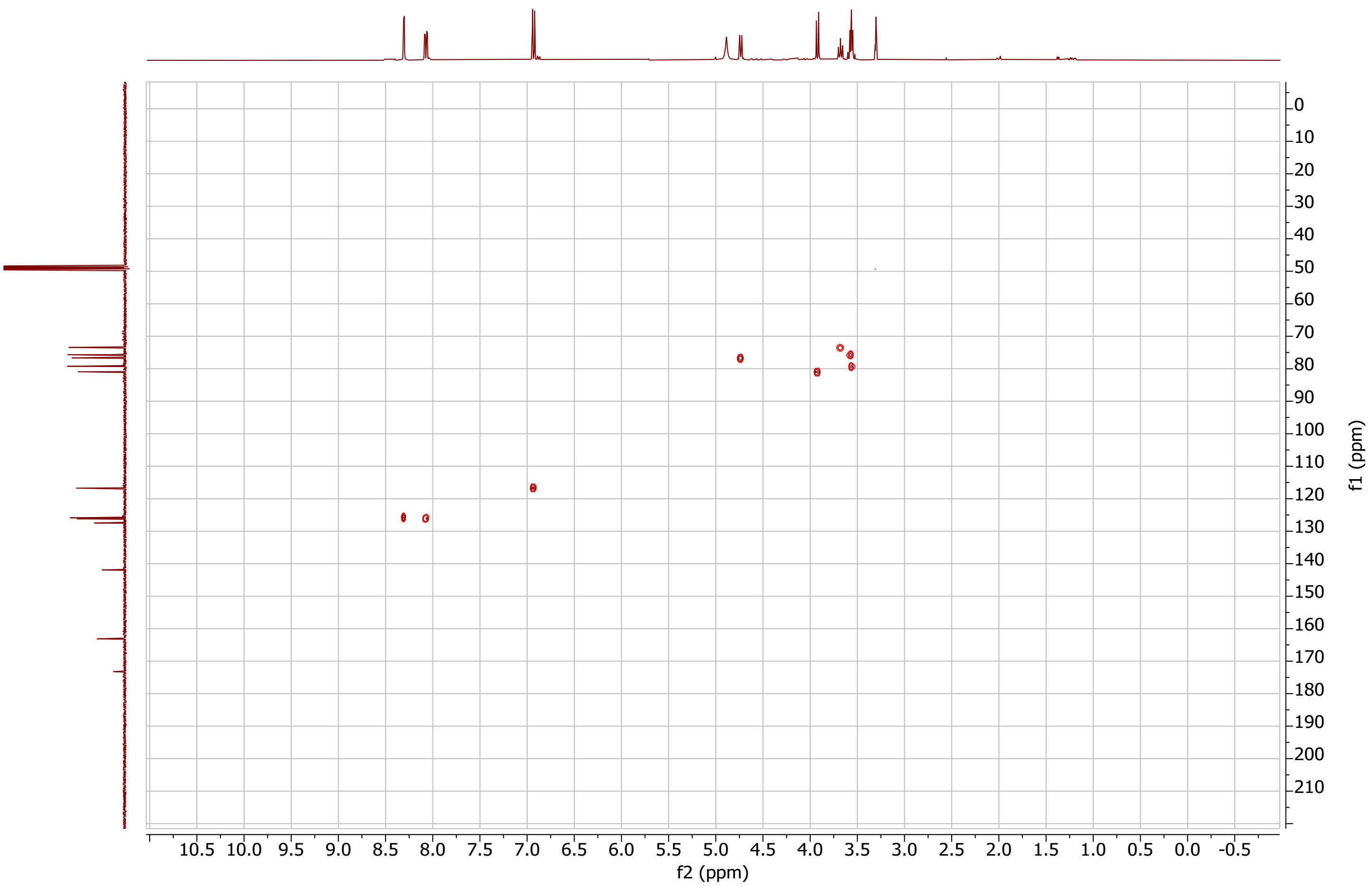
10.5 10.0 9.5 9.0 8.5 8.0 7.5 7.0 6.5 6.0 5.5 5.0 4.5 4.0 3.5 3.0 2.5 2.0 1.5 1.0 0.5 0.0 -0.5
f2 (ppm)
0 10 20 30 40 50 60 70 80 90 100 110 120 130 140 150 160 170 180 190 200 210
f1 (ppm)

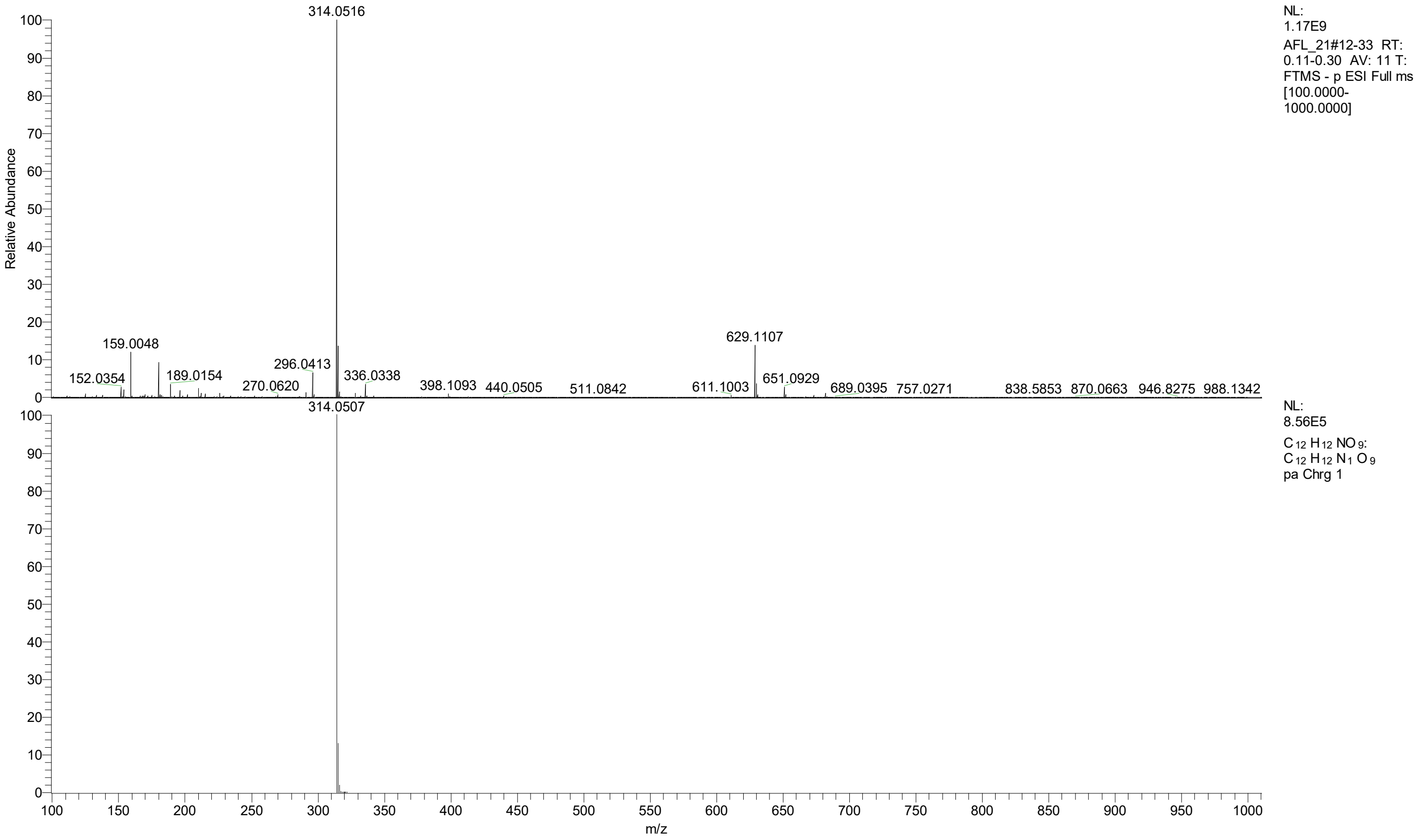
314.0516
NL:
1.17E9
AFL_21#12-33 RT:
0.11-0.30 AV: 11 T:
FTMS - p ESI Full ms
[100.0000-
1000.0000]
Relative Abundance
159.0048
152.0354
189.0154
270.0620
296.0413
336.0338
398.1093
440.0505
511.0842
611.1003
629.1107
651.0929
689.0395
757.0271
838.5853
870.0663
946.8275
988.1342
314.0507
NL:
8.56E5
C 12 H 12 NO 9:
C 12 H 12 N 1 O 9
pa Chrg 1
m/z

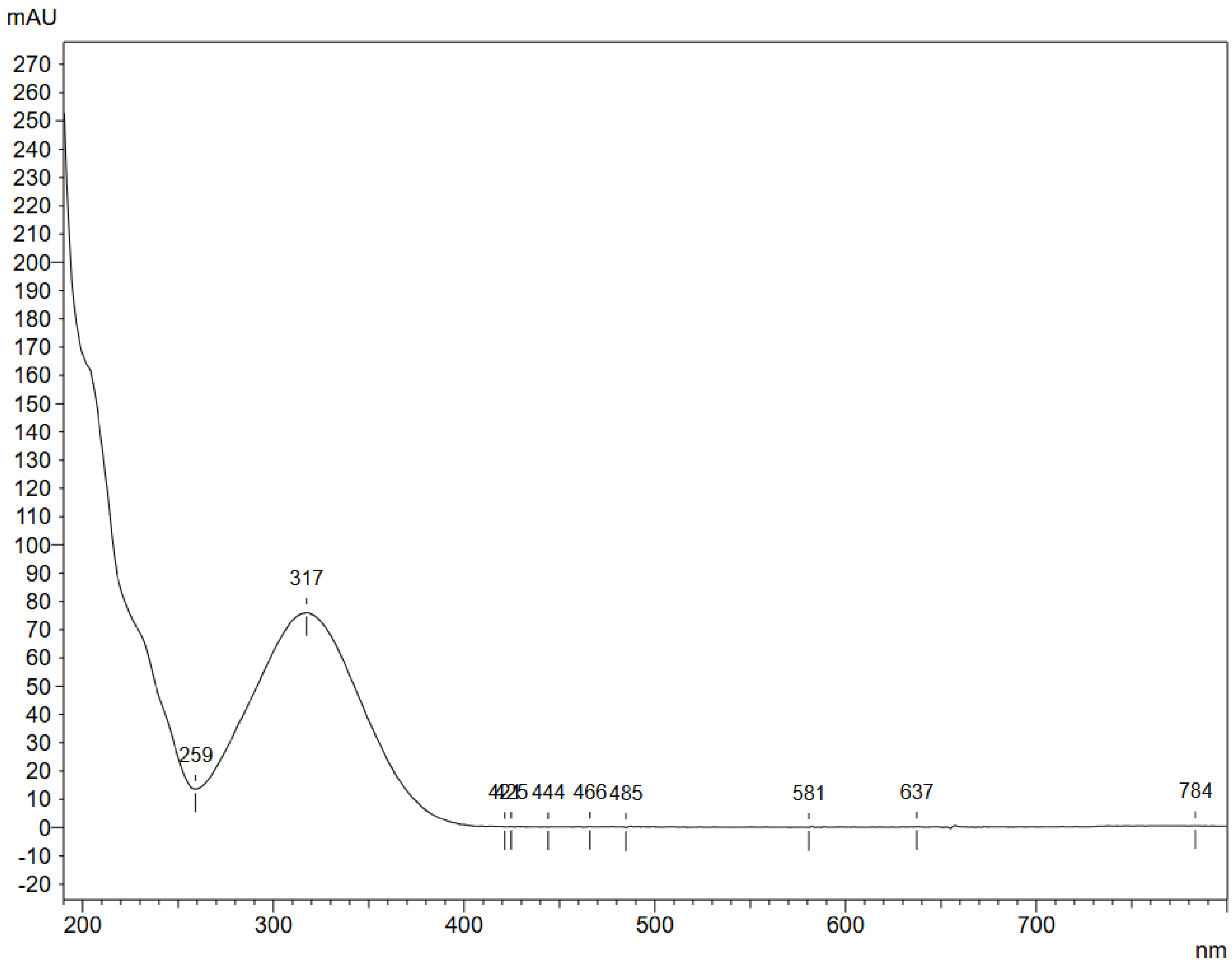
mAU
317
259
421
425
444
466
485
581
637
784
nm

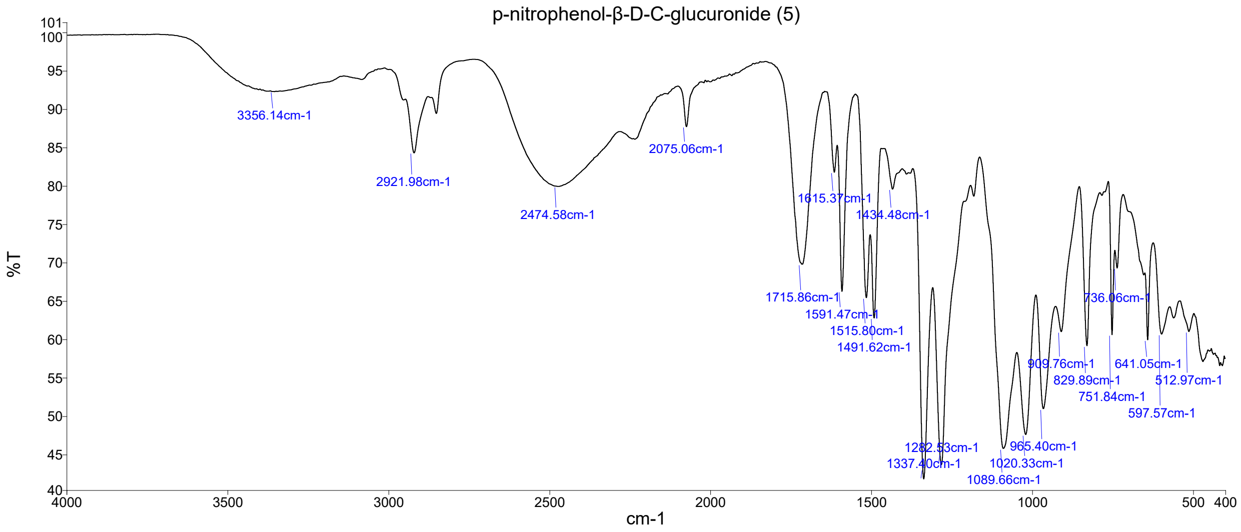
p-nitrophenol-β-D-C-glucuronide (5)
%T
cm-1
3356.14cm-1
2921.98cm-1
2474.58cm-1
2075.06cm-1
1615.37cm-1
1434.48cm-1
1715.86cm-1
1591.47cm-1
1515.80cm-1
1491.62cm-1
1282.53cm-1
1337.40cm-1
1089.66cm-1
1020.33cm-1
965.40cm-1
909.76cm-1
829.89cm-1
751.84cm-1
736.06cm-1
641.05cm-1
597.57cm-1
512.97cm-1

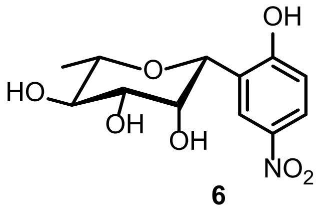
OH
HO
O
OH
OH
NO2

6

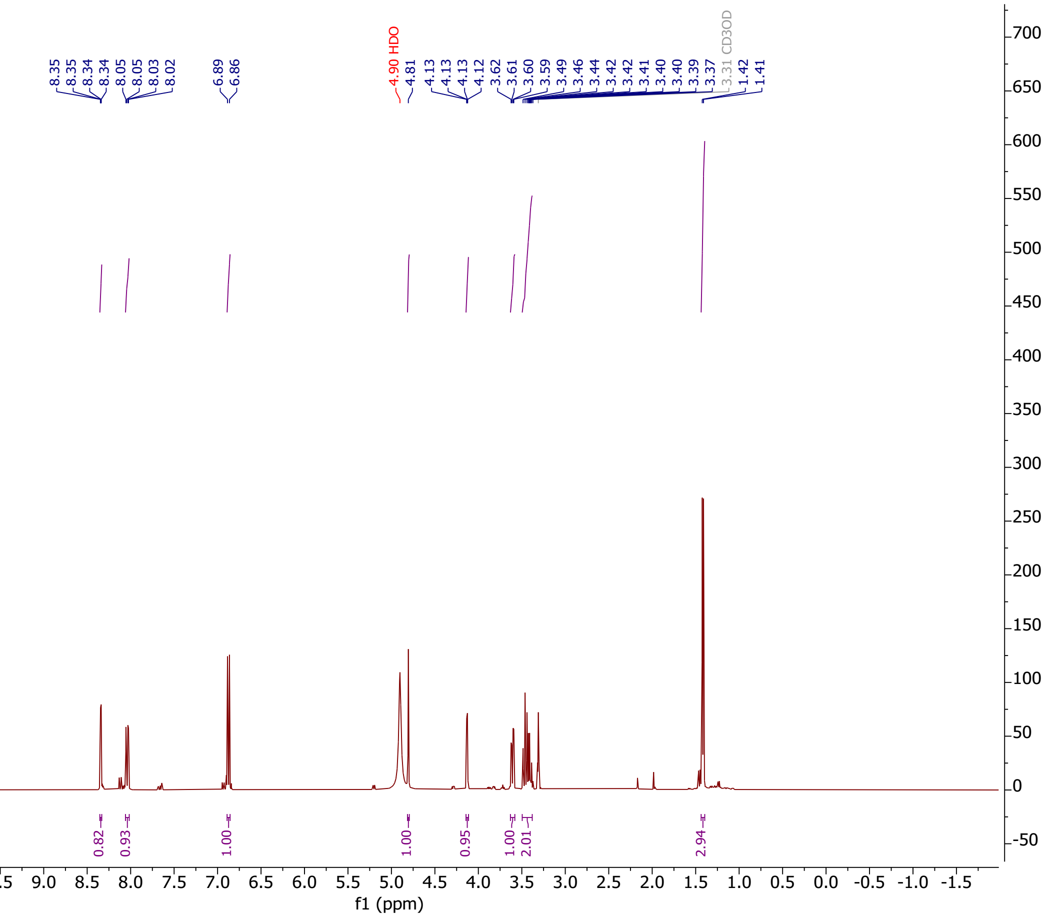
8.35
8.35
8.34
8.34
8.05
8.05
8.03
8.02
6.89
6.86
4.90 HDO
4.81
4.13
4.13
4.13
4.12
3.62
3.61
3.60
3.59
3.49
3.46
3.44
3.42
3.42
3.41
3.40
3.40
3.39
3.37
3.31 CD3OD
1.42
1.41
0.82
0.93
1.00
1.00
0.95
1.00
2.01
2.94
f1 (ppm)

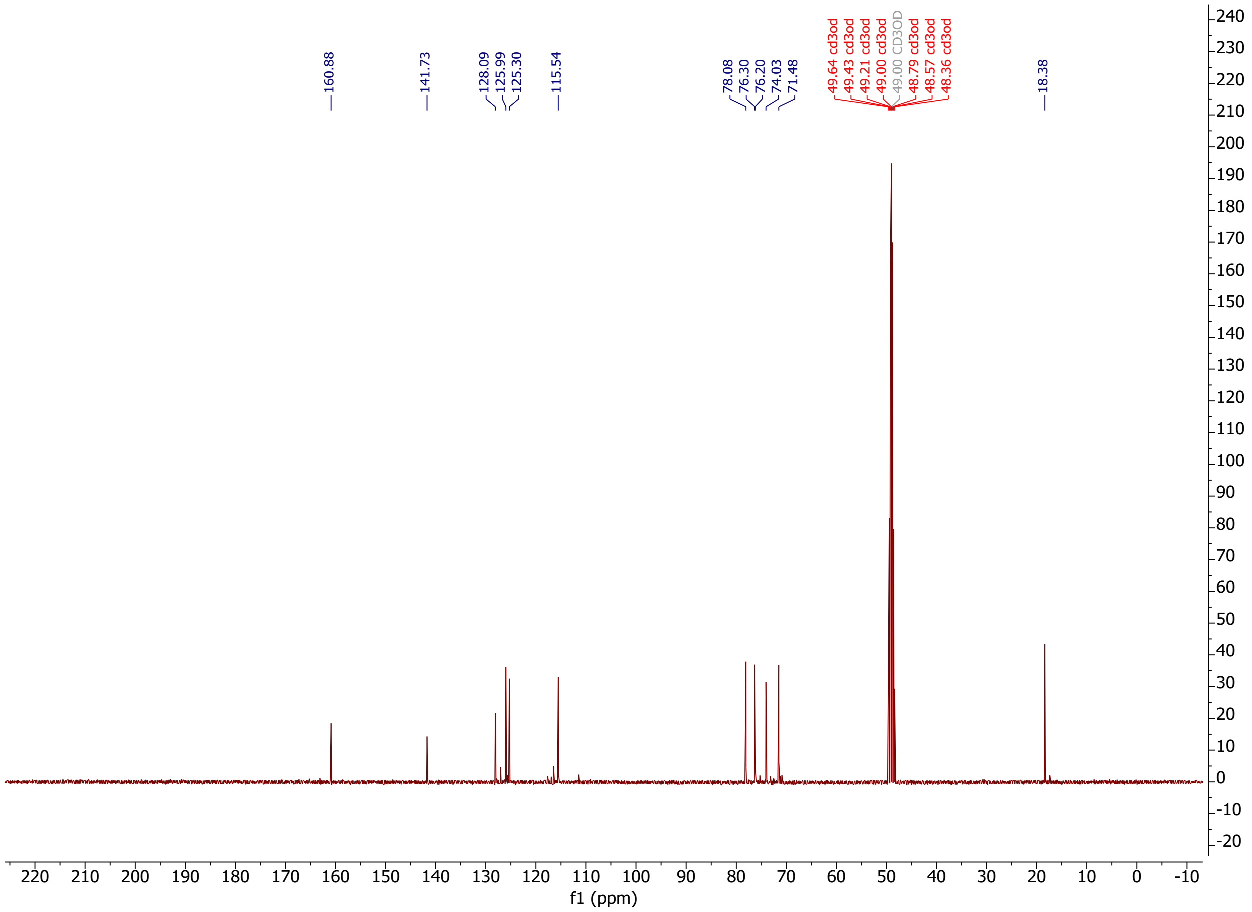
160.88
141.73
128.09
125.99
125.30
115.54
78.08
76.30
76.20
74.03
71.48
49.64 cd3od
49.43 cd3od
49.21 cd3od
49.00 CD3OD
48.79 cd3od
48.57 cd3od
48.36 cd3od
18.38
f1 (ppm)

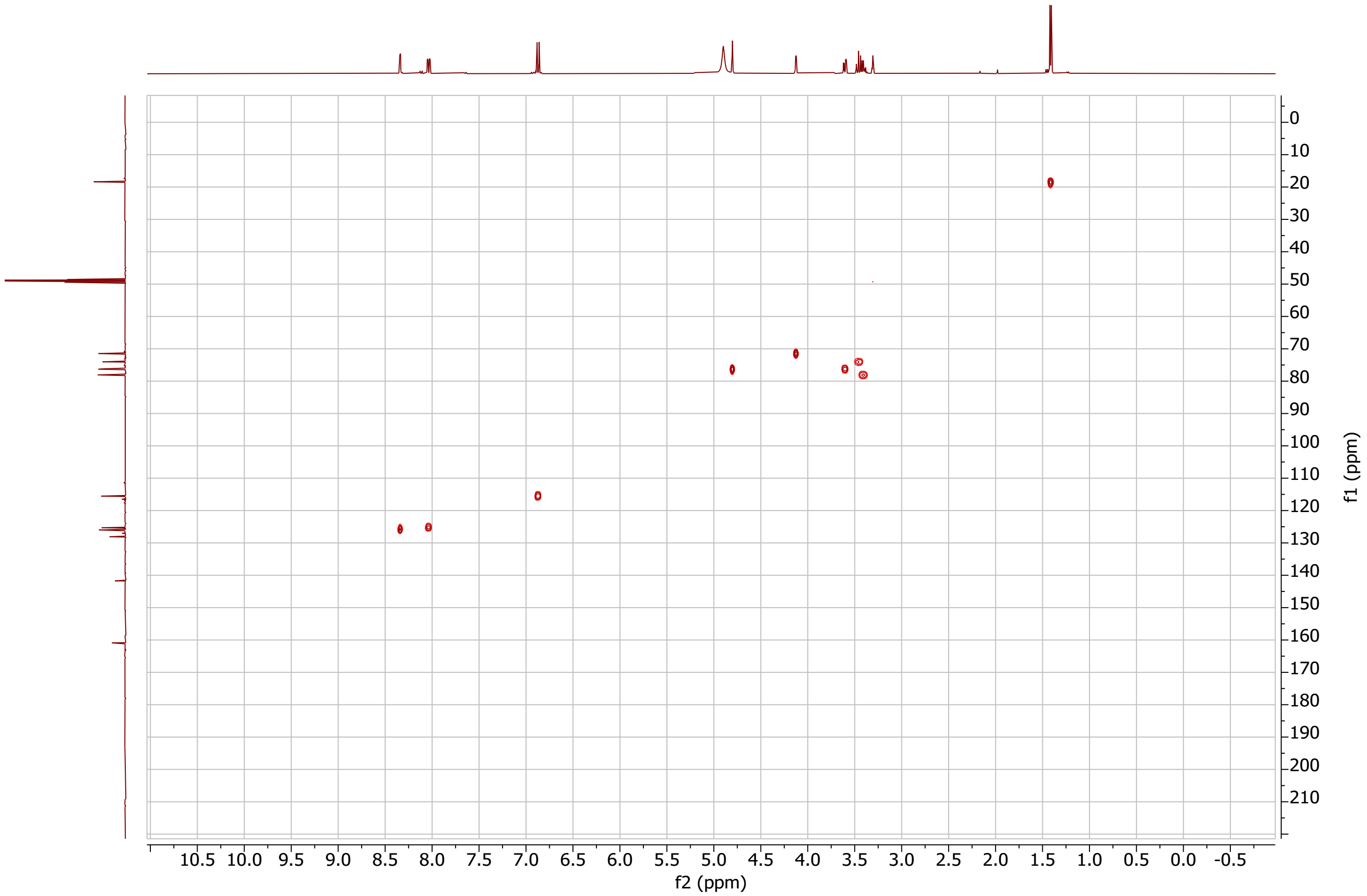
f2 (ppm)
f1 (ppm)

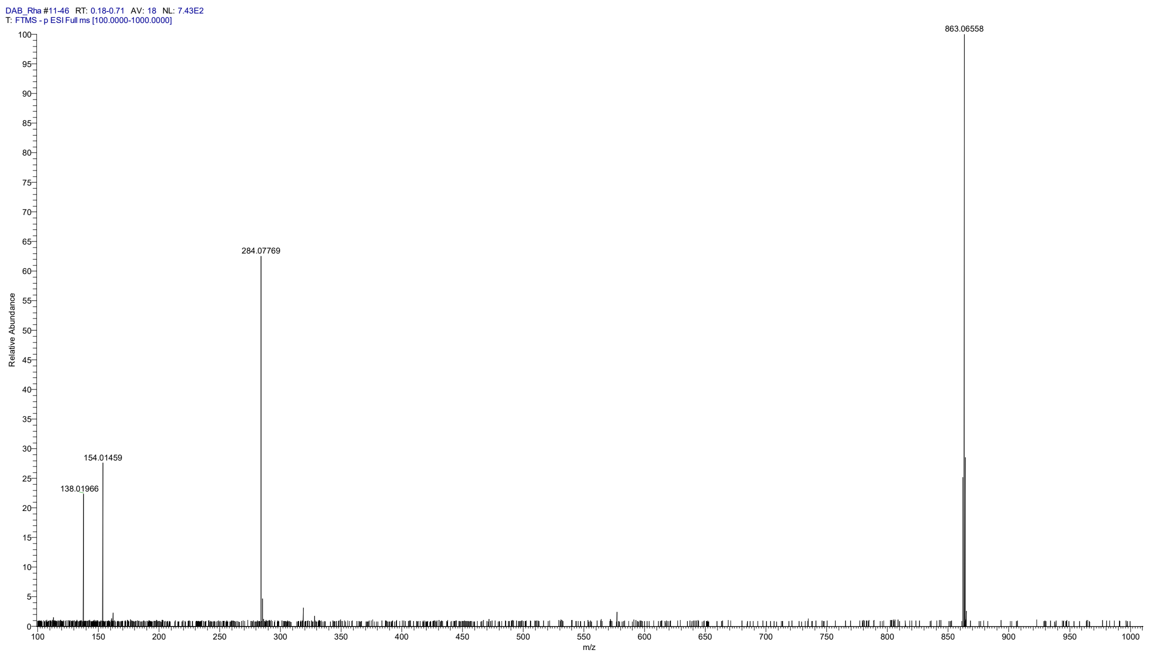
DAB_Rha #11-46 RT: 0.18-0.71 AV: 18 NL: 7.43E2
T: FTMS - p ESI Full ms [100.0000-1000.0000]
863.06558
284.07769
154.01459
138.01966
Relative Abundance
m/z

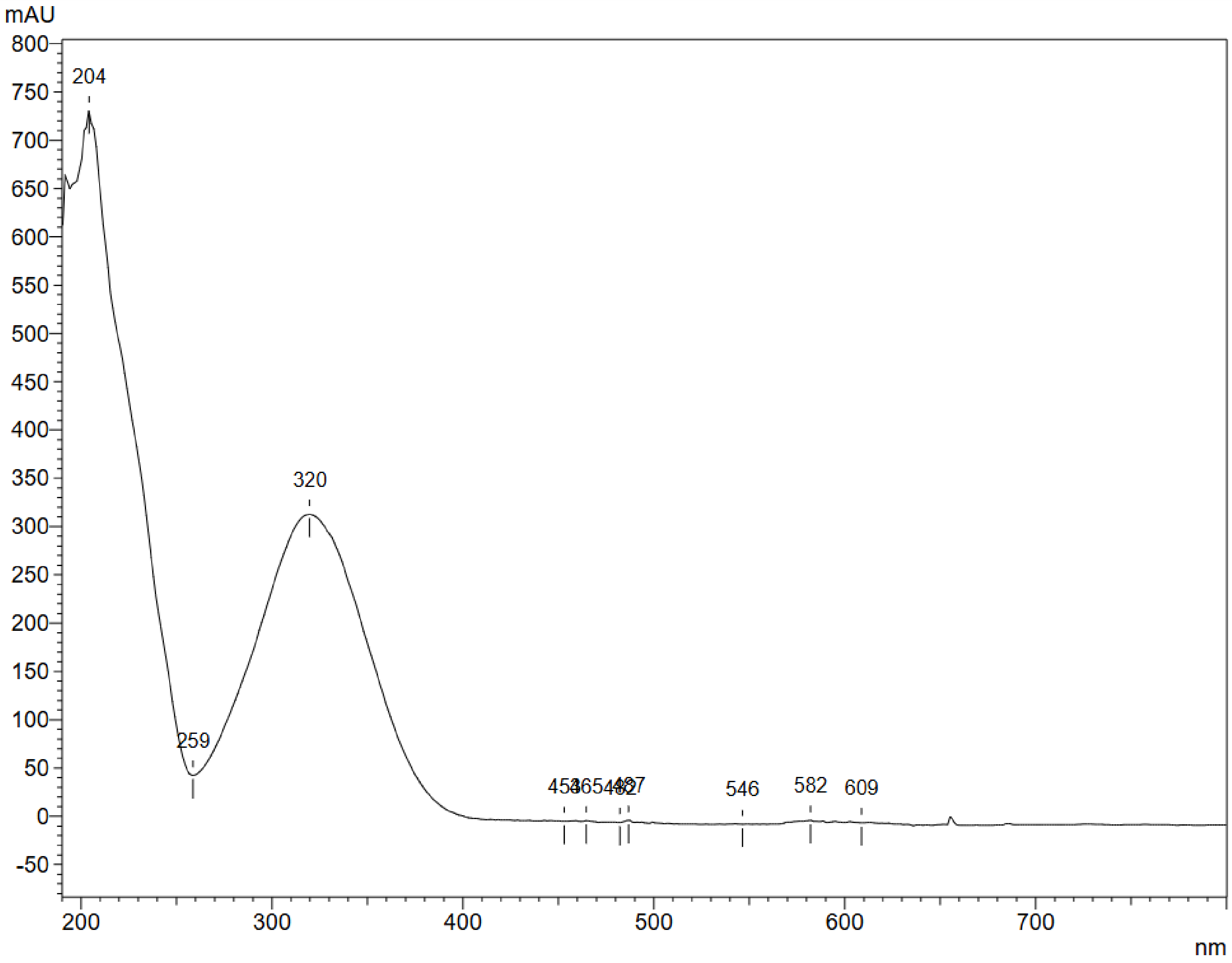

p-nitrophenol-β-L-C-rhamnoside (6)

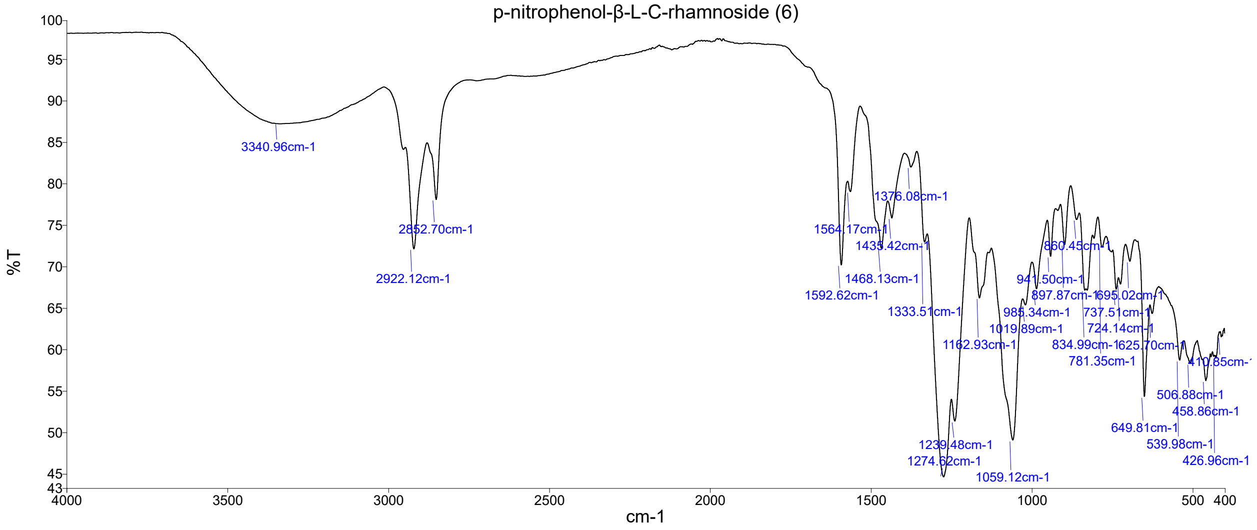

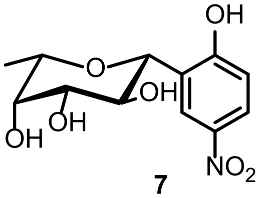
OH
O
OH
OH
OH
NO2

7

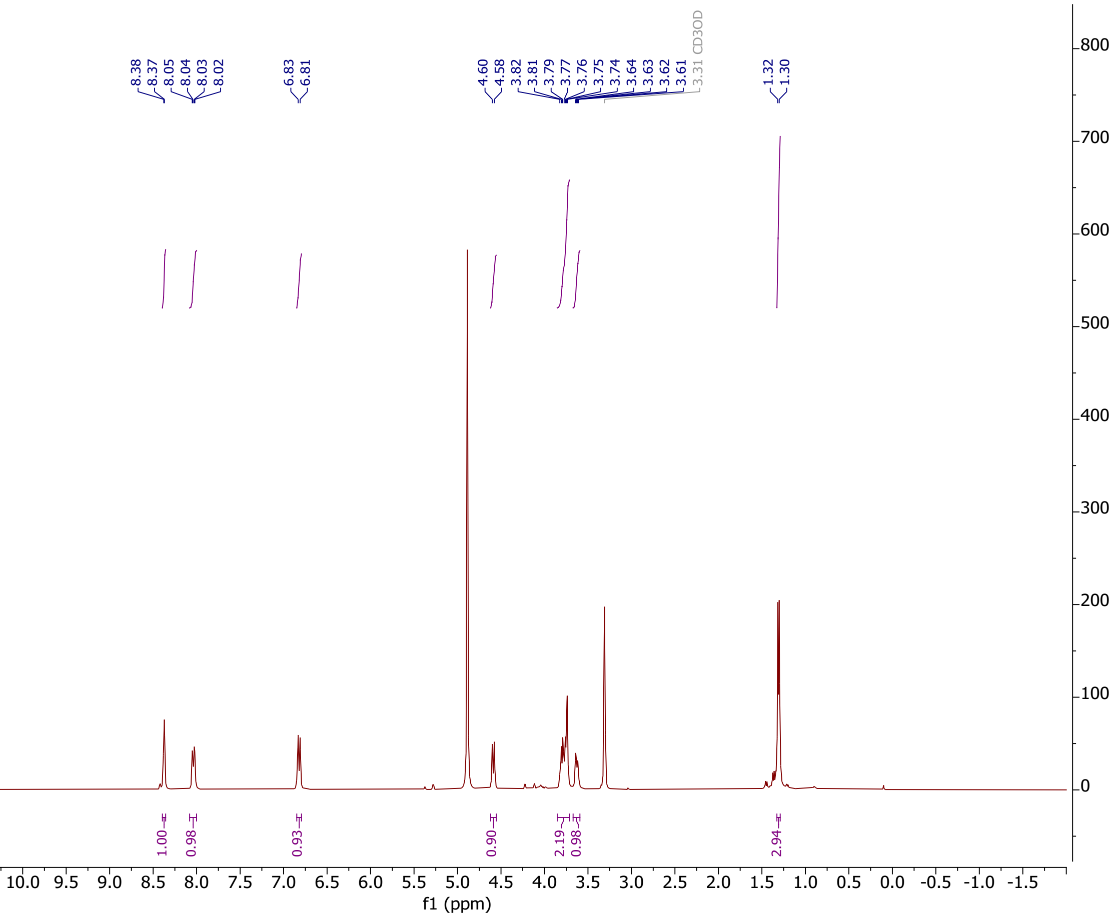
8.38
8.37
8.05
8.04
8.03
8.02
6.83
6.81
4.60
4.58
3.82
3.81
3.79
3.77
3.76
3.75
3.74
3.64
3.63
3.62
3.61
3.31 CD3OD
1.32
1.30
1.00
0.98
0.93
0.90
2.19
0.98
2.94
f1 (ppm)

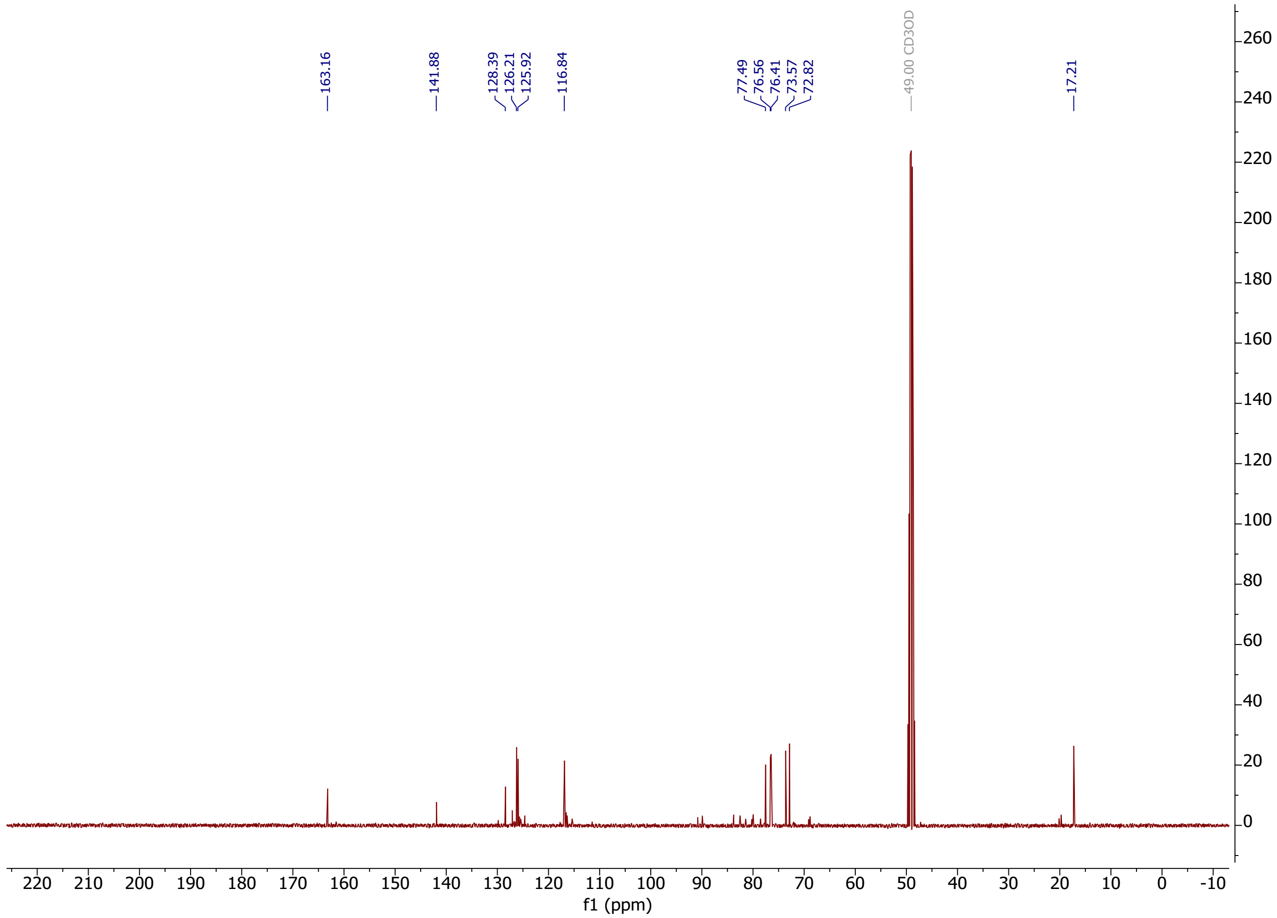
163.16
141.88
128.39
126.21
125.92
116.84
77.49
76.56
76.41
73.57
72.82
49.00 CD3OD
17.21
f1 (ppm)

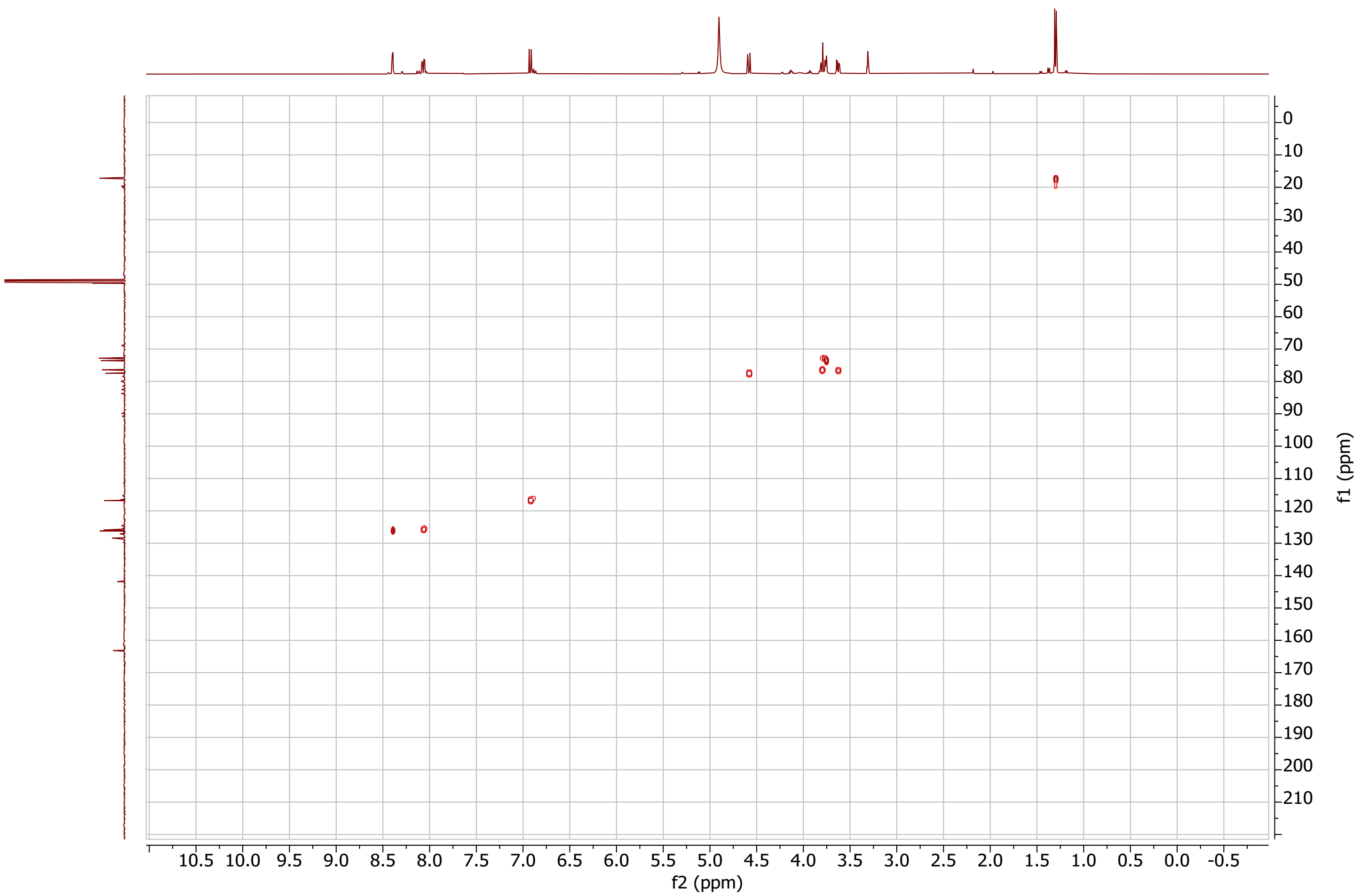
f1 (ppm)
f2 (ppm)

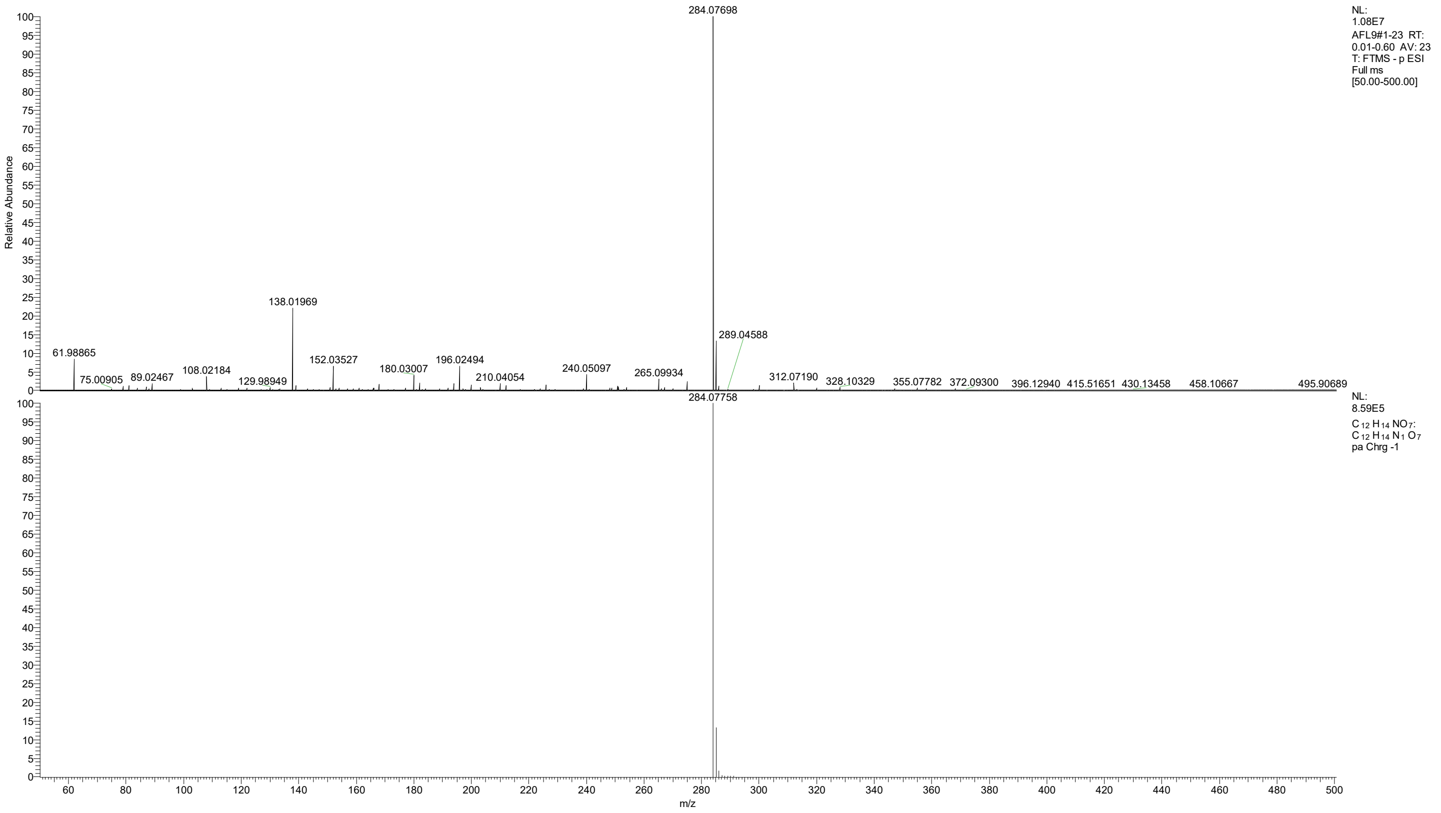
284.07698
NL:
1.08E7
AFL9#1-23 RT:
0.01-0.60 AV: 23
T: FTMS - p ESI
Full ms
[50.00-500.00]
Relative Abundance
138.01969
61.98865
75.00905
89.02467
108.02184
129.98949
152.03527
180.03007
196.02494
210.04054
240.05097
265.09934
289.04588
312.07190
328.10329
355.07782
372.09300
396.12940
415.51651
430.13458
458.10667
495.90689
284.07758
NL:
8.59E5
C 12 H 14 NO 7:
C 12 H 14 N 1 O 7
pa Chrg -1
m/z

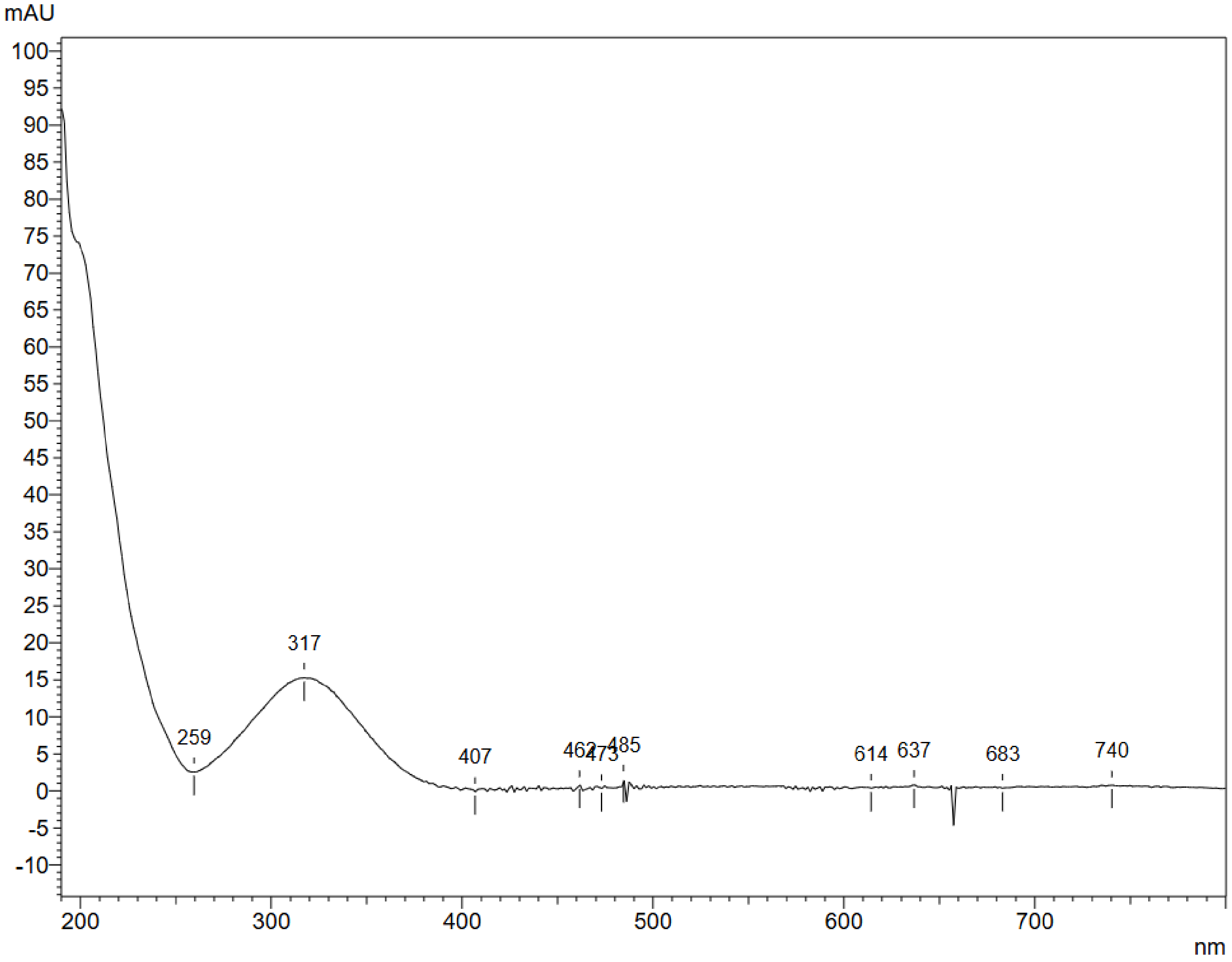
mAU
100
95
90
85
80
75
70
65
60
55
50
45
40
35
30
25
20
15
10
5
0
-5
-10
317
259
407
462
473
485
614
637
683
740
200
300
400
500
600
700
nm

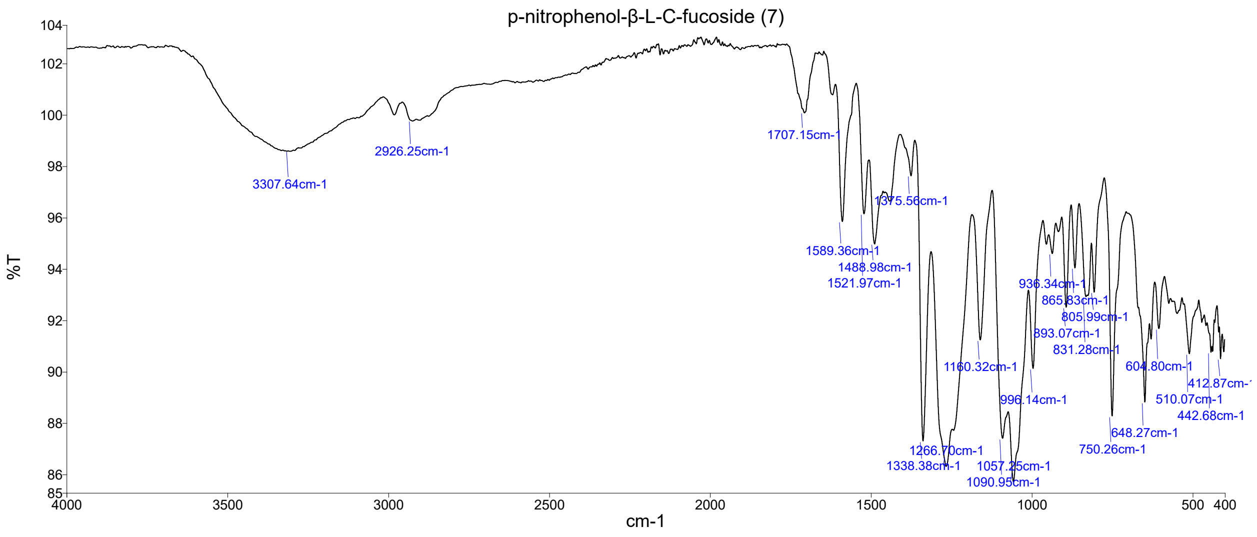
p-nitrophenol-β-L-C-fucoside (7)
%T
104
102
100
98
96
94
92
90
88
86
85
3307.64cm-1
2926.25cm-1
1707.15cm-1
1375.56cm-1
1589.36cm-1
1488.98cm-1
1521.97cm-1
936.34cm-1
865.83cm-1
805.99cm-1
893.07cm-1
831.28cm-1
1160.32cm-1
604.80cm-1
996.14cm-1
510.07cm-1
442.68cm-1
648.27cm-1
750.26cm-1
1266.70cm-1
1338.38cm-1
1057.25cm-1
1090.95cm-1
4000
3500
3000
2500
2000
1500
1000
500
400
cm-1

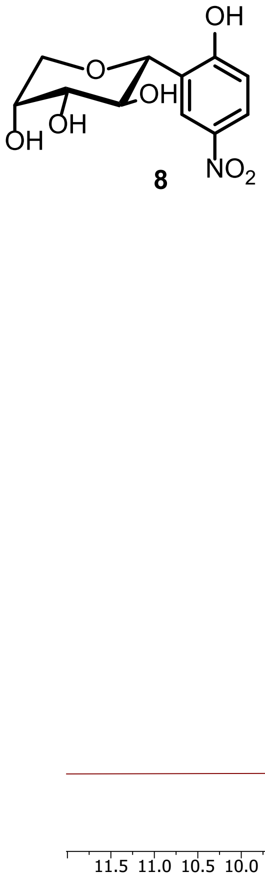
OH
O
OH
OH
OH
NO2

8

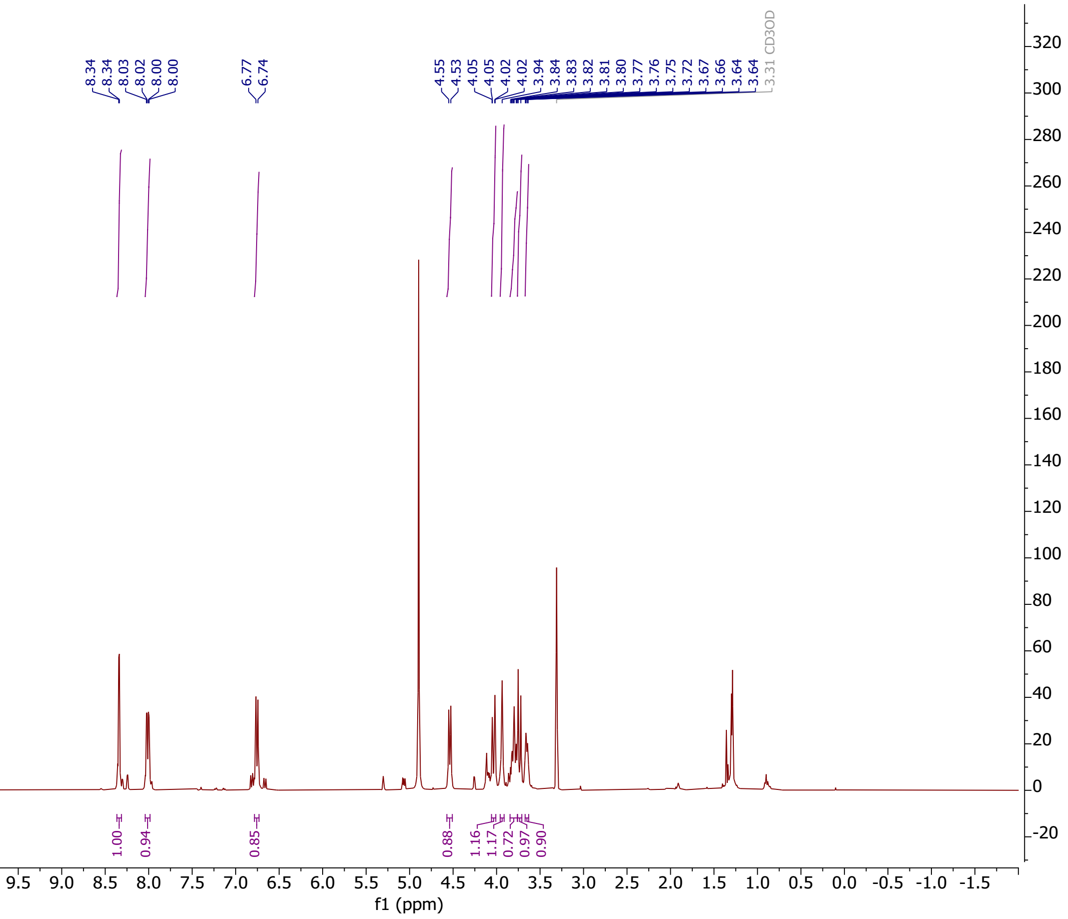
8.34
8.34
8.03
8.02
8.00
8.00
6.77
6.74
4.55
4.53
4.05
4.05
4.02
4.02
3.94
3.84
3.83
3.82
3.81
3.80
3.77
3.76
3.75
3.72
3.67
3.66
3.64
3.64
3.31 CD3OD
1.00
0.94
0.85
0.88
1.16
1.17
0.72
0.97
0.90
f1 (ppm)

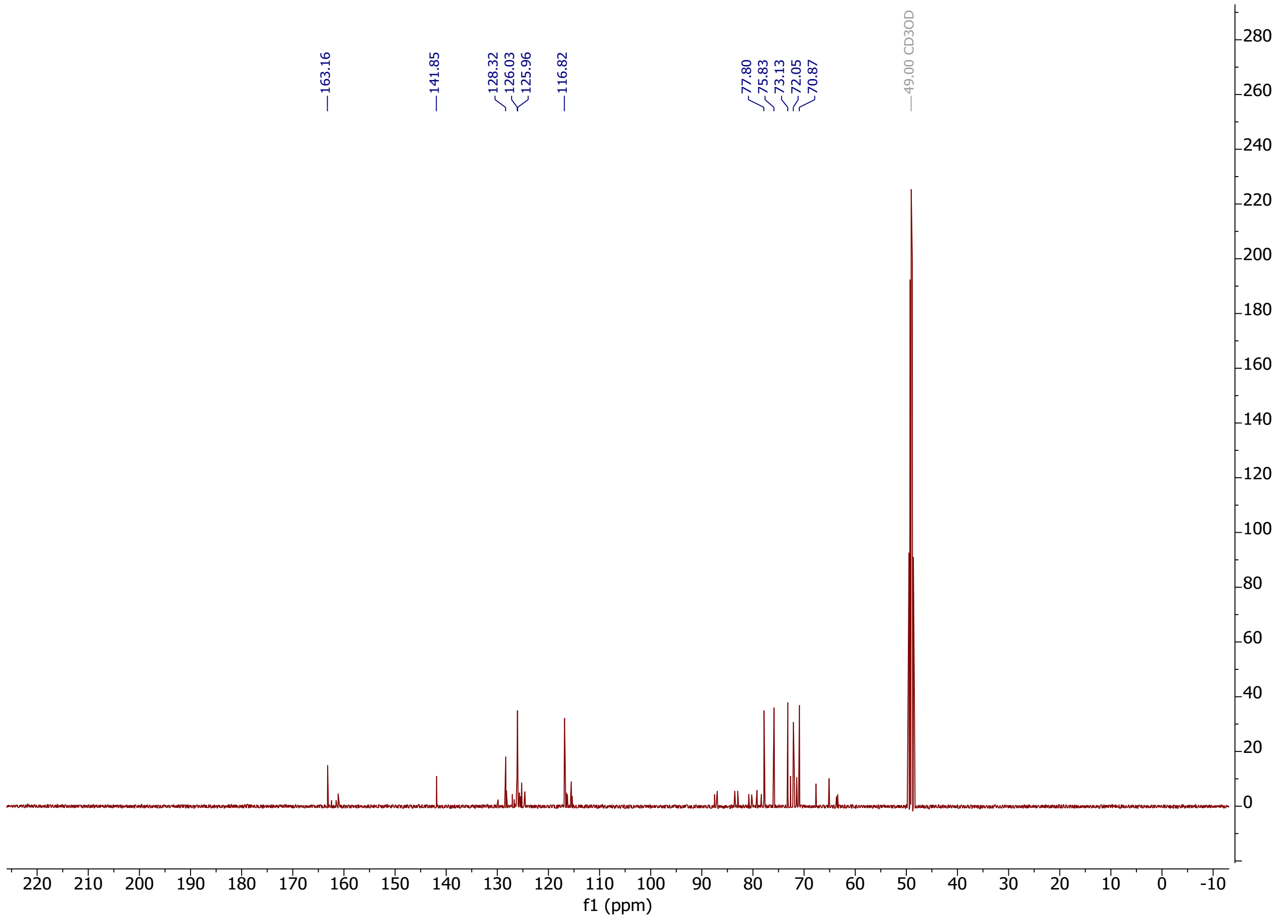
163.16
141.85
128.32
126.03
125.96
116.82
77.80
75.83
73.13
72.05
70.87
49.00 CD3OD
f1 (ppm)

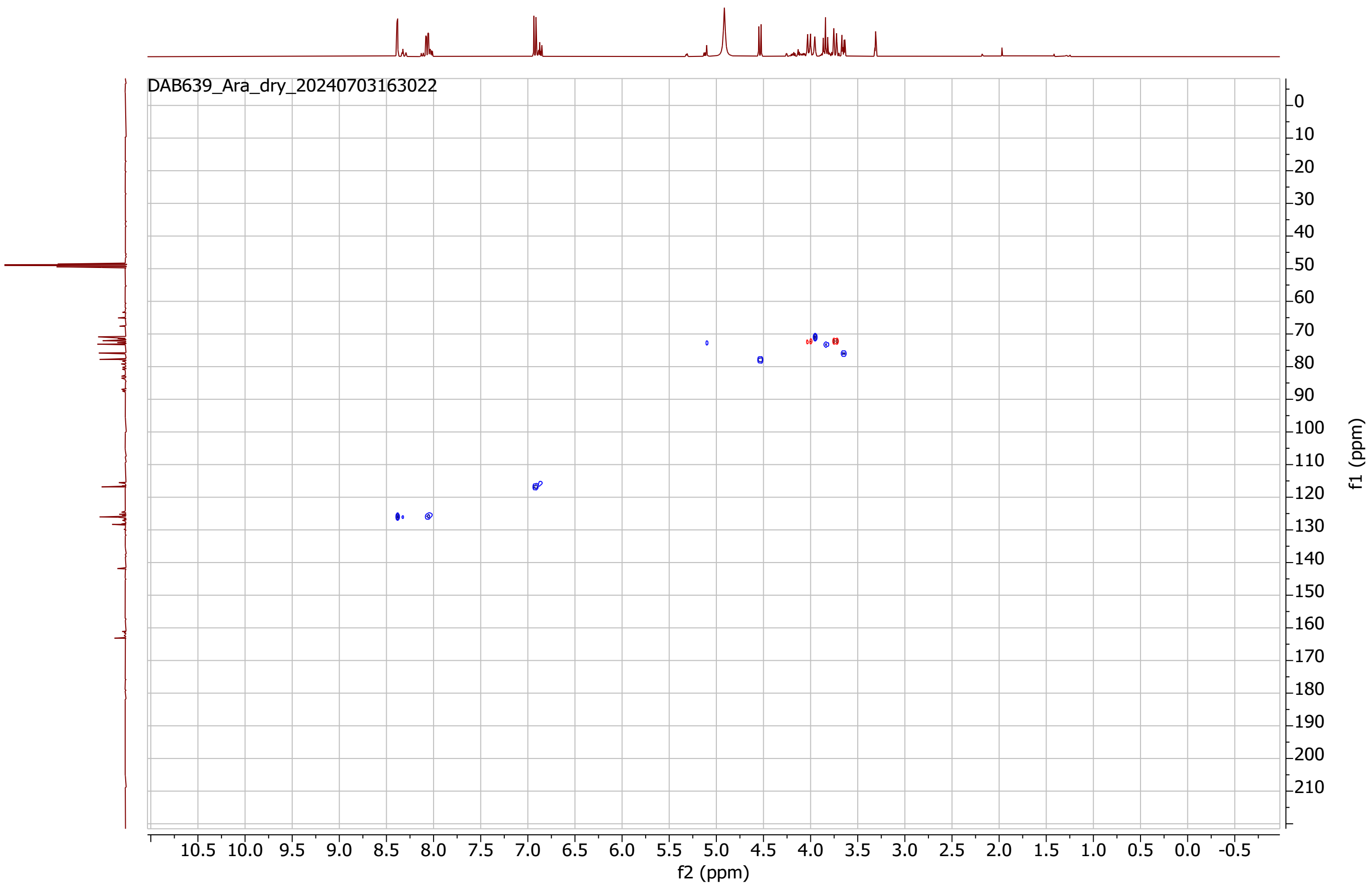
DAB639_Ara_dry_20240703163022
f2 (ppm)
f1 (ppm)

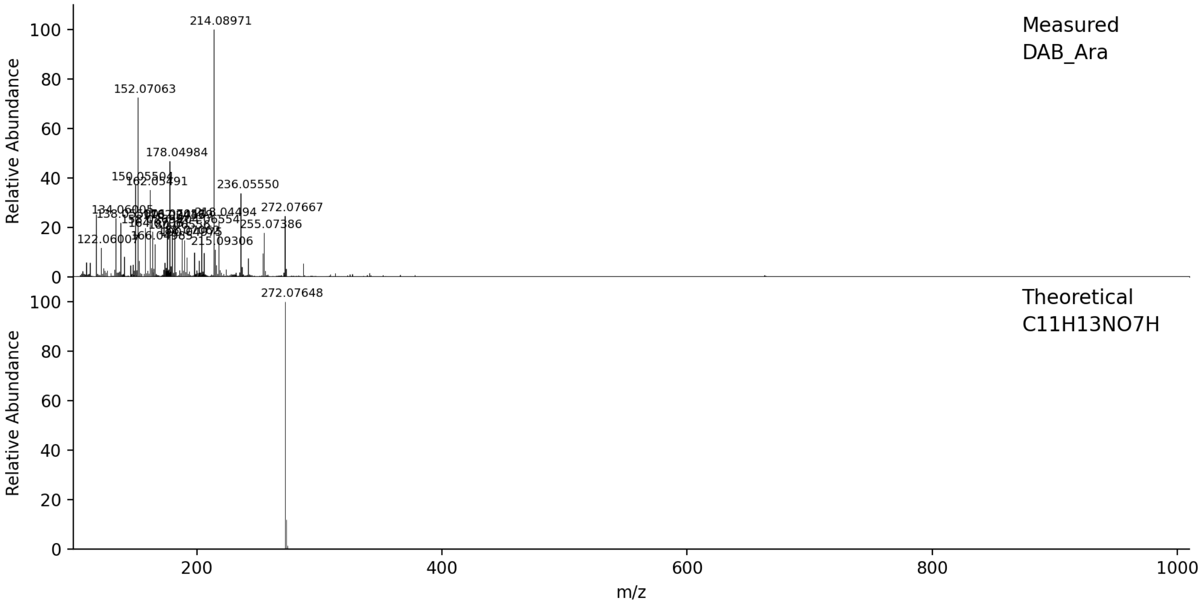
Measured
DAB_Ara
214.08971
152.07063
178.04984
236.05550
272.07667
255.07386
215.09306
122.0600
Theoretical
C11H13NO7H
272.07648
Relative Abundance
m/z

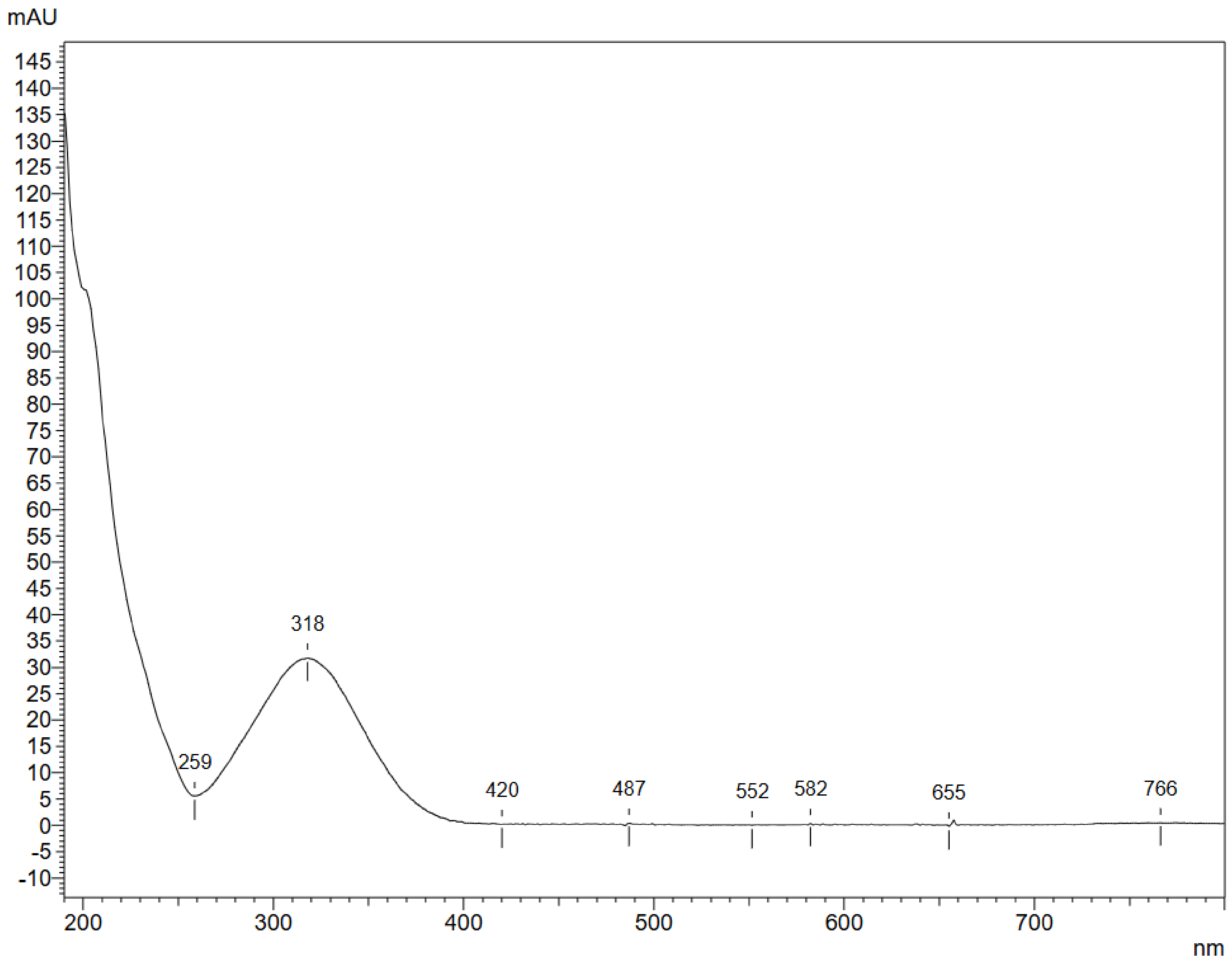
mAU
318
259
420
487
552
582
655
766
nm

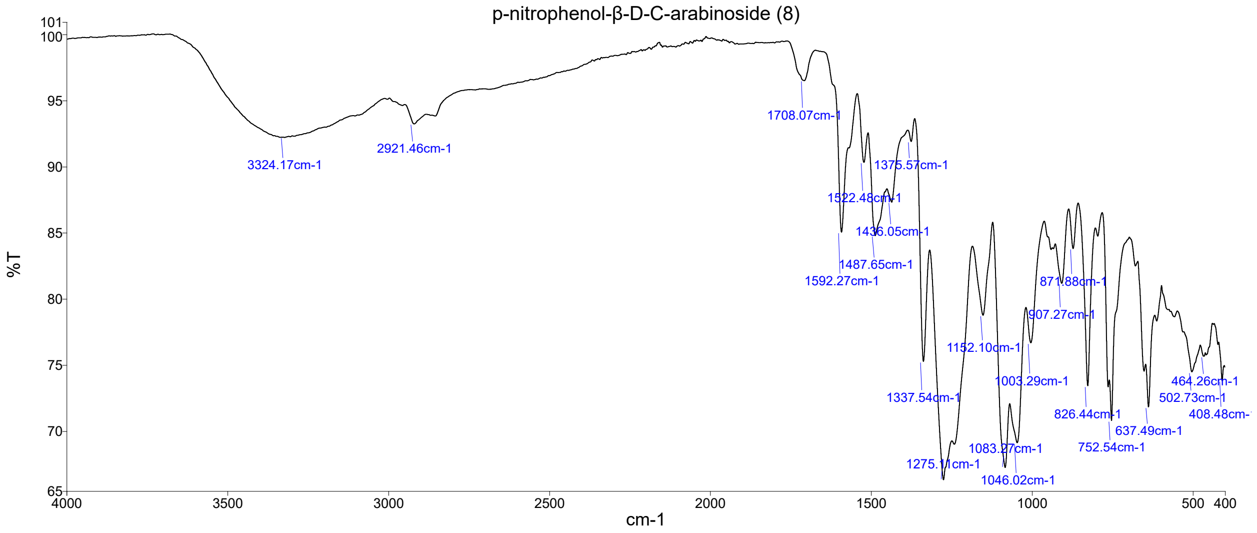
p-nitrophenol-β-D-C-arabinoside (8)
%T
cm-1
3324.17cm-1
2921.46cm-1
1708.07cm-1
1592.27cm-1
1522.48cm-1
1487.65cm-1
1436.05cm-1
1375.57cm-1
1337.54cm-1
1275.11cm-1
1152.10cm-1
1083.27cm-1
1046.02cm-1
1003.29cm-1
907.27cm-1
871.88cm-1
826.44cm-1
752.54cm-1
637.49cm-1
502.73cm-1
464.26cm-1

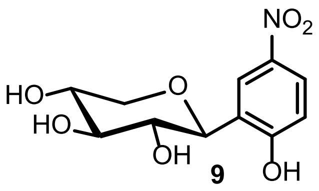
NO2
HO
HO
O
OH
OH

9

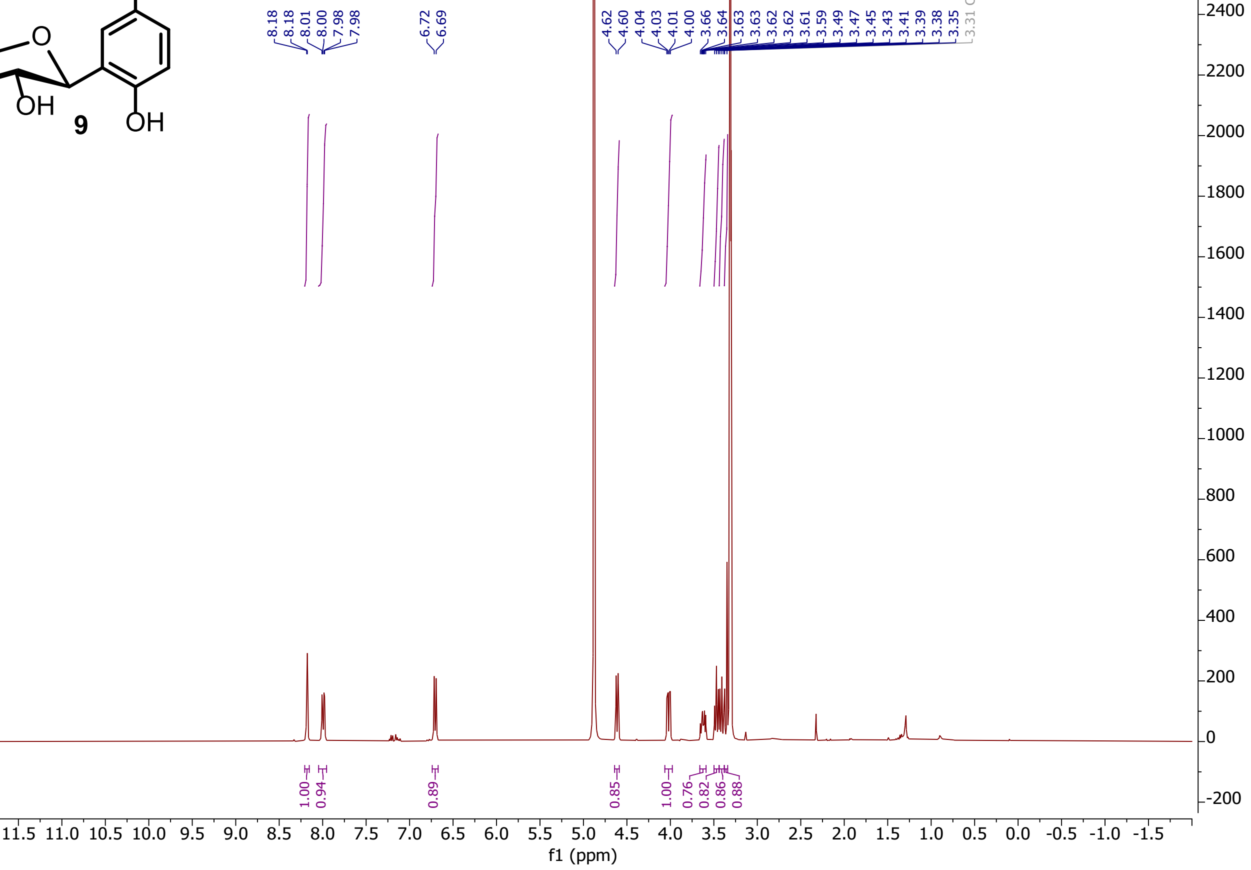
8.18
8.18
8.01
8.00
7.98
7.98
6.72
6.69
4.62
4.60
4.04
4.03
4.01
4.00
3.66
3.64
3.63
3.63
3.62
3.62
3.61
3.59
3.49
3.47
3.45
3.43
3.41
3.39
3.38
3.35
3.31 CD3OD
1.00
0.94
0.89
0.85
1.00
0.76
0.82
0.86
0.88
f1 (ppm)

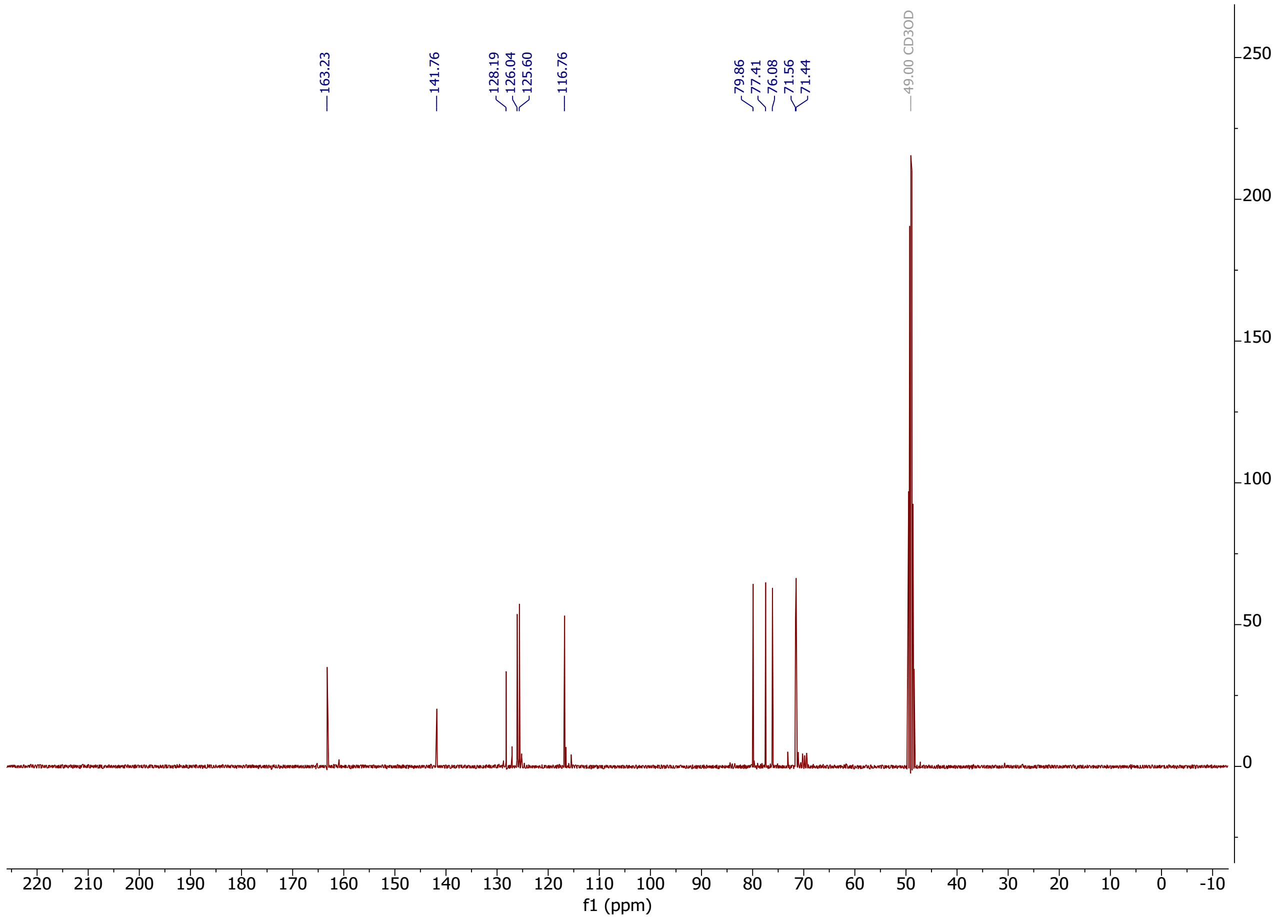
163.23
141.76
128.19
126.04
125.60
116.76
79.86
77.41
76.08
71.56
71.44
49.00 CD3OD
f1 (ppm)

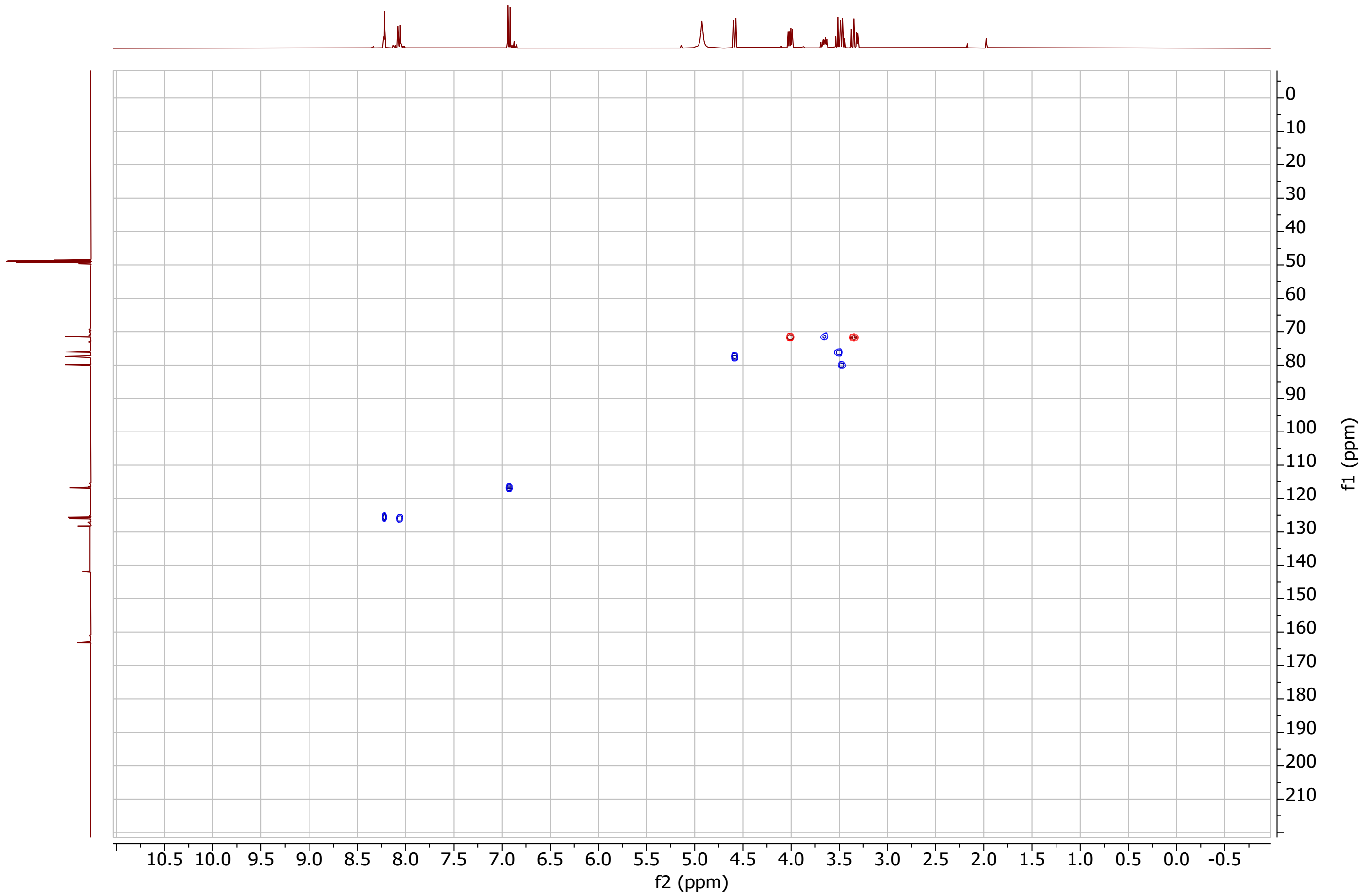
f1 (ppm)
f2 (ppm)

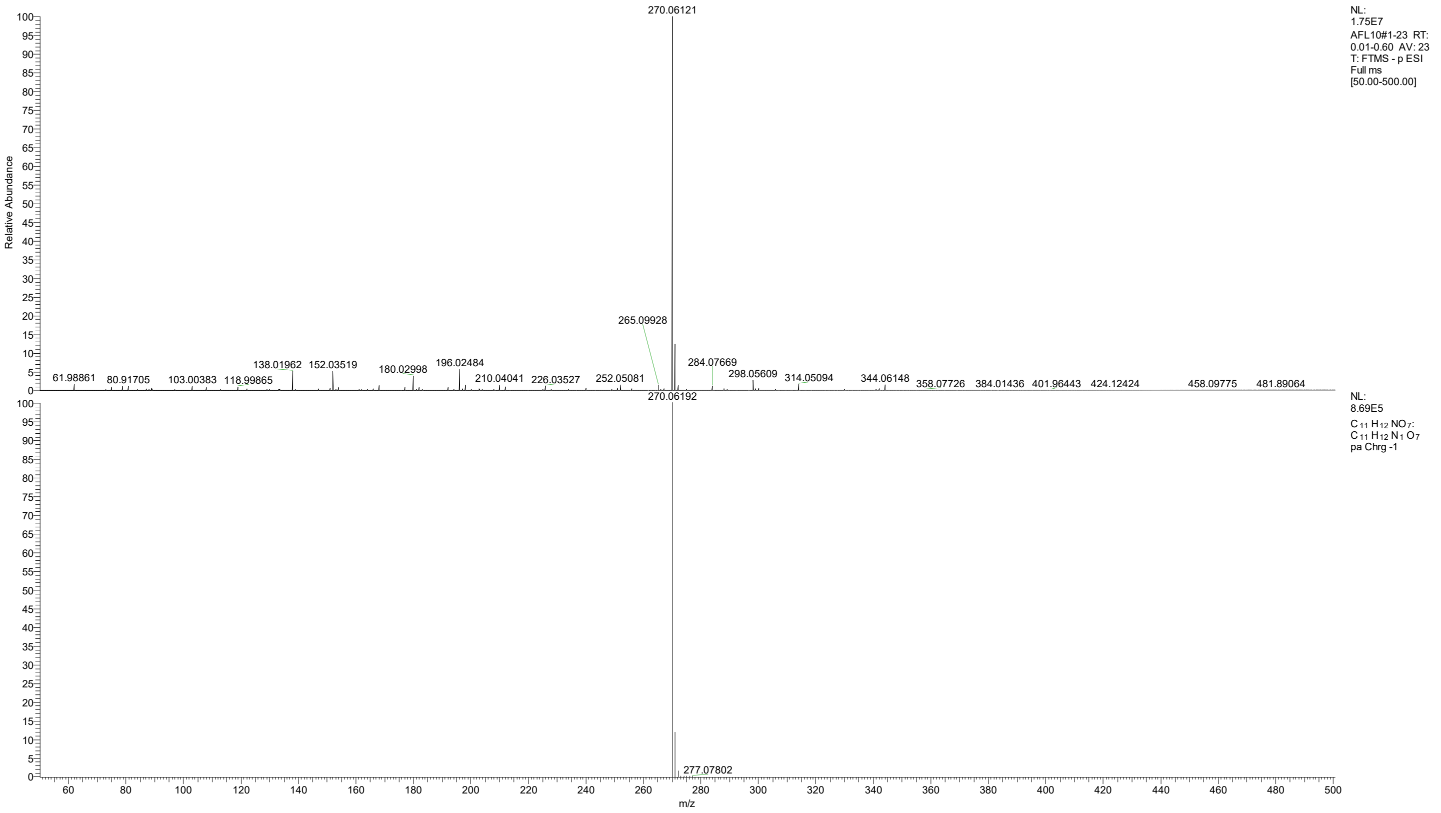
270.06121
NL:
1.75E7
AFL10#1-23 RT:
0.01-0.60 AV: 23
T: FTMS - p ESI
Full ms
[50.00-500.00]
265.09928
284.07669
298.05609
314.05094
344.06148
138.01962
152.03519
180.02998
196.02484
210.04041
226.03527
252.05081
61.98861
80.91705
103.00383
118.99865
358.07726
384.01436
401.96443
424.12424
458.09775
481.89064
270.06192
NL:
8.69E5
C 11 H 12 NO 7:
C 11 H 12 N 1 O 7
pa Chrg -1
277.07802
Relative Abundance
m/z

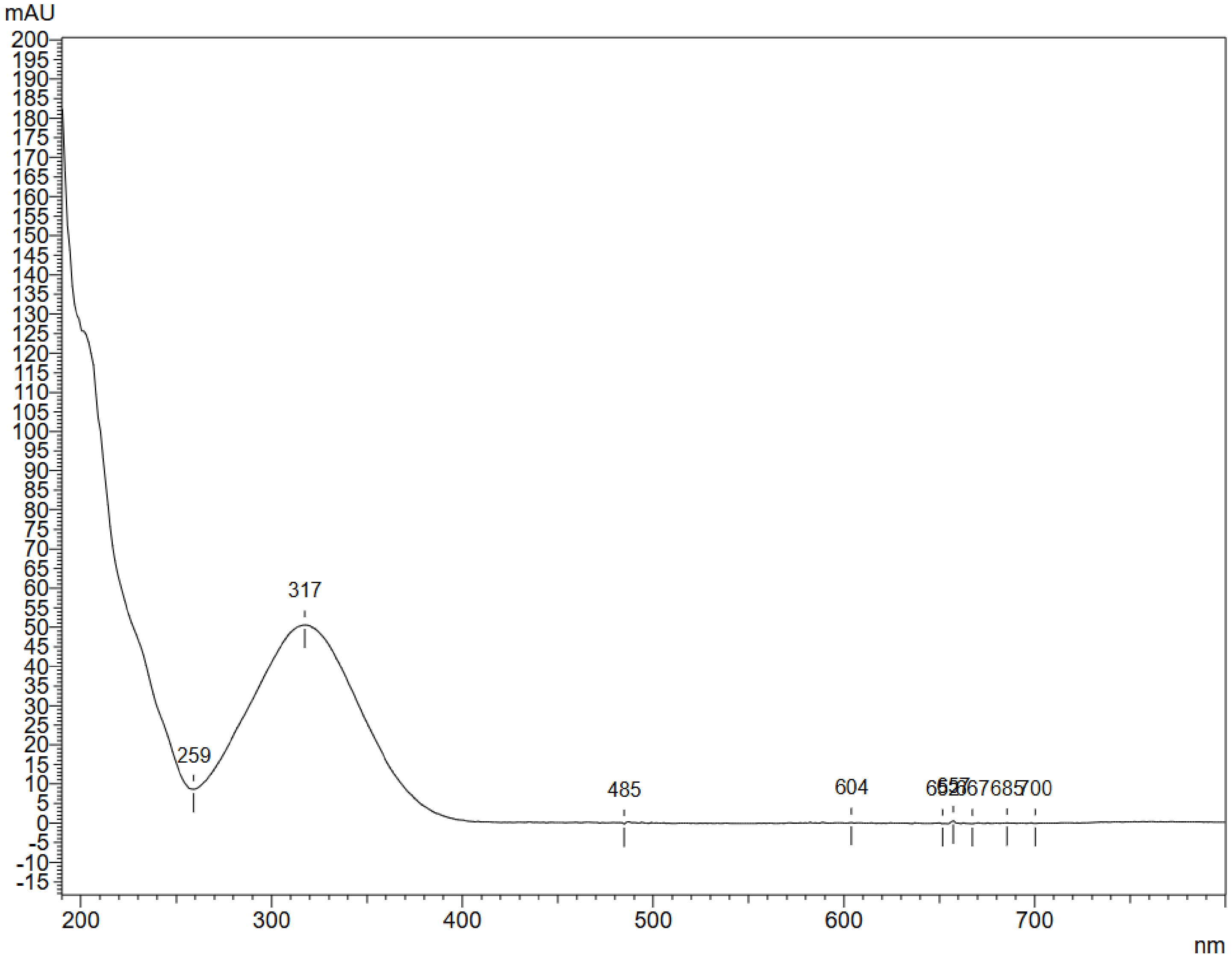
mAU
nm
317
259
485
604
700

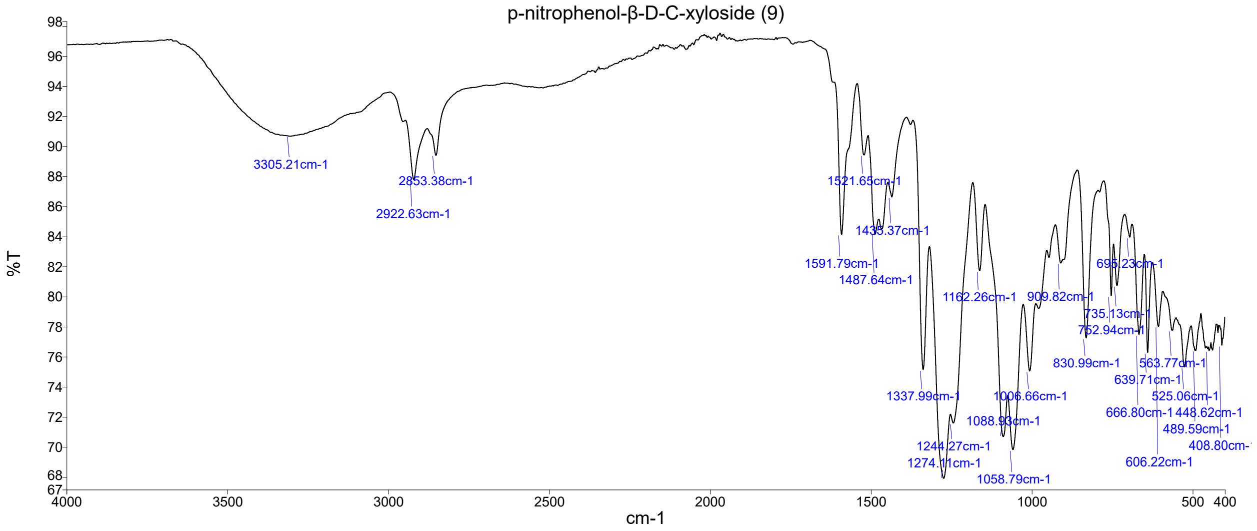
p-nitrophenol-β-D-C-xyloside (9)
%T
cm-1
3305.21cm-1
2853.38cm-1
2922.63cm-1
1521.65cm-1
1435.37cm-1
1591.79cm-1
1487.64cm-1
1162.26cm-1
909.82cm-1
1337.99cm-1
1006.66cm-1
1088.93cm-1
1244.27cm-1
1274.11cm-1
1058.79cm-1
735.13cm-1
752.94cm-1
830.99cm-1
563.77cm-1
639.71cm-1
525.06cm-1
666.80cm-1
448.62cm-1
489.59cm-1
606.22cm-1

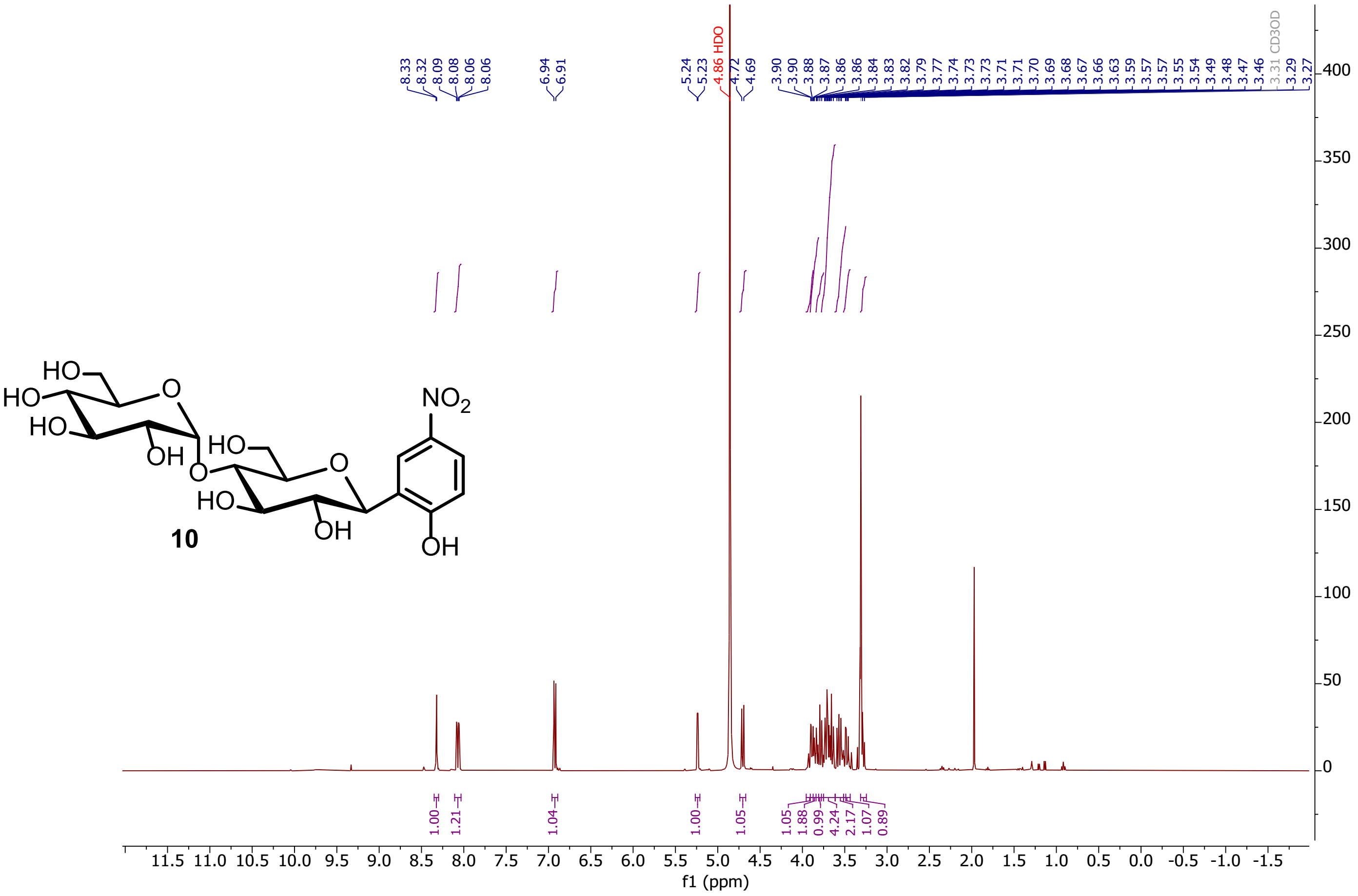
8.33 8.32 8.09 8.08 8.06 8.06
6.94 6.91
5.24 5.23
4.86 HDO
4.72 4.69
3.90 3.90 3.88 3.87 3.86 3.86 3.84 3.83 3.82 3.79 3.77 3.74 3.73 3.73 3.71 3.71 3.70 3.69 3.68 3.67 3.66 3.63 3.59 3.57 3.57 3.55 3.54 3.49 3.48 3.47 3.46
3.31 CD3OD
3.29 3.27
1.00 1.21 1.04 1.00 1.05 1.05 1.88 0.99 4.24 2.17 1.07 0.89
HO HO HO O OH HO O O HO OH NO2 OH
10
f1 (ppm)

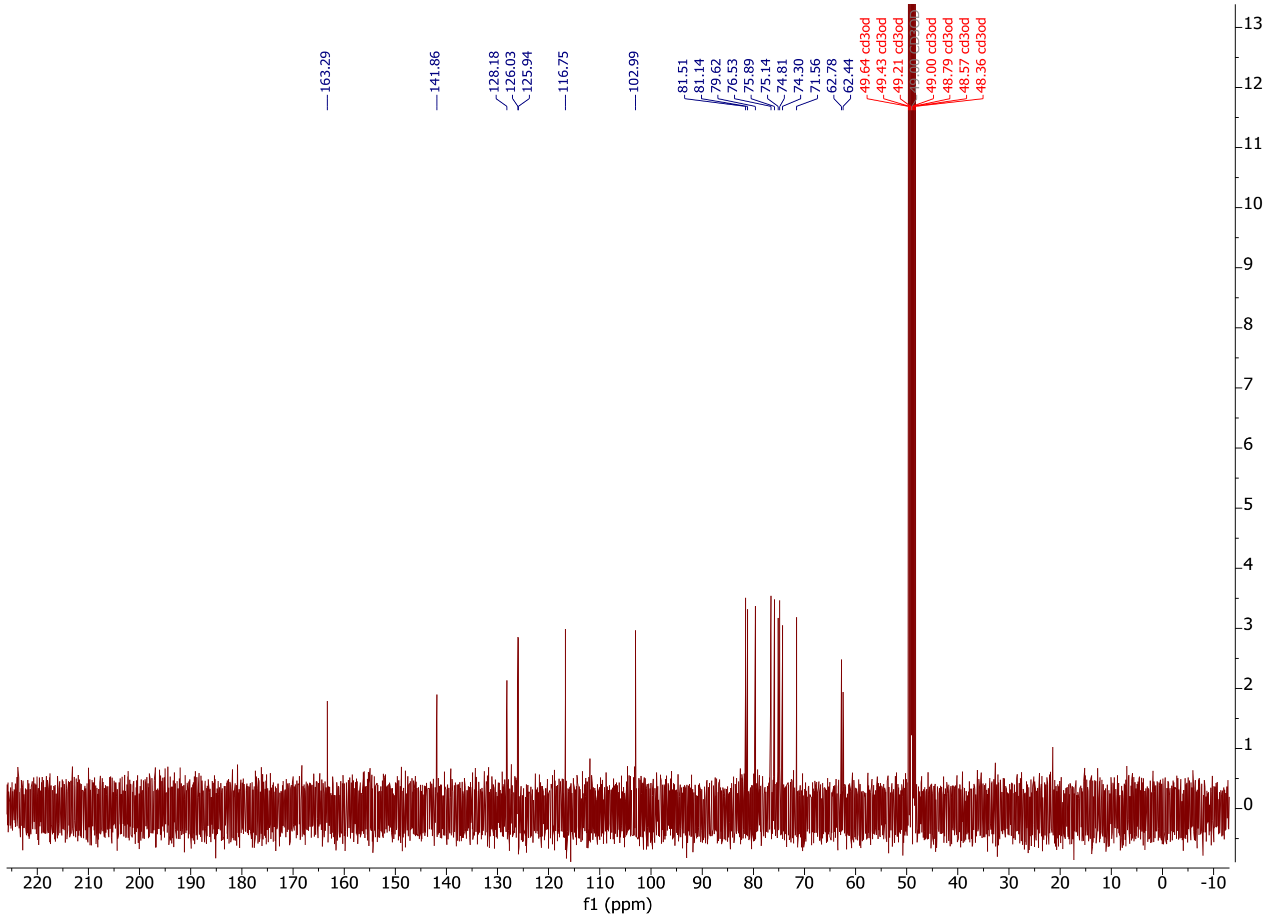
163.29
141.86
128.18 126.03 125.94
116.75
102.99
81.51 81.14 79.62 76.53 75.89 75.14 74.81 74.30 71.56 62.78 62.44
49.64 cd3od 49.43 cd3od 49.21 cd3od 49.00 CD3OD 49.00 cd3od 48.79 cd3od 48.57 cd3od 48.36 cd3od
f1 (ppm)

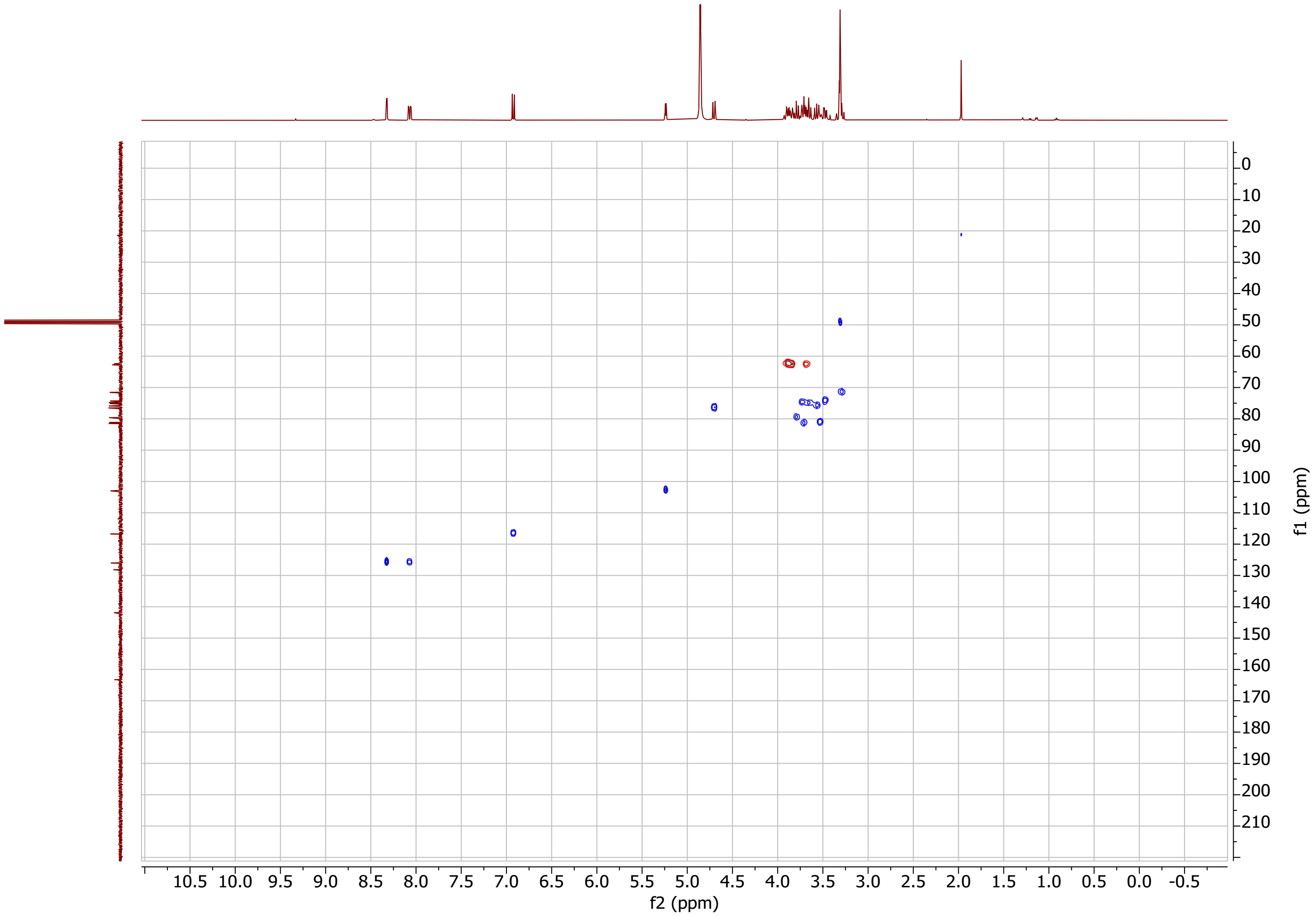

f2 (ppm)
f1 (ppm)

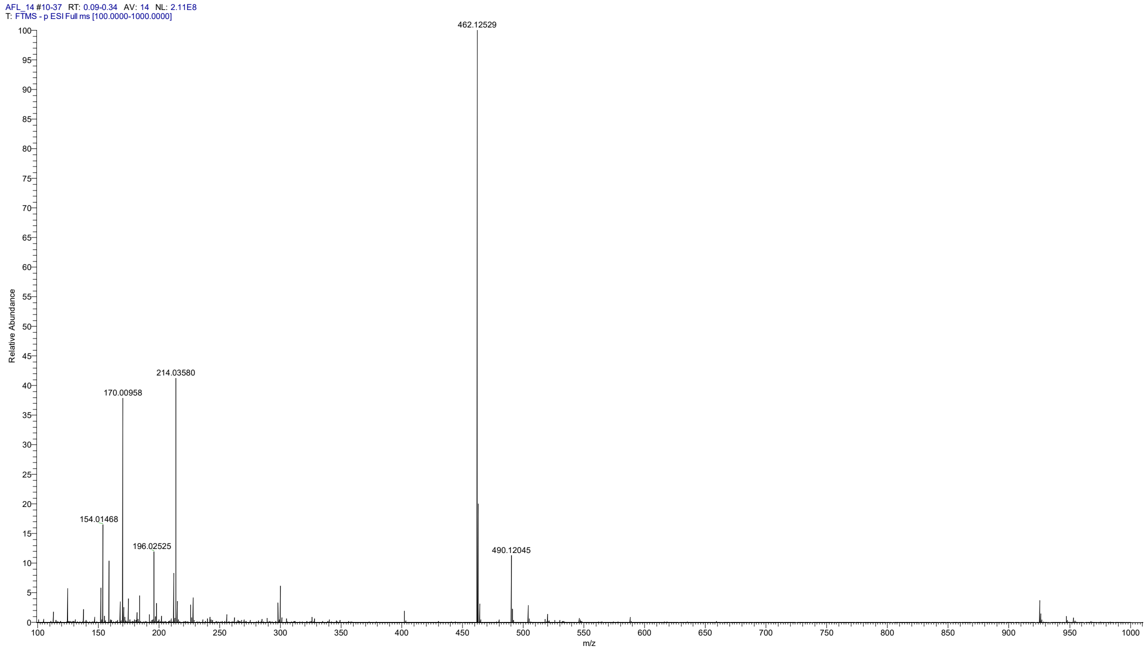

AFL_14 #10-37 RT: 0.09-0.34 AV: 14 NL: 2.11E8
T: FTMS - p ESI Full ms [100.0000-1000.0000]
462.12529
214.03580
170.00958
154.01468
196.02525
490.12045
Relative Abundance
m/z

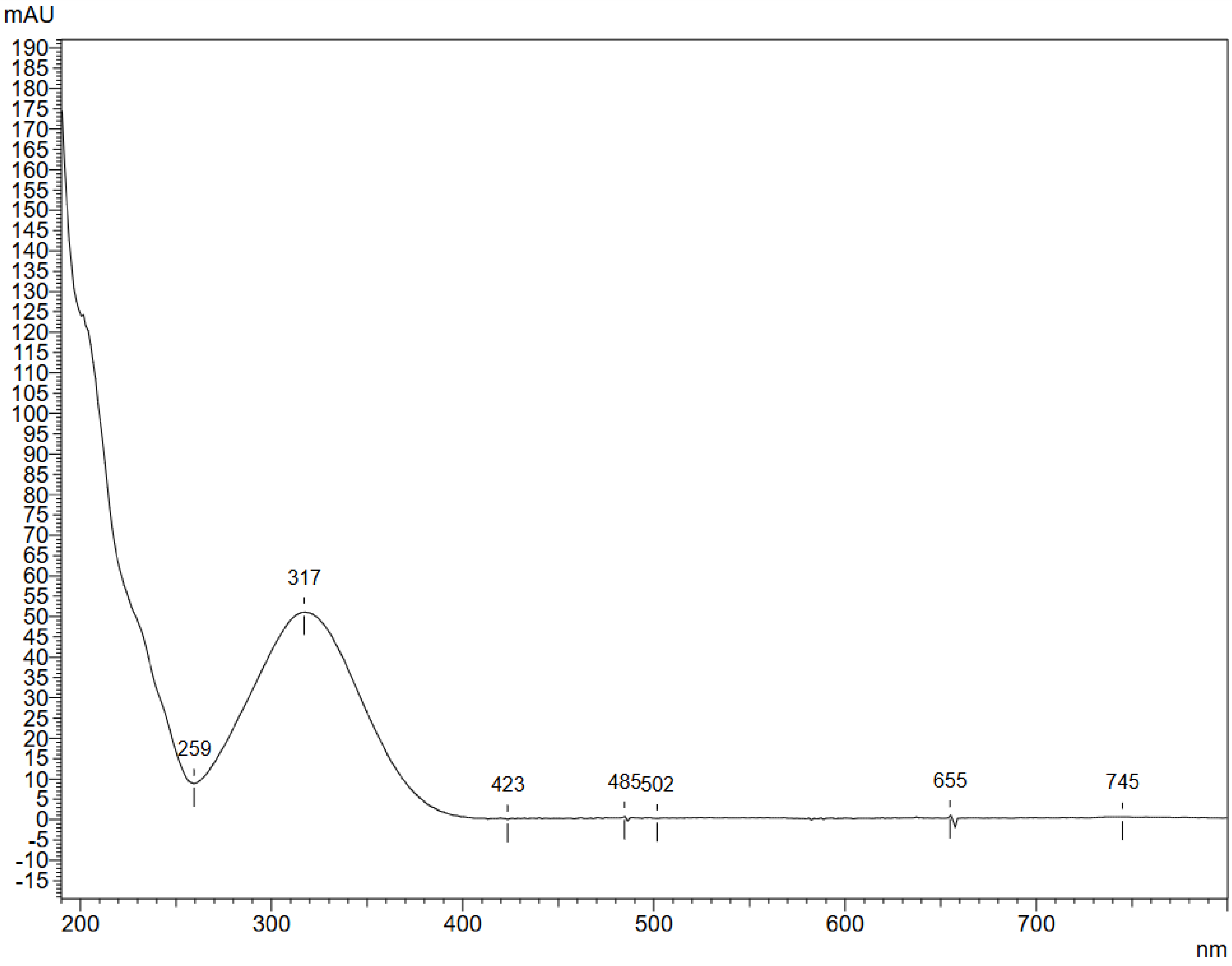
mAU
nm
317
259
423
485
502
655
745

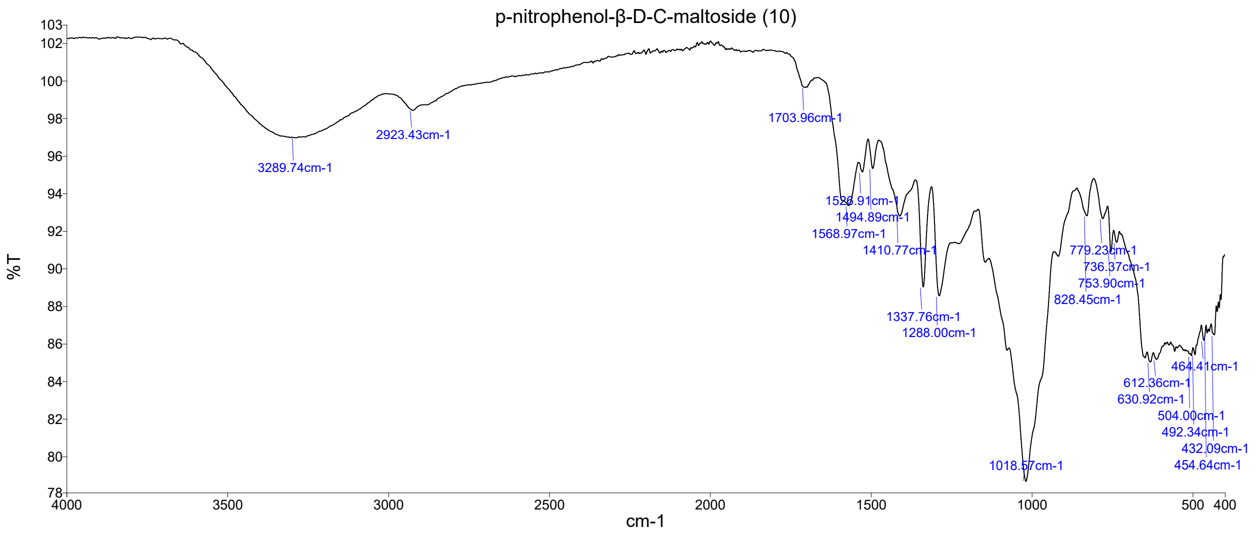
p-nitrophenol-β-D-C-maltoside (10)
%T
cm-1
3289.74cm-1
2923.43cm-1
1703.96cm-1
1526.91cm-1
1494.89cm-1
1568.97cm-1
1410.77cm-1
1337.76cm-1
1288.00cm-1
1018.57cm-1
779.23cm-1
736.37cm-1
753.90cm-1
828.45cm-1
464.41cm-1
612.36cm-1
630.92cm-1
504.00cm-1
492.34cm-1
432.09cm-1
454.64cm-1

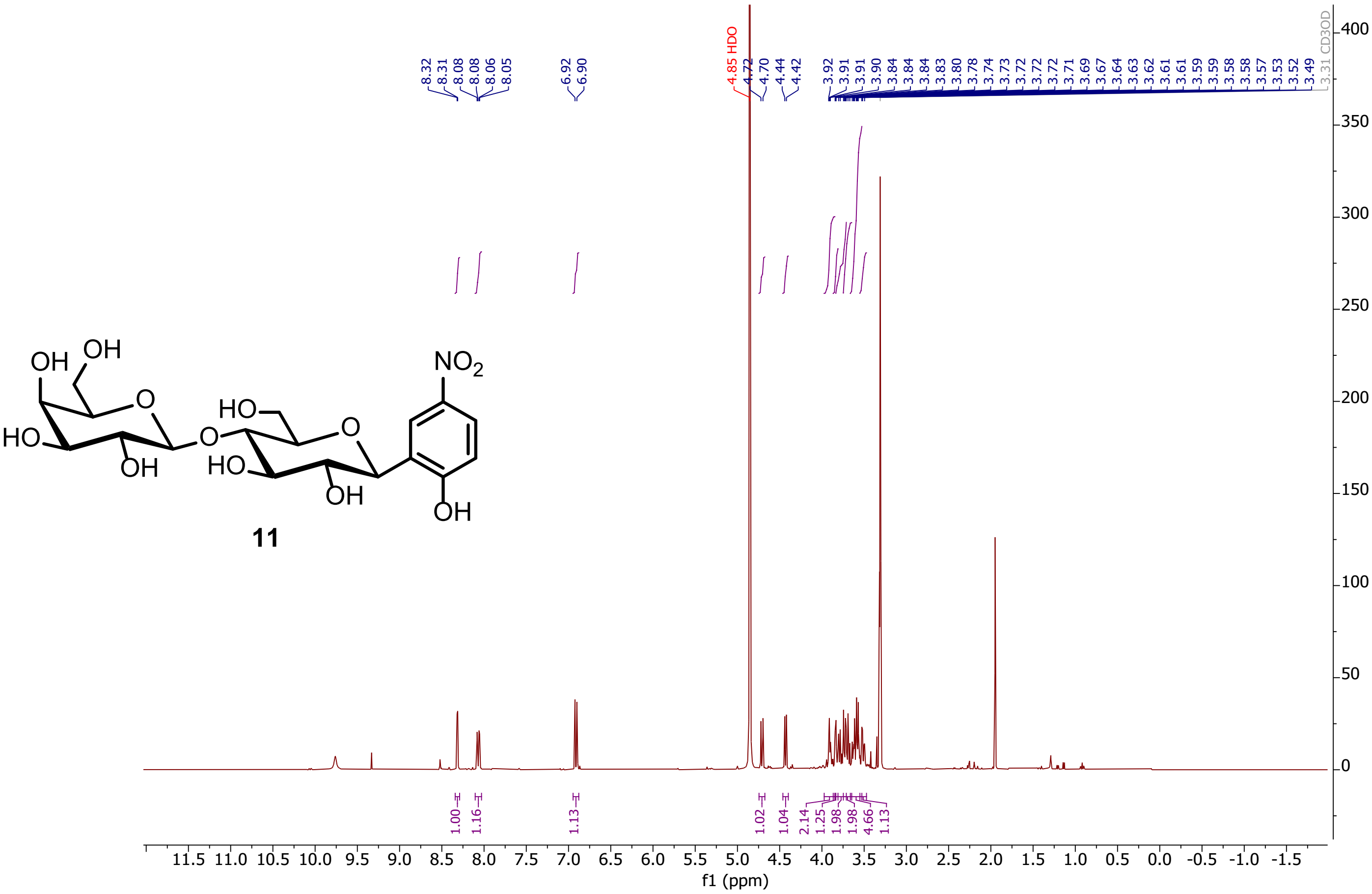

OH
OH
O
HO
O
HO
O
OH
HO
OH
NO2
OH
11
8.32
8.31
8.08
8.08
8.06
8.05
6.92
6.90
4.85 HDO
4.72
4.70
4.44
4.42
3.92
3.91
3.91
3.90
3.84
3.84
3.84
3.83
3.80
3.78
3.74
3.73
3.72
3.72
3.72
3.71
3.69
3.67
3.64
3.63
3.62
3.61
3.61
3.59
3.59
3.58
3.57
3.53
3.52
3.49
3.31 CD3OD
1.00
1.16
1.13
1.02
1.04
2.14
1.25
1.98
1.98
4.66
1.13
f1 (ppm)

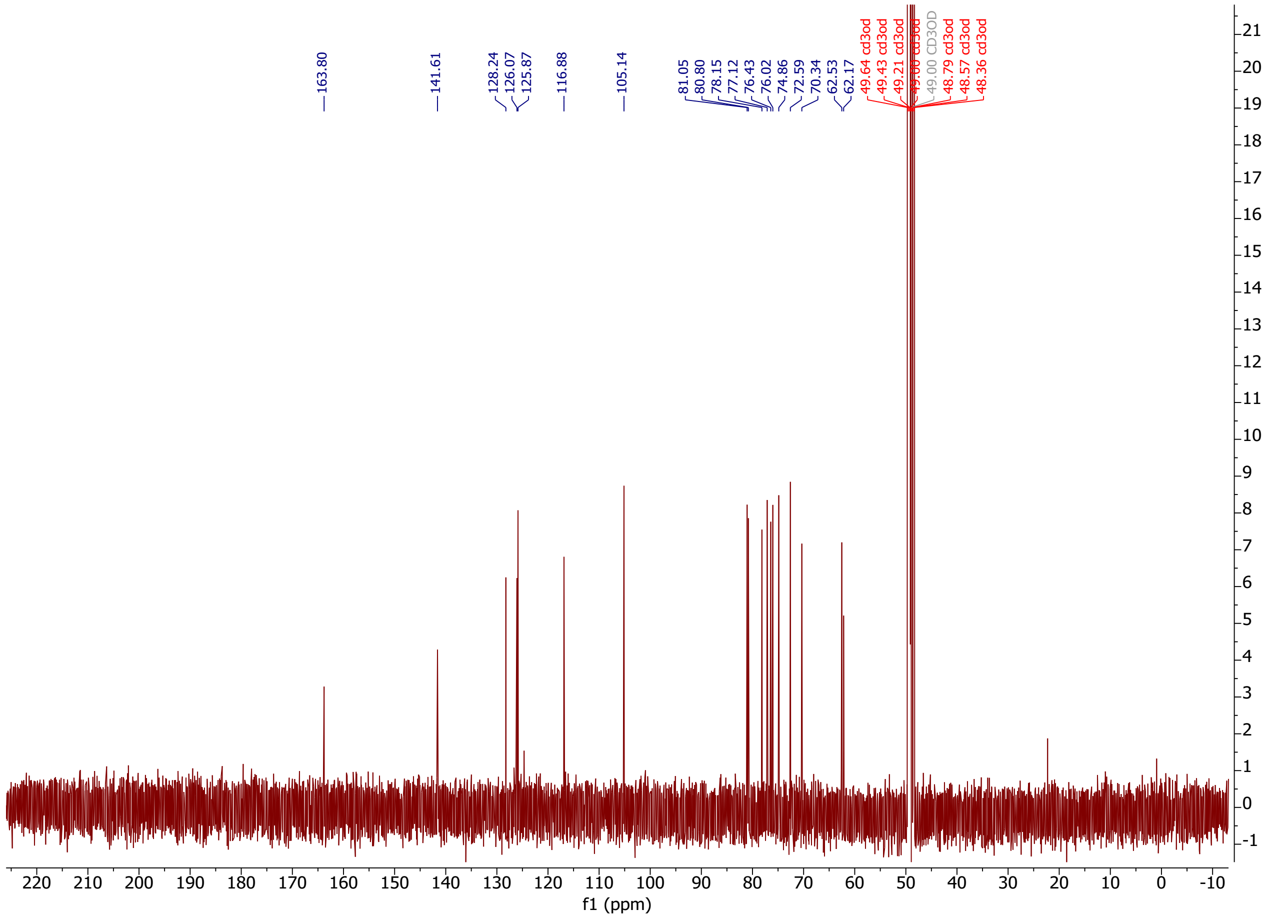

163.80
141.61
128.24
126.07
125.87
116.88
105.14
81.05
80.80
78.15
77.12
76.43
76.02
74.86
72.59
70.34
62.53
62.17
49.64 cd3od
49.43 cd3od
49.21 cd3od
49.00 cd3od
49.00 CD3OD
48.79 cd3od
48.57 cd3od
48.36 cd3od
f1 (ppm)

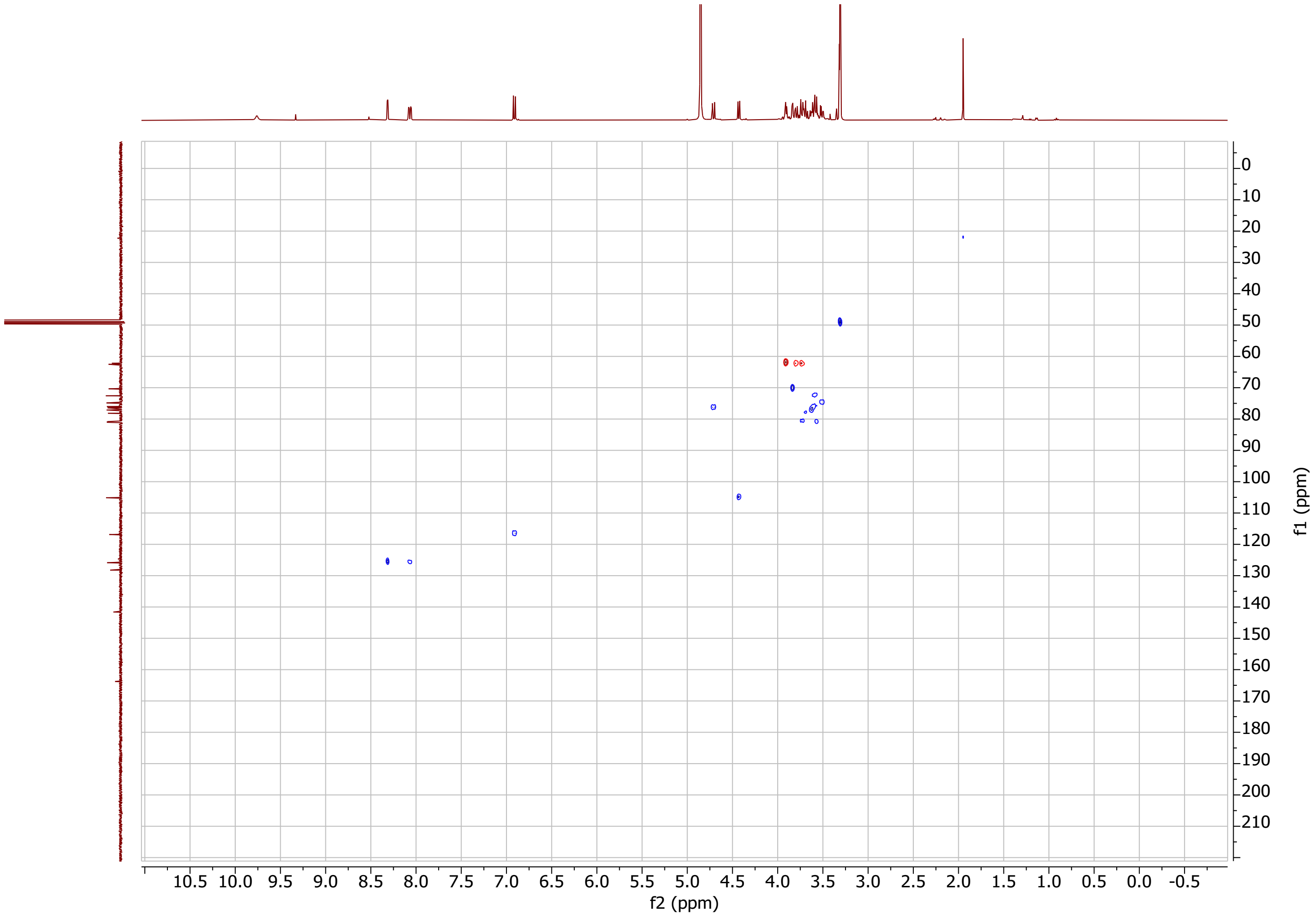
f2 (ppm)
f1 (ppm)

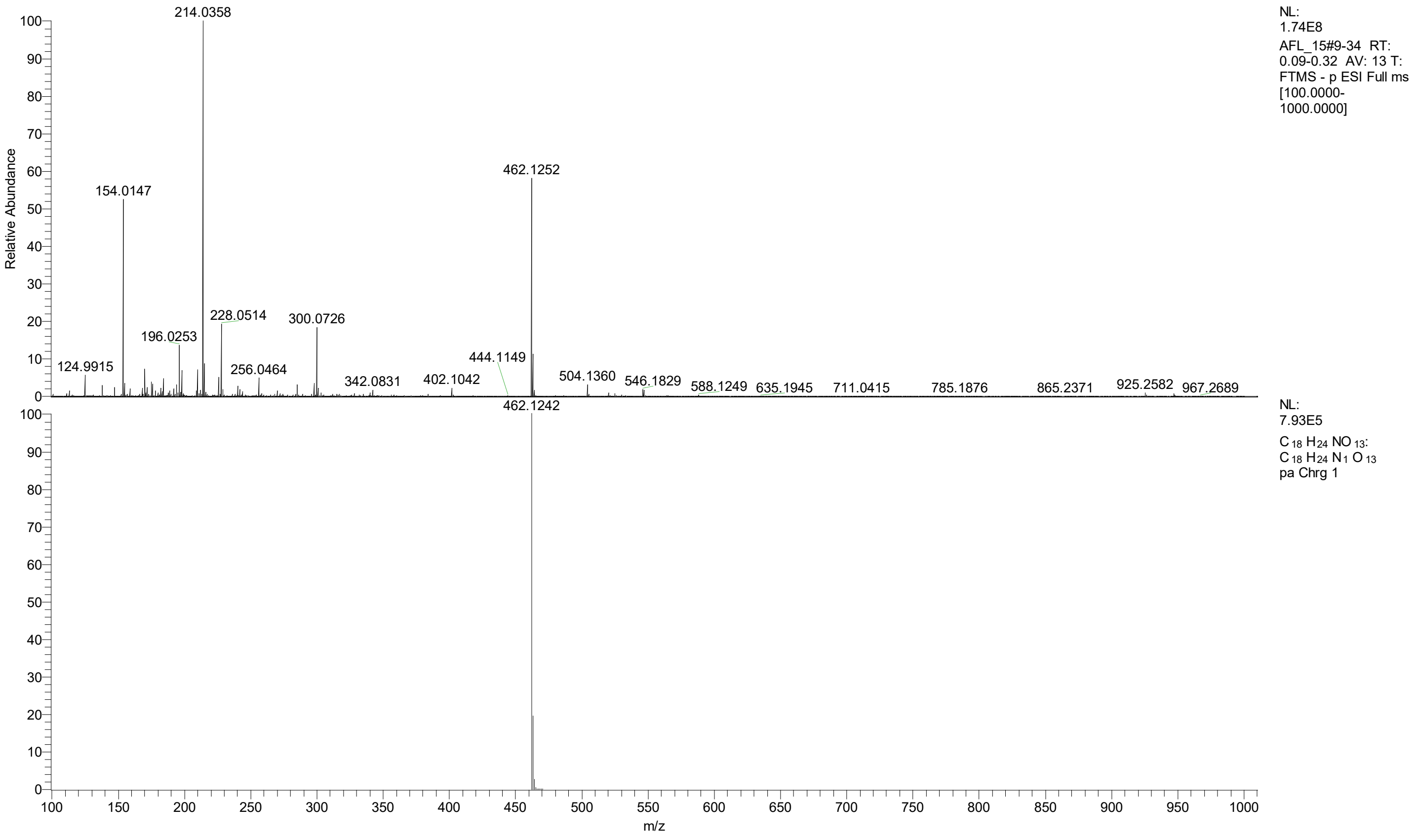
NL:
1.74E8
AFL_15#9-34 RT:
0.09-0.32 AV: 13 T:
FTMS - p ESI Full ms
[100.0000-
1000.0000]
214.0358
462.1252
154.0147
228.0514
300.0726
196.0253
124.9915
256.0464
444.1149
504.1360
546.1829
342.0831
402.1042
588.1249
635.1945
711.0415
785.1876
865.2371
925.2582
967.2689
462.1242
NL:
7.93E5
C 18 H24 NO 13:
C 18 H24 N1 O 13
pa Chrg 1
Relative Abundance
m/z

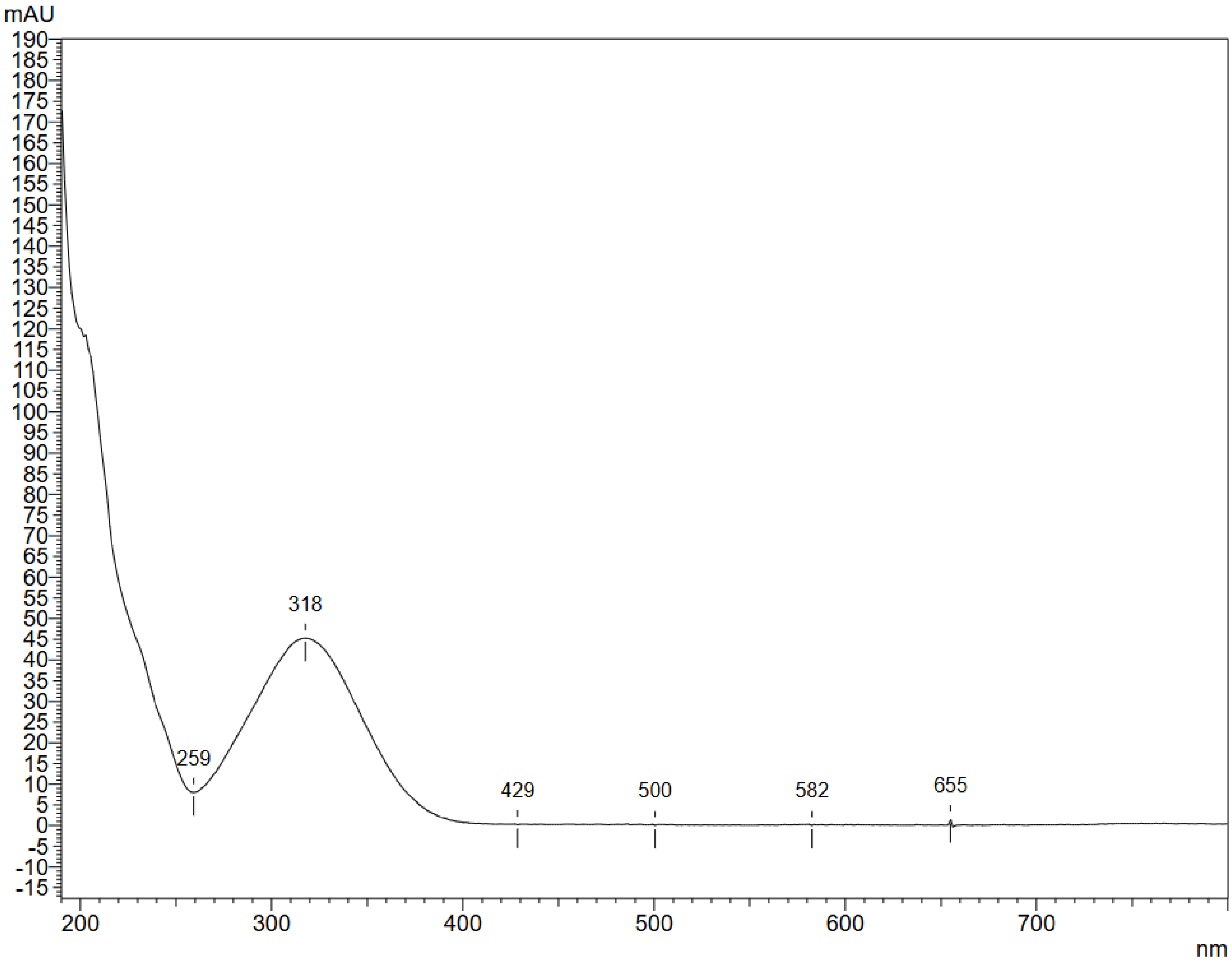
mAU
318
259
429
500
582
655
nm

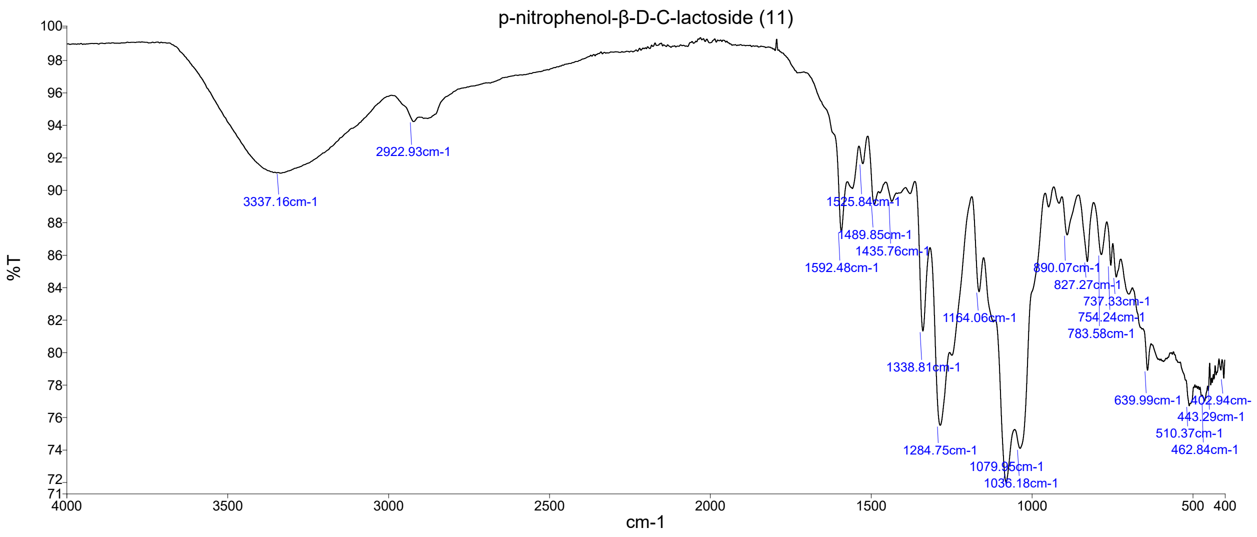
p-nitrophenol-β-D-C-lactoside (11)
%T
cm-1
3337.16cm-1
2922.93cm-1
1525.84cm-1
1489.85cm-1
1435.76cm-1
1592.48cm-1
1338.81cm-1
1284.75cm-1
1164.06cm-1
1079.95cm-1
1036.18cm-1
890.07cm-1
827.27cm-1
737.33cm-1
754.24cm-1
783.58cm-1
639.99cm-1
402.94cm-
443.29cm-1
510.37cm-1
462.84cm-1

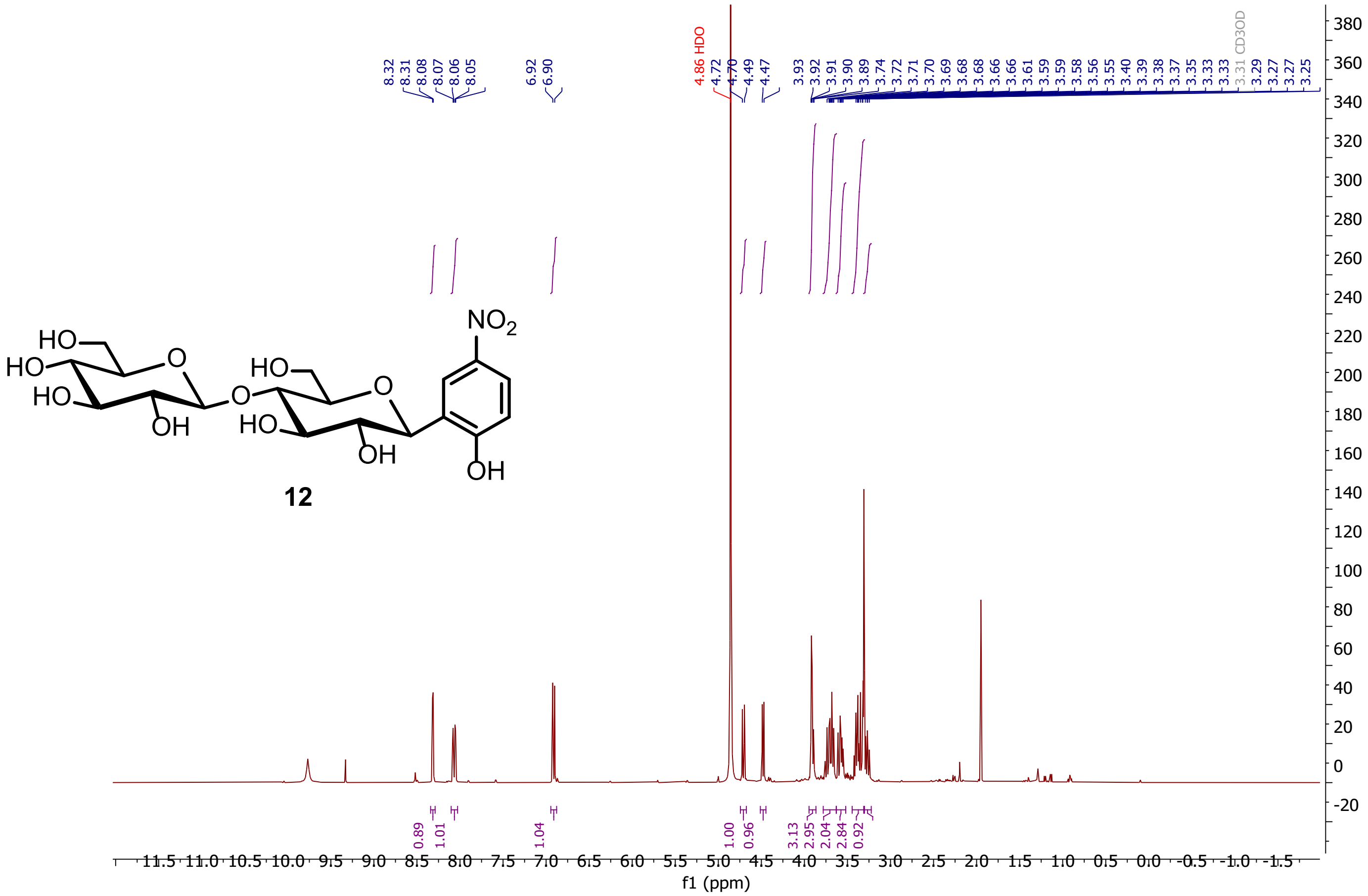

12

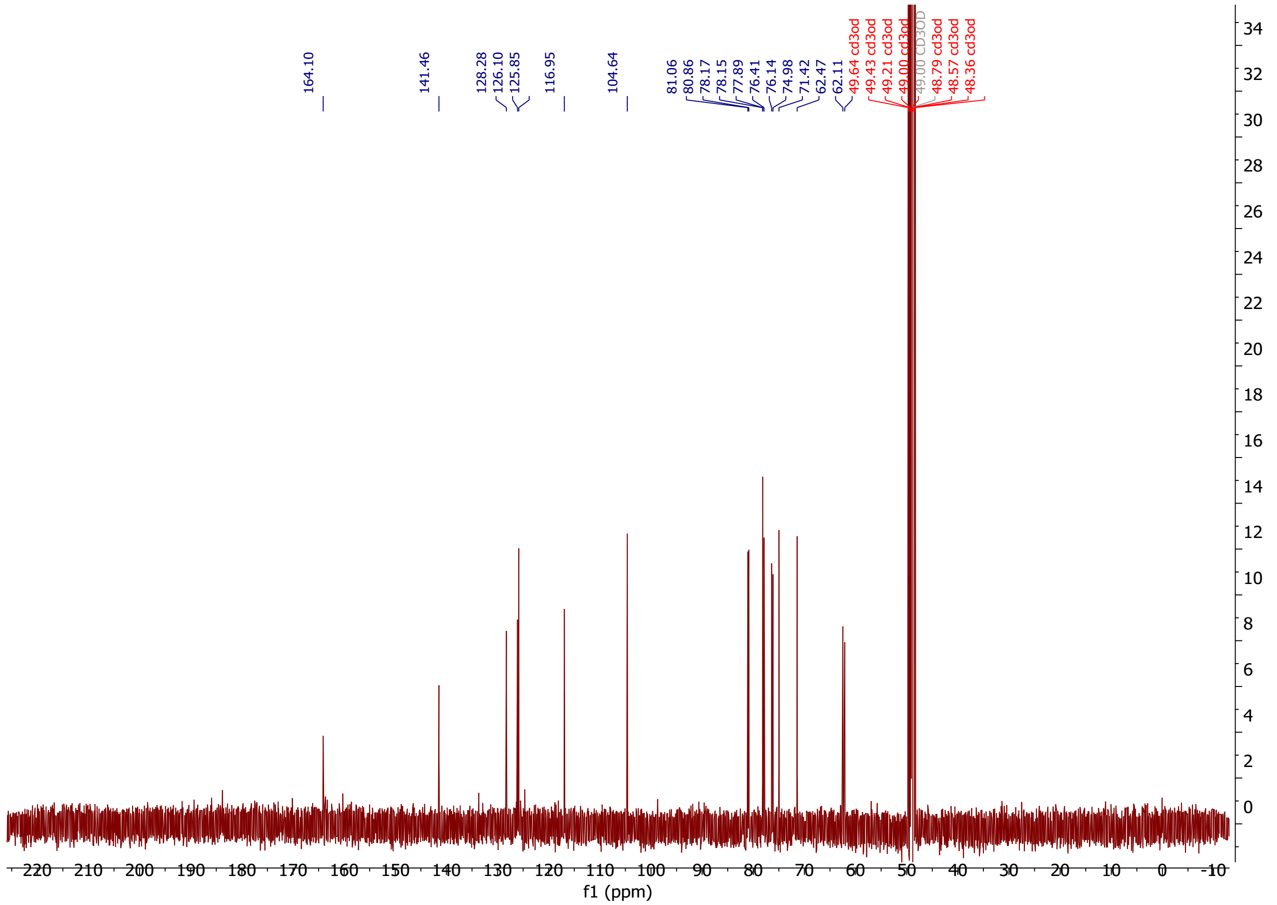

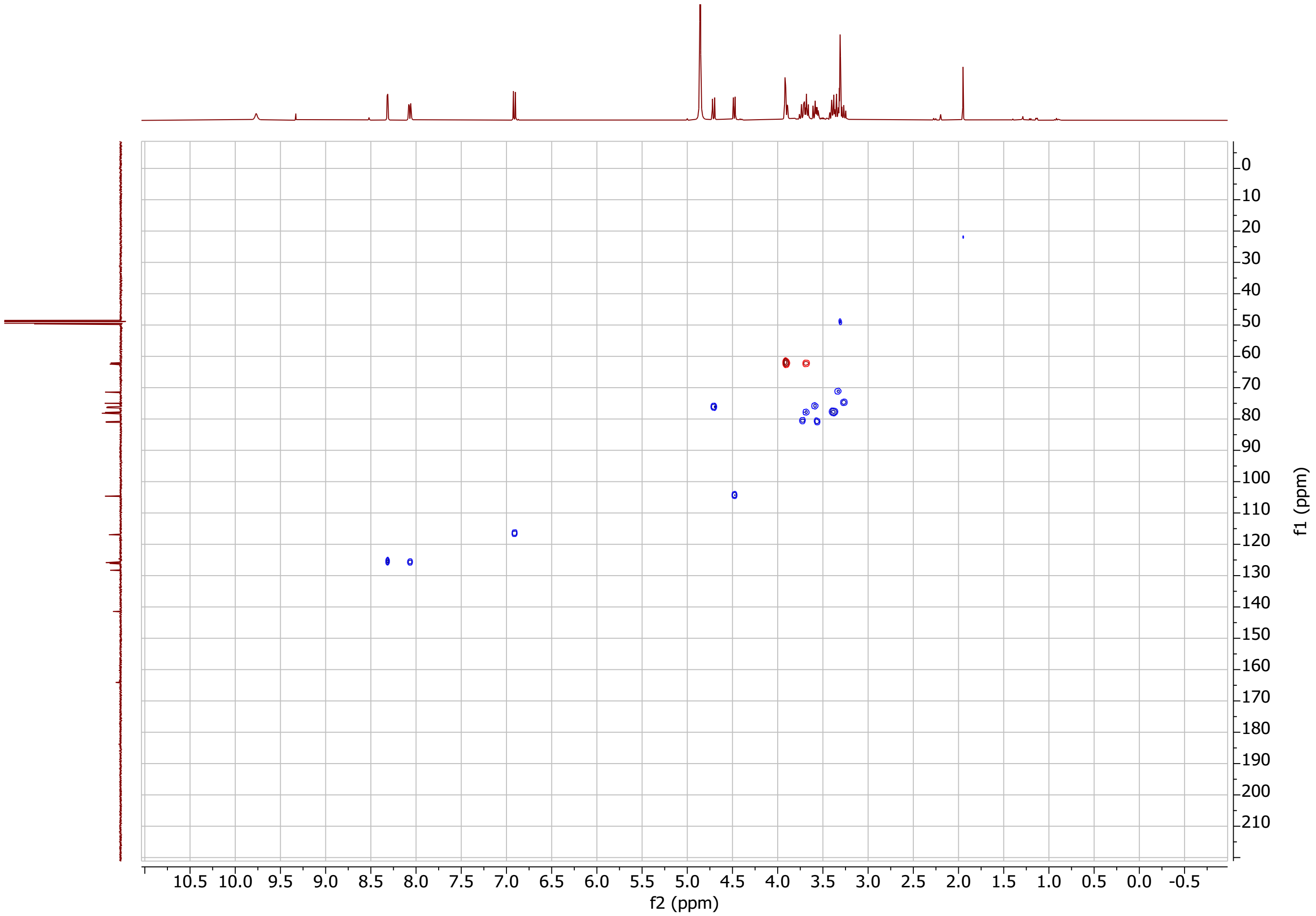
f2 (ppm)
f1 (ppm)

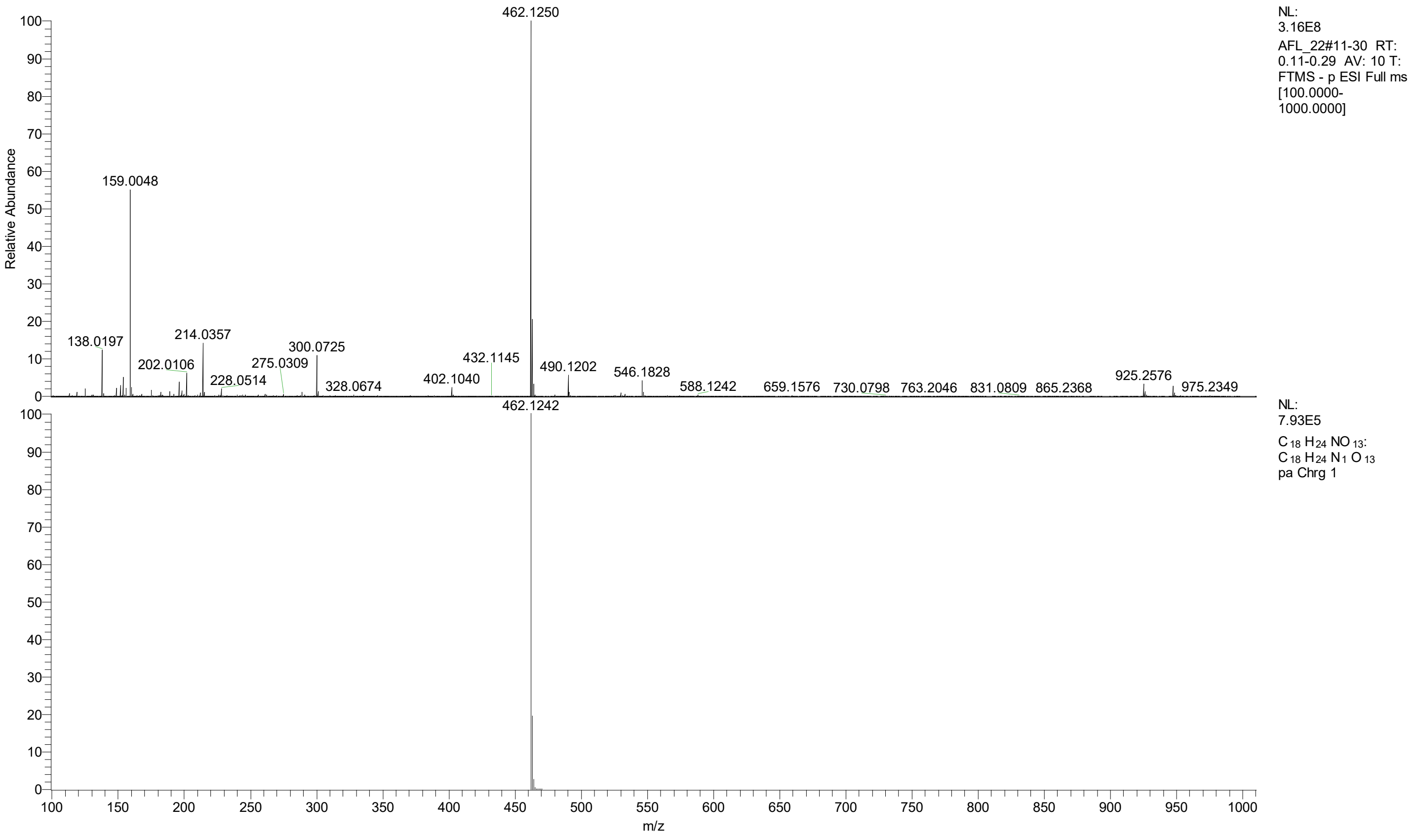
462.1250
NL:
3.16E8
AFL_22#11-30 RT:
0.11-0.29 AV: 10 T:
FTMS - p ESI Full ms
[100.0000-
1000.0000]
159.0048
138.0197
214.0357
202.0106
300.0725
275.0309
228.0514
328.0674
432.1145
402.1040
490.1202
546.1828
588.1242
659.1576
730.0798
763.2046
831.0809
865.2368
925.2576
975.2349
462.1242
NL:
7.93E5
C 18 H 24 NO 13:
C 18 H 24 N 1 O 13
pa Chrg 1
Relative Abundance
m/z

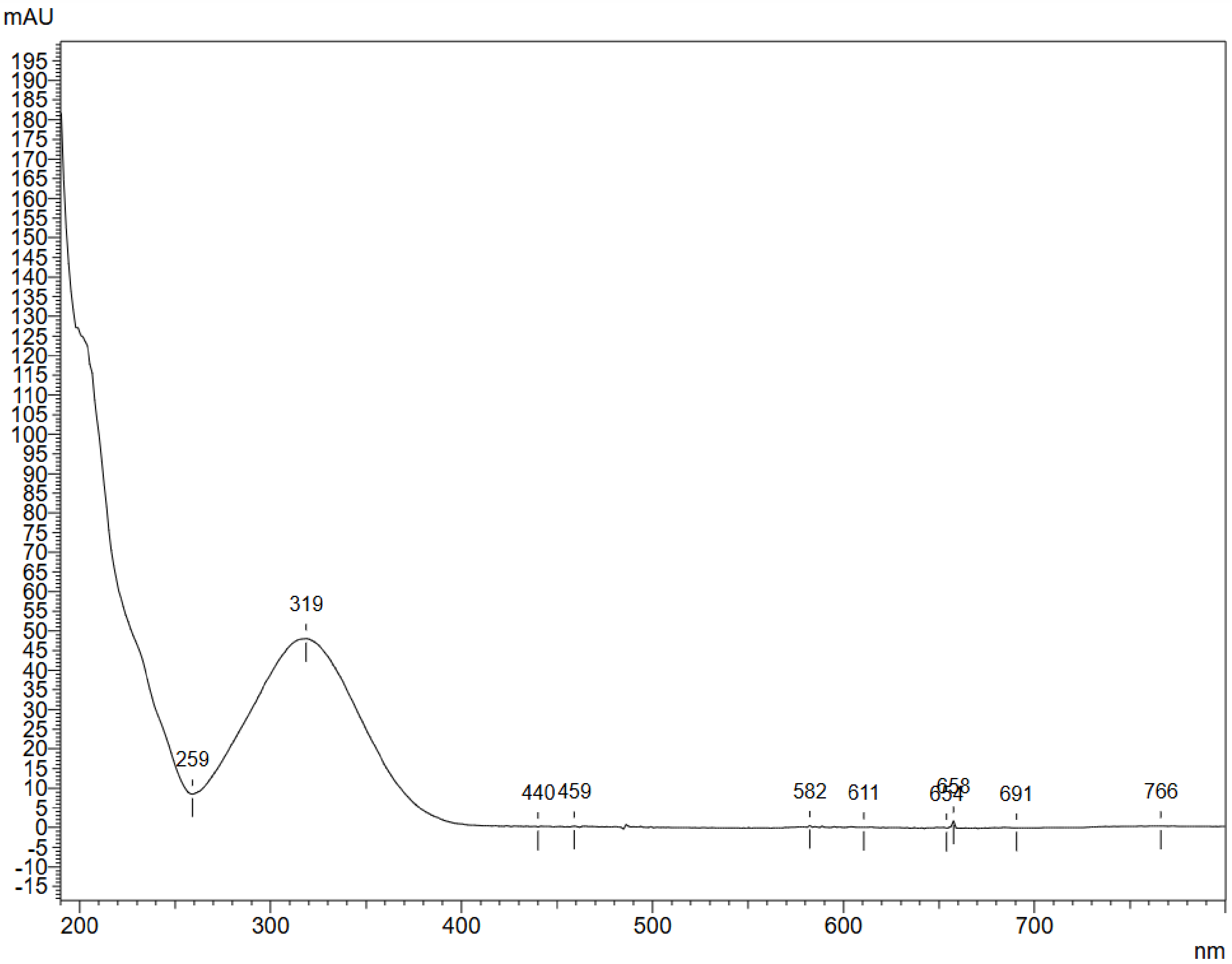
mAU
nm
319
259
440
459
582
611
654
658
691
766

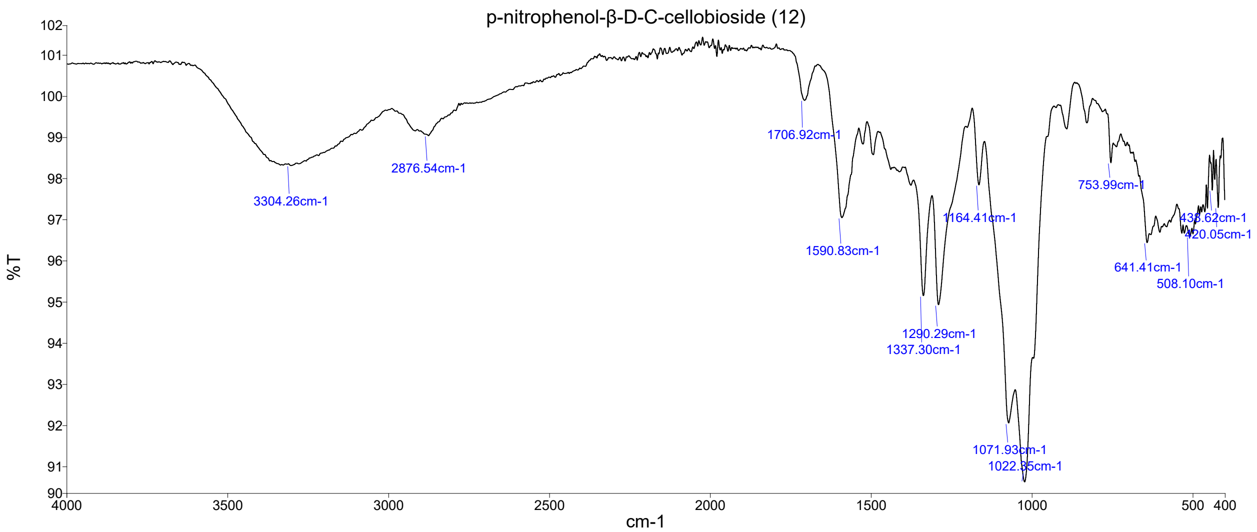
p-nitrophenol-β-D-C-cellobioside (12)
%T
cm-1
3304.26cm-1
2876.54cm-1
1706.92cm-1
1590.83cm-1
1337.30cm-1
1290.29cm-1
1164.41cm-1
1071.93cm-1
1022.35cm-1
753.99cm-1
641.41cm-1
508.10cm-1
438.62cm-1
420.05cm-1

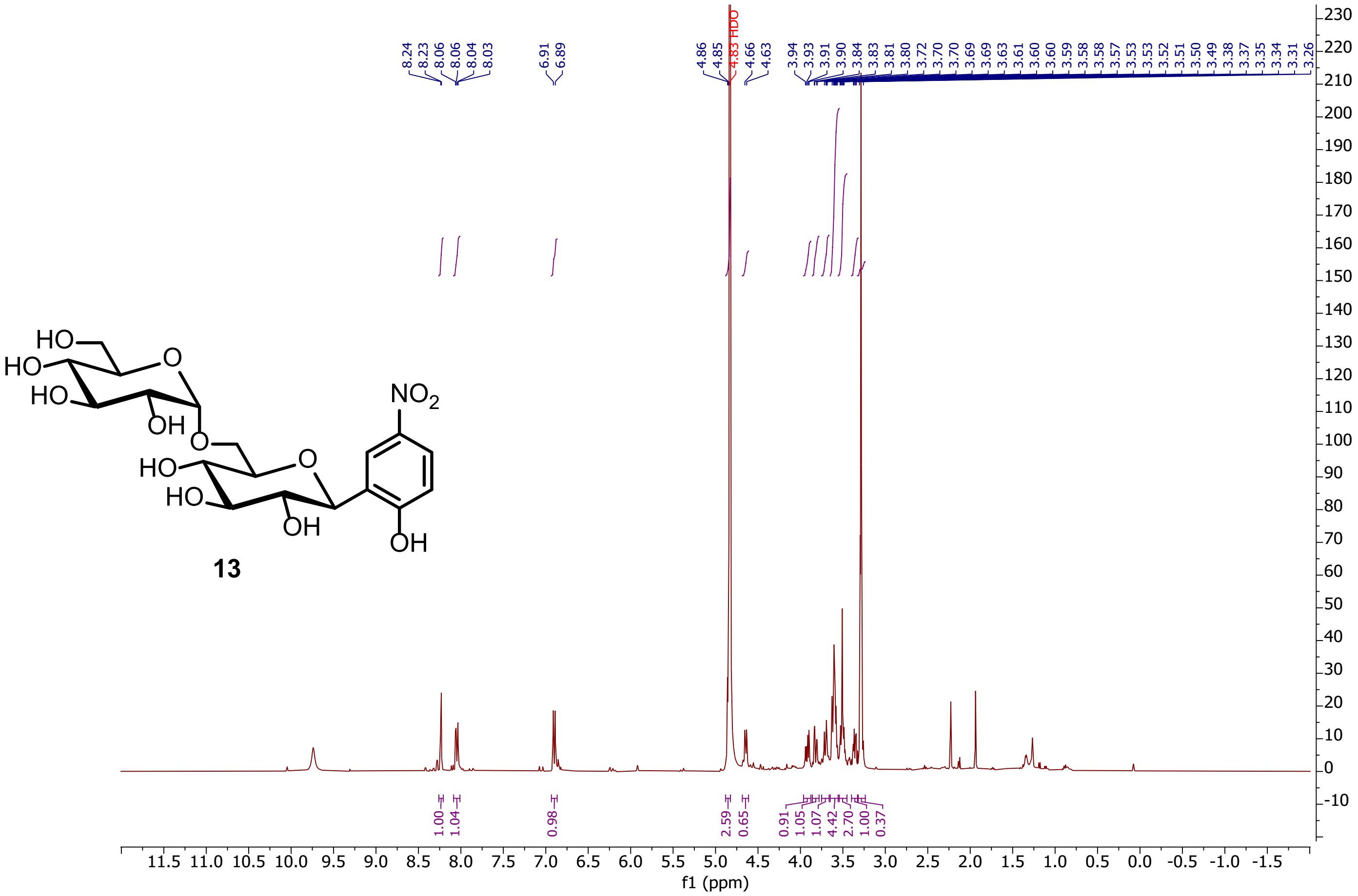
HO
HO
HO
OH
O
O
HO
HO
OH
O
NO2
OH
4.83 HDO
f1 (ppm)

13

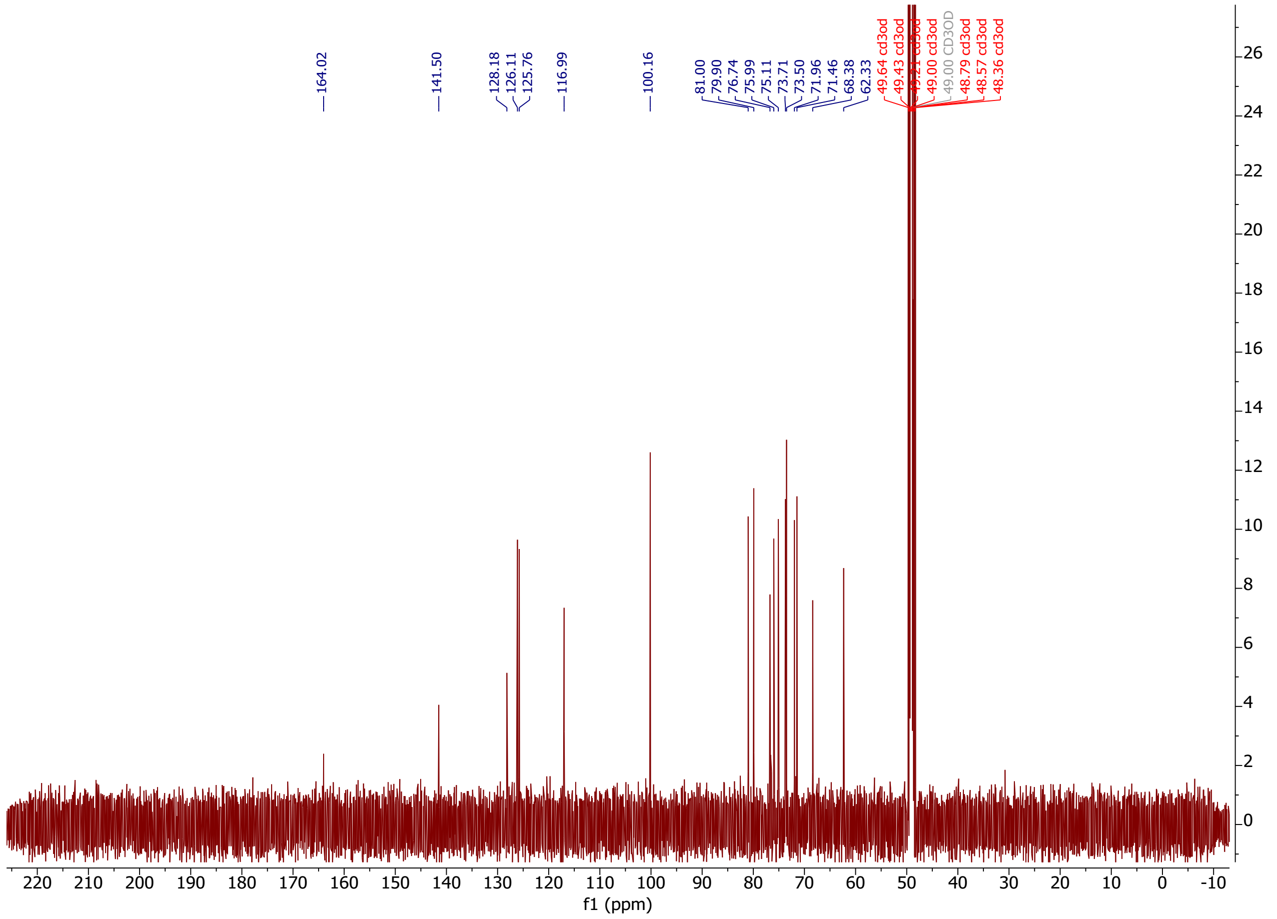
164.02
141.50
128.18
126.11
125.76
116.99
100.16
81.00
79.90
76.74
75.99
75.11
73.71
73.50
71.96
71.46
68.38
62.33
49.00 CD3OD
f1 (ppm)

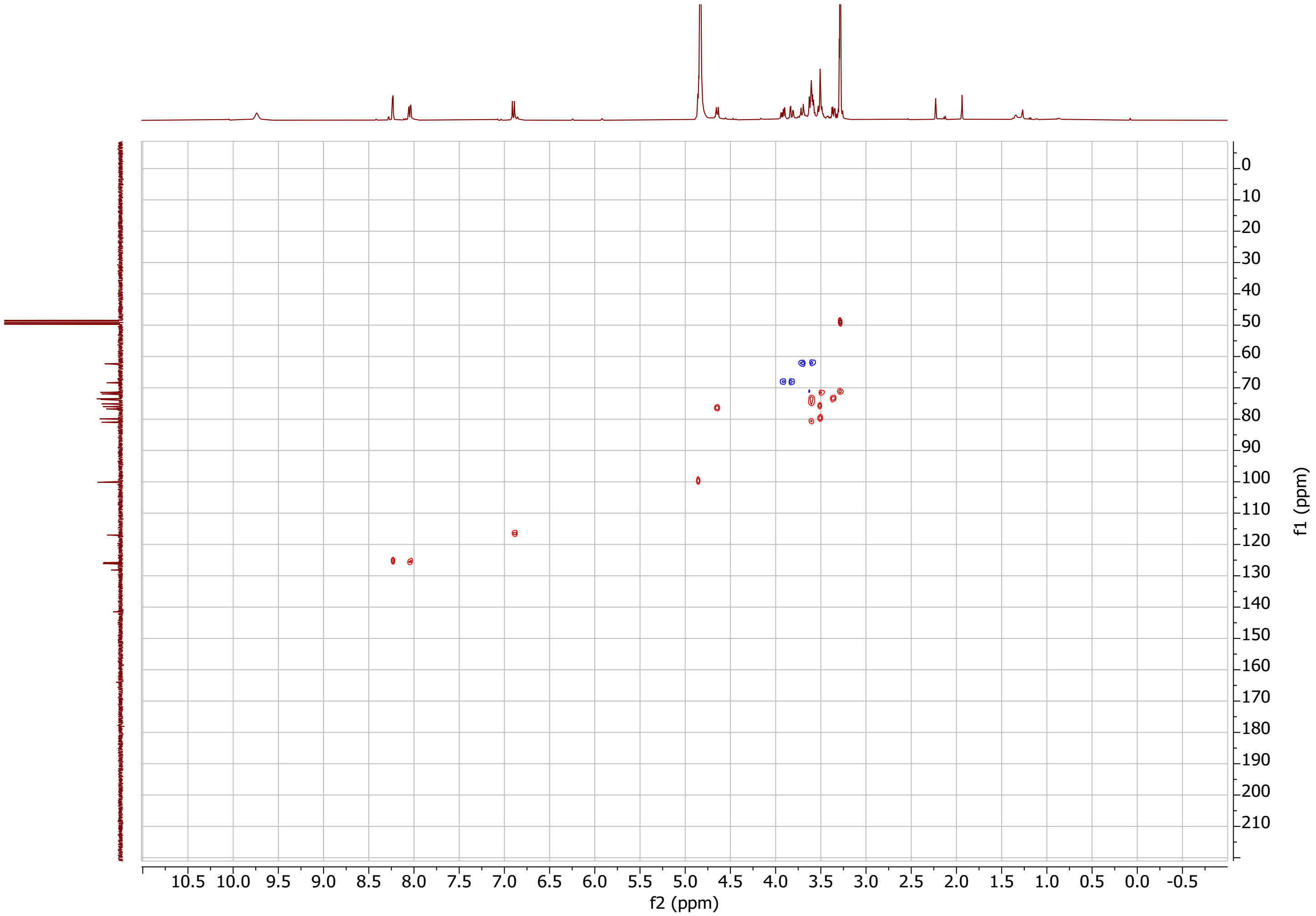

f2 (ppm)
f1 (ppm)

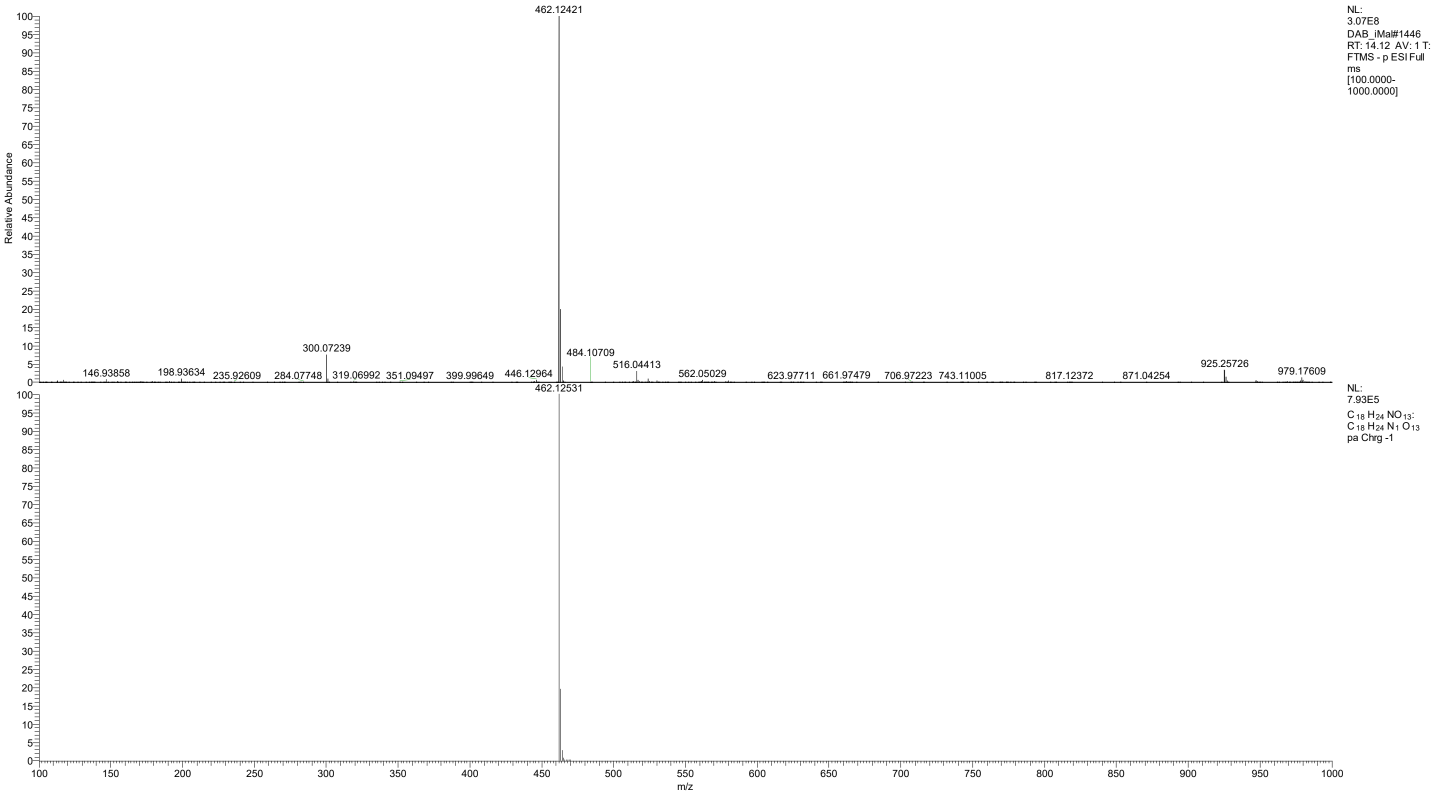

462.12421
300.07239
484.10709
516.04413
925.25726
979.17609
146.93858
198.93634
235.92609
284.07748
319.06992
351.09497
399.99649
446.12964
562.05029
623.97711
661.97479
706.97223
743.11005
817.12372
871.04254
NL: 3.07E8
DAB_iMal#1446
RT: 14.12 AV: 1 T: FTMS - p ESI Full ms [100.0000-1000.0000]
462.12531
NL: 7.93E5
C 18 H 24 NO 13: C 18 H 24 N 1 O 13
pa Chrg -1
Relative Abundance
m/z

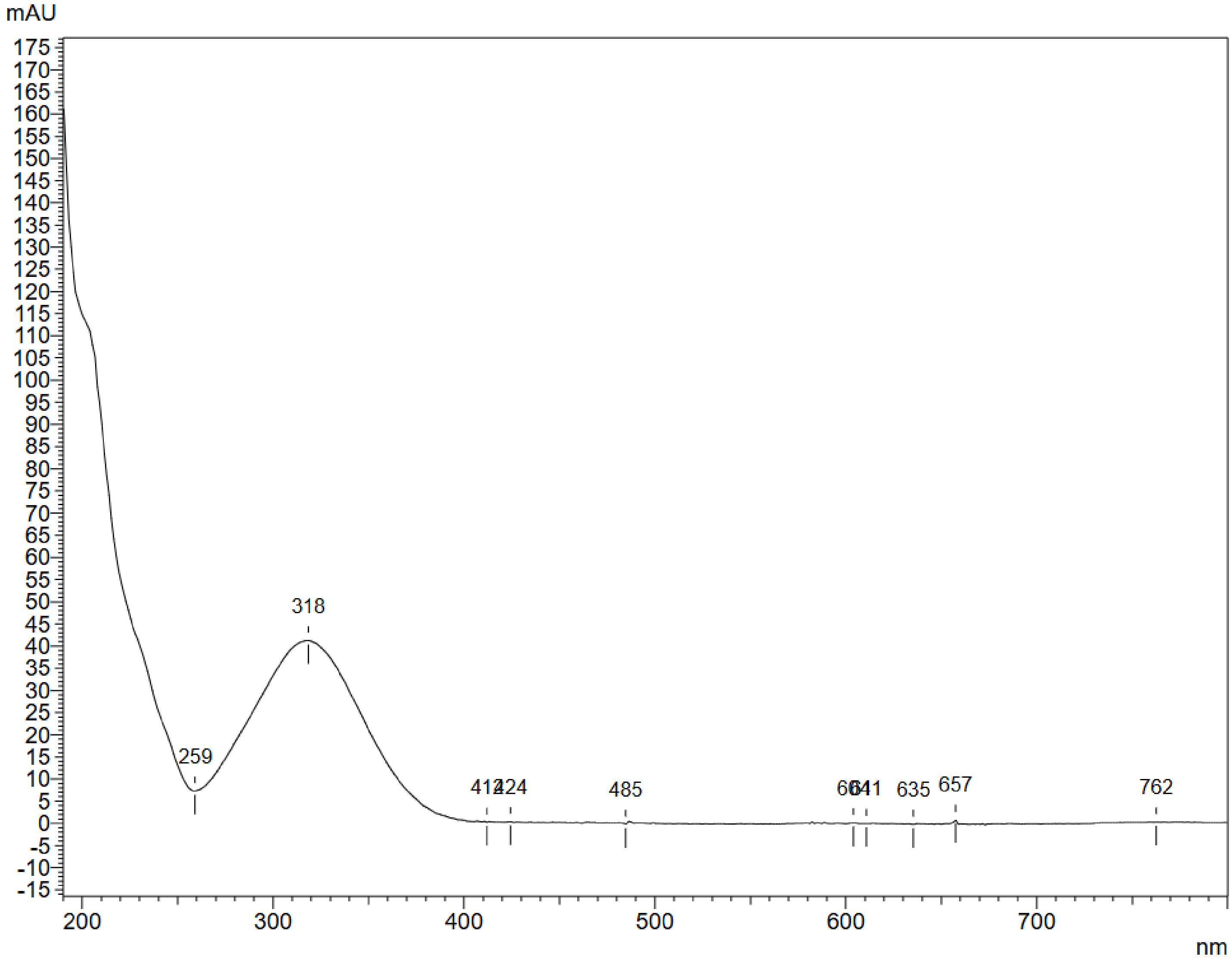
mAU
318
259
414
424
485
604
611
635
657
762
nm

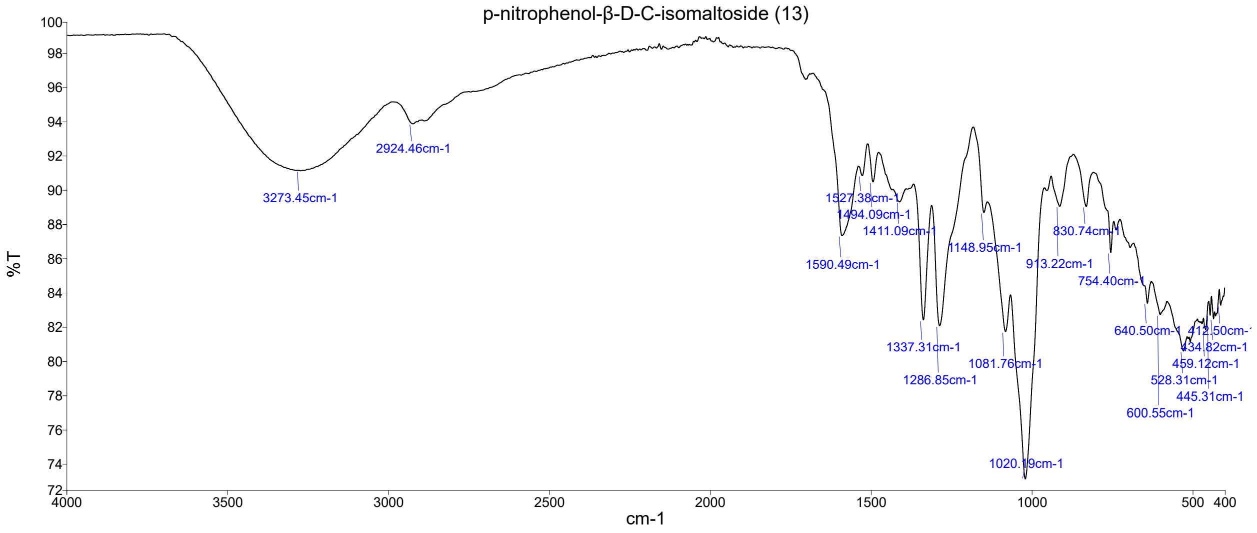
p-nitrophenol-β-D-C-isomaltoside (13)
%T
cm-1
3273.45cm-1
2924.46cm-1
1590.49cm-1
1527.38cm-1
1494.09cm-1
1411.09cm-1
1337.31cm-1
1286.85cm-1
1148.95cm-1
1081.76cm-1
1020.19cm-1
913.22cm-1
830.74cm-1
754.40cm-1
640.50cm-1
600.55cm-1
528.31cm-1
459.12cm-1
445.31cm-1
434.82cm-1

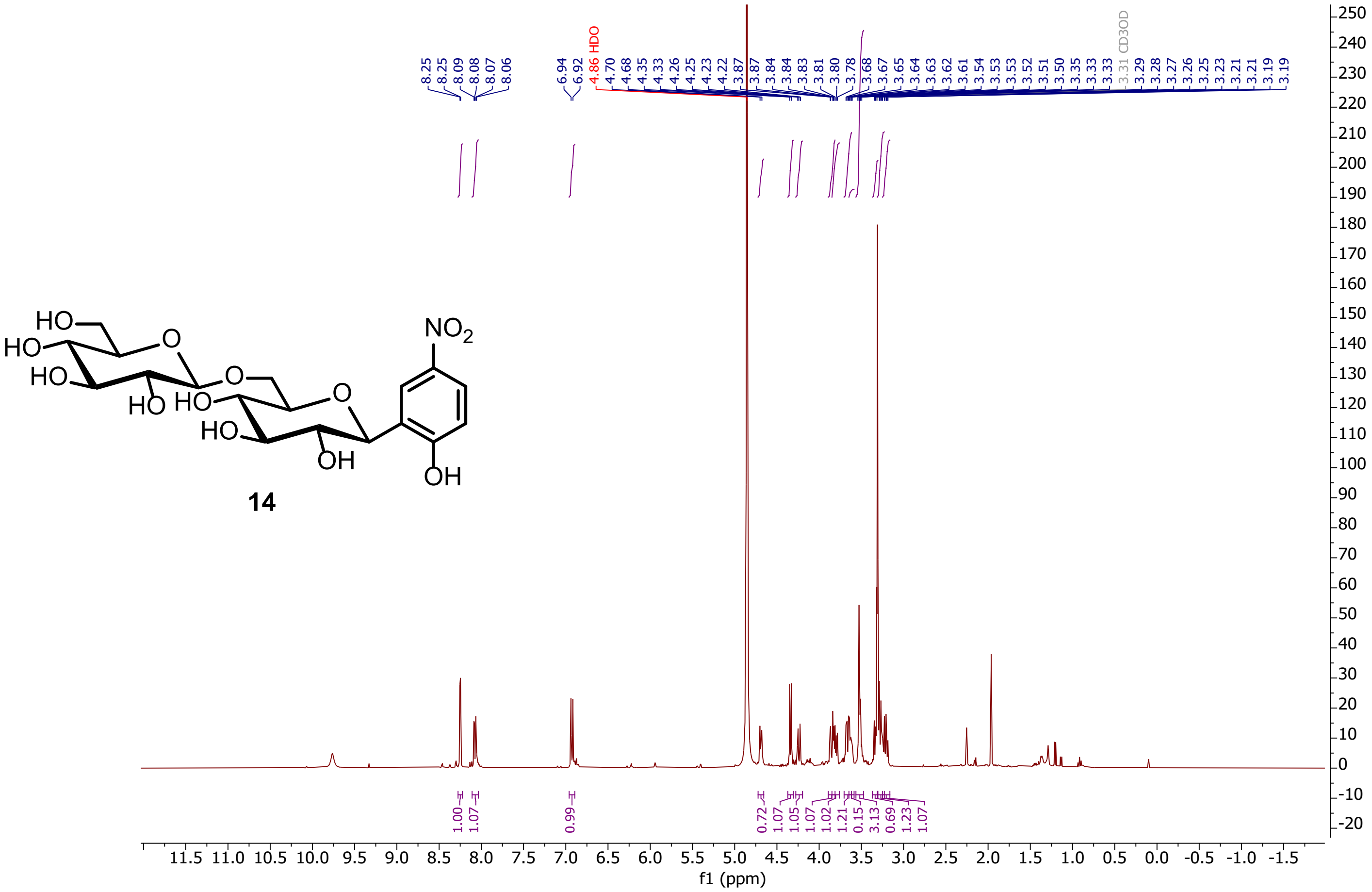

8.25 8.25 8.09 8.08 8.07 8.06 6.94 6.92 4.86 HDO 4.70 4.68 4.35 4.33 4.26 4.25 4.23 4.22 3.87 3.87 3.84 3.84 3.83 3.81 3.80 3.78 3.68 3.67 3.65 3.64 3.63 3.62 3.61 3.54 3.53 3.53 3.52 3.51 3.50 3.35 3.33 3.33 3.31 CD3OD 3.29 3.28 3.27 3.26 3.25 3.23 3.21 3.21 3.19 3.19
1.00 1.07 0.99 0.72 1.07 1.05 1.07 1.02 1.21 0.15 3.13 0.69 1.23 1.07
f1 (ppm)
HO
HO
HO
HO
O
O
HO
HO
O
OH
NO2
OH

14

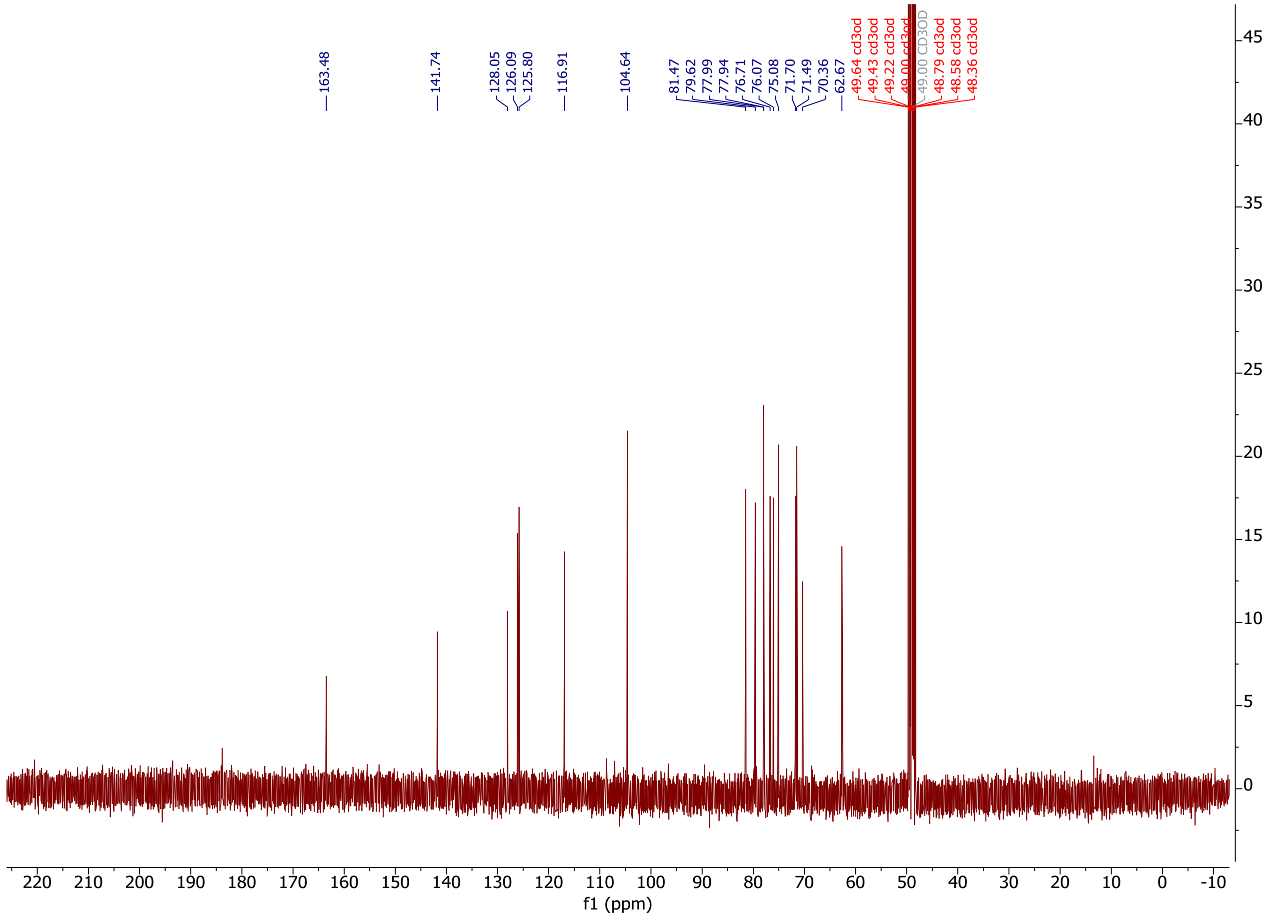

163.48 141.74 128.05 126.09 125.80 116.91 104.64 81.47 79.62 77.99 77.94 76.71 76.07 75.08 71.70 71.49 70.36 62.67
49.64 cd3od 49.43 cd3od 49.22 cd3od 49.00 cd3od 49.00 CD3OD 48.79 cd3od 48.58 cd3od 48.36 cd3od
f1 (ppm)

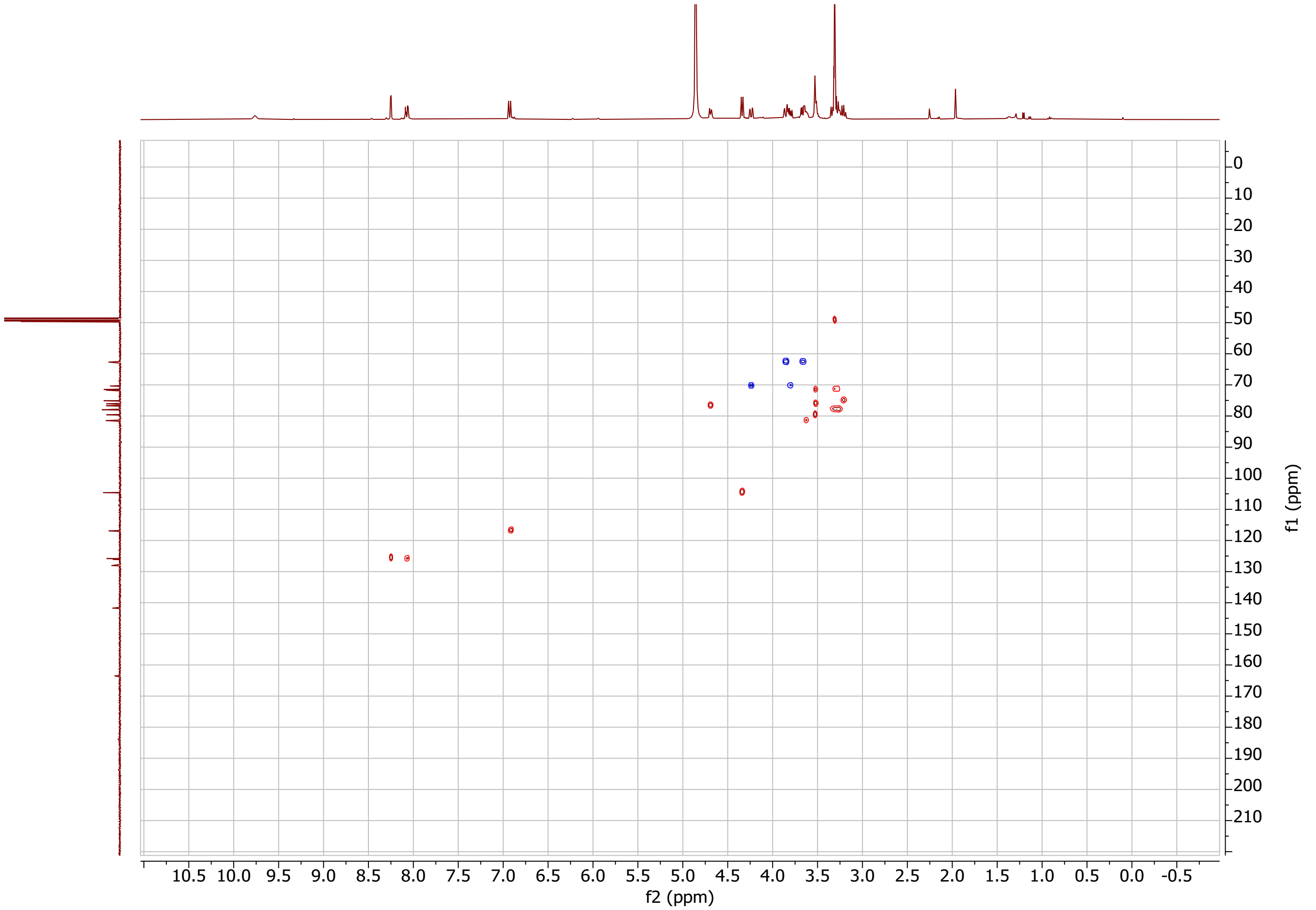
f1 (ppm)
f2 (ppm)
0
10
20
30
40
50
60
70
80
90
100
110
120
130
140
150
160
170
180
190
200
210
10.5 10.0 9.5 9.0 8.5 8.0 7.5 7.0 6.5 6.0 5.5 5.0 4.5 4.0 3.5 3.0 2.5 2.0 1.5 1.0 0.5 0.0 -0.5

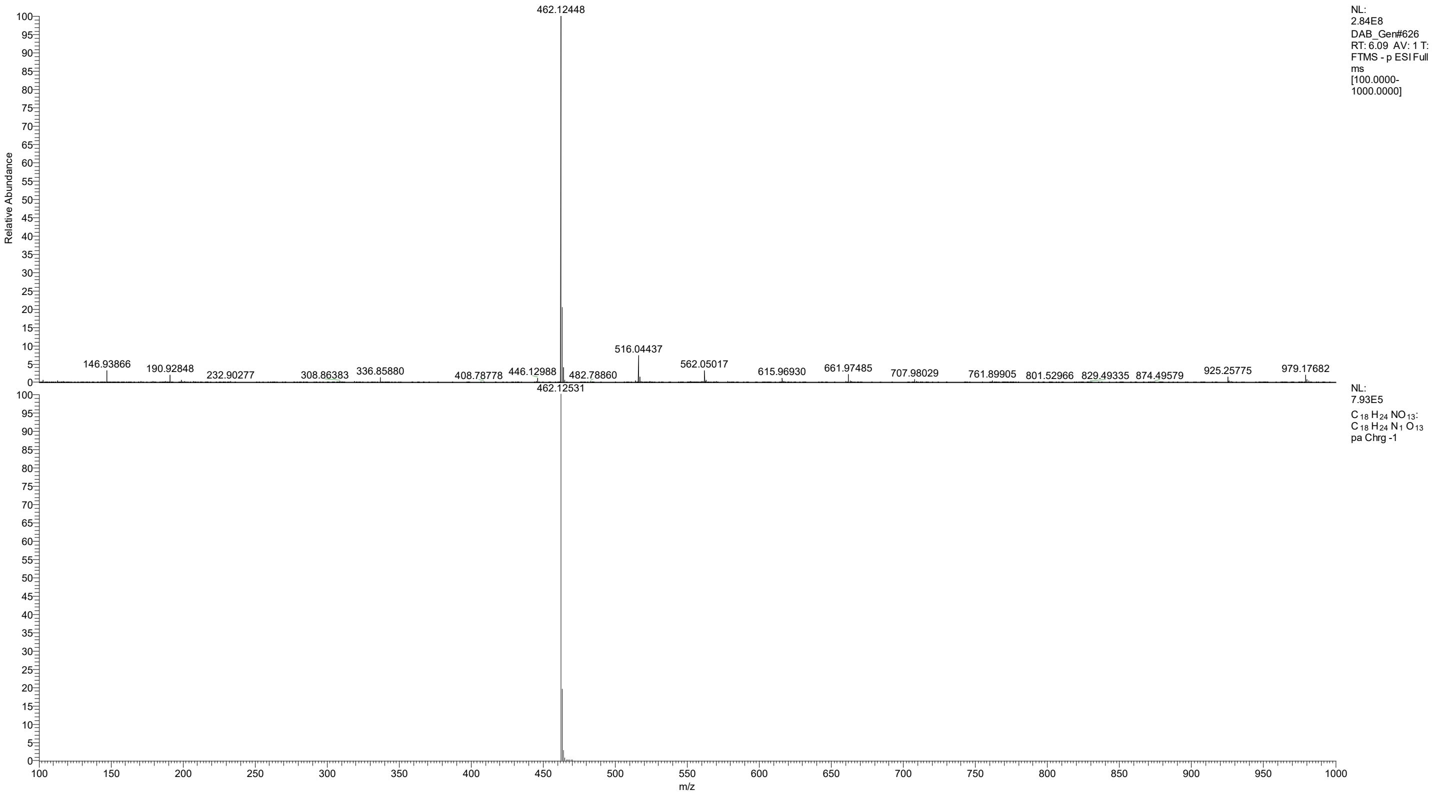
Relative Abundance
m/z
462.12448
146.93866
190.92848
232.90277
308.86383
336.85880
408.78778
446.12988
482.78860
516.04437
562.05017
615.96930
661.97485
707.98029
761.89905
801.52966
829.49335
874.49579
925.25775
979.17682
462.12531
NL:
2.84E8
DAB_Gen#626
RT: 6.09 AV: 1 T:
FTMS - p ESI Full
ms
[100.0000-
1000.0000]
NL:
7.93E5
C 18 H 24 NO 13:
C 18 H 24 N 1 O 13
pa Chrg -1

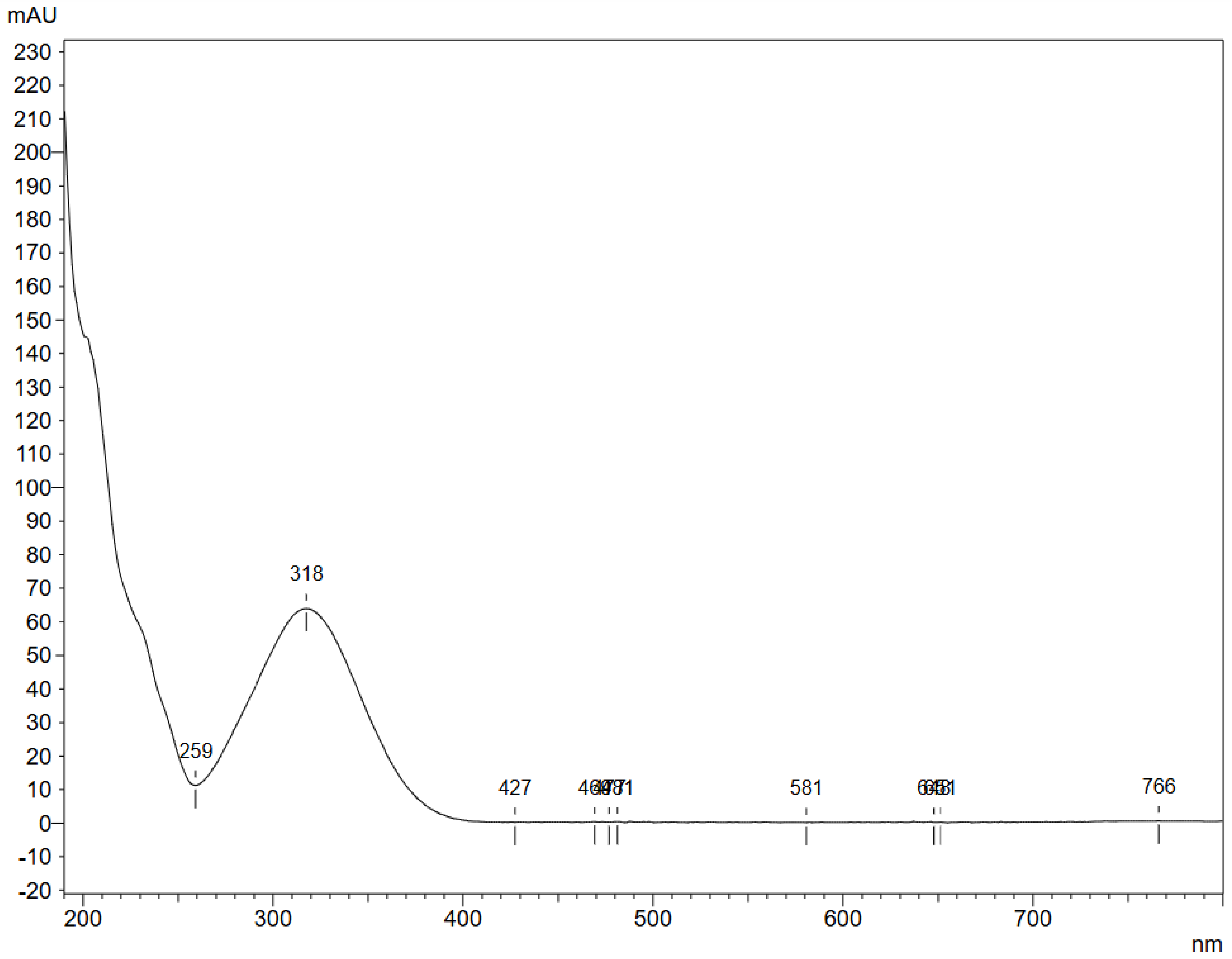

mAU
318
259
427
581
766
nm

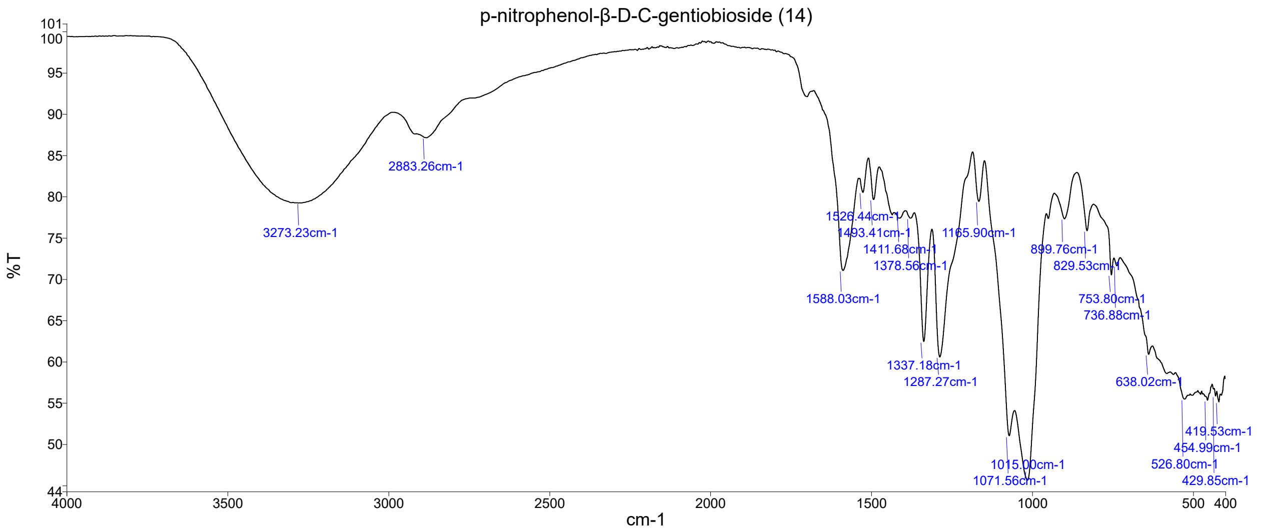

p-nitrophenol-β-D-C-gentiobioside (14)
%T
cm-1
3273.23cm-1
2883.26cm-1
1588.03cm-1
1526.44cm-1
1493.41cm-1
1411.68cm-1
1378.56cm-1
1337.18cm-1
1287.27cm-1
1165.90cm-1
1071.56cm-1
1015.00cm-1
899.76cm-1
829.53cm-1
753.80cm-1
736.88cm-1
638.02cm-1
526.80cm-1
454.99cm-1
429.85cm-1
419.53cm-1

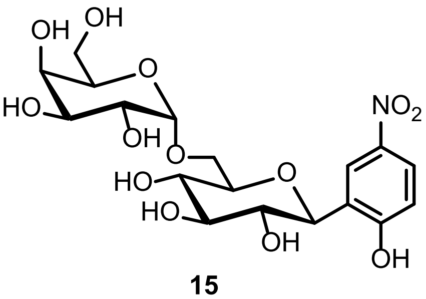
OH
OH
O
HO
OH
O
NO2
HO
O
HO
OH
OH

15

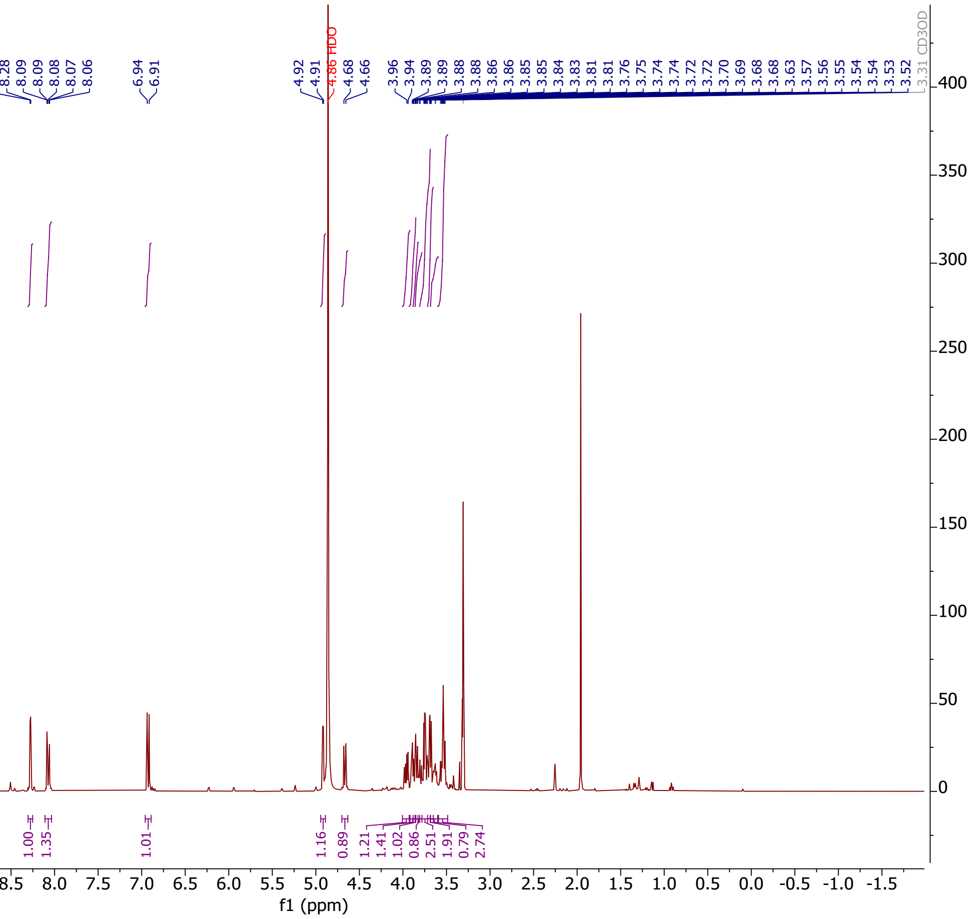
8.28
8.28
8.09
8.09
8.08
8.07
8.06
6.94
6.91
4.92
4.91
4.86 HDO
4.68
4.66
3.96
3.94
3.89
3.89
3.88
3.88
3.86
3.86
3.85
3.85
3.84
3.83
3.81
3.81
3.76
3.75
3.74
3.74
3.72
3.72
3.70
3.69
3.68
3.68
3.63
3.57
3.56
3.55
3.54
3.54
3.53
3.52
3.31 CD3OD
1.00
1.35
1.01
1.16
0.89
1.21
1.41
1.02
0.86
2.51
1.91
0.79
2.74
f1 (ppm)

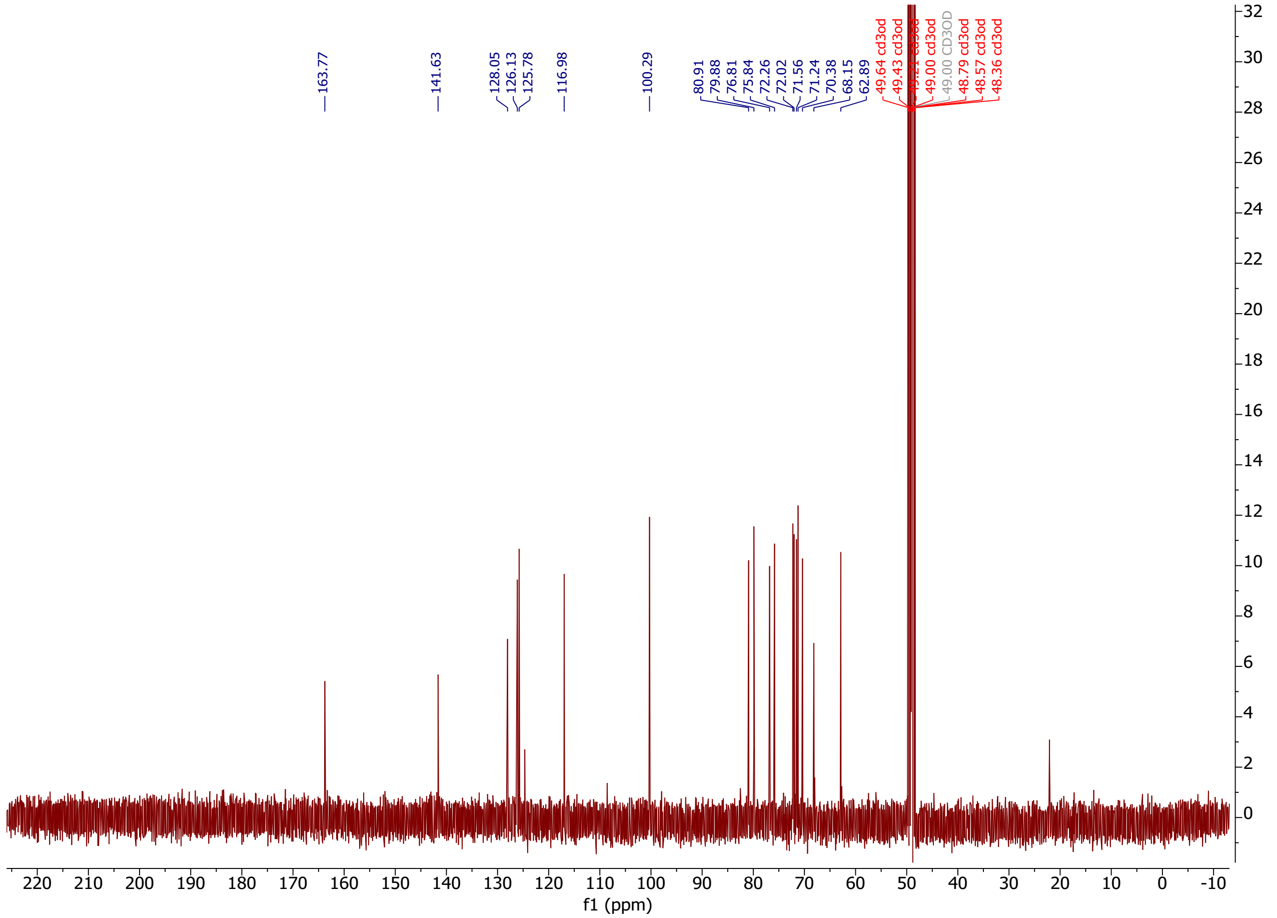
163.77
141.63
128.05
126.13
125.78
116.98
100.29
80.91
79.88
76.81
75.84
72.26
72.02
71.56
71.24
70.38
68.15
62.89
49.64 cd3od
49.43 cd3od
49.00 cd3od
49.00 CD3OD
48.79 cd3od
48.57 cd3od
48.36 cd3od
f1 (ppm)

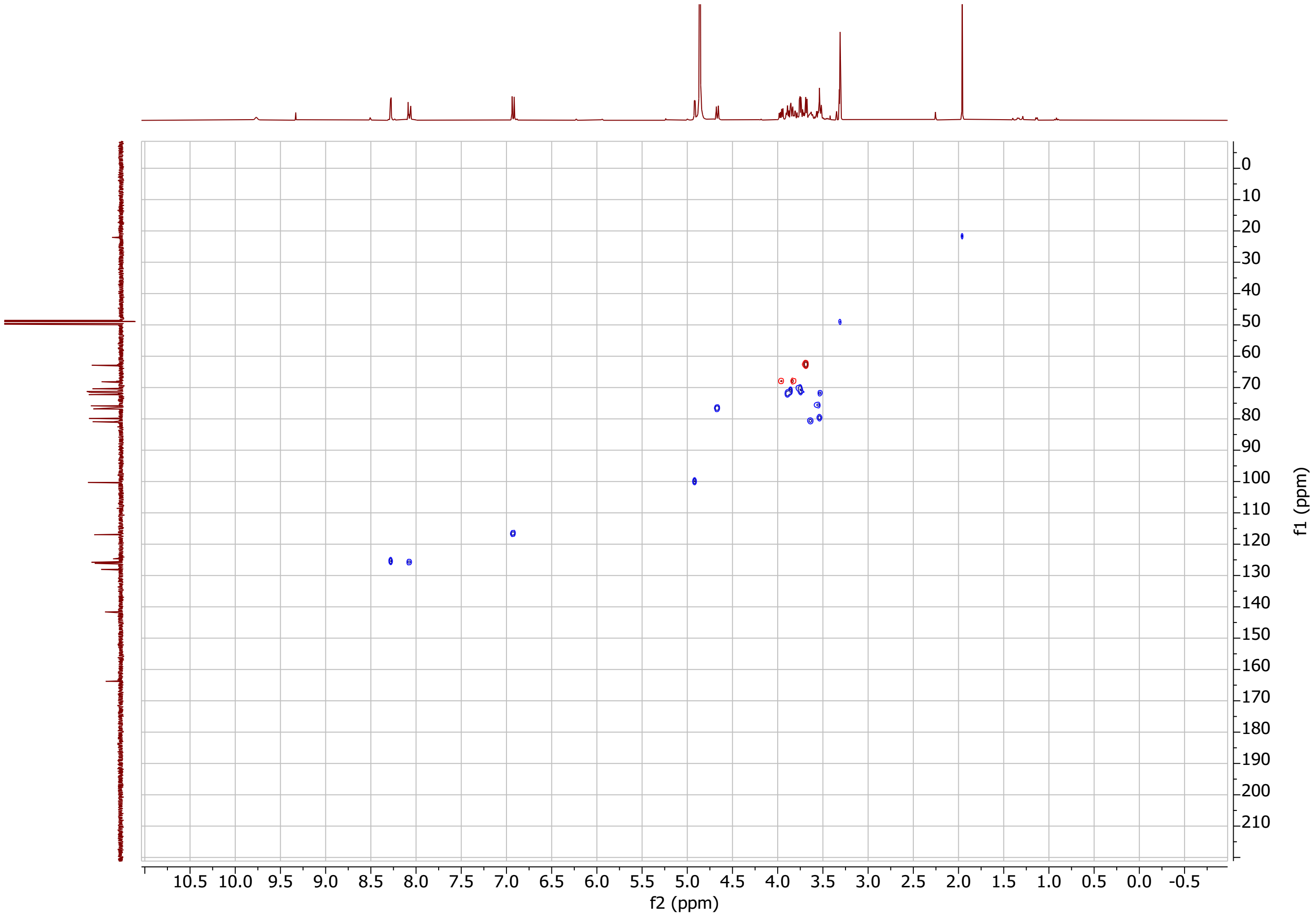

f2 (ppm)
f1 (ppm)

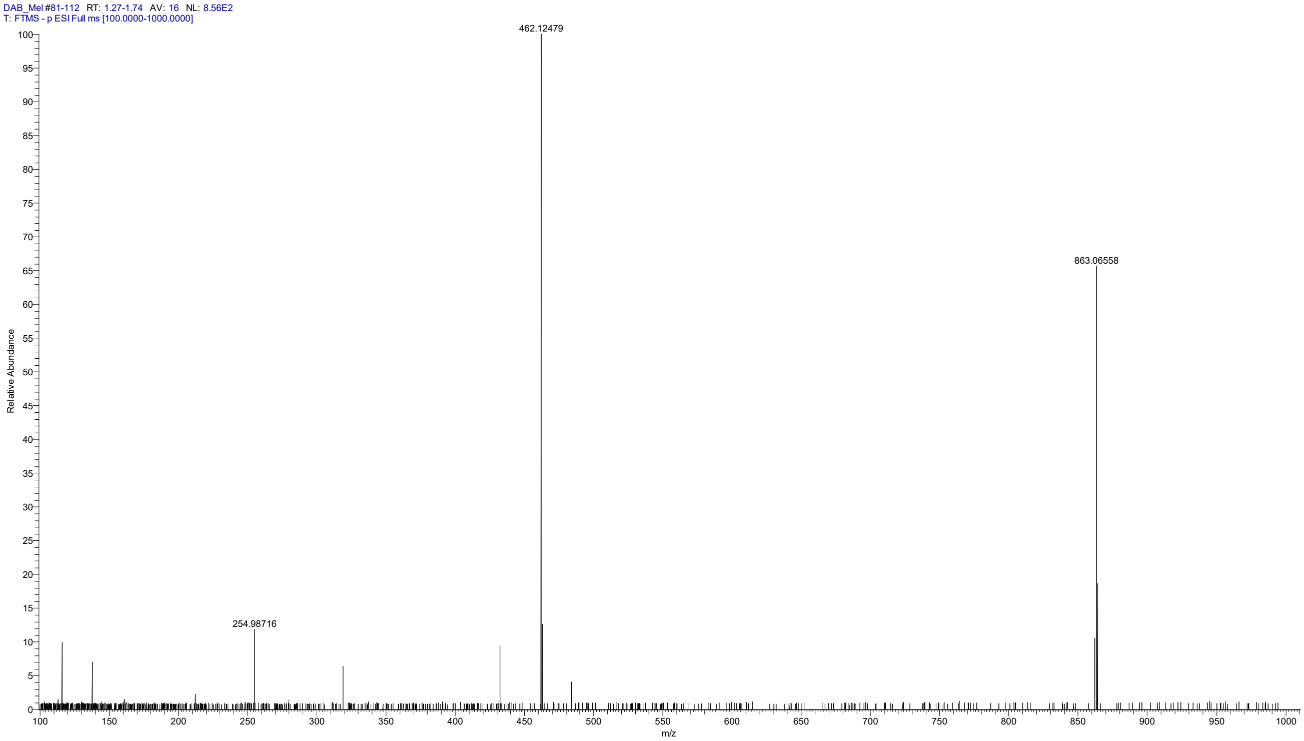

DAB_Mel #81-112 RT: 1.27-1.74 AV: 16 NL: 8.56E2
T: FTMS - p ESI Full ms [100.0000-1000.0000]
462.12479
863.06558
254.98716
Relative Abundance
m/z

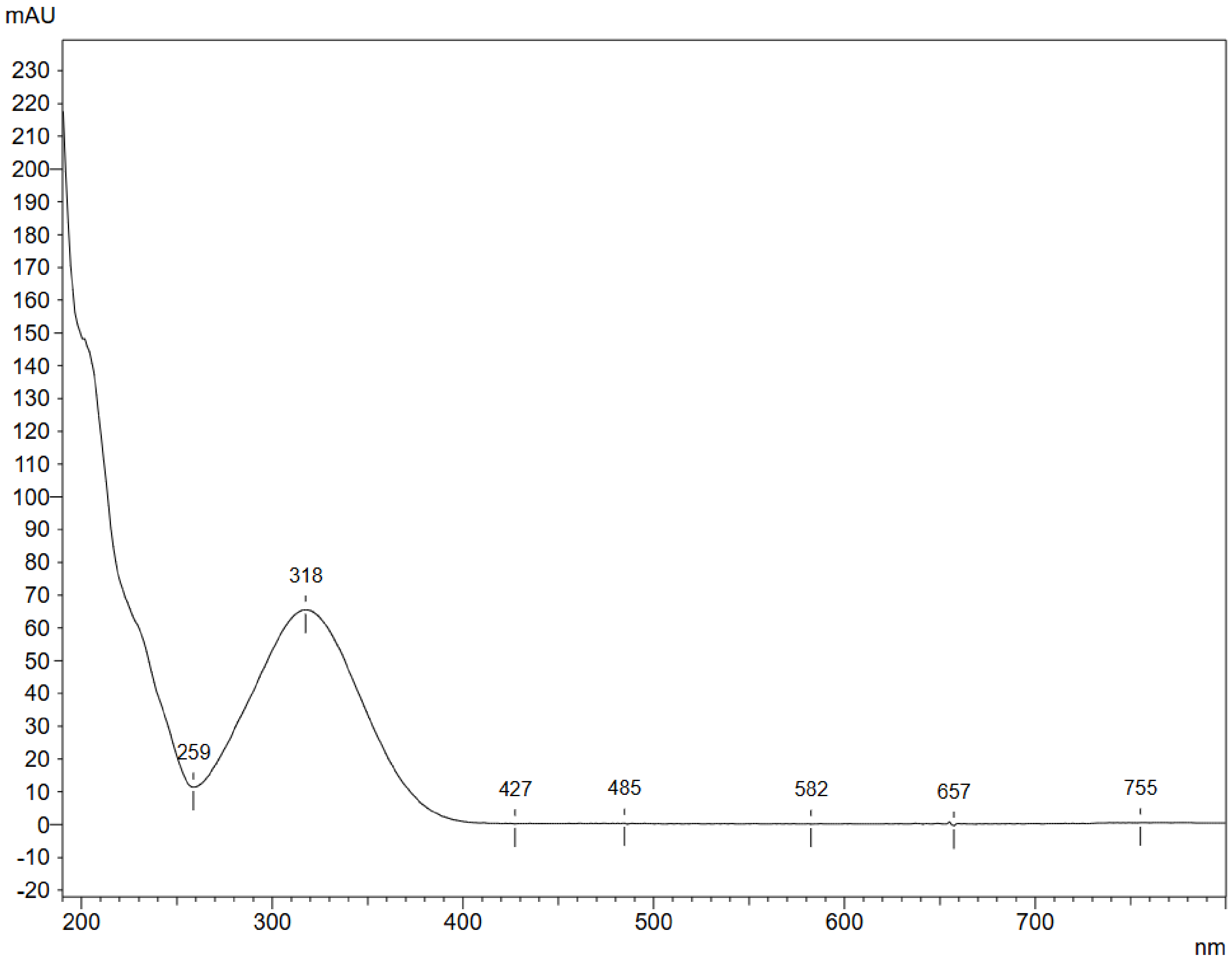

p-nitrophenol-β-D-C-melibioside (15)

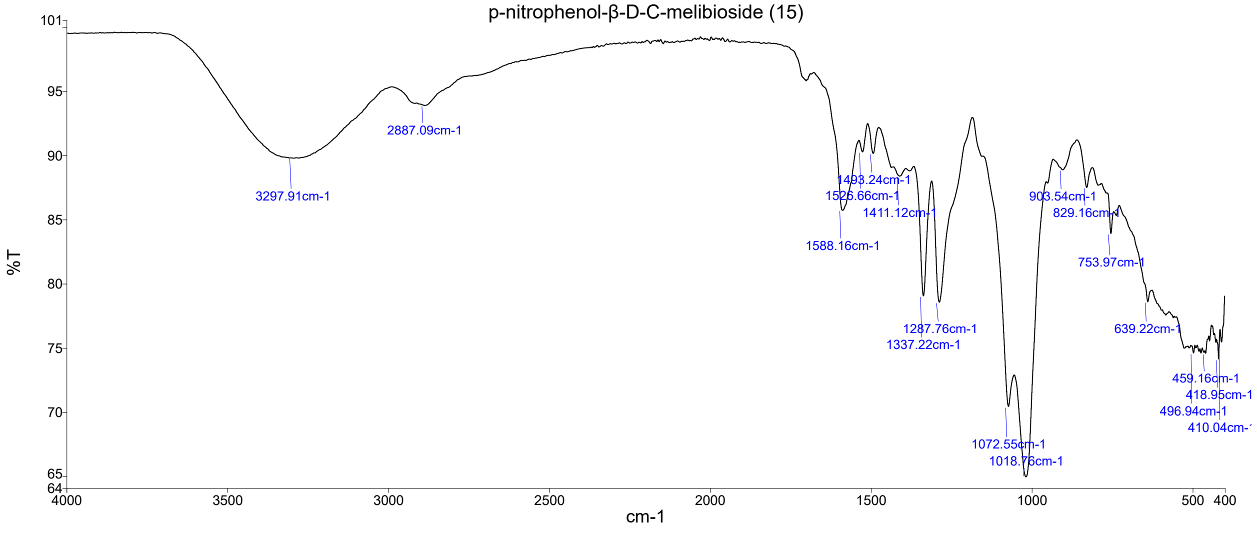

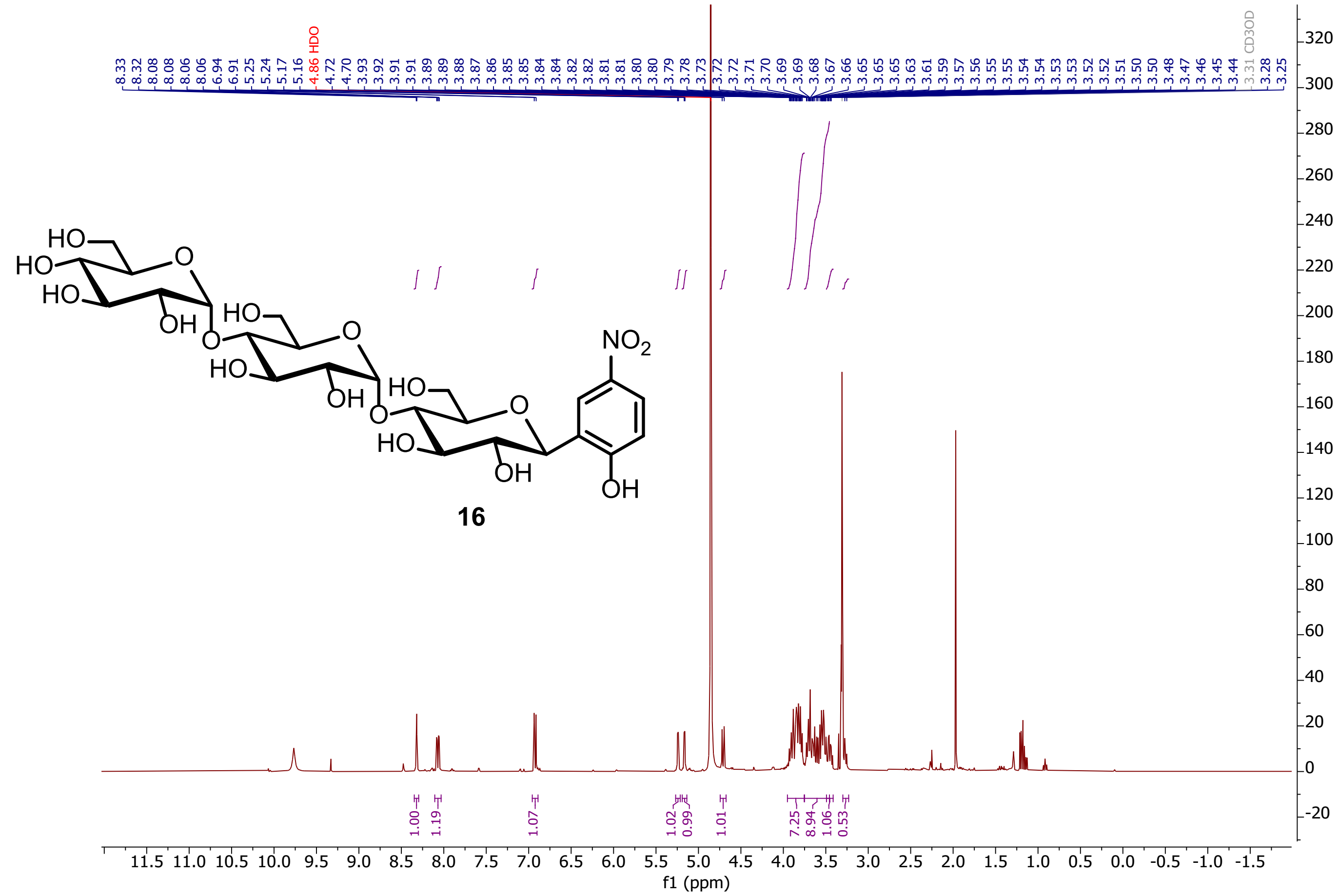
8.33 8.32 8.08 8.08 8.06 8.06 6.94 6.91 5.25 5.24 5.17 5.16 4.86 HDO 4.72 4.70 3.93 3.92 3.91 3.91 3.89 3.89 3.88 3.87 3.86 3.85 3.85 3.84 3.84 3.82 3.82 3.81 3.81 3.80 3.80 3.79 3.78 3.73 3.72 3.72 3.71 3.70 3.69 3.69 3.68 3.67 3.66 3.65 3.65 3.63 3.61 3.59 3.57 3.56 3.55 3.55 3.54 3.54 3.53 3.52 3.52 3.51 3.50 3.50 3.48 3.47 3.46 3.45 3.44 3.31 CD3OD 3.28 3.25
1.00 1.19 1.07 1.02 0.99 1.01 7.25 8.94 1.06 0.53
NO2
HO
OH
O
16
f1 (ppm)

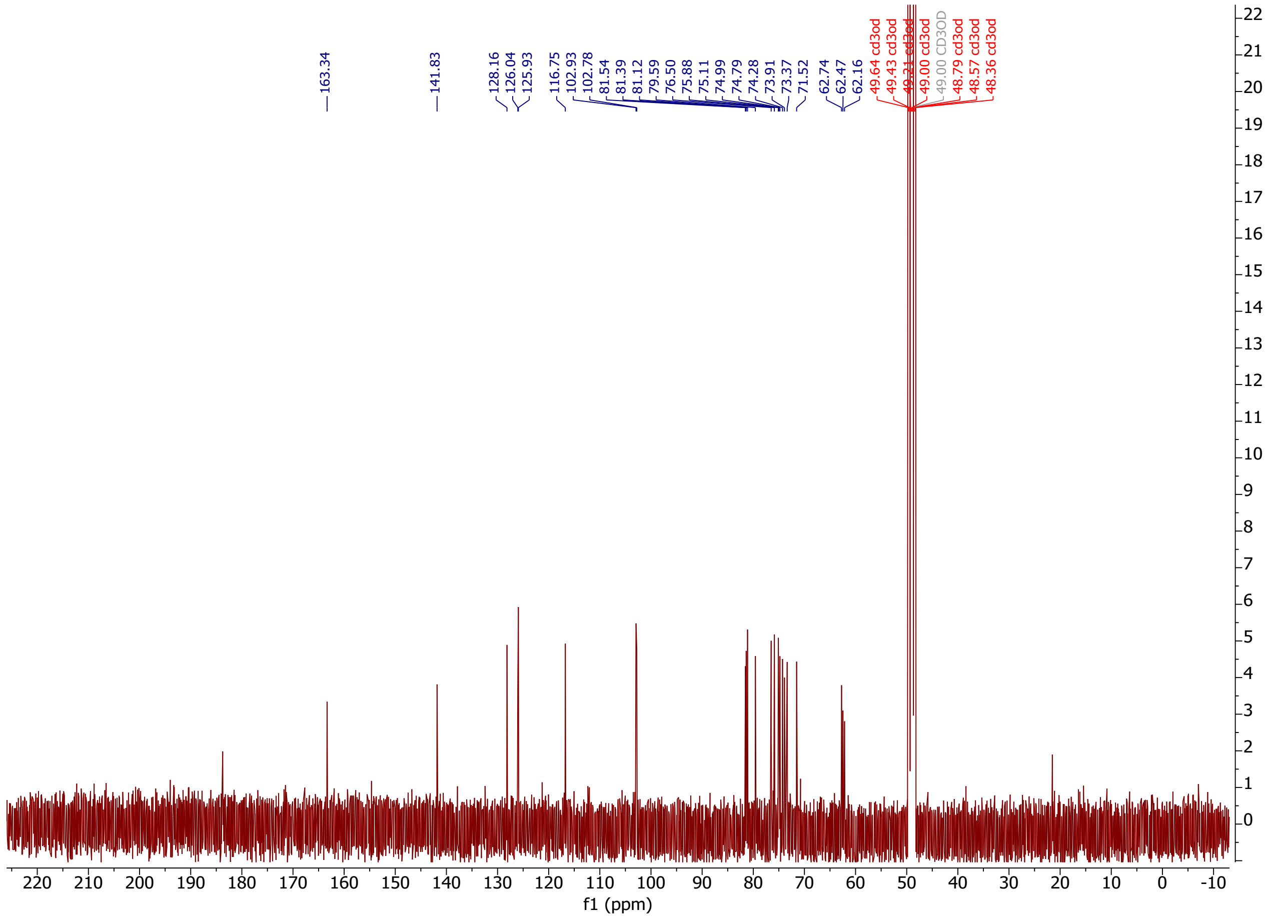
163.34 141.83 128.16 126.04 125.93 116.75 102.93 102.78 81.54 81.39 81.12 79.59 76.50 75.88 75.11 74.99 74.79 74.28 73.91 73.37 71.52 62.74 62.47 62.16 49.64 cd3od 49.43 cd3od 49.21 cd3od 49.00 cd3od 49.00 CD3OD 48.79 cd3od 48.57 cd3od 48.36 cd3od
f1 (ppm)

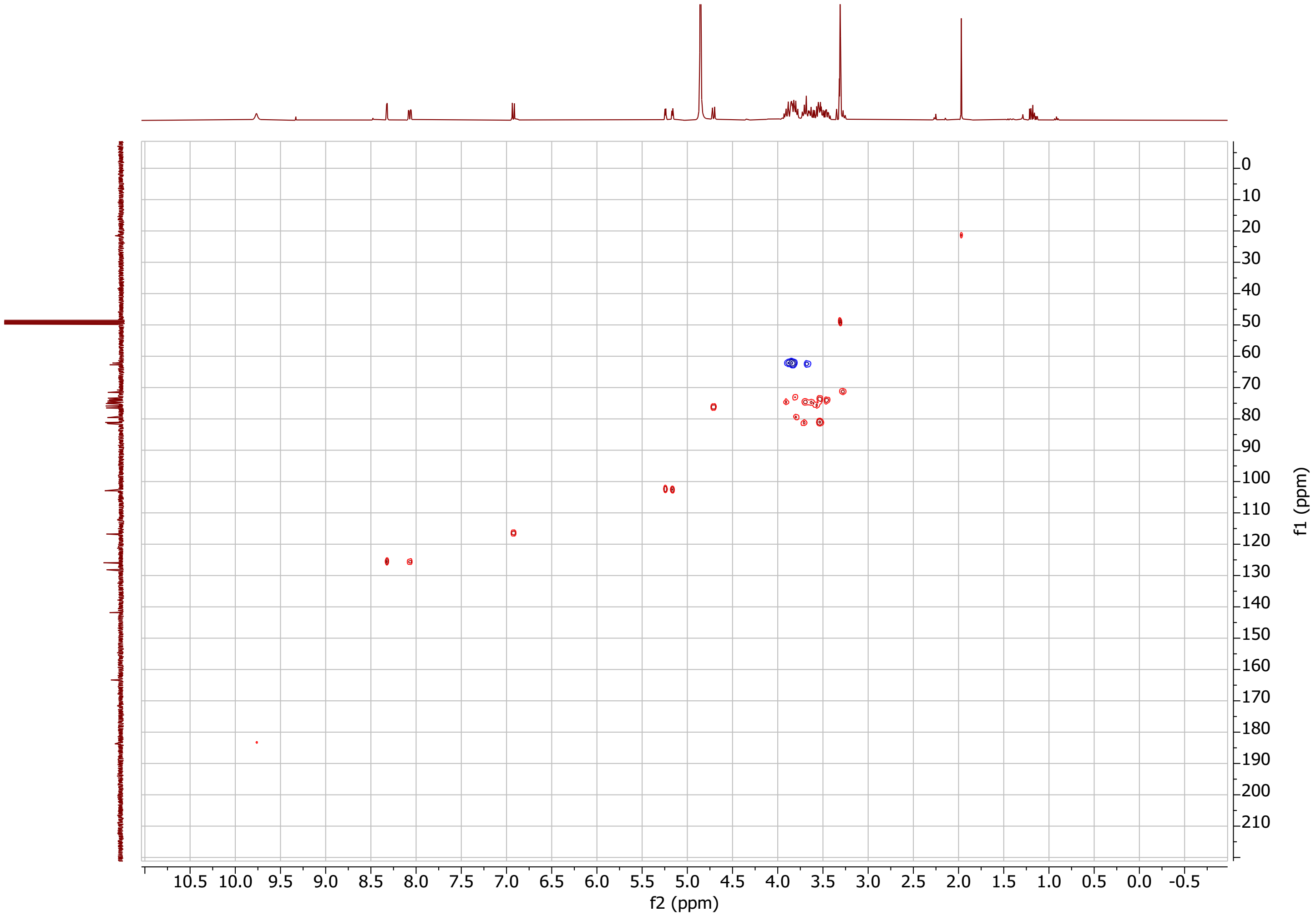
f2 (ppm)
f1 (ppm)

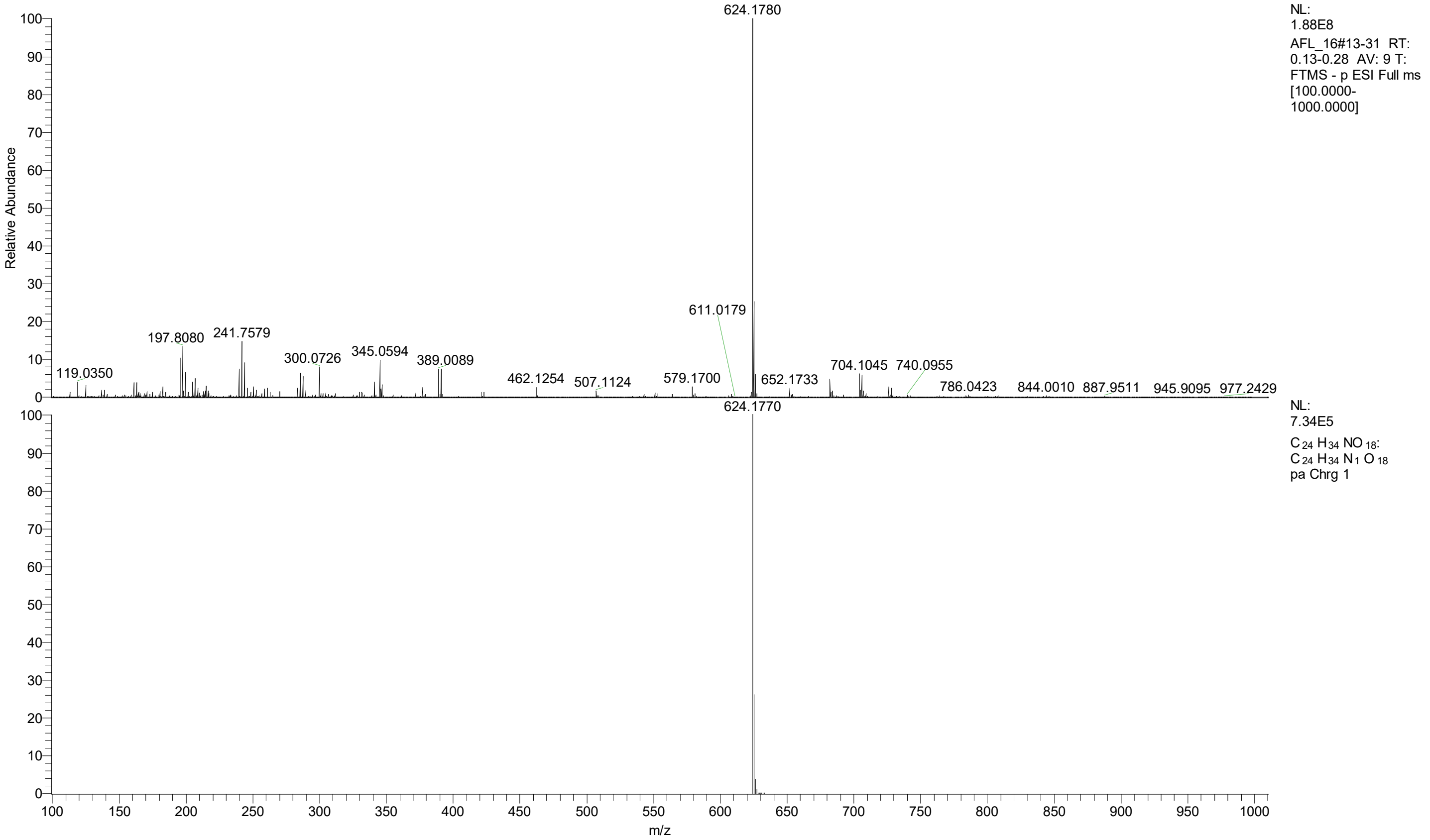
624.1780
NL: 1.88E8
AFL_16#13-31 RT: 0.13-0.28 AV: 9 T: FTMS - p ESI Full ms [100.0000-1000.0000]
611.0179
197.8080
241.7579
345.0594
300.0726
389.0089
119.0350
462.1254
507.1124
579.1700
652.1733
704.1045
740.0955
786.0423
844.0010
887.9511
945.9095
977.2429
624.1770
NL: 7.34E5
C 24 H34 NO 18: C 24 H34 N1 O 18
pa Chrg 1
Relative Abundance
m/z

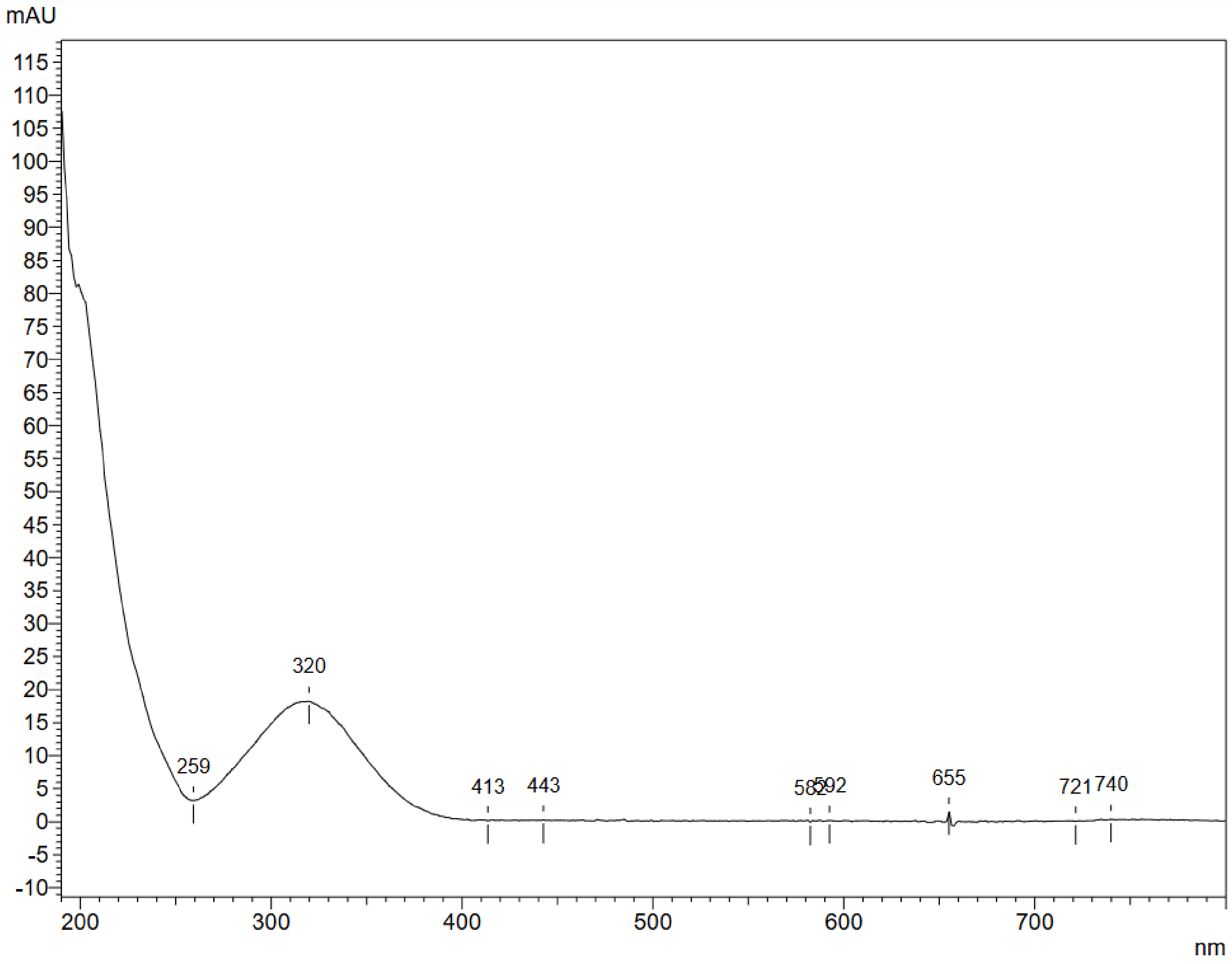

mAU
115
110
105
100
95
90
85
80
75
70
65
60
55
50
45
40
35
30
25
20
15
10
5
0
-5
-10
320
259
413
443
582
592
655
721
740
200
300
400
500
600
700
nm

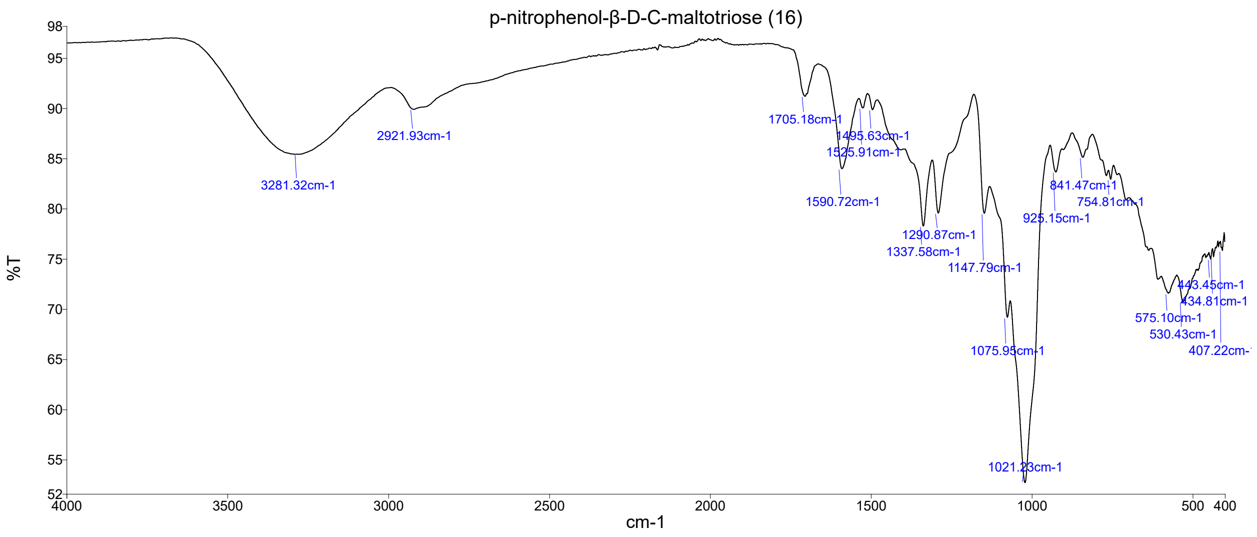

p-nitrophenol-β-D-C-maltotriose (16)
%T
3281.32cm-1
2921.93cm-1
1705.18cm-1
1495.63cm-1
1525.91cm-1
1590.72cm-1
1290.87cm-1
1337.58cm-1
1147.79cm-1
1075.95cm-1
1021.23cm-1
925.15cm-1
841.47cm-1
754.81cm-1
575.10cm-1
530.43cm-1
443.45cm-1
434.81cm-1
cm-1
4000
3500
3000
2500
2000
1500
1000
500
400

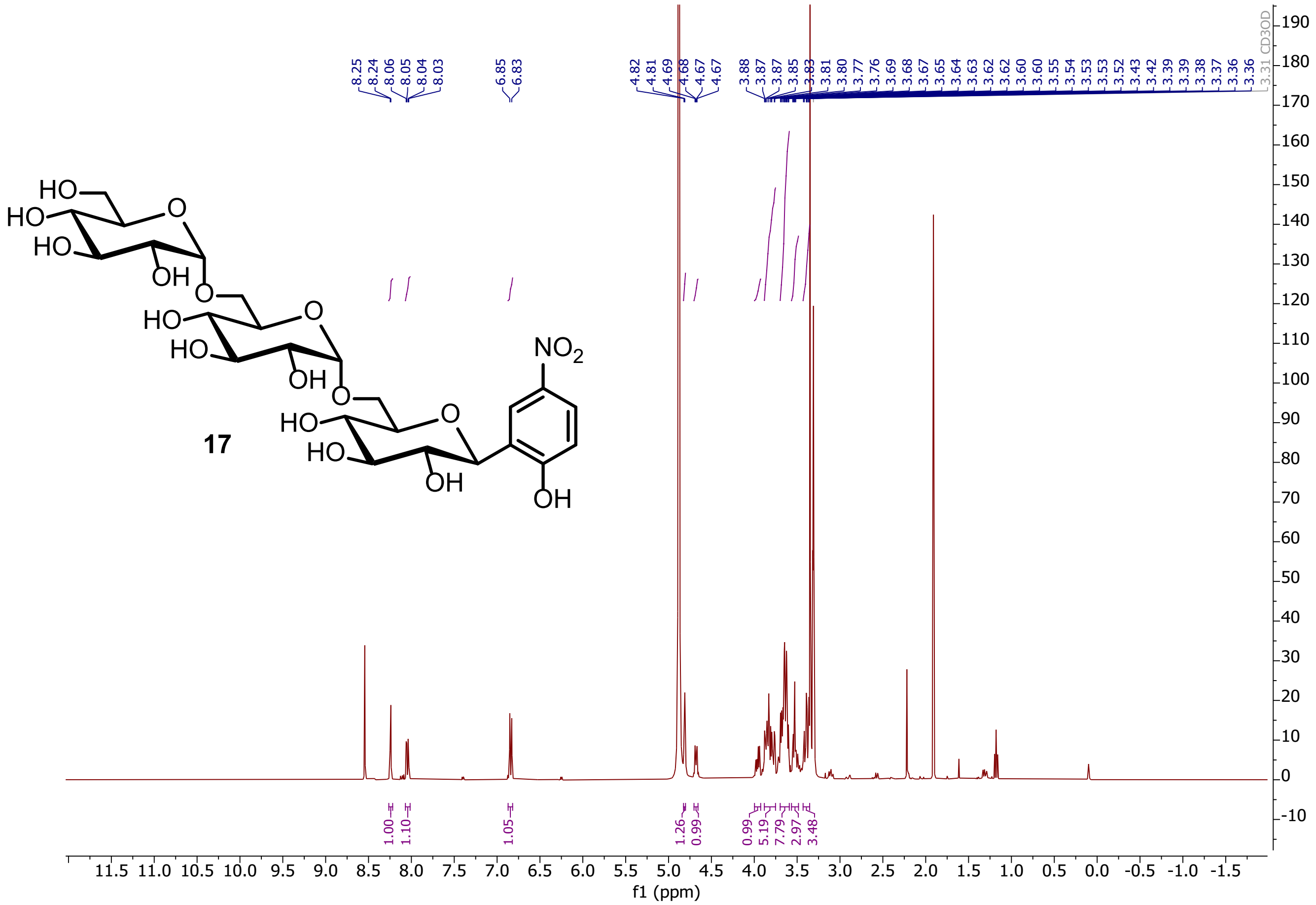
17
NO2
OH
8.25
8.24
8.06
8.05
8.04
8.03
6.85
6.83
4.82
4.81
4.69
4.68
4.67
4.67
3.88
3.87
3.87
3.85
3.83
3.81
3.80
3.77
3.76
3.69
3.68
3.67
3.65
3.64
3.63
3.62
3.62
3.60
3.55
3.54
3.53
3.53
3.52
3.43
3.42
3.39
3.39
3.38
3.37
3.36
3.36
3.31 CD3OD
1.00
1.10
1.05
1.26
0.99
0.99
5.19
7.79
2.97
3.48
f1 (ppm)

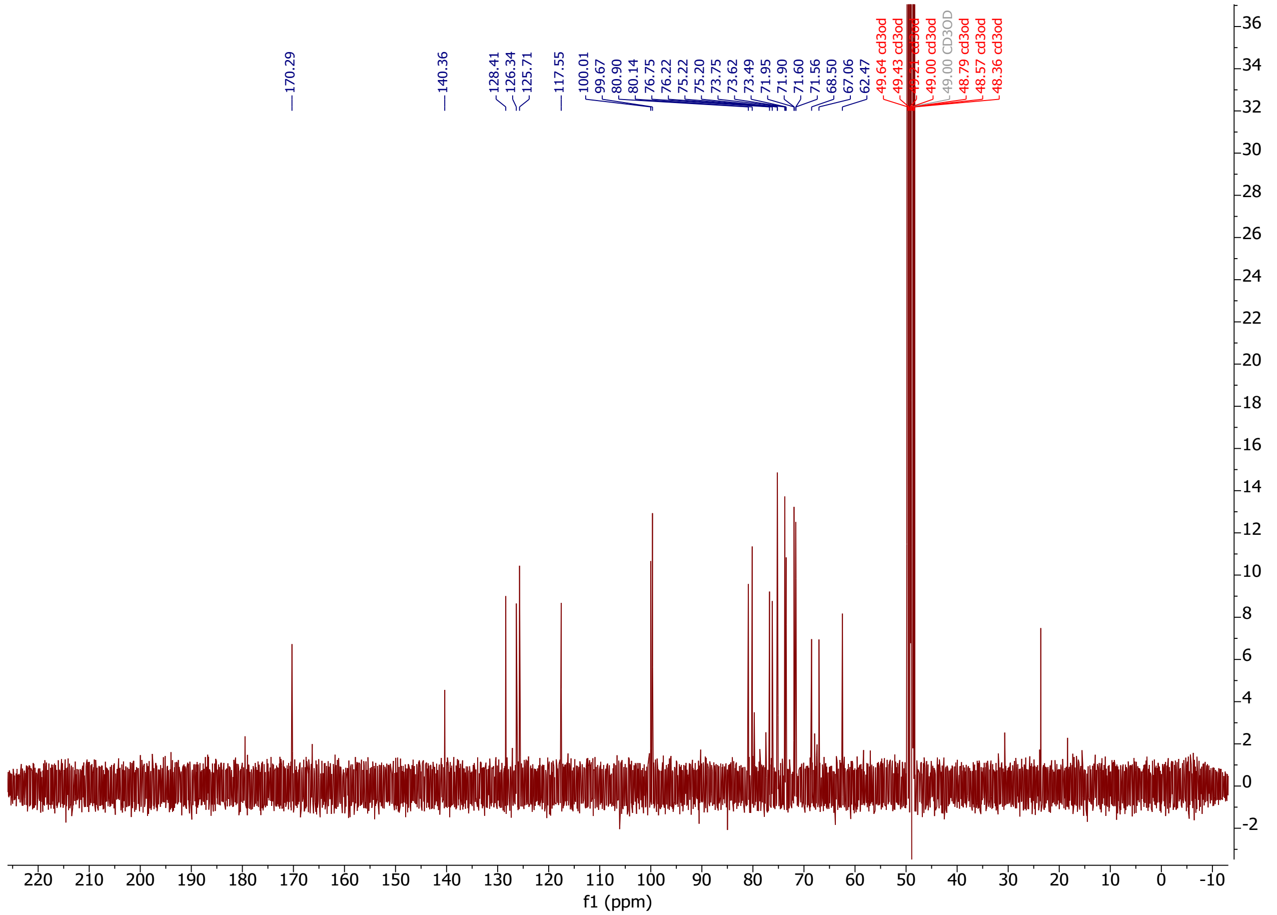
170.29
140.36
128.41
126.34
125.71
117.55
100.01
99.67
80.90
80.14
76.75
76.22
75.22
75.20
73.75
73.62
73.49
71.95
71.90
71.60
71.56
68.50
67.06
62.47
49.64 cd3od
49.43 cd3od
49.00 cd3od
49.00 CD3OD
48.79 cd3od
48.57 cd3od
48.36 cd3od
f1 (ppm)

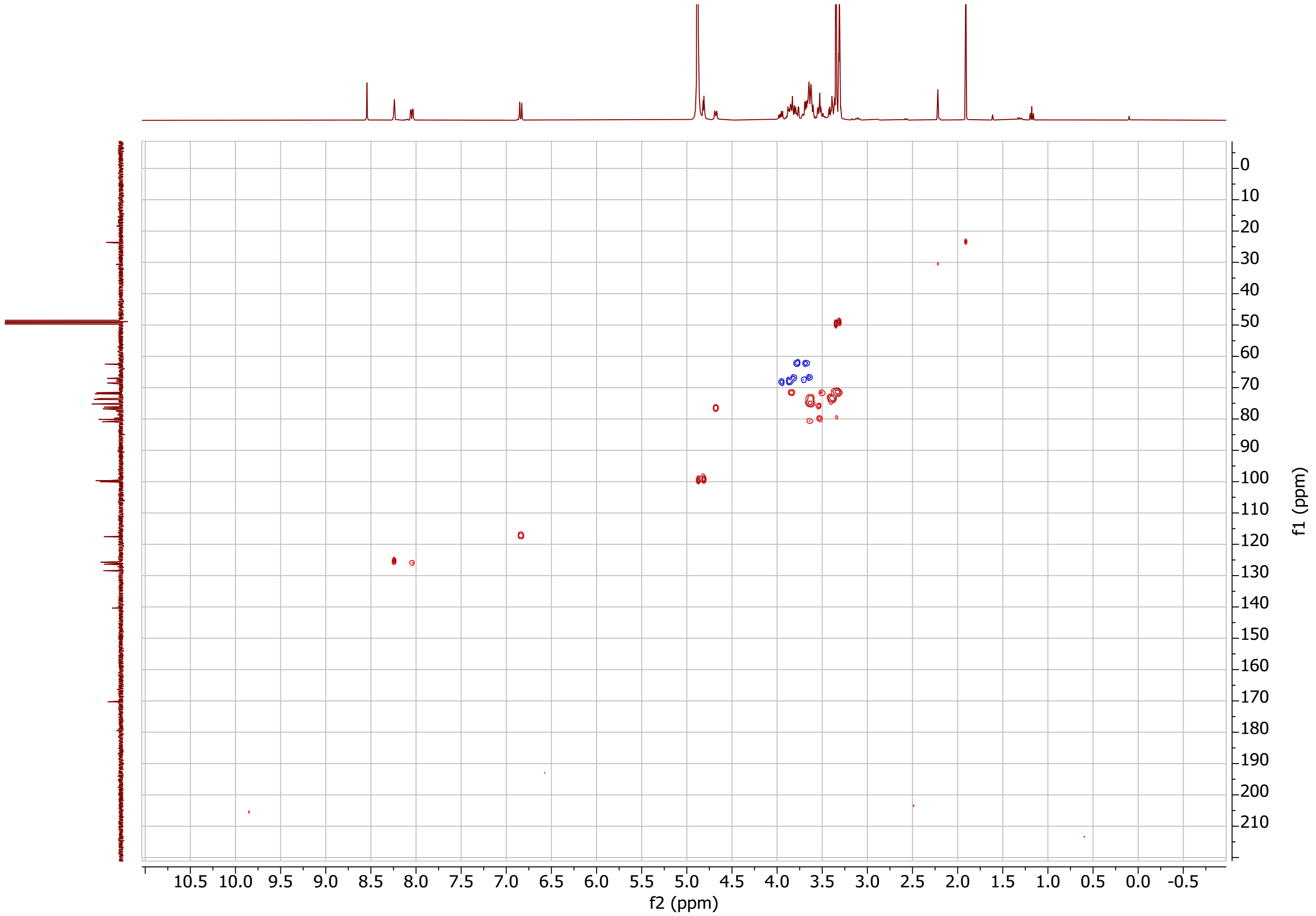
f1 (ppm)
f2 (ppm)
10.5 10.0 9.5 9.0 8.5 8.0 7.5 7.0 6.5 6.0 5.5 5.0 4.5 4.0 3.5 3.0 2.5 2.0 1.5 1.0 0.5 0.0 -0.5
0 10 20 30 40 50 60 70 80 90 100 110 120 130 140 150 160 170 180 190 200 210

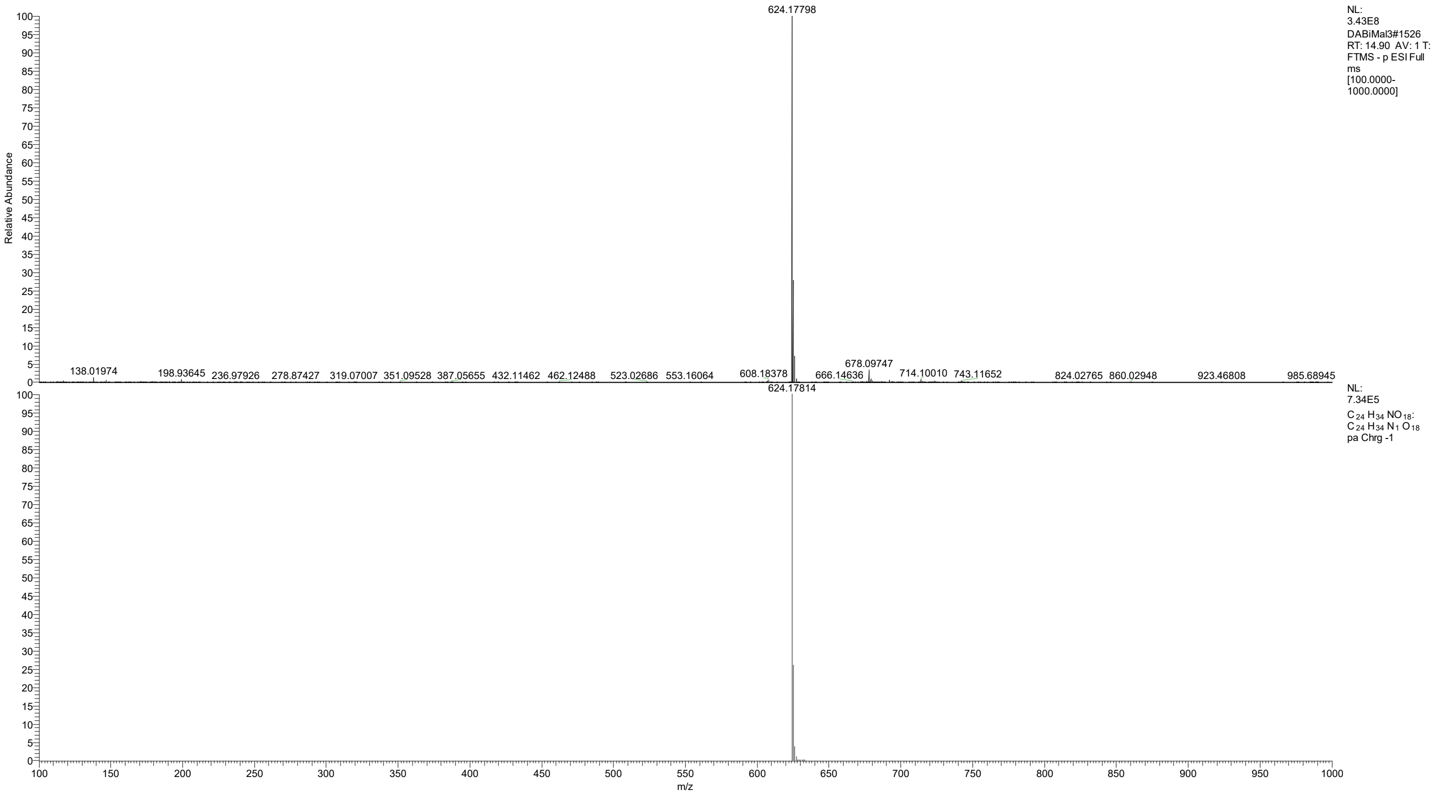
624.17798
NL:
3.43E8
DABiMal3#1526
RT: 14.90 AV: 1 T:
FTMS - p ESI Full
ms
[100.0000-
1000.0000]
Relative Abundance
138.01974
198.93645
236.97926
278.87427
319.07007
351.09528
387.05655
432.11462
462.12488
523.02686
553.16064
608.18378
666.14636
678.09747
714.10010
743.11652
824.02765
860.02948
923.46808
985.68945
624.17814
NL:
7.34E5
C 24 H 34 NO 18:
C 24 H 34 N 1 O 18
pa Chrg -1
m/z

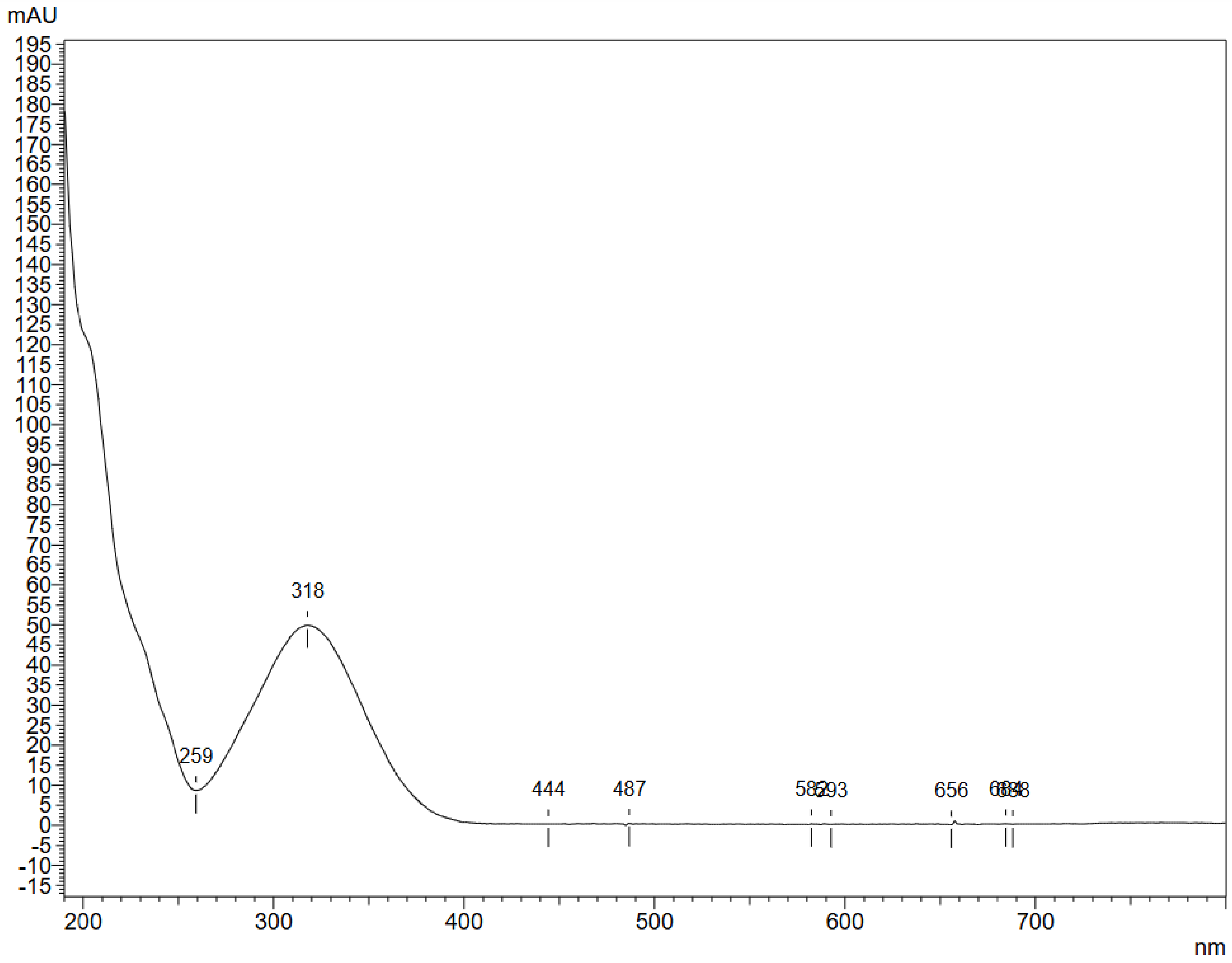
mAU
318
259
444
487
582
593
656
nm

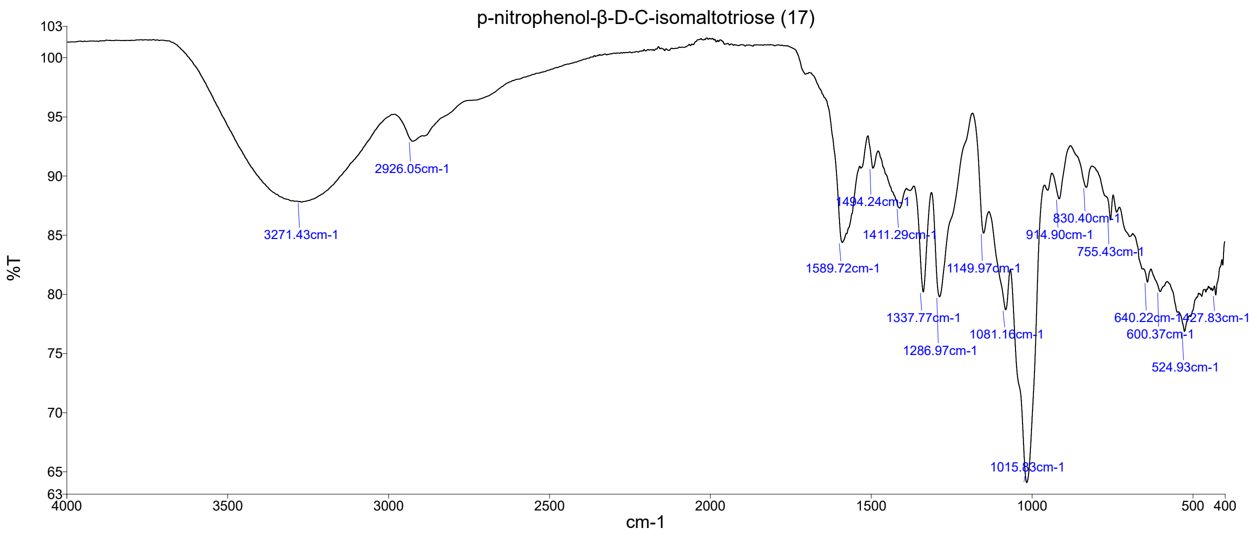
p-nitrophenol-β-D-C-isomaltotriose (17)
%T
cm-1
3271.43cm-1
2926.05cm-1
1589.72cm-1
1494.24cm-1
1411.29cm-1
1337.77cm-1
1286.97cm-1
1149.97cm-1
1081.16cm-1
1015.83cm-1
914.90cm-1
830.40cm-1
755.43cm-1
640.22cm-1
600.37cm-1
524.93cm-1
427.83cm-1

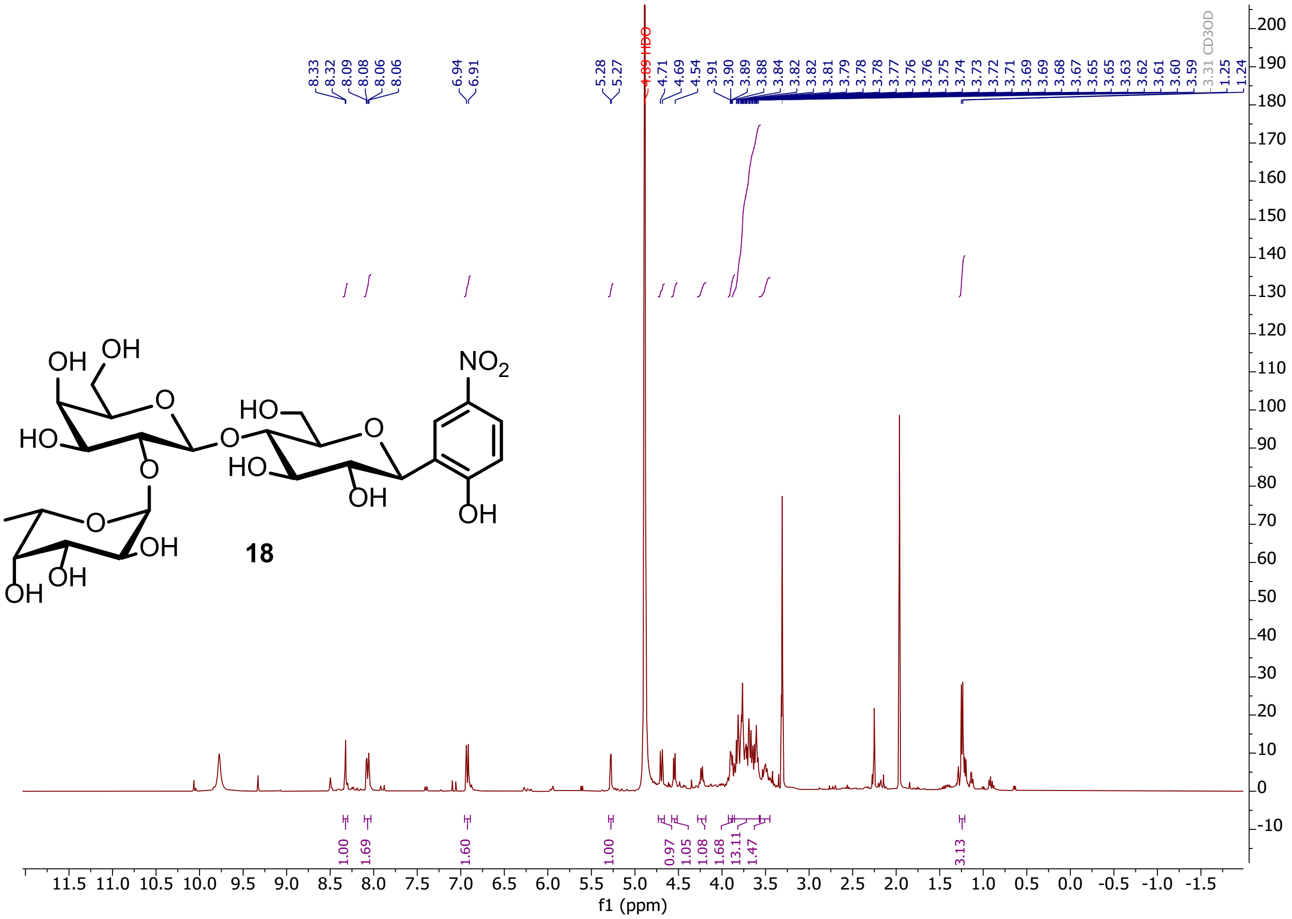
8.33 8.32 8.09 8.08 8.06 8.06
6.94 6.91
5.28 5.27
4.89 HDO
4.71 4.69 4.54
3.91 3.90 3.89 3.88 3.84 3.82 3.82 3.81 3.79 3.78 3.78 3.77 3.76 3.76 3.75 3.74 3.73 3.72 3.71 3.69 3.69 3.68 3.67 3.65 3.65 3.63 3.62 3.61 3.60 3.59
3.31 CD3OD
1.25 1.24
1.00 1.69 1.60 1.00 0.97 1.05 1.08 1.68 13.11 1.47 3.13
f1 (ppm)
NO2
18

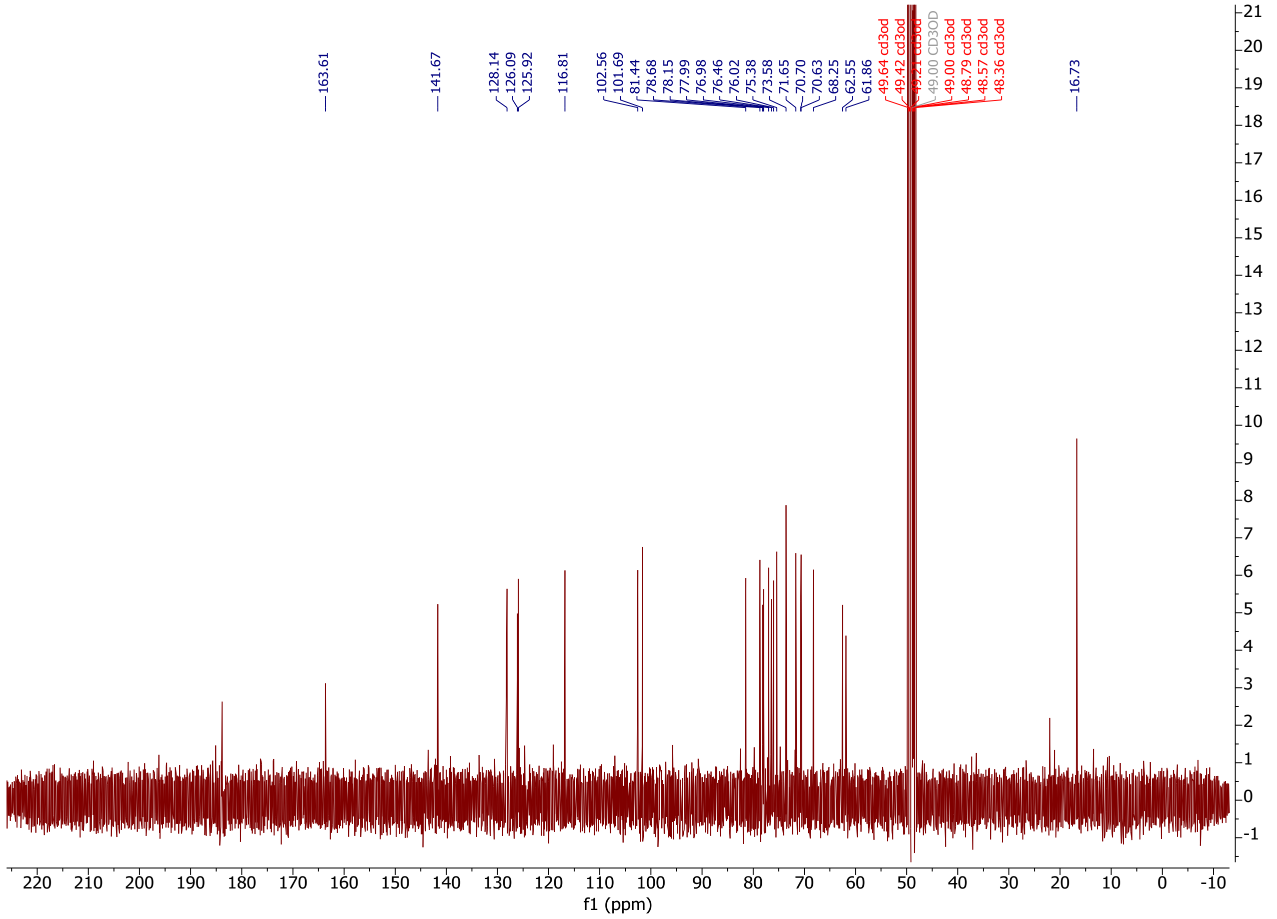
163.61
141.67
128.14 126.09 125.92
116.81
102.56 101.69 81.44 78.68 78.15 77.99 76.98 76.46 76.02 75.38 73.58 71.65 70.70 70.63 68.25 62.55 61.86
49.64 cd3od 49.42 cd3od 49.21 cd3od 49.00 CD3OD 49.00 cd3od 48.79 cd3od 48.57 cd3od 48.36 cd3od
16.73
f1 (ppm)

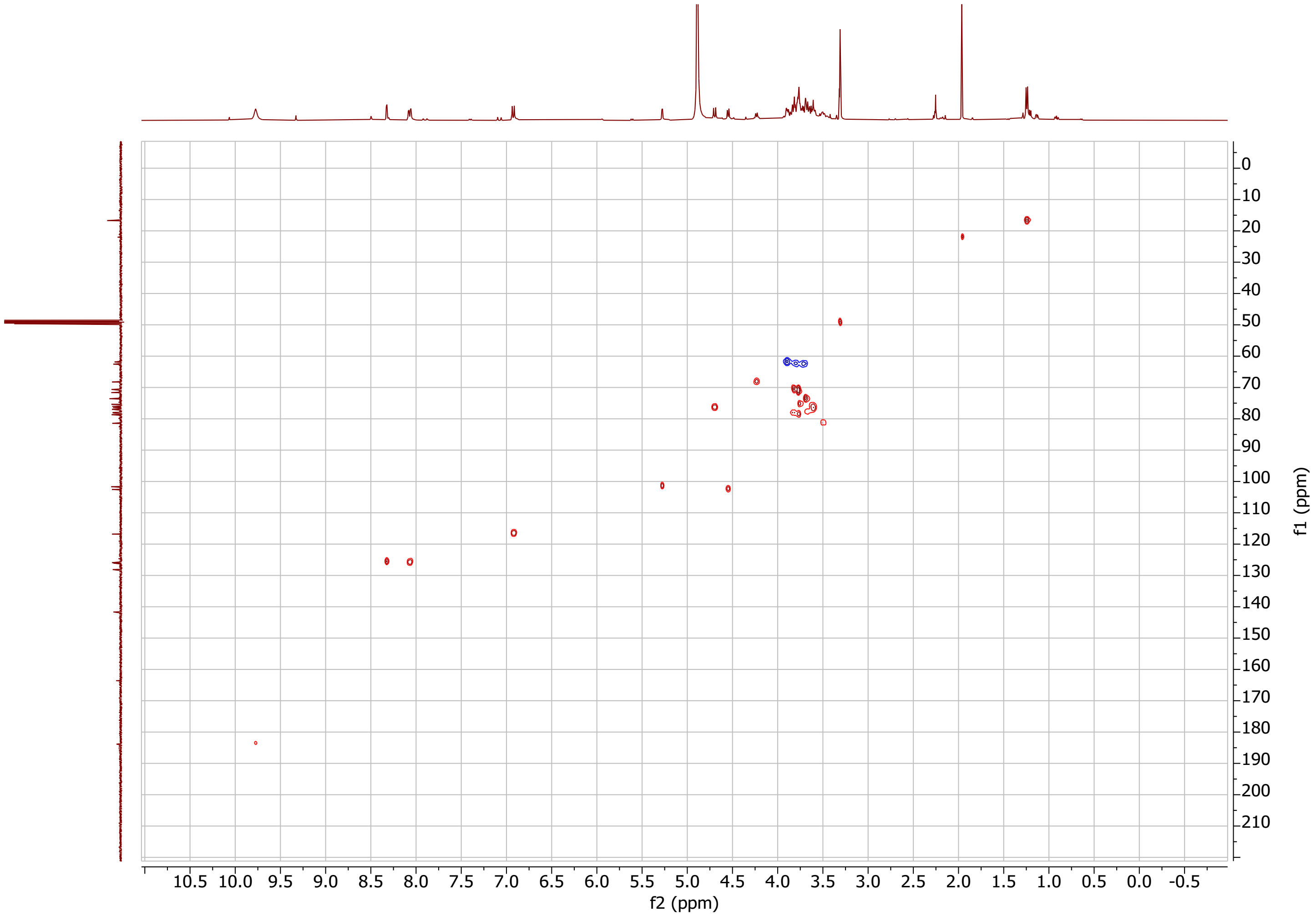
f2 (ppm)
f1 (ppm)

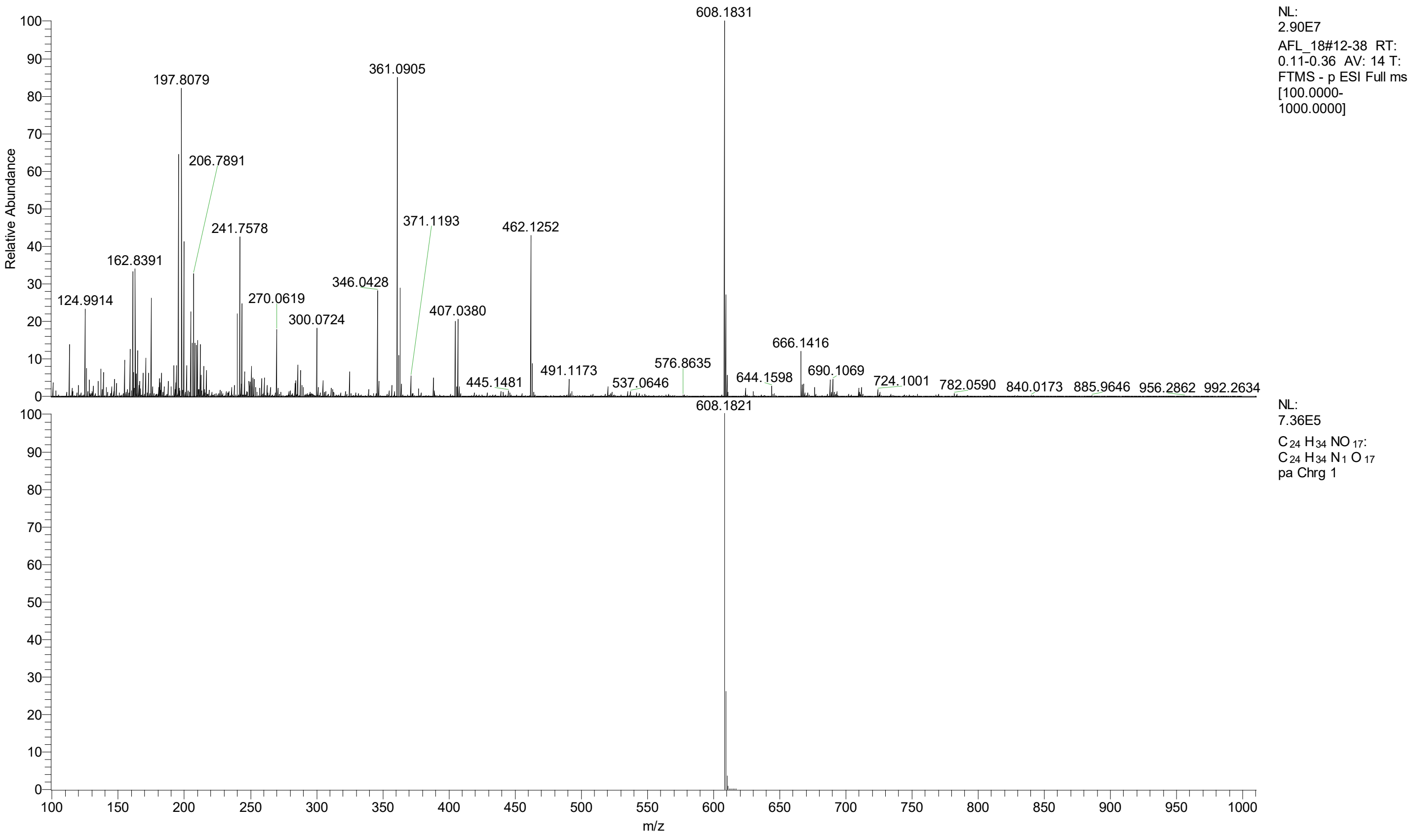
608.1831
197.8079
361.0905
206.7891
241.7578
371.1193
462.1252
162.8391
346.0428
124.9914
270.0619
300.0724
407.0380
666.1416
576.8635
491.1173
690.1069
644.1598
445.1481
537.0646
724.1001
782.0590
840.0173
885.9646
956.2862
992.2634
NL: 2.90E7
AFL_18#12-38 RT: 0.11-0.36 AV: 14 T: FTMS - p ESI Full ms [100.0000-1000.0000]
608.1821
NL: 7.36E5
C 24 H 34 NO 17: C 24 H 34 N 1 O 17 pa Chrg 1
Relative Abundance
m/z

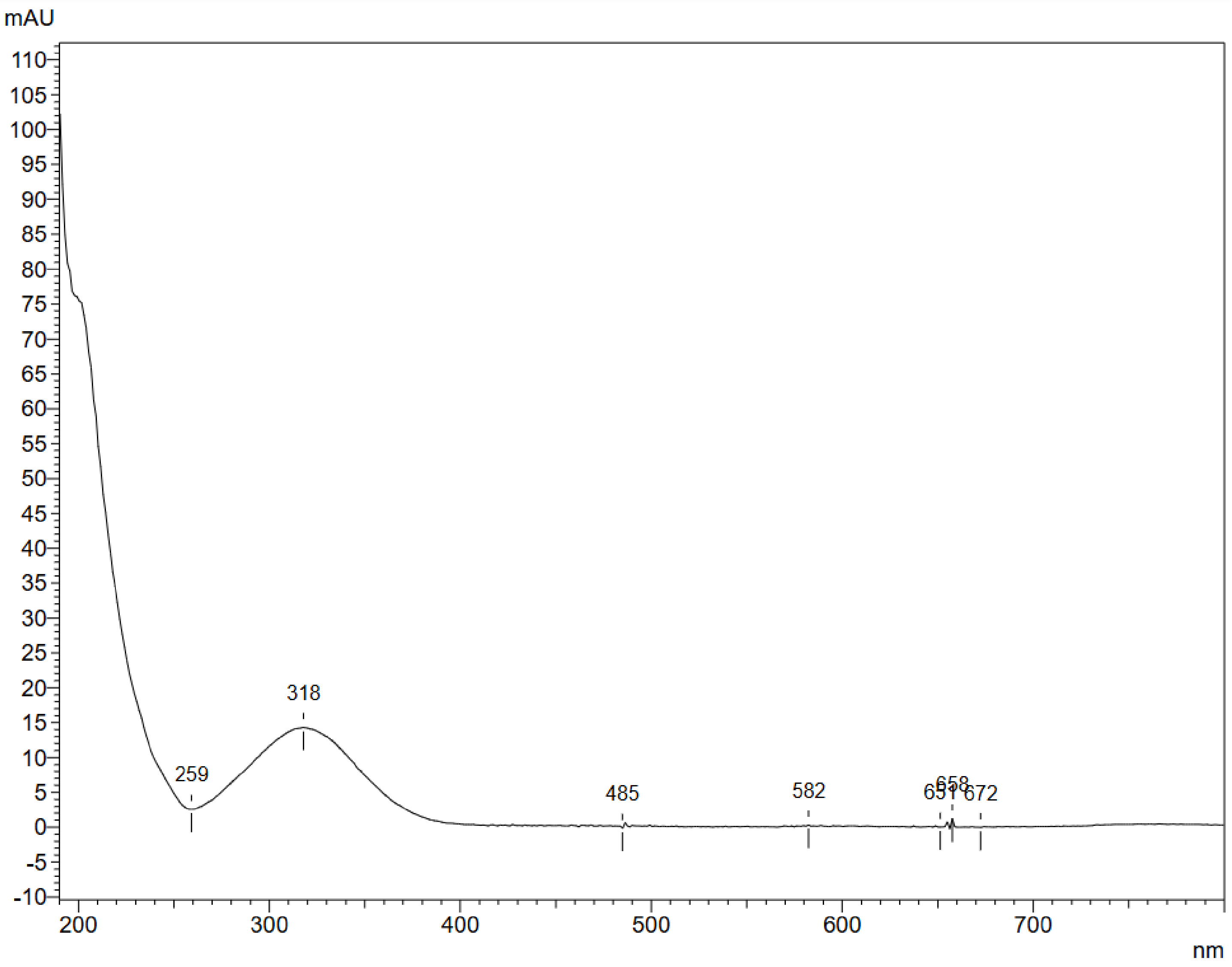

p-nitrophenol-β-C-2'-fucosyllactoside (18)

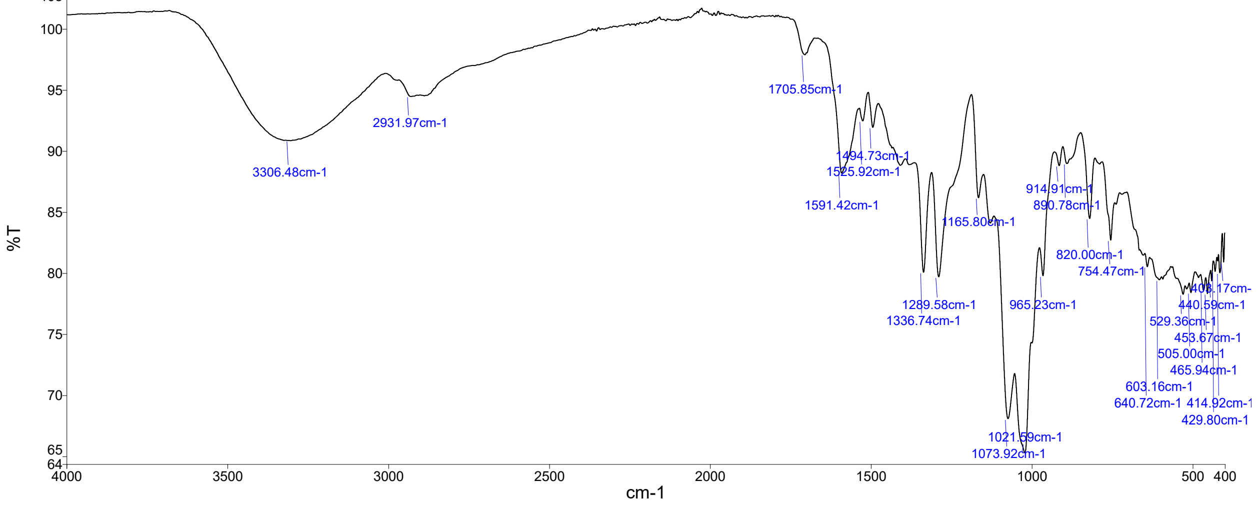

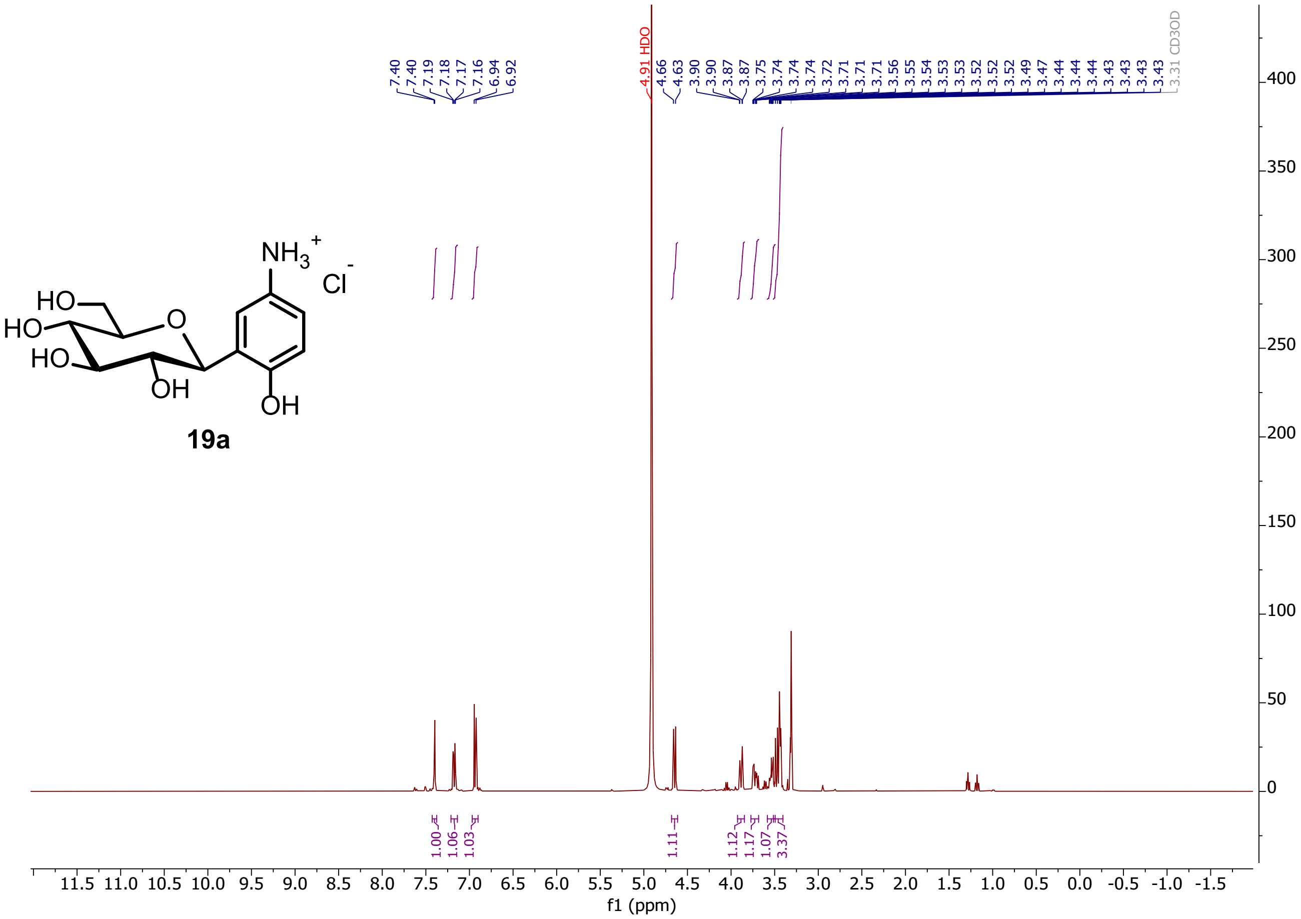
NH3
Cl
HO
HO
HO
O
OH
OH
19a
7.40
7.40
7.19
7.18
7.17
7.16
6.94
6.92
4.91 HDO
4.66
4.63
3.90
3.90
3.87
3.87
3.75
3.74
3.74
3.74
3.72
3.71
3.71
3.71
3.56
3.55
3.54
3.53
3.53
3.52
3.52
3.52
3.49
3.47
3.44
3.44
3.44
3.43
3.43
3.43
3.43
3.31 CD3OD
1.00
1.06
1.03
1.11
1.12
1.17
1.07
3.37
f1 (ppm)

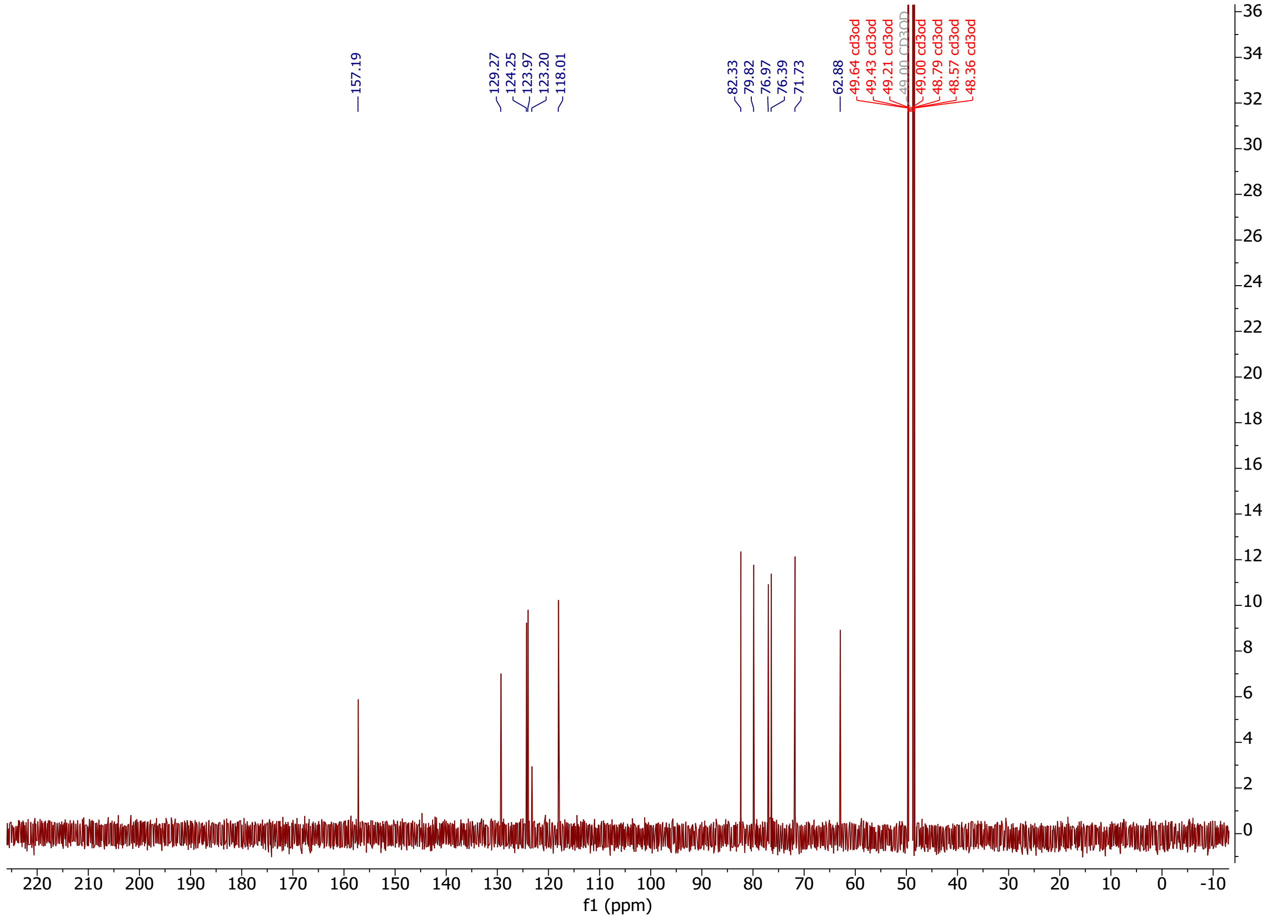
157.19
129.27
124.25
123.97
123.20
118.01
82.33
79.82
76.97
76.39
71.73
62.88
49.64 cd3od
49.43 cd3od
49.21 cd3od
49.00 CD3OD
49.00 cd3od
48.79 cd3od
48.57 cd3od
48.36 cd3od
f1 (ppm)

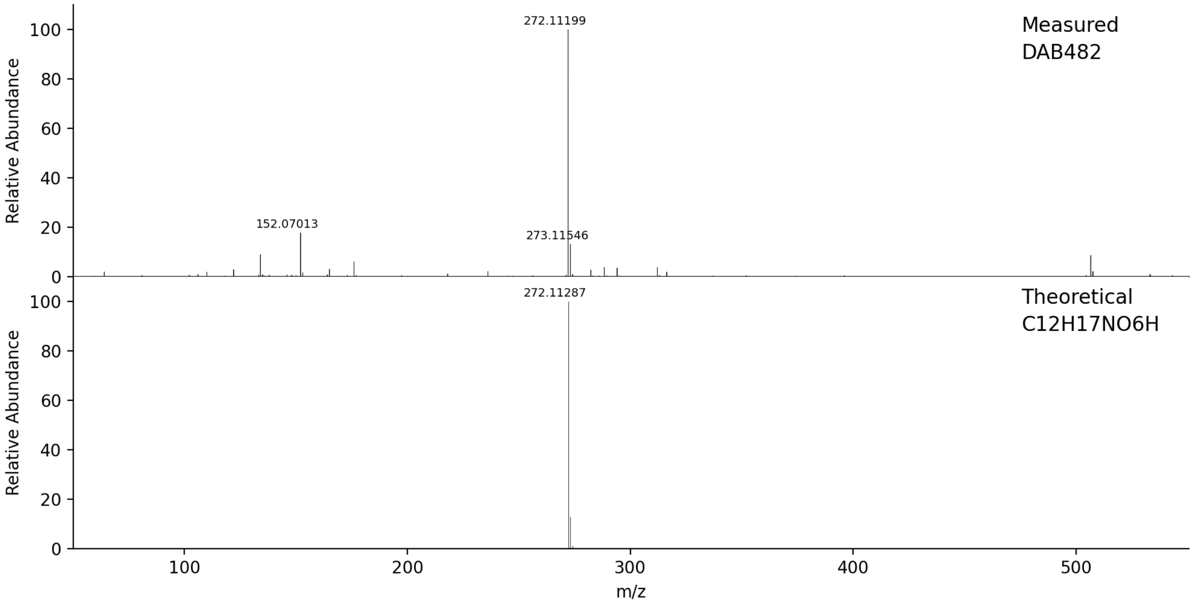

272.11199
Measured
DAB482
152.07013
273.11546
Relative Abundance
100
80
60
40
20
0
272.11287
Theoretical
C12H17NO6H
Relative Abundance
100
80
60
40
20
0
100
200
300
400
500
m/z

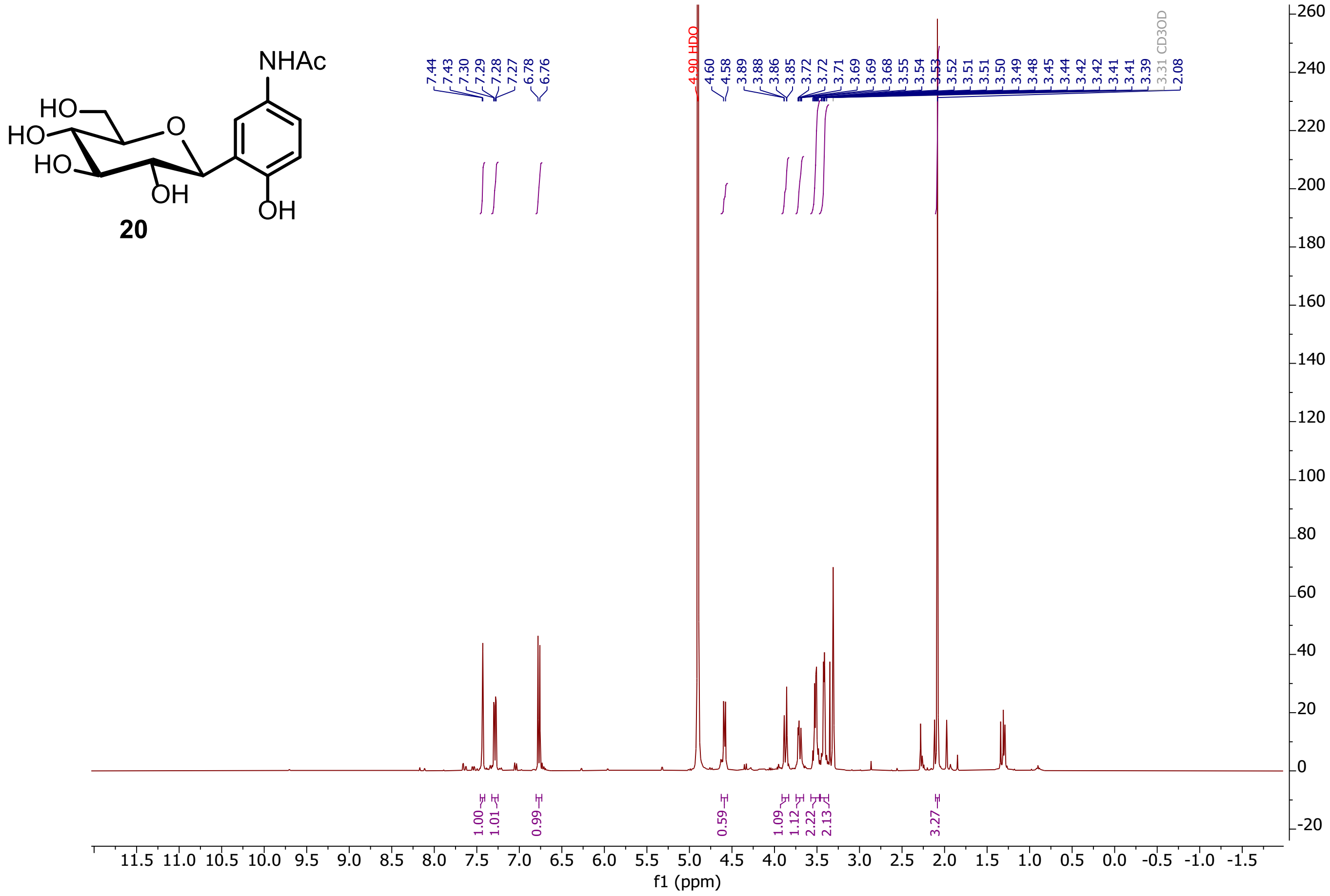
NHAc
HO
HO
HO
O
OH
OH
20
7.44
7.43
7.30
7.29
7.28
7.27
6.78
6.76
4.90 HDO
4.60
4.58
3.89
3.88
3.86
3.85
3.72
3.72
3.71
3.69
3.69
3.68
3.55
3.54
3.53
3.52
3.51
3.51
3.50
3.49
3.48
3.45
3.44
3.42
3.42
3.41
3.41
3.39
3.31 CD3OD
2.08
1.00
1.01
0.99
0.59
1.09
1.12
2.22
2.13
3.27
f1 (ppm)

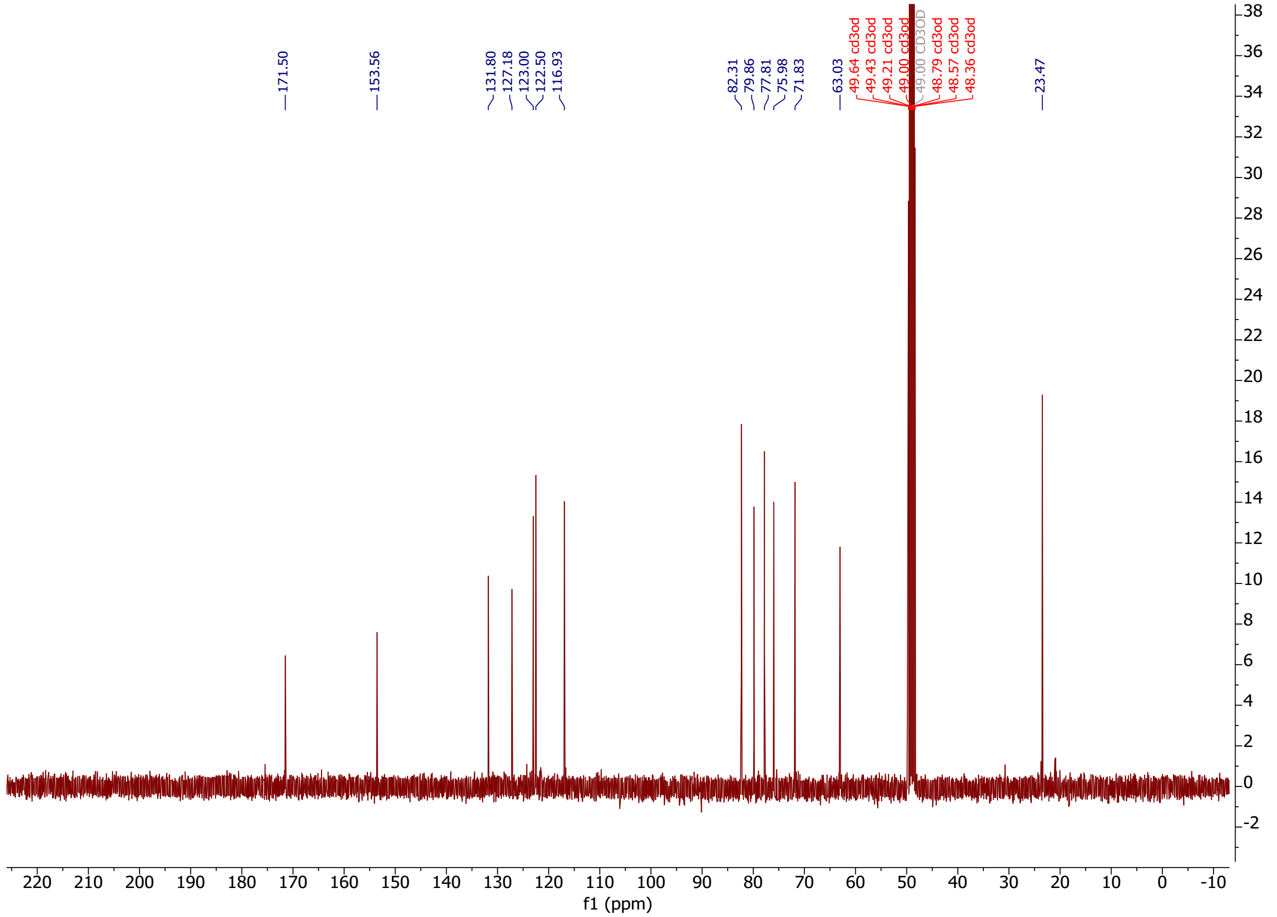
171.50
153.56
131.80
127.18
123.00
122.50
116.93
82.31
79.86
77.81
75.98
71.83
63.03
49.64 cd3od
49.43 cd3od
49.21 cd3od
49.00 cd3od
49.00 CD3OD
48.79 cd3od
48.57 cd3od
48.36 cd3od
23.47
f1 (ppm)

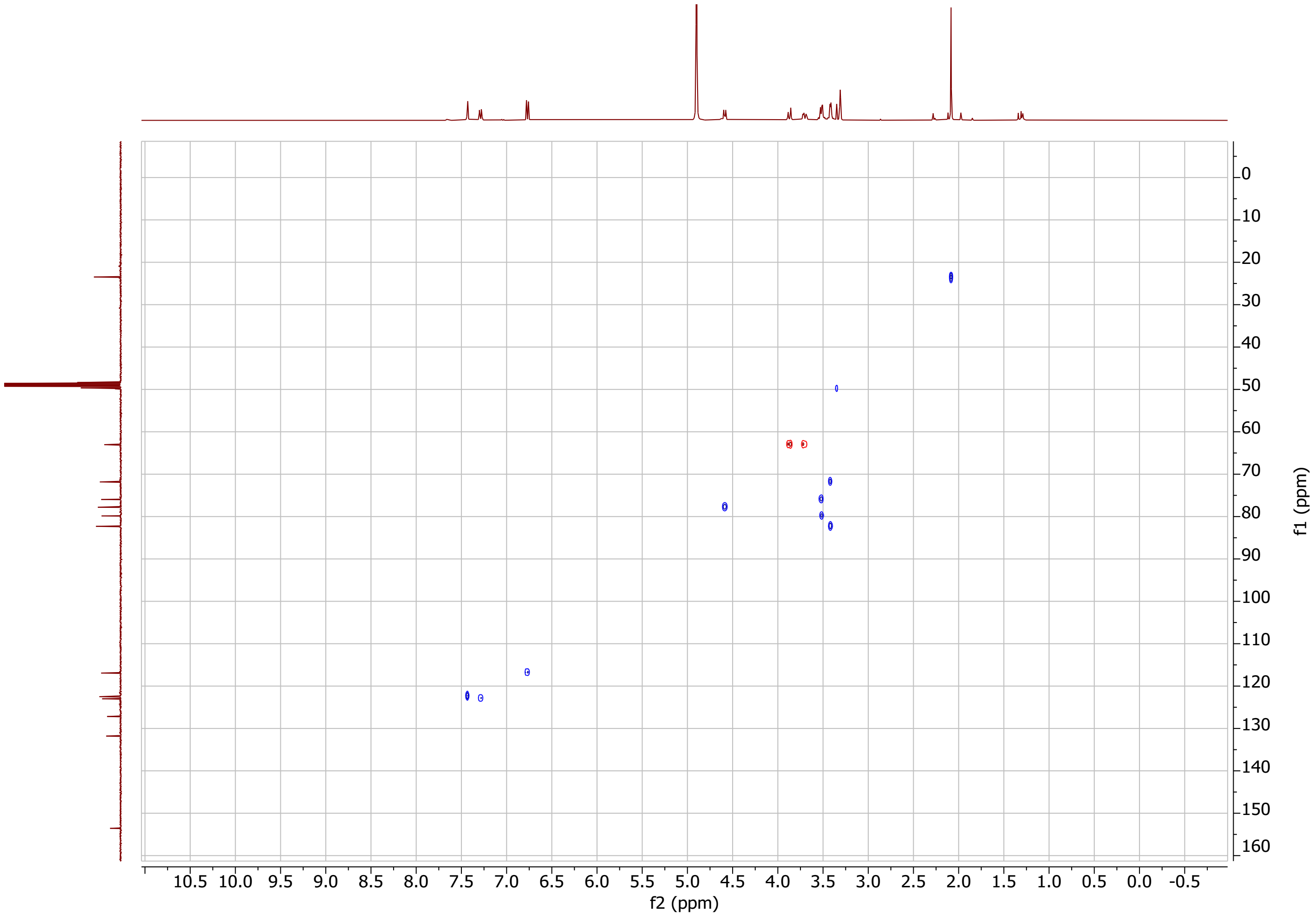
f2 (ppm)
f1 (ppm)

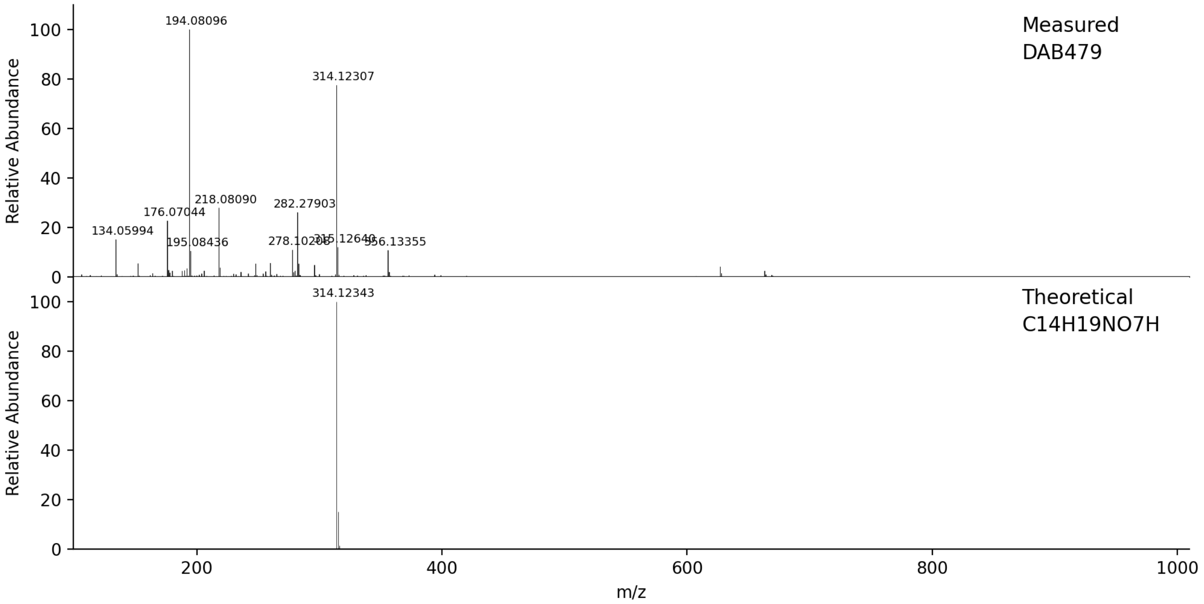
Measured
DAB479
194.08096
314.12307
218.08090
282.27903
176.07044
134.05994
195.08436
278.10208
315.12640
356.13355
Theoretical
C14H19NO7H
314.12343
Relative Abundance
m/z

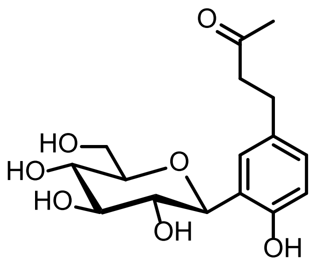
O
HO
HO
O
HO
OH
OH

21

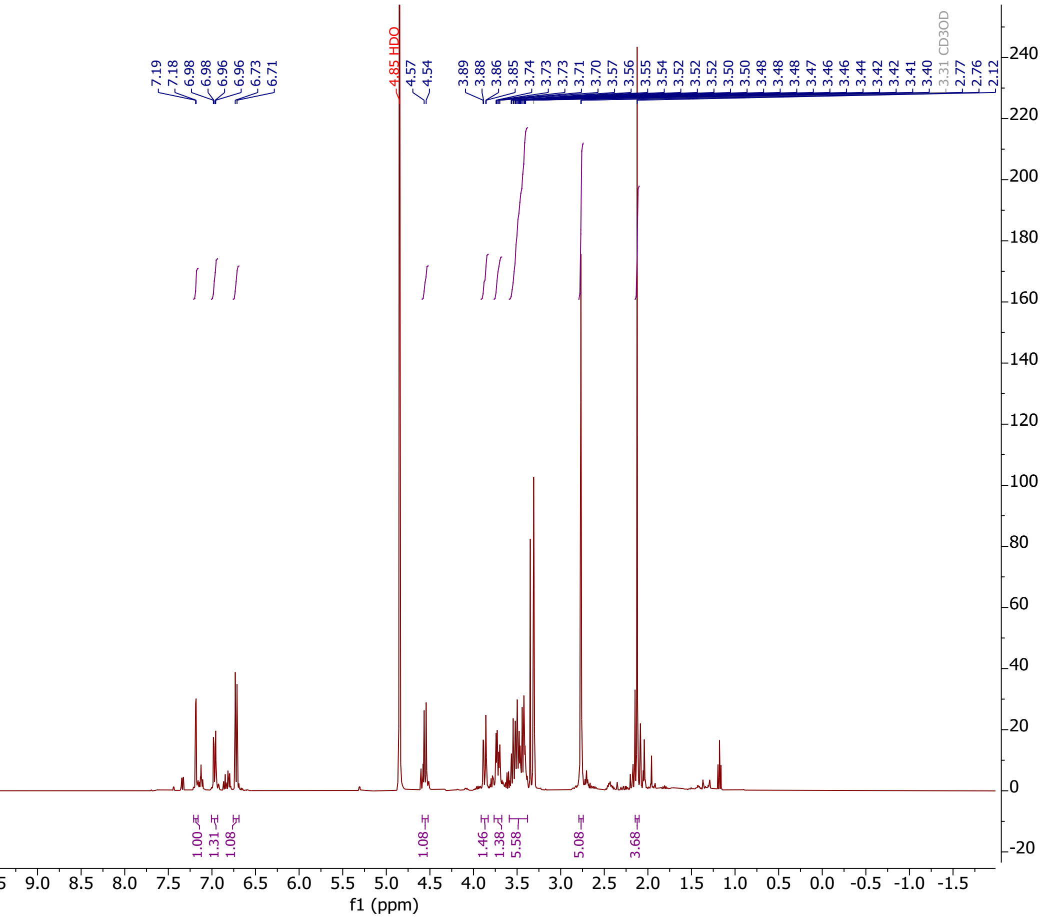
7.19
7.18
6.98
6.98
6.96
6.96
6.73
6.71
4.85 HDO
4.57
4.54
3.89
3.88
3.86
3.85
3.74
3.73
3.73
3.71
3.70
3.57
3.56
3.55
3.54
3.52
3.52
3.52
3.50
3.50
3.48
3.48
3.48
3.47
3.46
3.46
3.44
3.42
3.42
3.41
3.40
3.31 CD3OD
2.77
2.76
2.12
1.00
1.31
1.08
1.08
1.46
1.38
5.58
5.08
3.68
f1 (ppm)

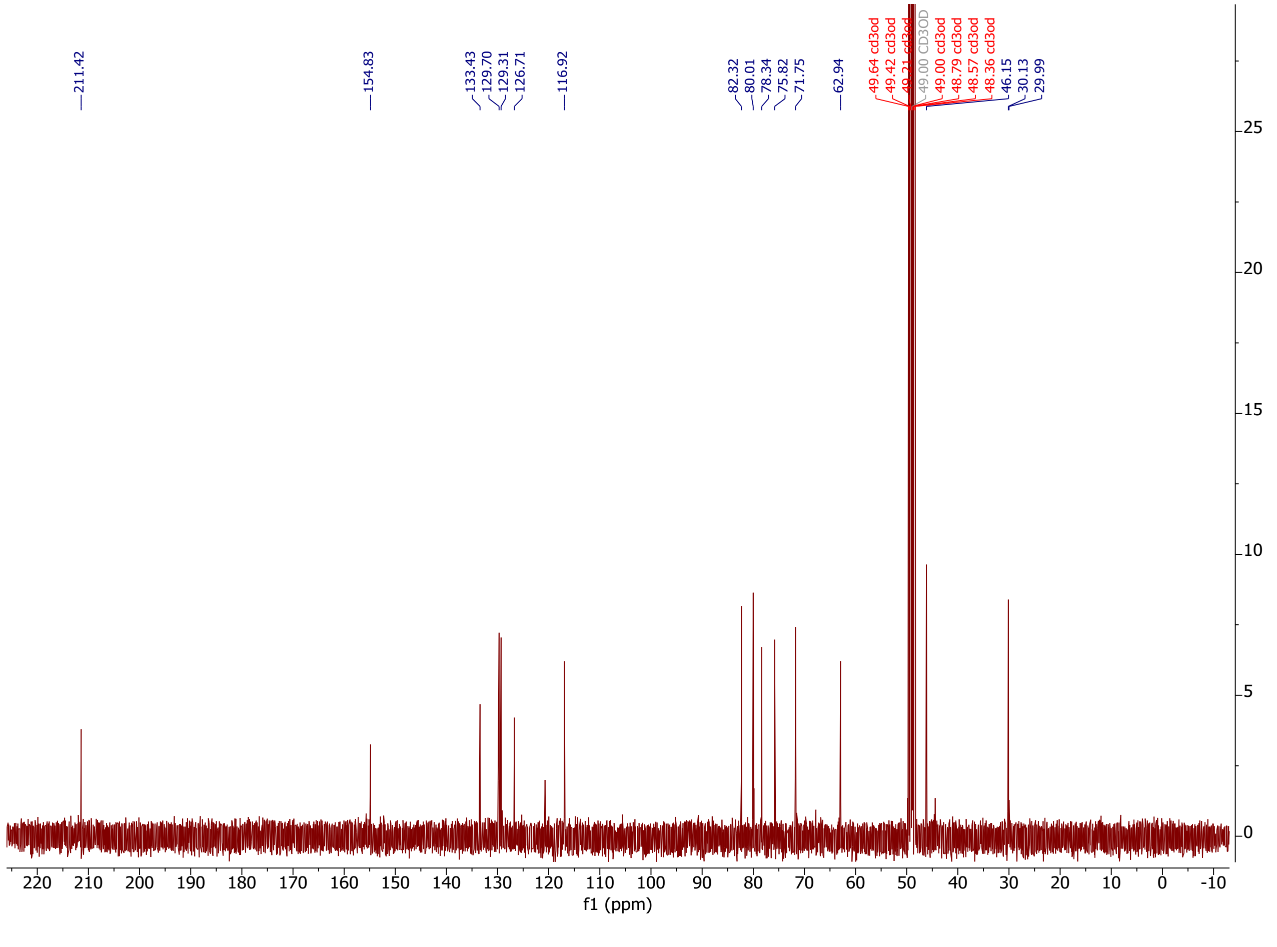
211.42
154.83
133.43
129.70
129.31
126.71
116.92
82.32
80.01
78.34
75.82
71.75
62.94
49.64 cd3od
49.42 cd3od
49.21 cd3od
49.00 CD3OD
49.00 cd3od
48.79 cd3od
48.57 cd3od
48.36 cd3od
46.15
30.13
29.99
f1 (ppm)

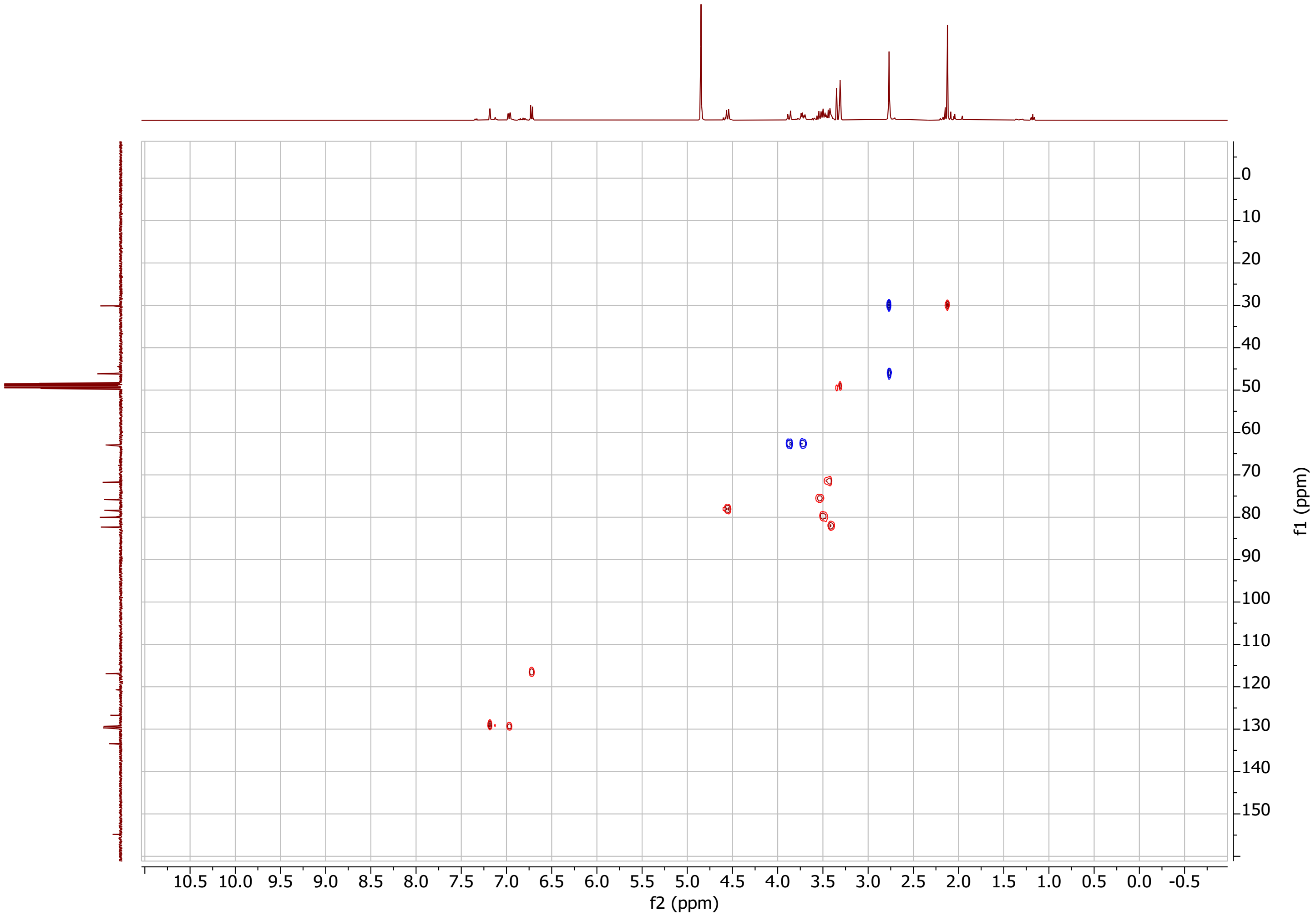
f1 (ppm)
f2 (ppm)

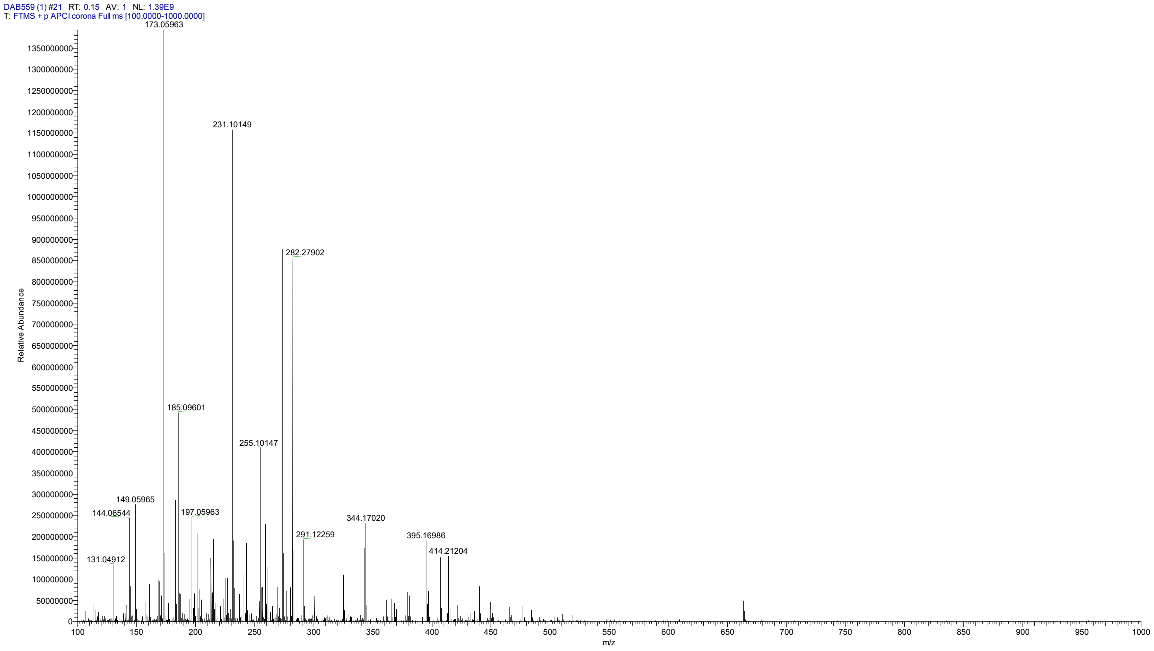
DAB559 (1) #21 RT: 0.15 AV: 1 NL: 1.39E9
T: FTMS + p APCI corona Full ms [100.0000-1000.0000]
173.05963
231.10149
282.27902
185.09601
255.10147
149.05965
144.06544
197.05963
344.17020
291.12259
395.16986
414.21204
131.04912
Relative Abundance
m/z

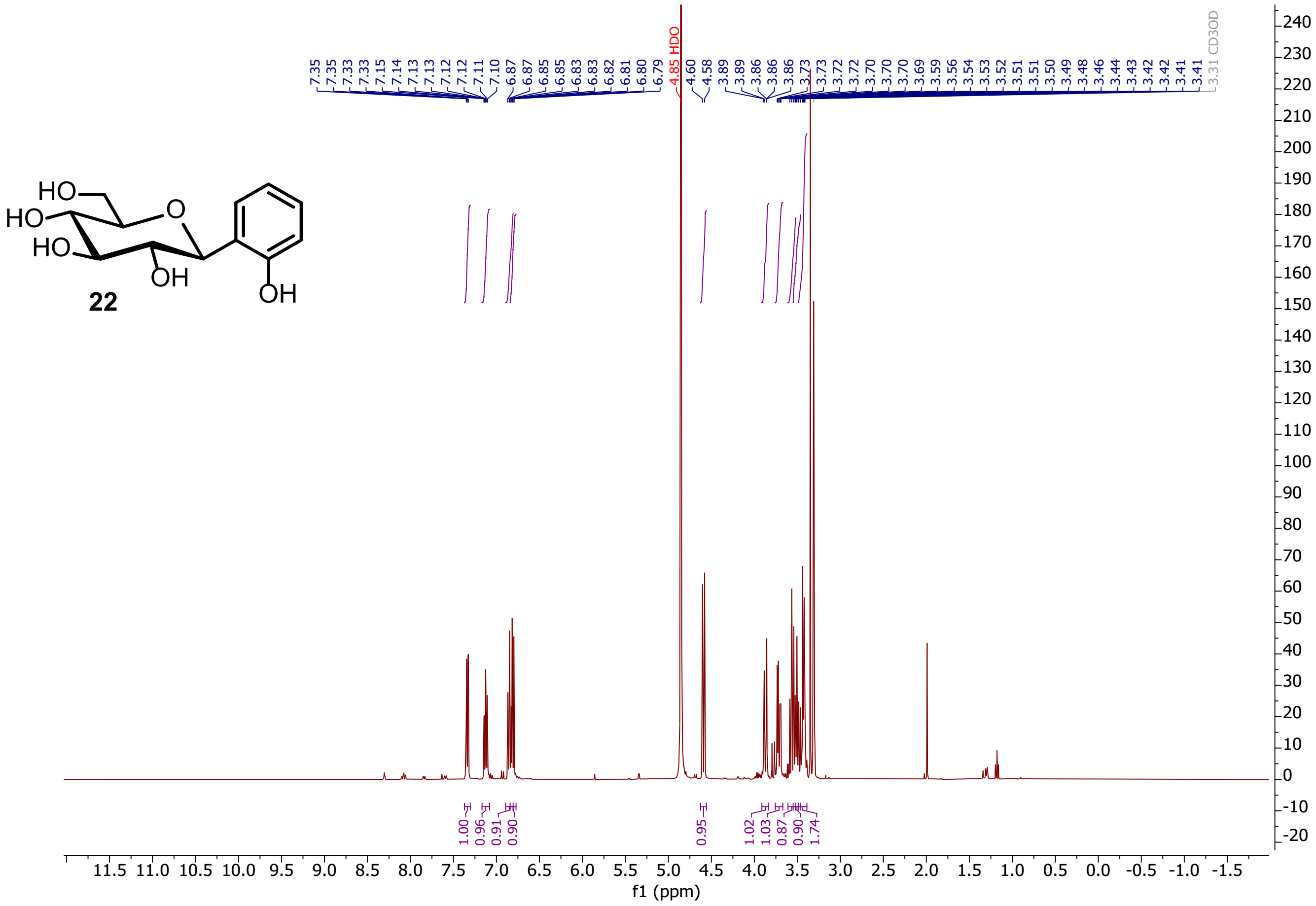
HO
HO
HO
O
OH
OH
22
7.35
7.35
7.33
7.33
7.15
7.14
7.13
7.13
7.12
7.11
7.10
6.87
6.87
6.85
6.85
6.83
6.83
6.82
6.81
6.80
6.79
4.85 HDO
4.60
4.58
3.89
3.89
3.86
3.86
3.73
3.73
3.72
3.72
3.70
3.70
3.70
3.69
3.59
3.56
3.54
3.53
3.52
3.51
3.51
3.50
3.49
3.48
3.46
3.44
3.43
3.42
3.42
3.41
3.31 CD3OD
1.00
0.96
0.91
0.90
0.95
1.02
1.03
0.87
0.90
1.74
f1 (ppm)

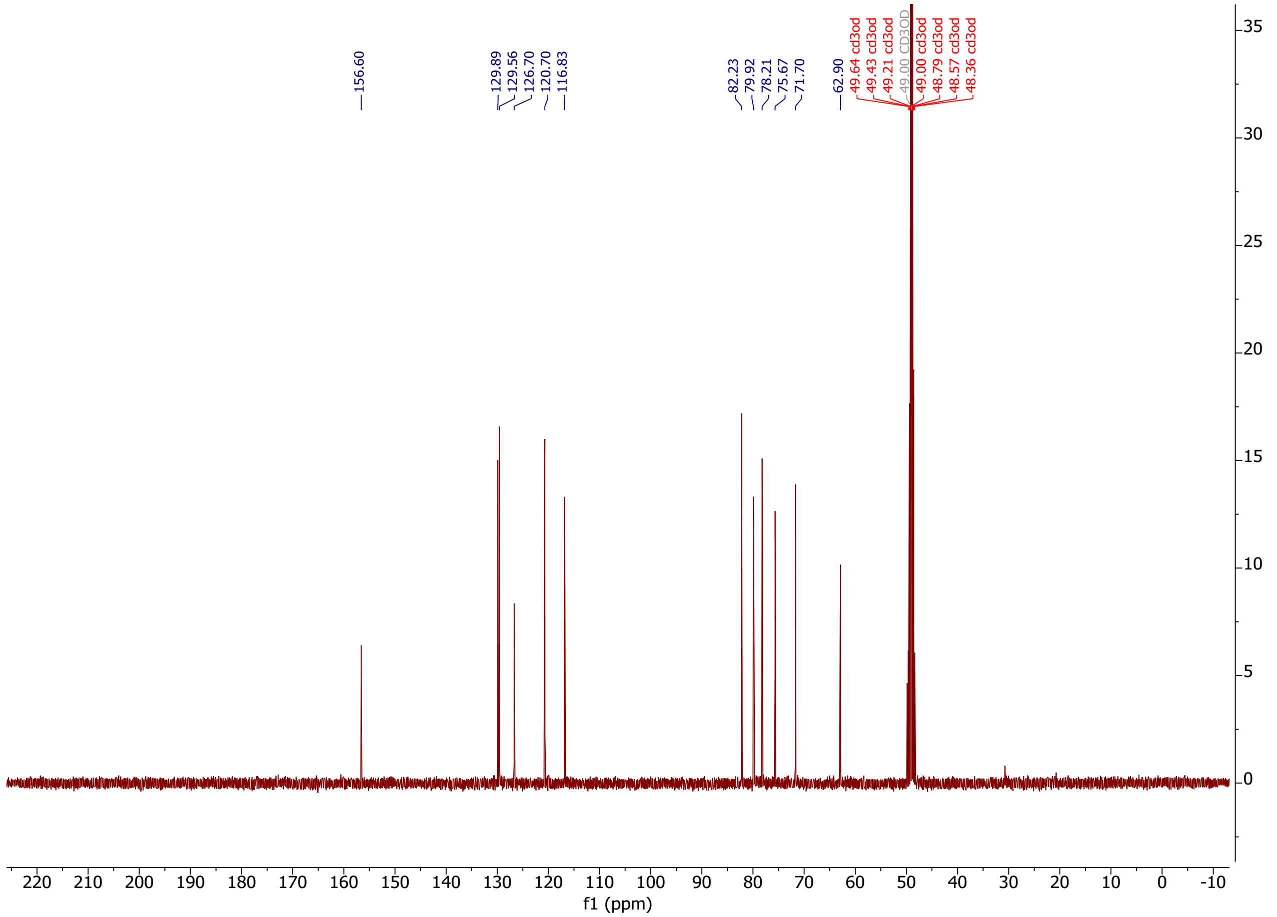
156.60
129.89
129.56
126.70
120.70
116.83
82.23
79.92
78.21
75.67
71.70
62.90
49.64 cd3od
49.43 cd3od
49.21 cd3od
49.00 CD3OD
49.00 cd3od
48.79 cd3od
48.57 cd3od
48.36 cd3od
f1 (ppm)

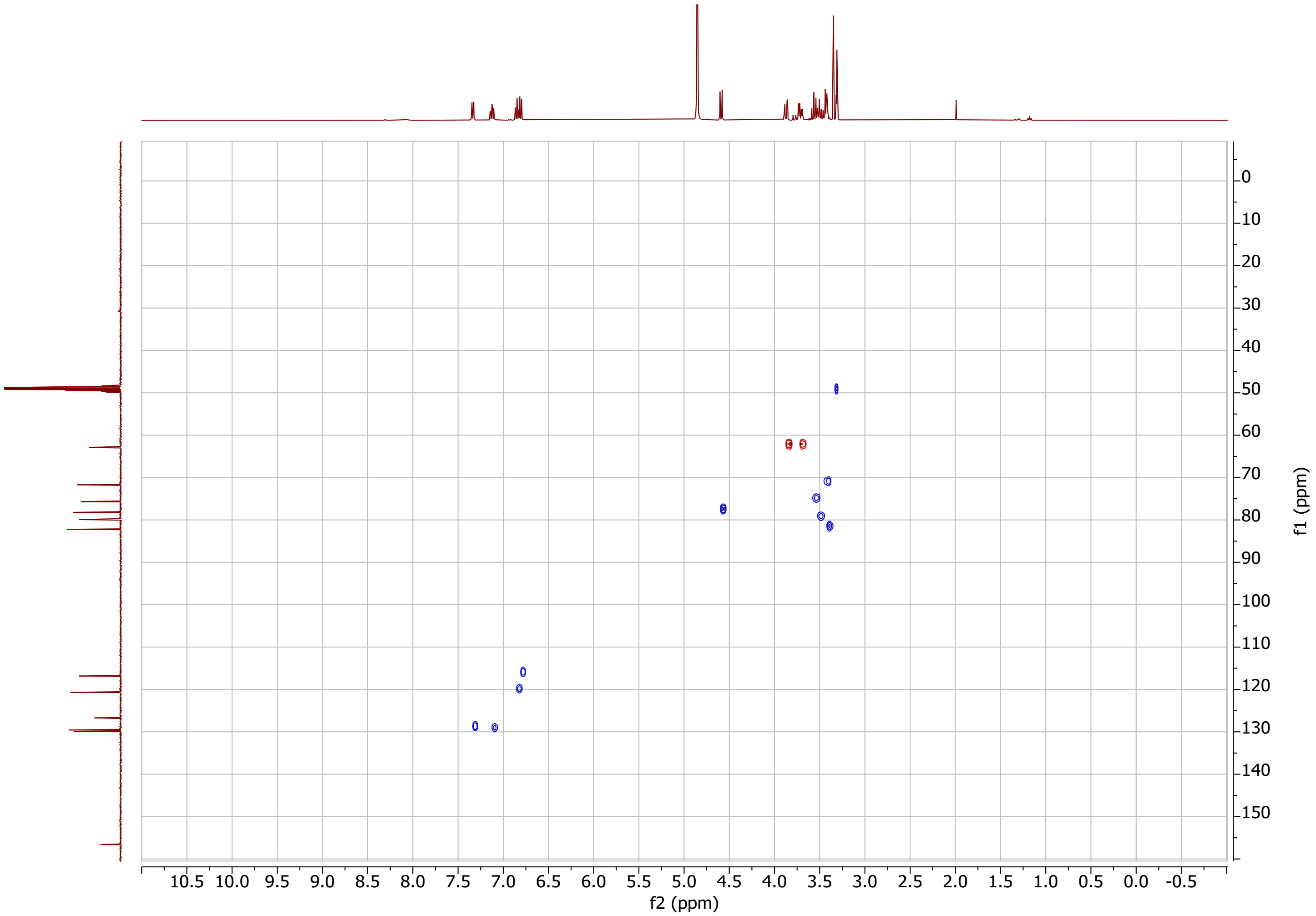

f2 (ppm)
f1 (ppm)

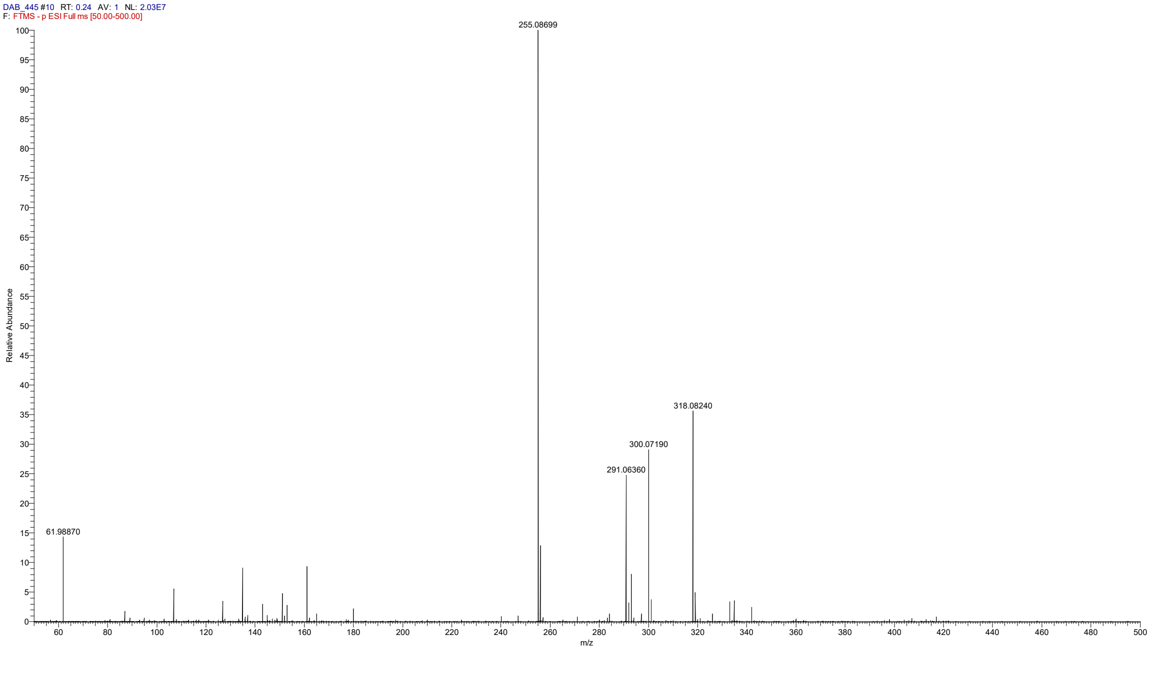

DAB_445 #10 RT: 0.24 AV: 1 NL: 2.03E7
F: FTMS - p ESI Full ms [50.00-500.00]
255.08699
318.08240
300.07190
291.06360
61.98870
Relative Abundance
m/z

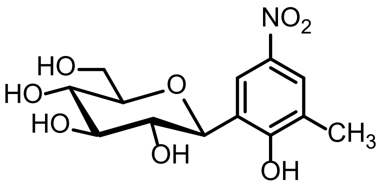
NO2
HO
HO
HO
O
OH
OH
CH3

23

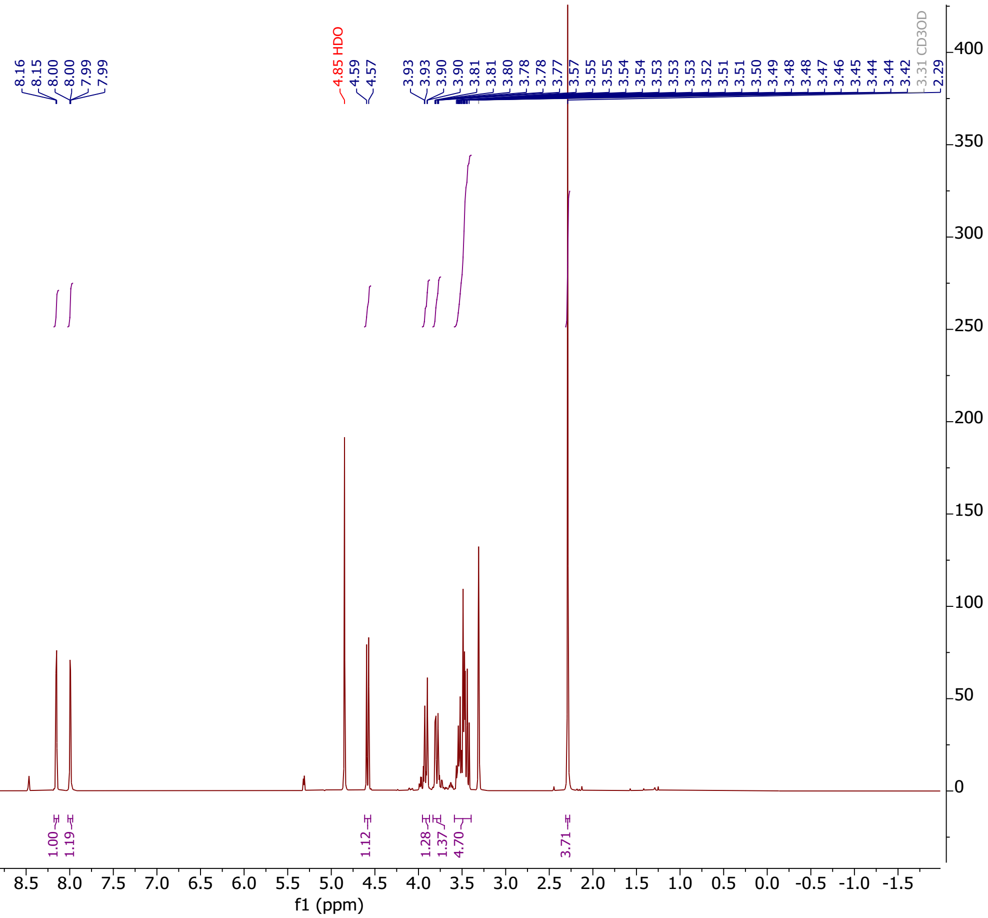
8.16
8.15
8.00
8.00
7.99
7.99
4.85 HDO
4.59
4.57
3.93
3.93
3.90
3.90
3.81
3.81
3.80
3.78
3.78
3.77
3.57
3.55
3.55
3.54
3.54
3.53
3.53
3.53
3.52
3.51
3.51
3.50
3.49
3.48
3.48
3.48
3.47
3.46
3.45
3.44
3.44
3.42
3.31 CD3OD
2.29
1.00
1.19
1.12
1.28
1.37
4.70
3.71
f1 (ppm)

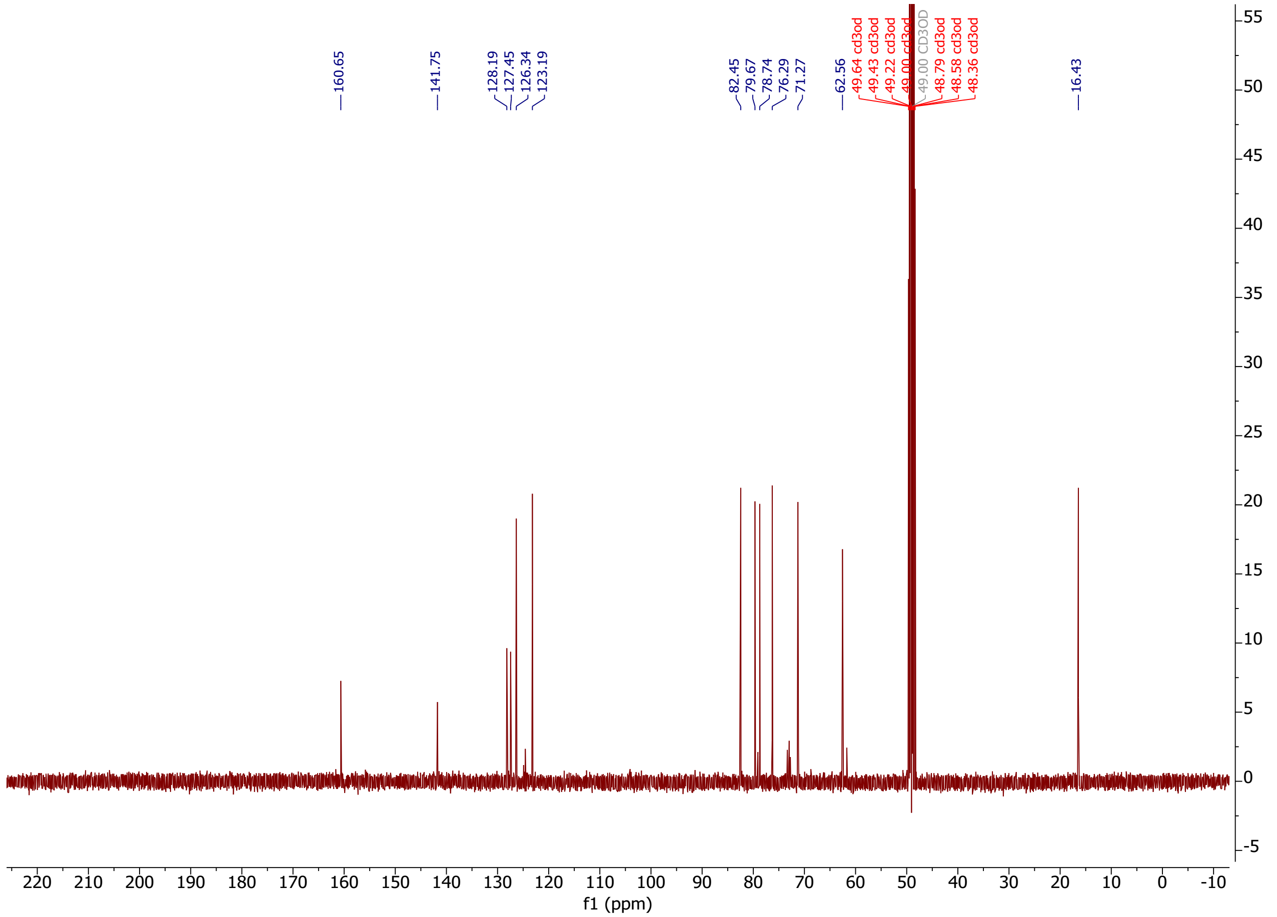
160.65
141.75
128.19
127.45
126.34
123.19
82.45
79.67
78.74
76.29
71.27
62.56
49.64 cd3od
49.43 cd3od
49.22 cd3od
49.00 cd3od
49.00 CD3OD
48.79 cd3od
48.58 cd3od
48.36 cd3od
16.43
f1 (ppm)

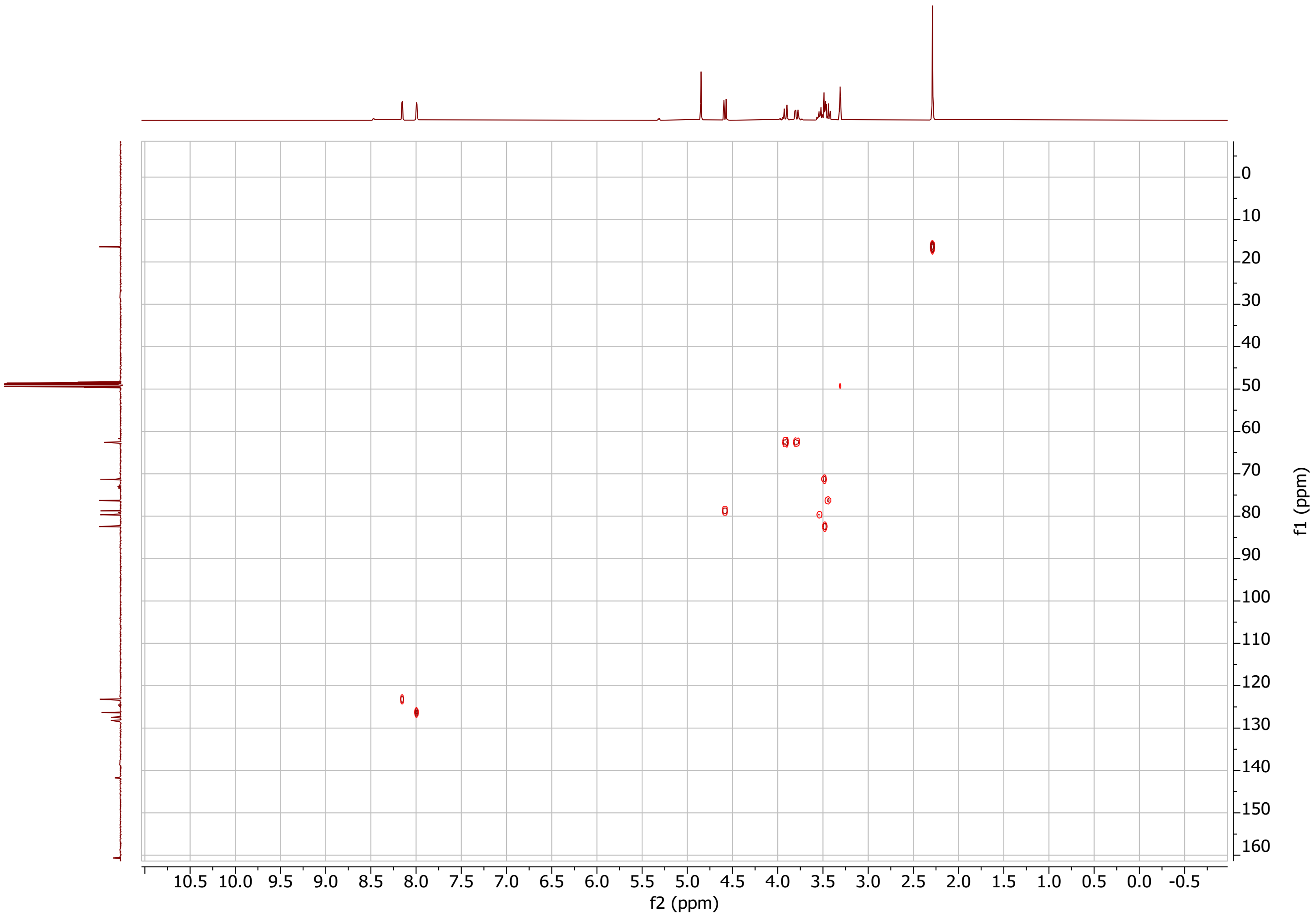

f2 (ppm)
f1 (ppm)

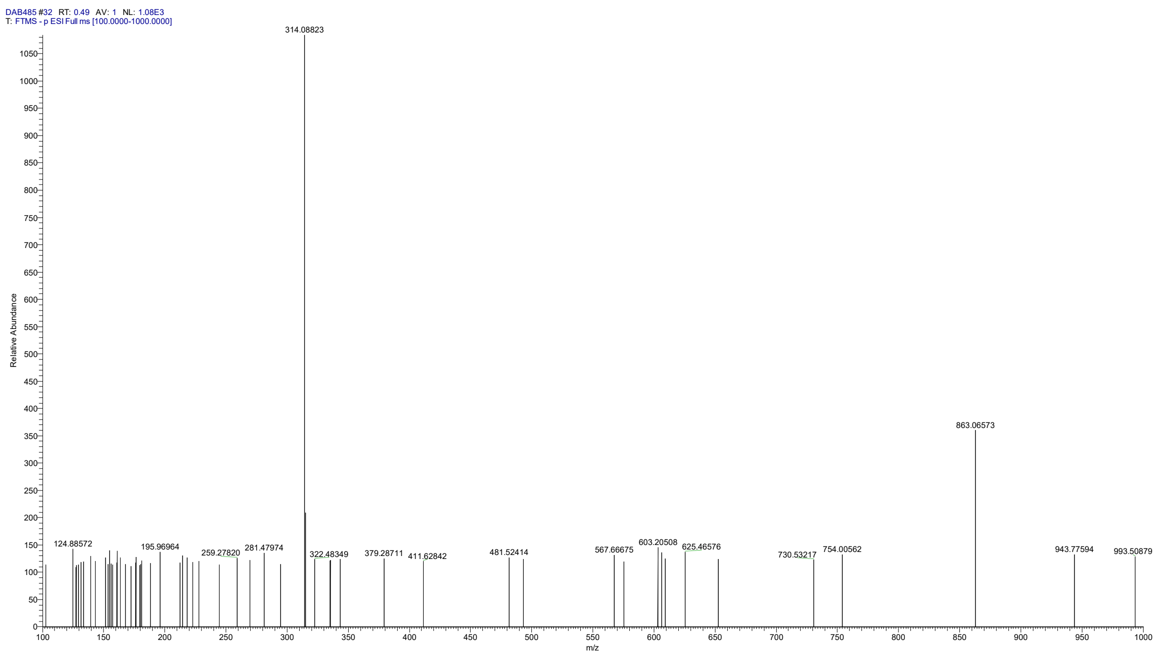

DAB485 #32 RT: 0.49 AV: 1 NL: 1.08E3
T: FTMS - p ESI Full ms [100.0000-1000.0000]
314.08823
863.06573
124.88572
195.96964
259.27820
281.47974
322.48349
379.28711
411.62842
481.52414
567.66675
603.20508
625.46576
730.53217
754.00562
943.77594
993.50879
Relative Abundance
m/z

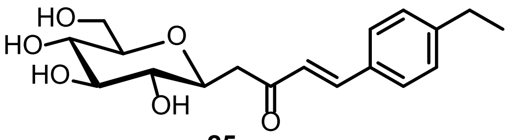
HO
HO
HO
O
OH
O

25

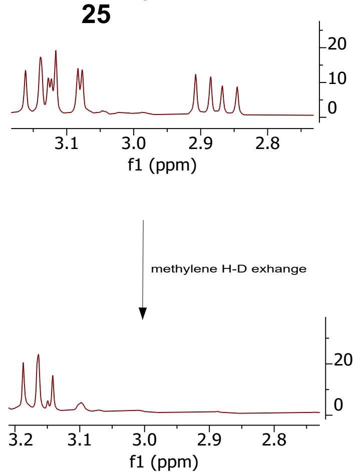
methylene H-D exhange
f1 (ppm)

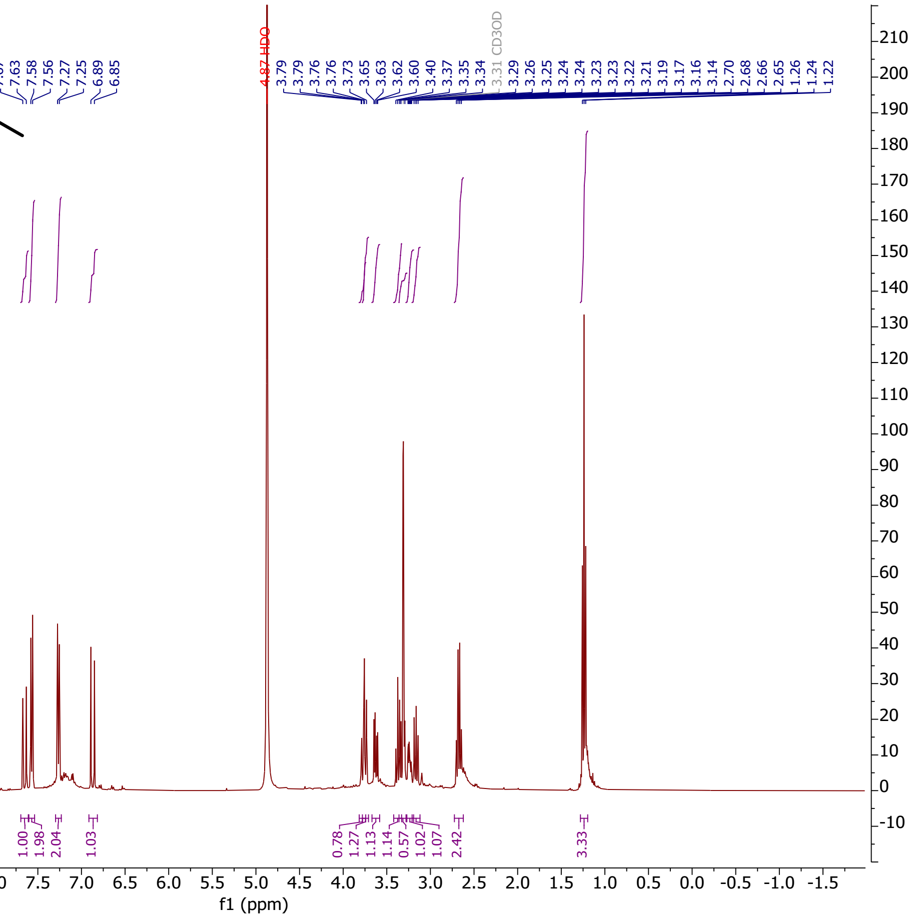
7.67
7.63
7.58
7.56
7.27
7.25
6.89
6.85
4.87 HDO
3.79
3.79
3.76
3.76
3.73
3.65
3.63
3.62
3.60
3.40
3.37
3.35
3.34
3.31 CD3OD
3.29
3.26
3.25
3.24
3.24
3.23
3.23
3.22
3.21
3.19
3.17
3.16
3.14
2.70
2.68
2.66
2.65
1.26
1.24
1.22
1.00
1.98
2.04
1.03
0.78
1.27
1.13
1.14
0.57
1.02
1.07
2.42
3.33
f1 (ppm)

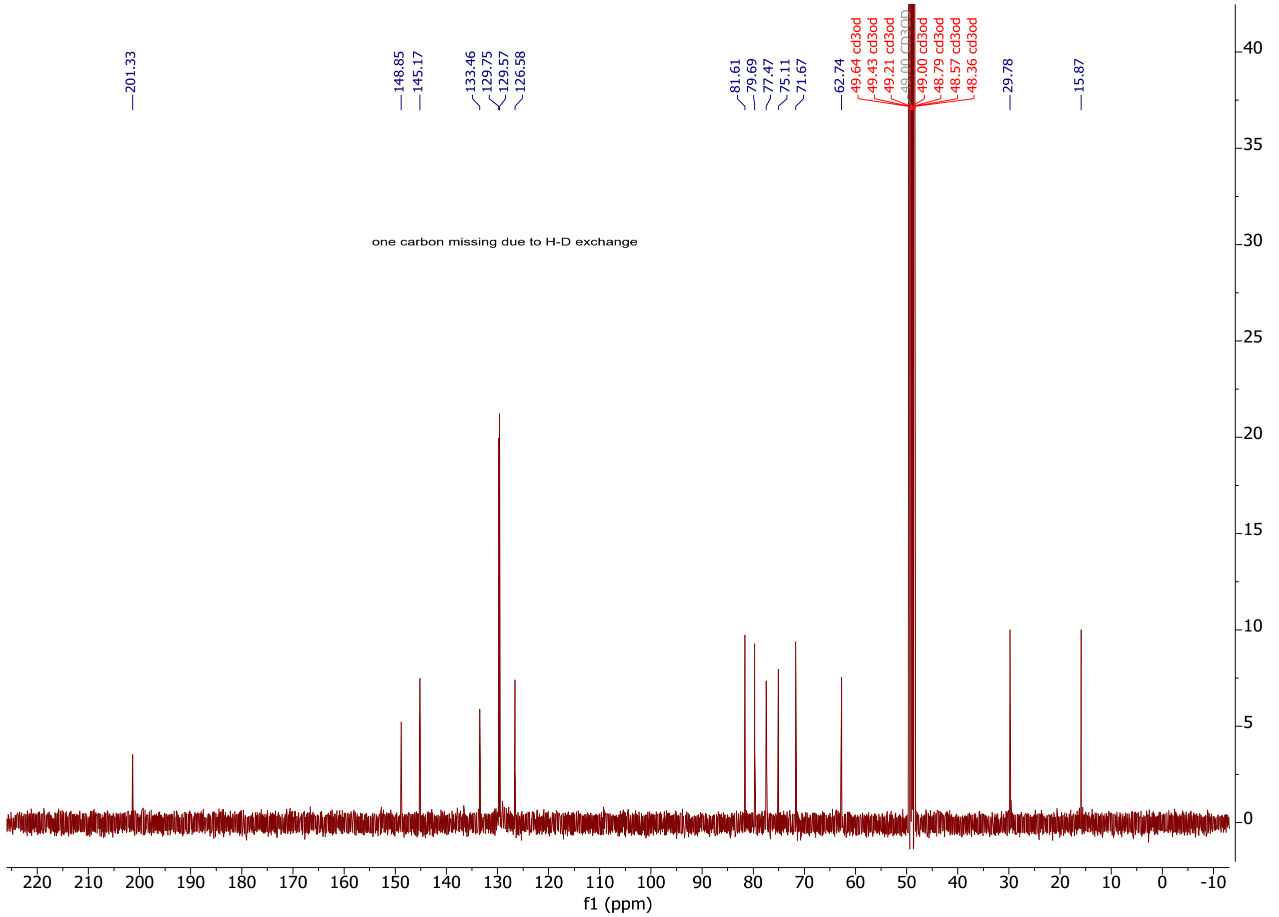
201.33
148.85
145.17
133.46
129.75
129.57
126.58
81.61
79.69
77.47
75.11
71.67
62.74
49.64 cd3od
49.43 cd3od
49.21 cd3od
49.00 CD3OD
49.00 cd3od
48.79 cd3od
48.57 cd3od
48.36 cd3od
29.78
15.87
one carbon missing due to H-D exchange
f1 (ppm)

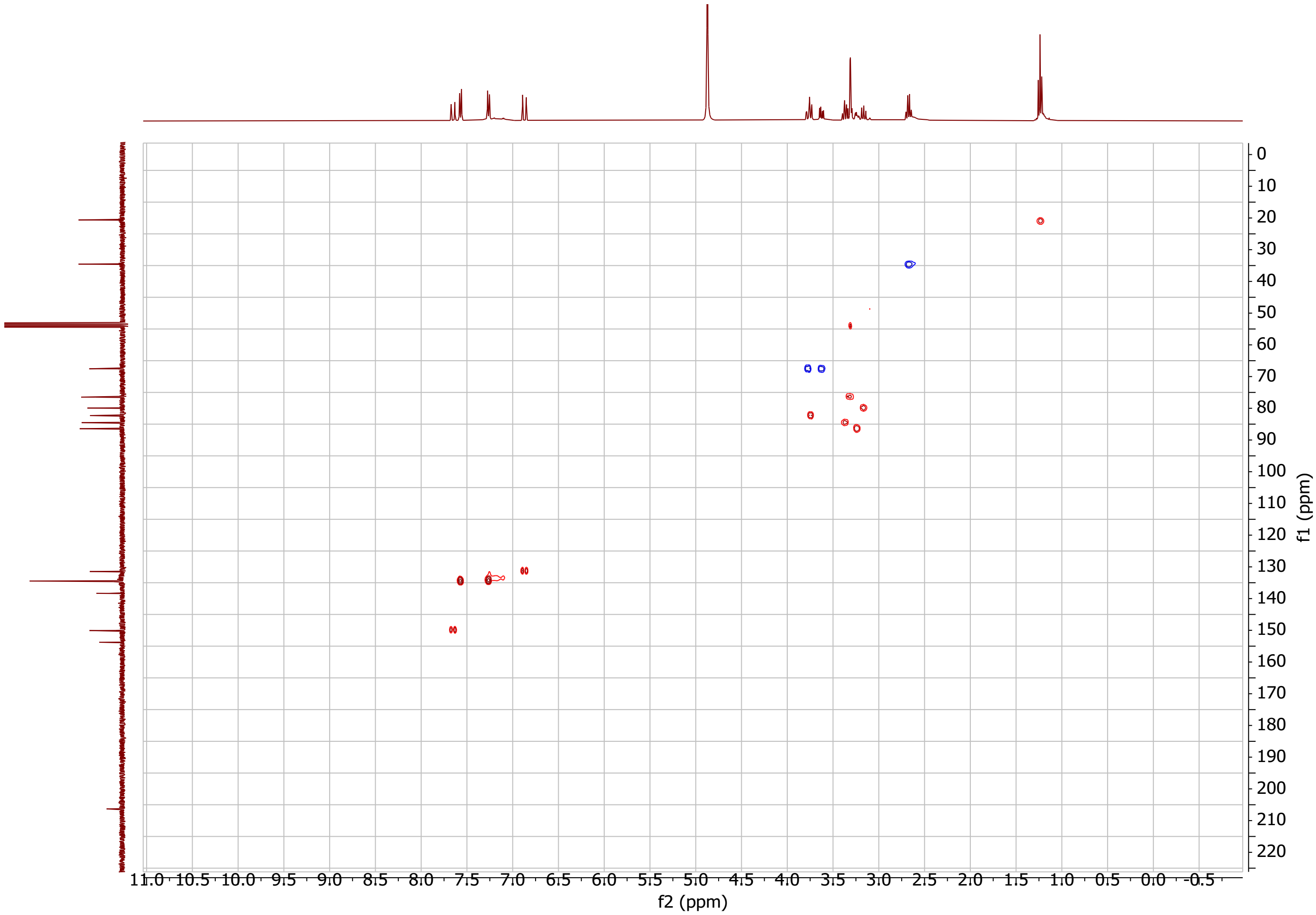
f2 (ppm)
f1 (ppm)

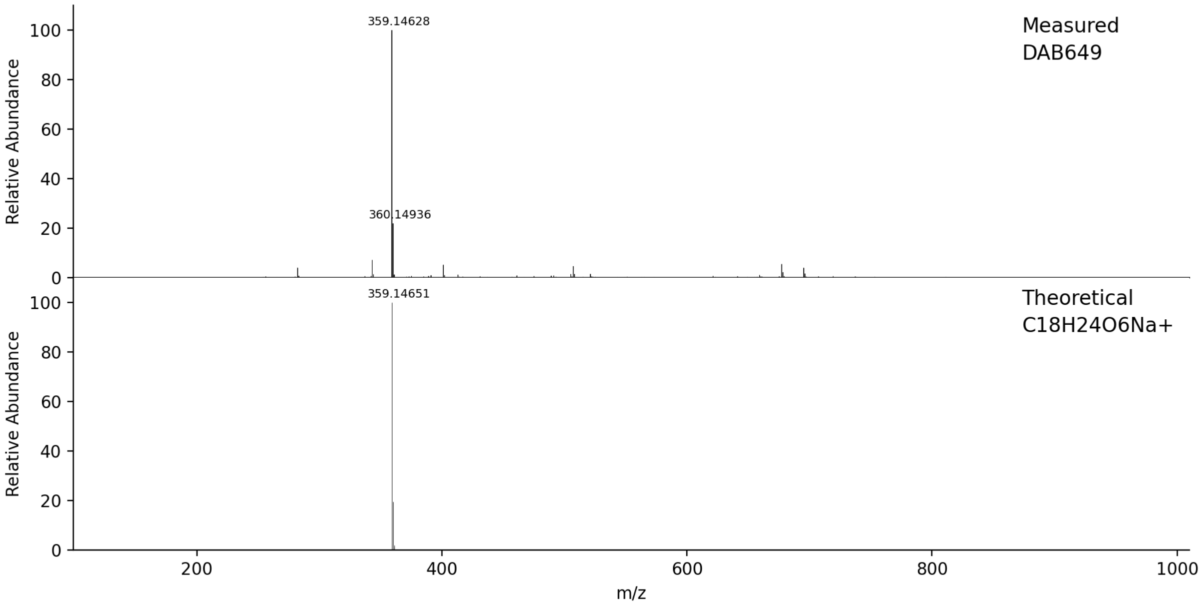
Measured
DAB649
359.14628
360.14936
Theoretical
C18H24O6Na+
359.14651
Relative Abundance
m/z

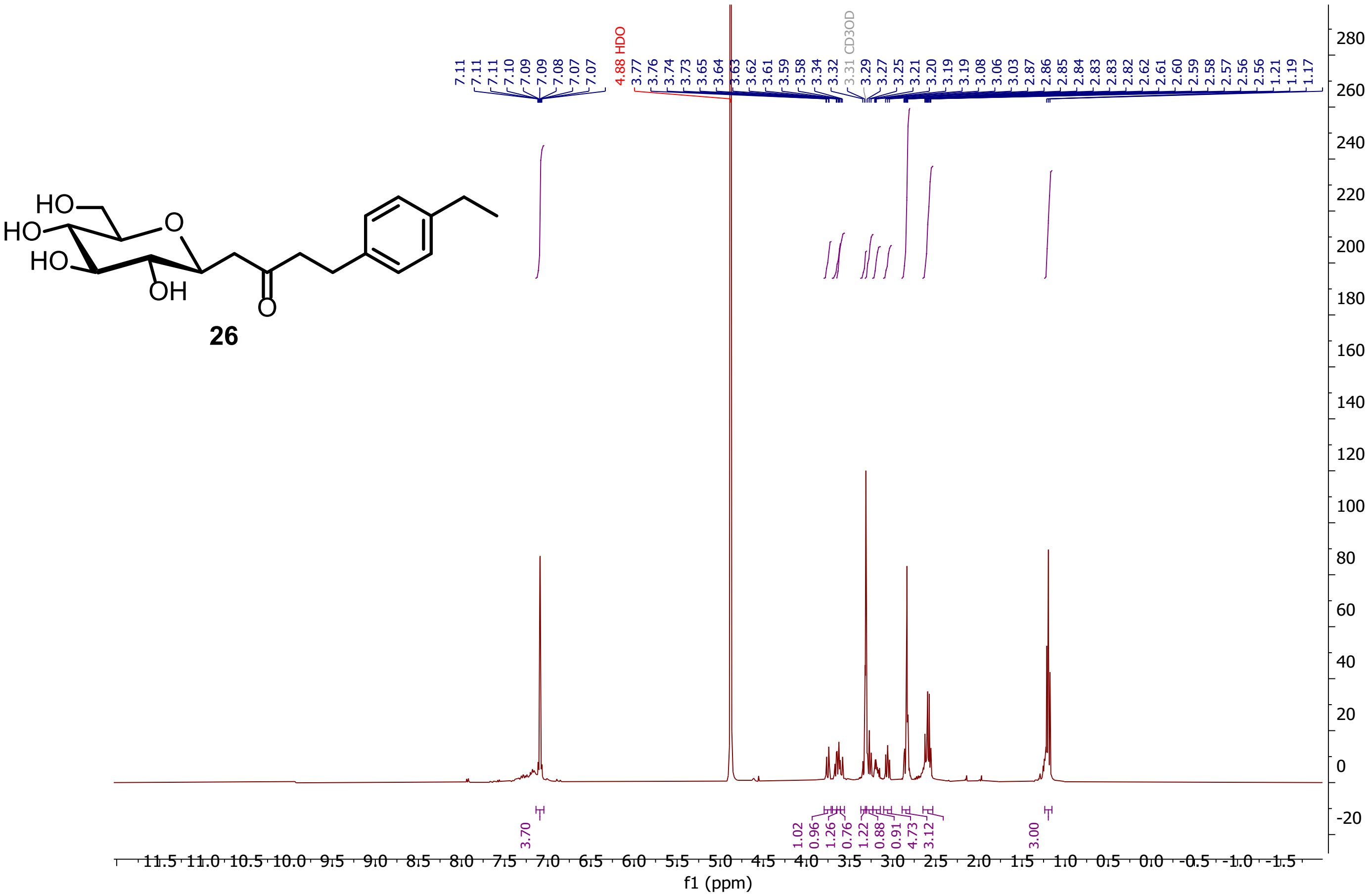
26
f1 (ppm)

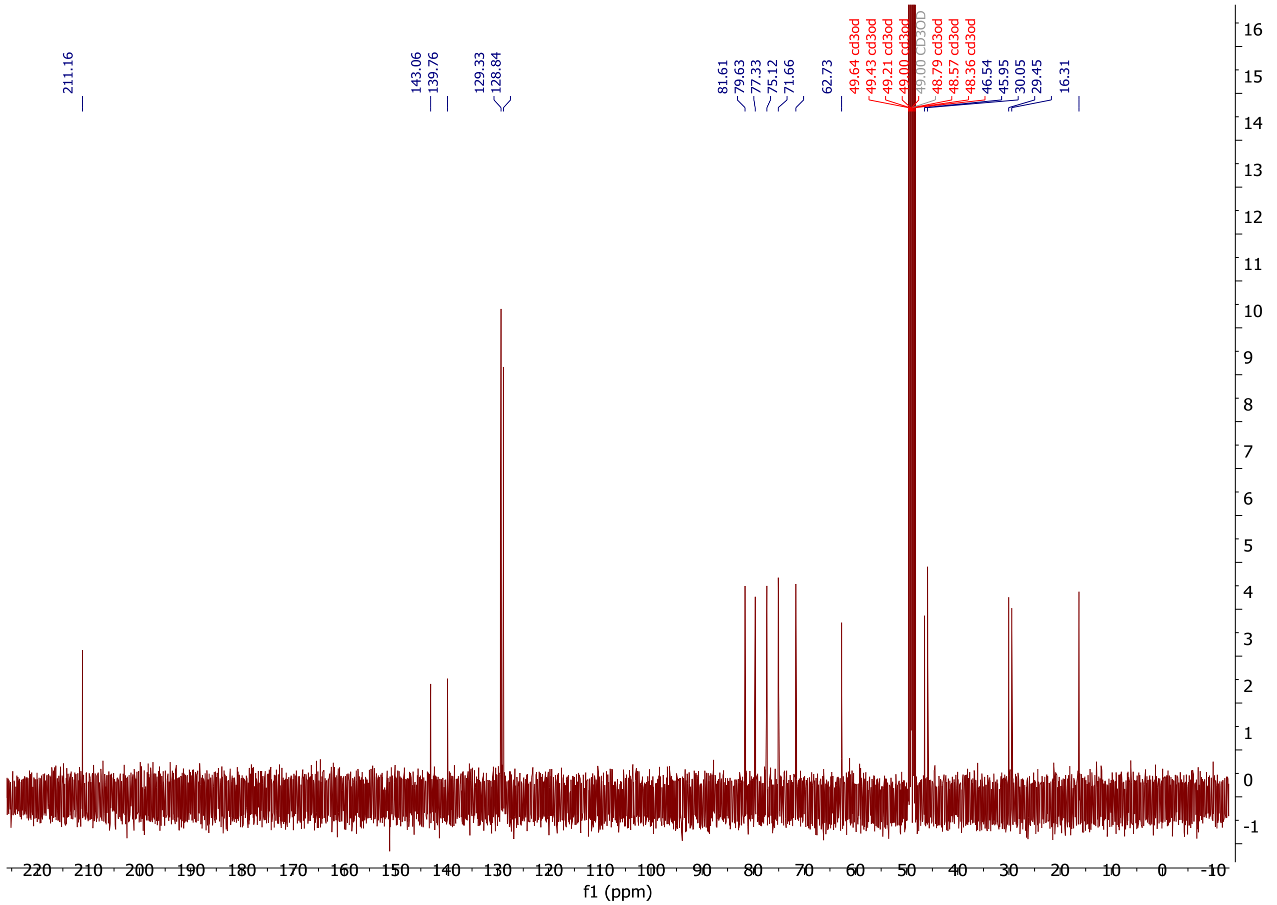
f1 (ppm)

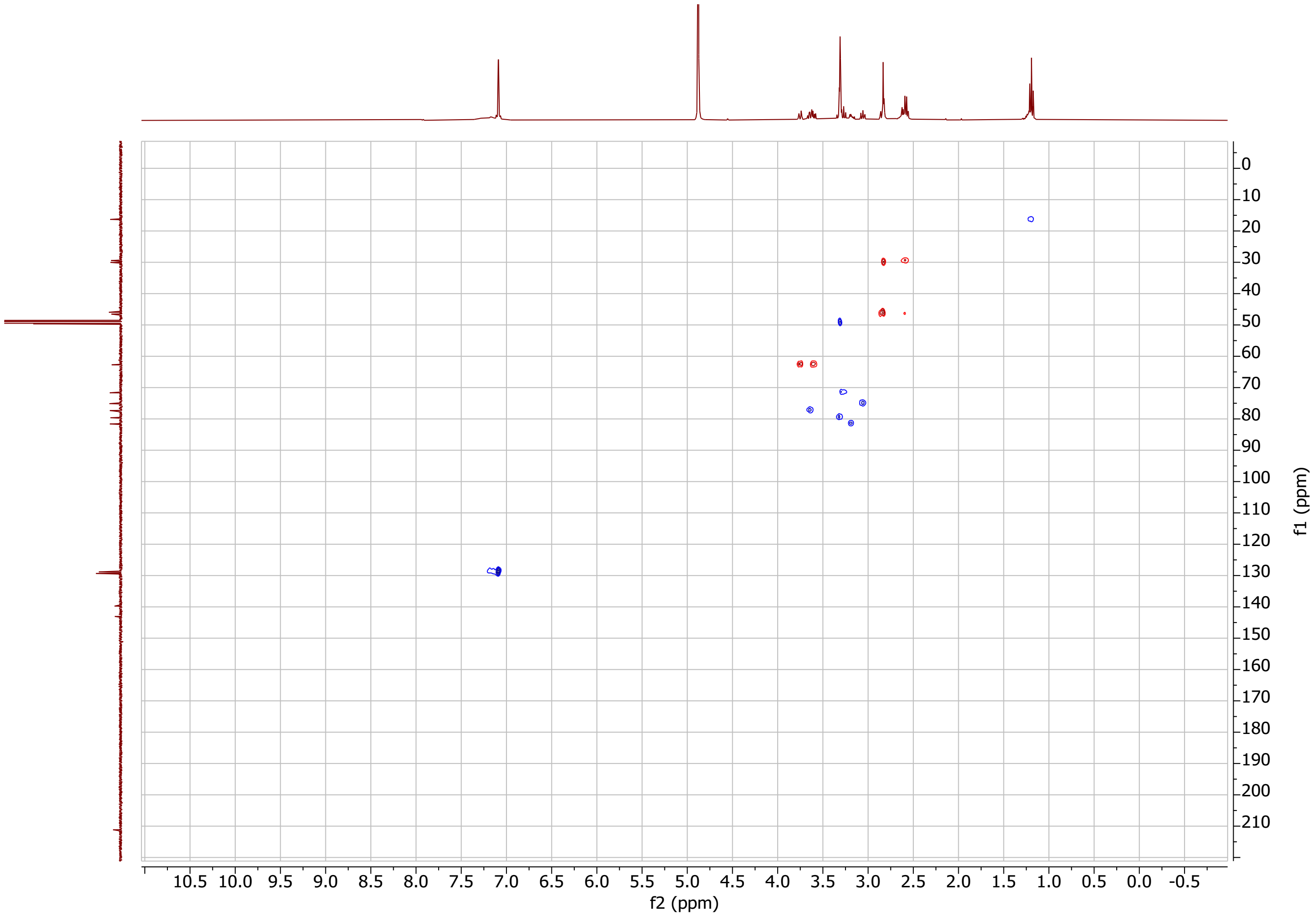
f2 (ppm)
f1 (ppm)

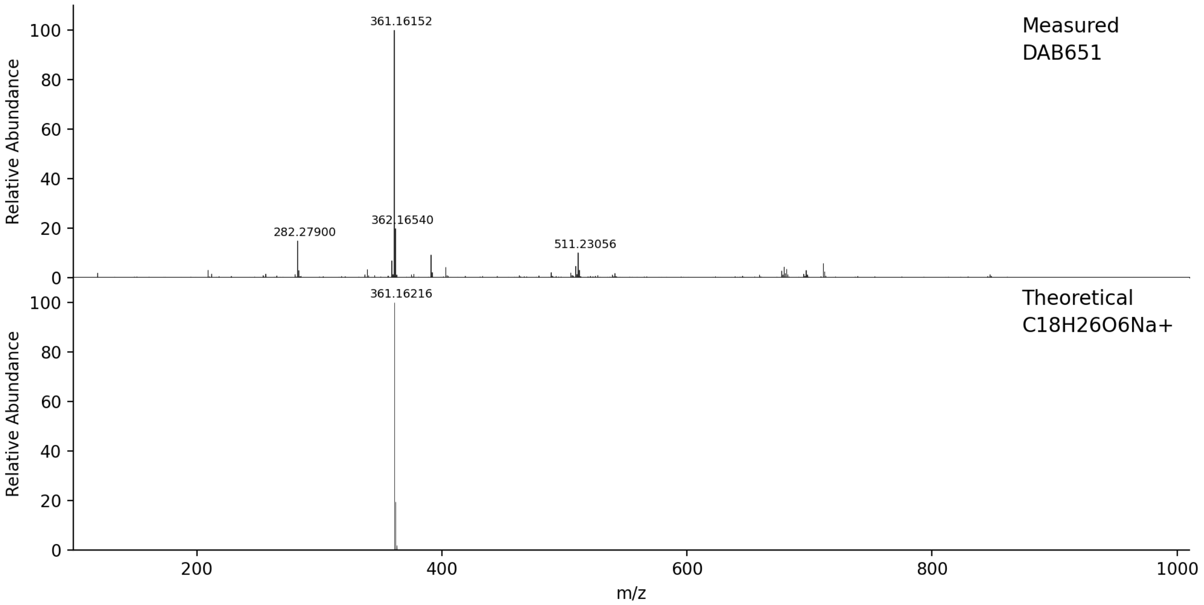
Measured
DAB651
361.16152
362.16540
282.27900
511.23056
Theoretical
C18H26O6Na+
361.16216
Relative Abundance
m/z

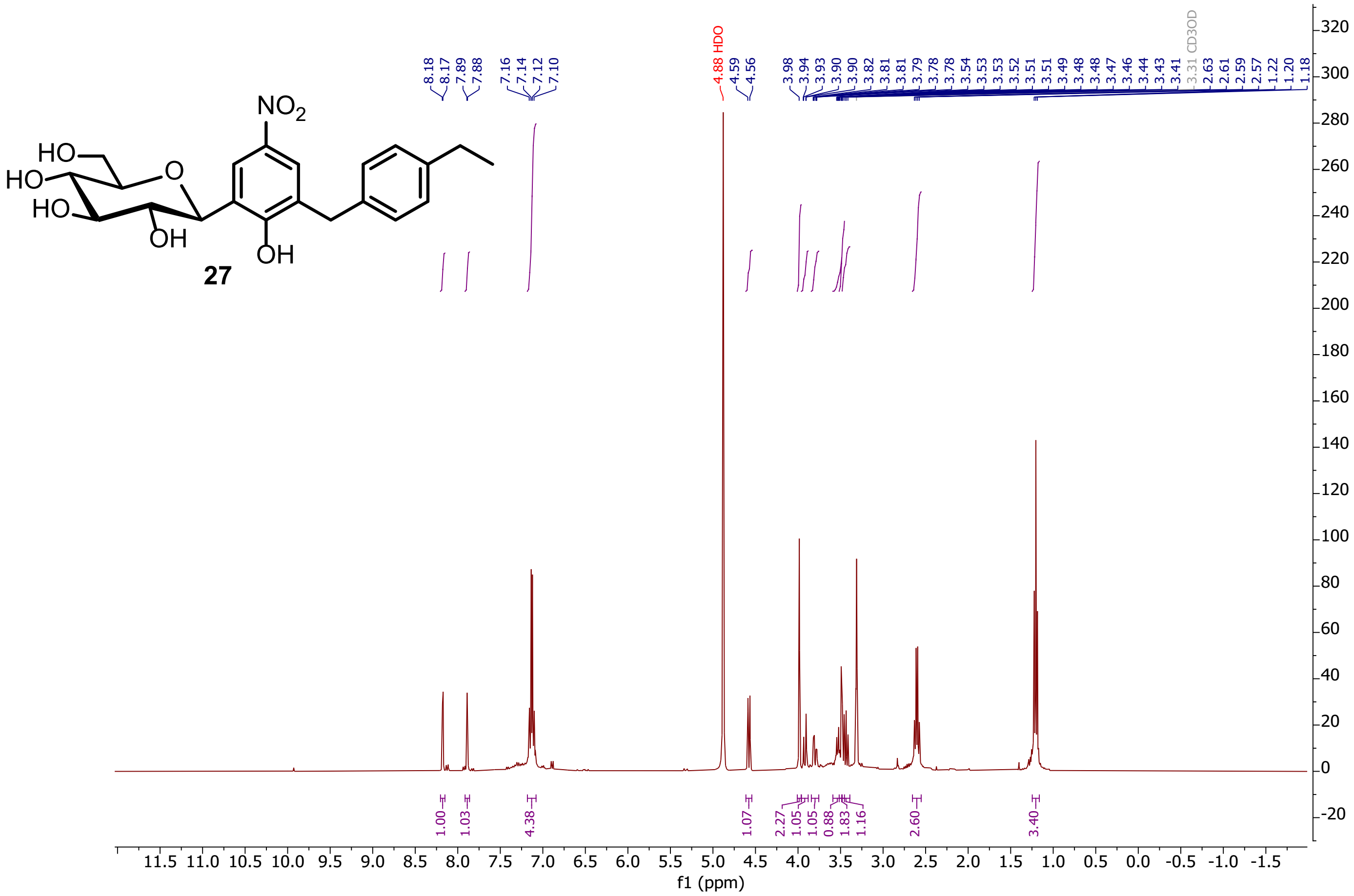

27
8.18
8.17
7.89
7.88
7.16
7.14
7.12
7.10
4.88 HDO
4.59
4.56
3.98
3.94
3.93
3.90
3.90
3.82
3.81
3.81
3.79
3.78
3.78
3.54
3.53
3.53
3.52
3.51
3.51
3.49
3.48
3.48
3.47
3.46
3.44
3.43
3.41
3.31 CD3OD
2.63
2.61
2.59
2.57
1.22
1.20
1.18
1.00
1.03
4.38
1.07
2.27
1.05
1.05
0.88
1.83
1.16
2.60
3.40
f1 (ppm)

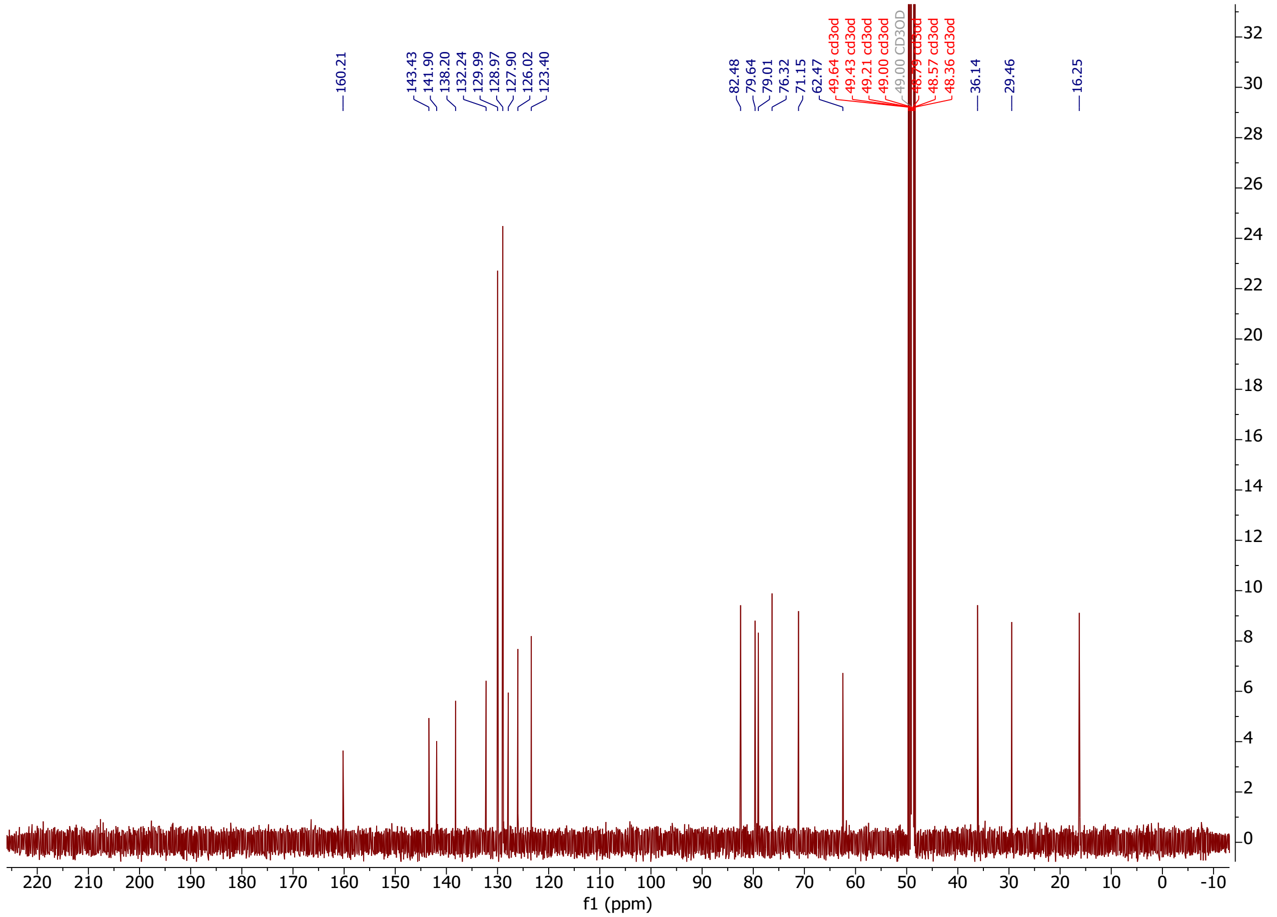
160.21
143.43
141.90
138.20
132.24
129.99
128.97
127.90
126.02
123.40
82.48
79.64
79.01
76.32
71.15
62.47
49.64 cd3od
49.43 cd3od
49.21 cd3od
49.00 CD3OD
48.79 cd3od
48.57 cd3od
48.36 cd3od
36.14
29.46
16.25
f1 (ppm)

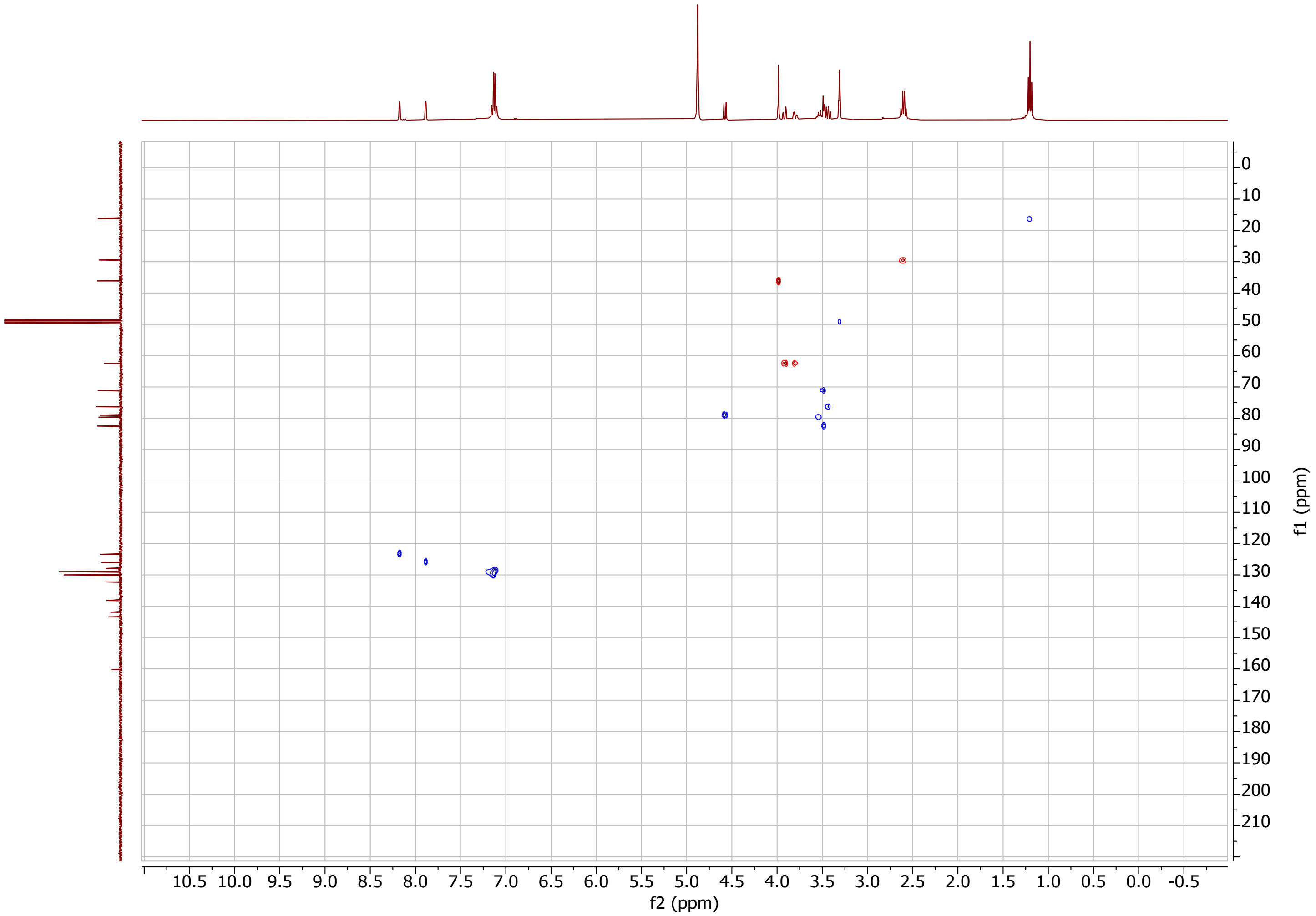
f2 (ppm)
f1 (ppm)

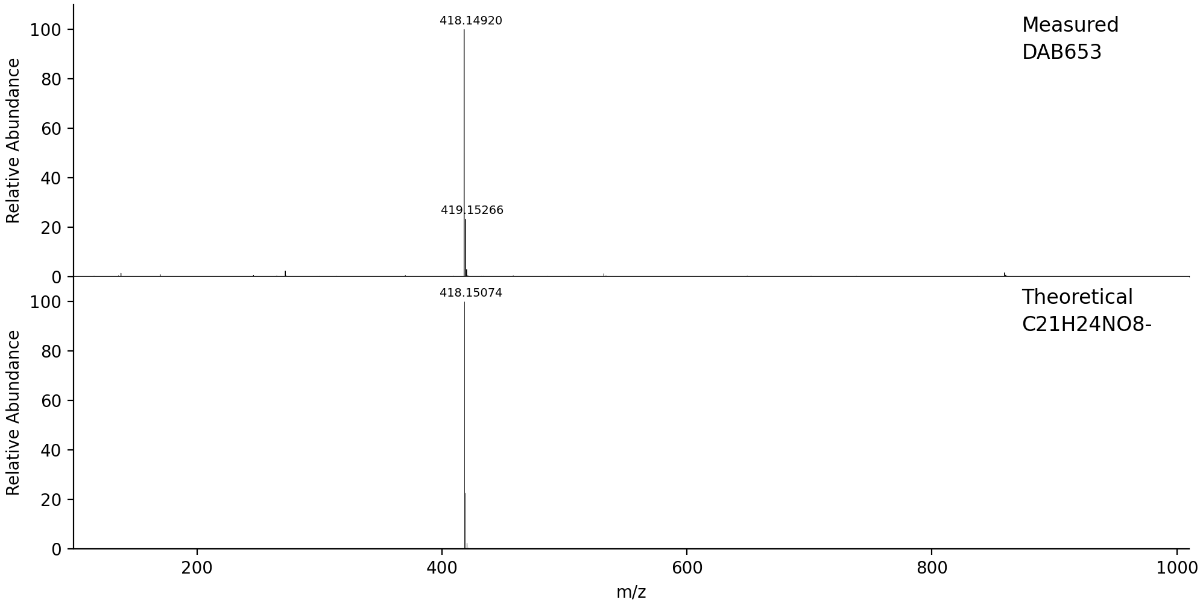
Measured
DAB653
418.14920
419.15266
Theoretical
C21H24NO8-
418.15074
Relative Abundance
m/z

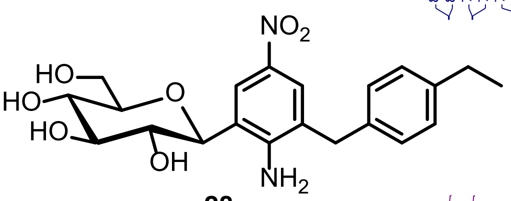
NO2
HO
HO
HO
O
OH
NH2

28

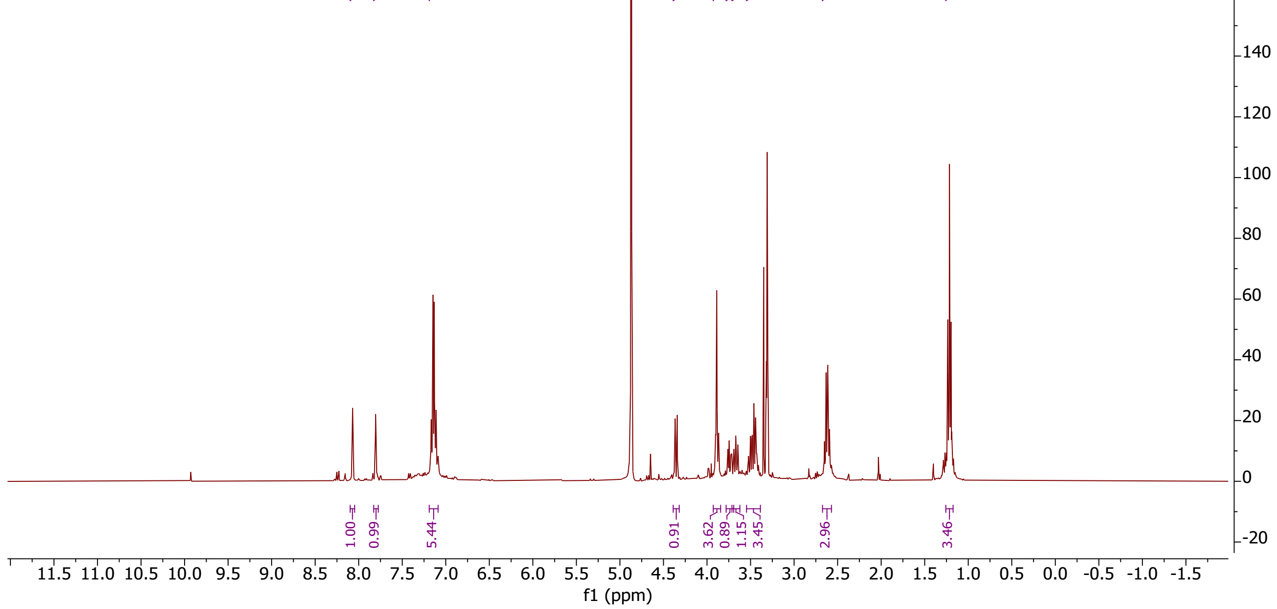

8.08
8.07
7.81
7.80
7.17
7.16
7.15
7.15
7.13
7.12
7.12
7.11
4.87 HDO
4.37
4.34
3.91
3.90
3.90
3.89
3.87
3.87
3.76
3.75
3.73
3.72
3.69
3.67
3.67
3.65
3.50
3.50
3.49
3.48
3.48
3.46
3.45
3.44
3.44
3.43
3.31 CD3OD
2.65
2.63
2.61
2.59
1.24
1.22
1.20
1.00
0.99
5.44
0.91
3.62
0.89
1.15
3.45
2.96
3.46
f1 (ppm)

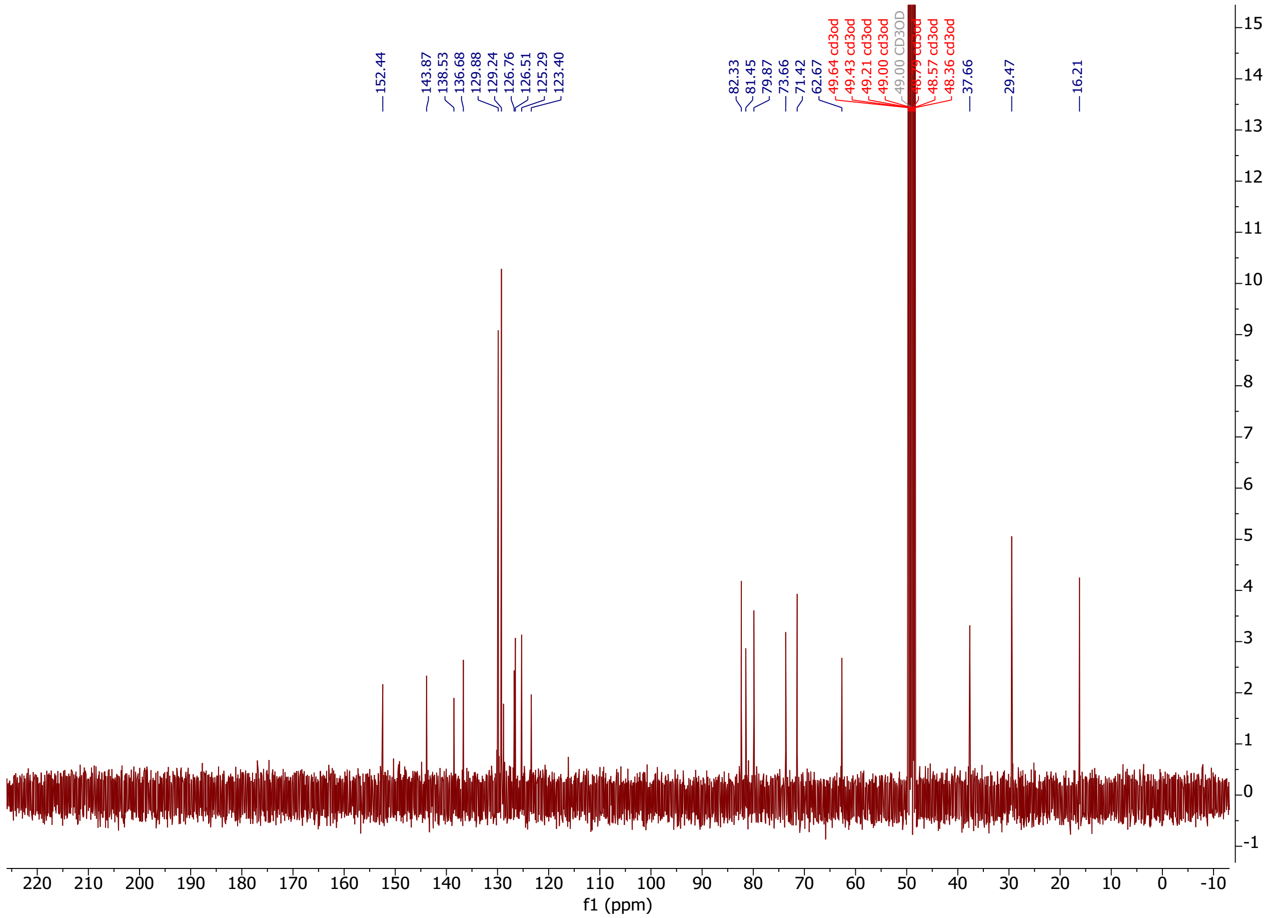
152.44
143.87
138.53
136.68
129.88
129.24
126.76
126.51
125.29
123.40
82.33
81.45
79.87
73.66
71.42
62.67
49.64 cd3od
49.43 cd3od
49.21 cd3od
49.00 CD3OD
48.79 cd3od
48.57 cd3od
48.36 cd3od
37.66
29.47
16.21
f1 (ppm)

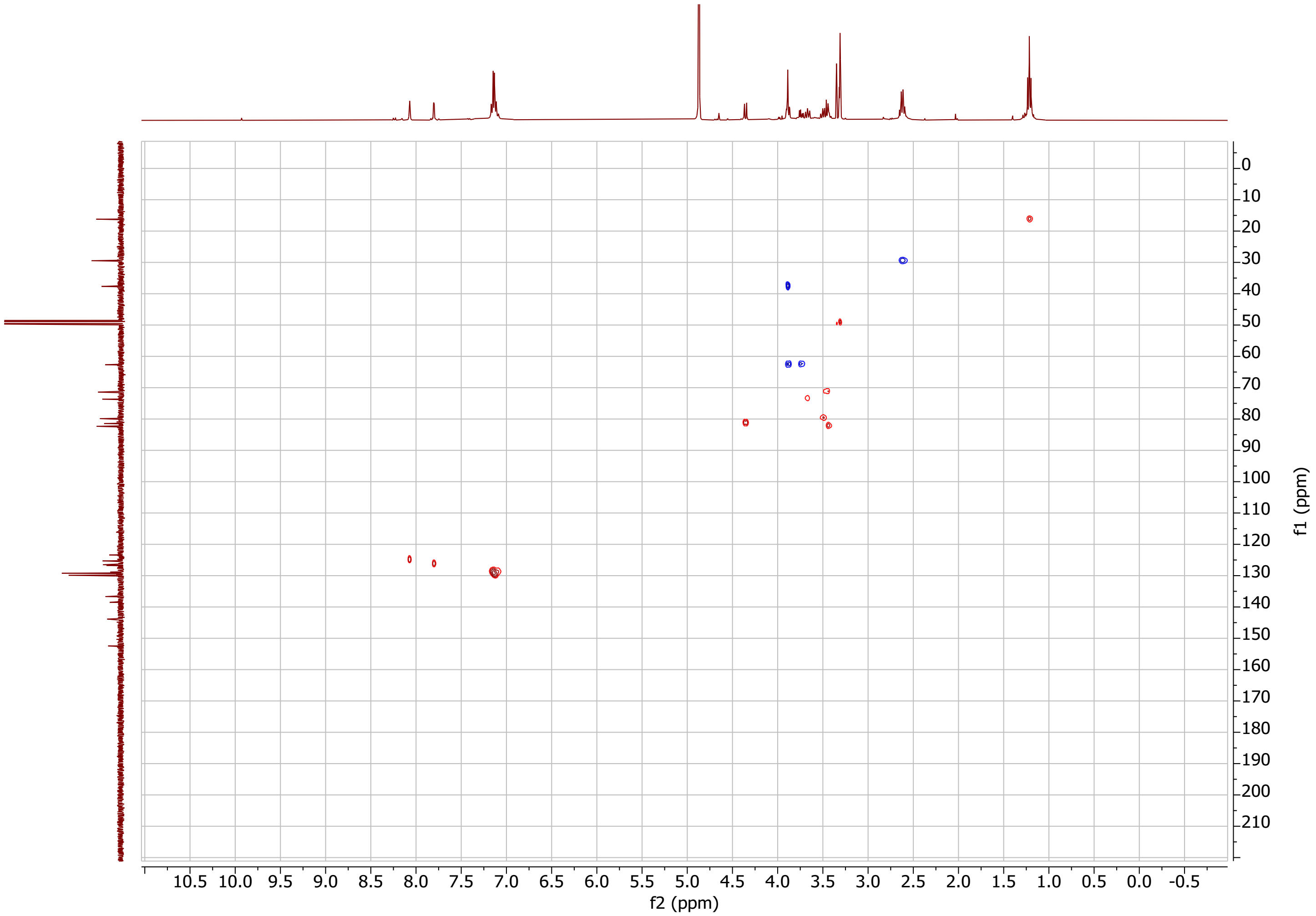
f2 (ppm)
f1 (ppm)

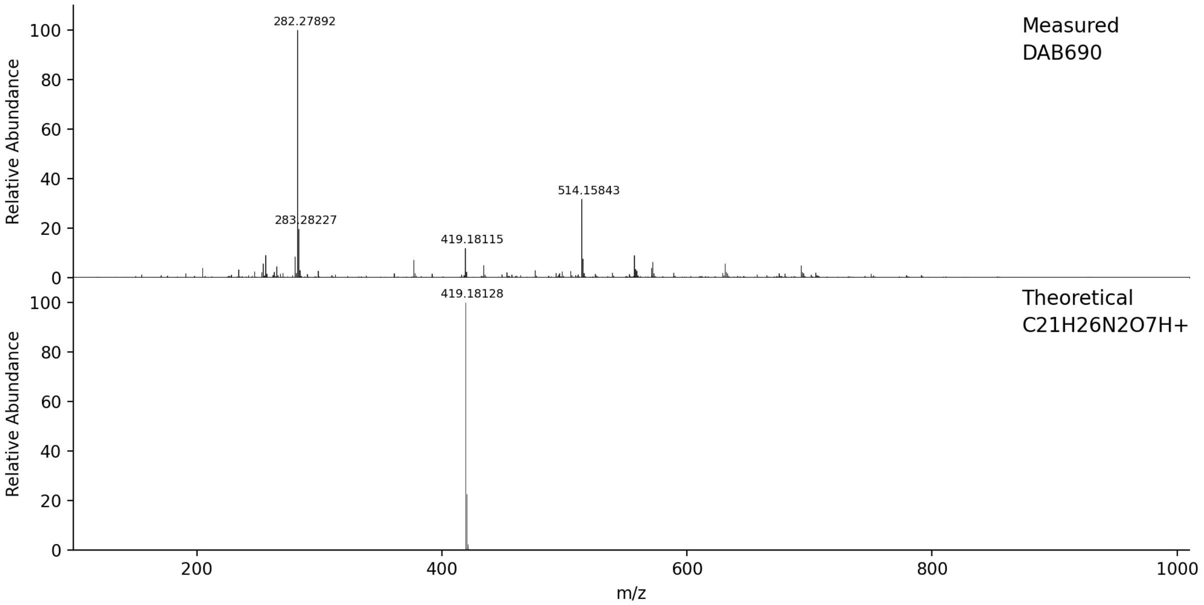
282.27892
283.28227
419.18115
514.15843
Measured
DAB690
419.18128
Theoretical
C21H26N2O7H+
Relative Abundance
m/z

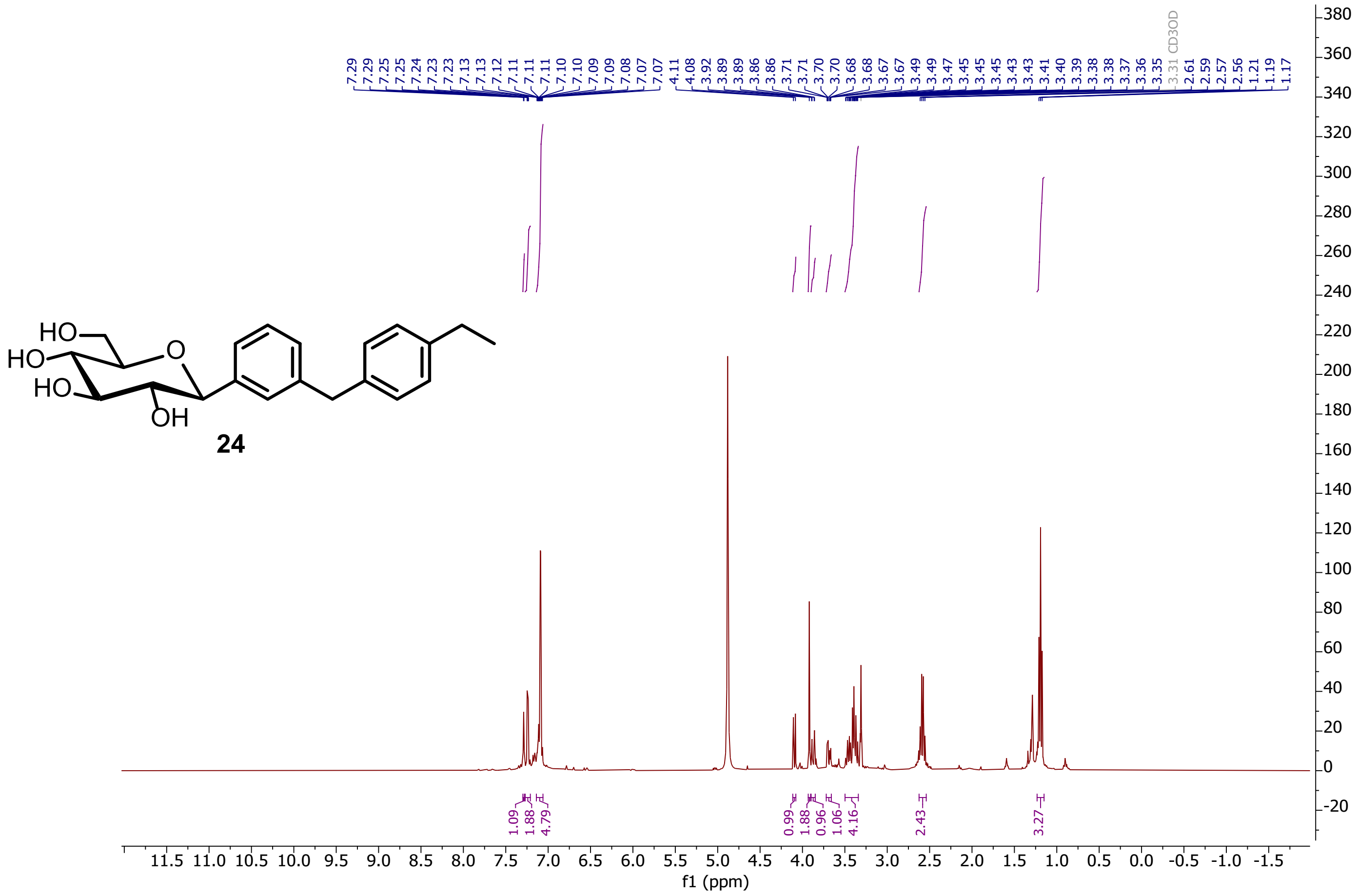
7.29 7.29 7.25 7.24 7.23 7.23 7.13 7.13 7.12 7.11 7.11 7.11 7.10 7.10 7.09 7.09 7.08 7.07 7.07
4.11 4.08 3.92 3.89 3.89 3.86 3.86 3.71 3.71 3.70 3.70 3.68 3.68 3.67 3.67 3.49 3.49 3.47 3.45 3.45 3.45 3.43 3.43 3.41 3.40 3.39 3.38 3.38 3.37 3.36 3.35 3.31 CD3OD 2.61 2.59 2.57 2.56 1.21 1.19 1.17
1.09 1.88 4.79 0.99 1.88 0.96 1.06 4.16 2.43 3.27
f1 (ppm)
HO
HO
HO
O
OH

24

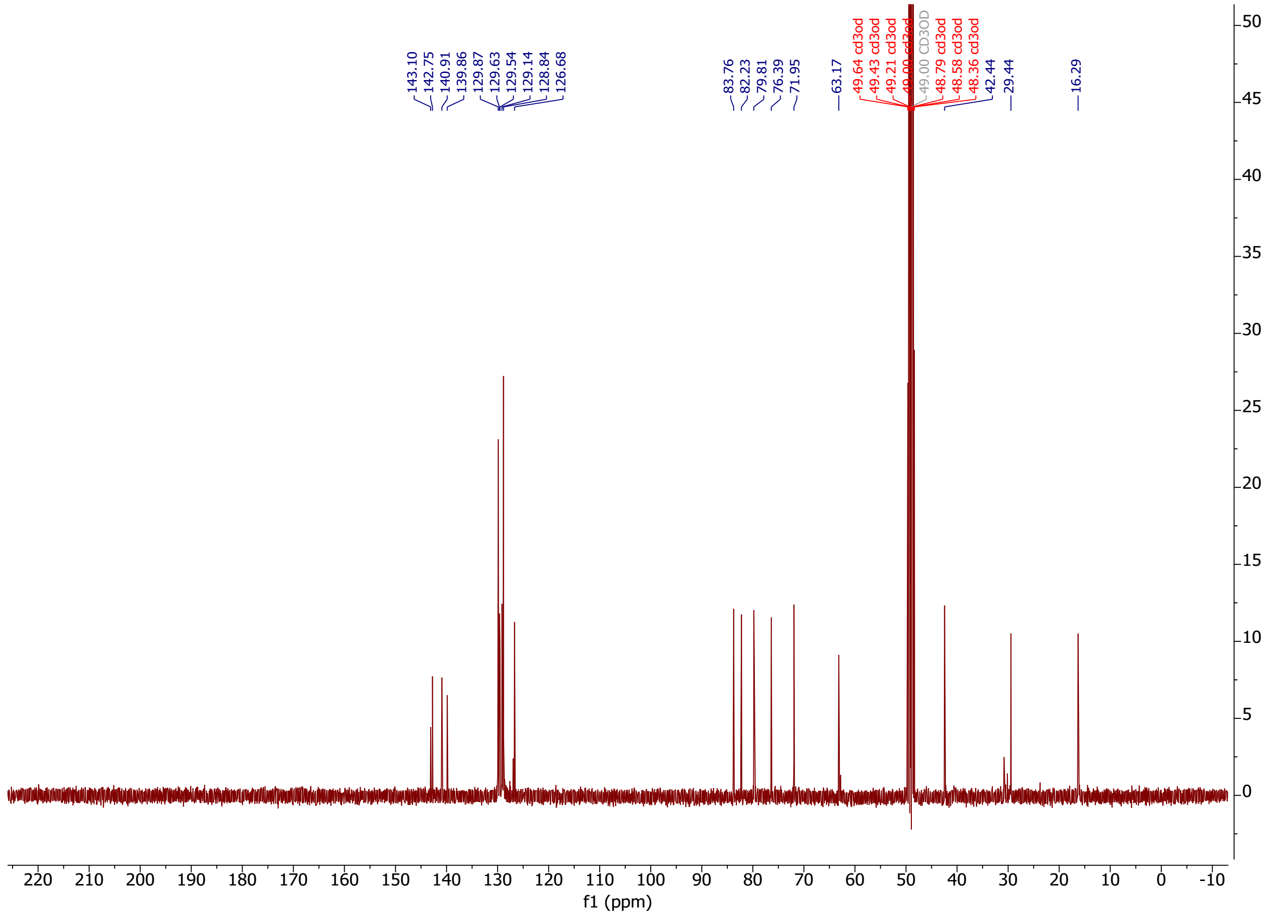
143.10 142.75 140.91 139.86 129.87 129.63 129.54 129.14 128.84 126.68
83.76 82.23 79.81 76.39 71.95 63.17
49.64 cd3od 49.43 cd3od 49.21 cd3od 49.00 cd3od 49.00 CD3OD 48.79 cd3od 48.58 cd3od 48.36 cd3od
42.44 29.44 16.29
f1 (ppm)

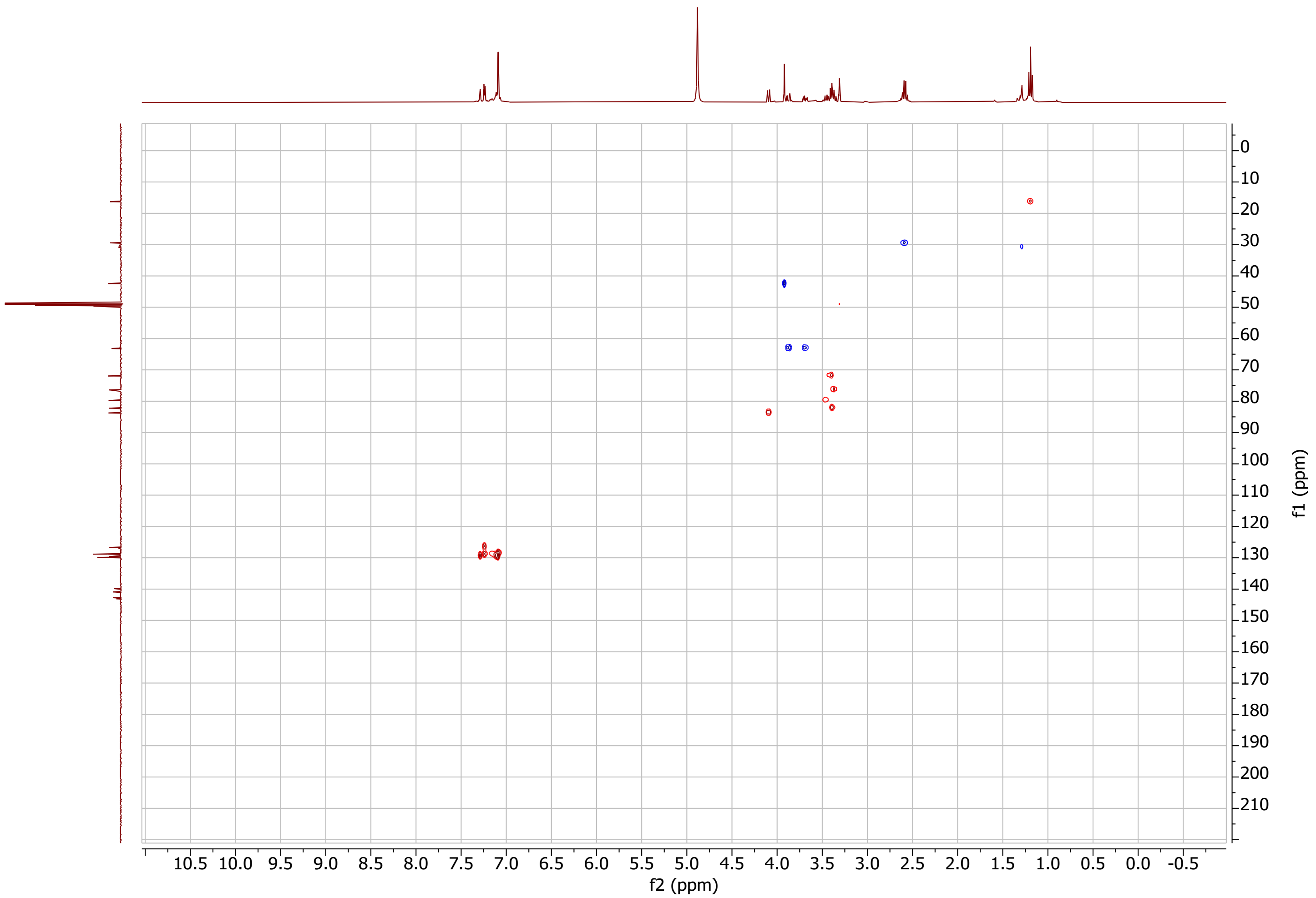
f2 (ppm)
f1 (ppm)

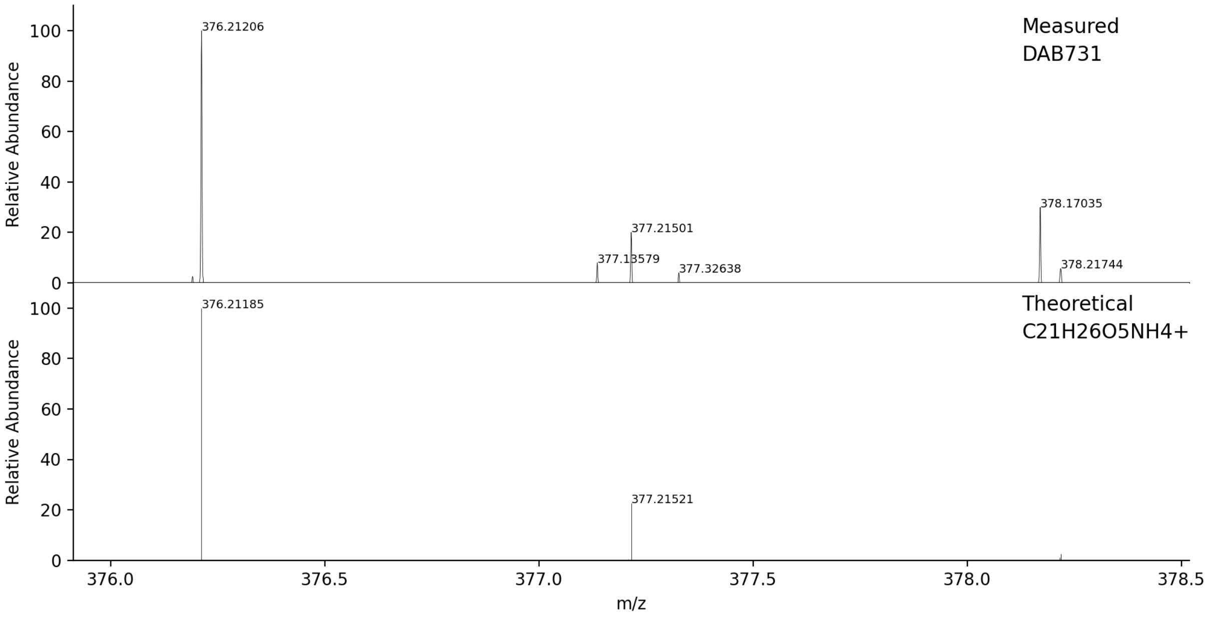
Measured
DAB731
376.21206
377.13579
377.21501
377.32638
378.17035
378.21744
Theoretical
C21H26O5NH4+
376.21185
377.21521
Relative Abundance
m/z
